# Clinical practice guideline for psoriasis management in Latin America^؆^

**DOI:** 10.1016/j.abd.2026.501449

**Published:** 2026-08-29

**Authors:** Fernando Valenzuela, Juan Raúl Castro Ayarza, Débora Kaplan, Angela María Londoño-García, Karla del Rocío Macías García, Gabriel Magariños, Enrique Salvador Rivas Záldivar, Manuel Franco, Sara Cano, Carolina Cortés, Paola Jimena Cárdenas, Carla Castro, Renata Ferreira, Susan Martínez, Linda Ibatá Bernal, Julieth Carolina Castillo, Jorge Ospina

**Affiliations:** a Department of Dermatology, Faculty of Medicine, Universidad de Chile, Santiago, Chile; b Department of Dermatology, Centro Internacional de Estudios Clínicos, Santiago, Chile; c Postgraduate Program of Dermatology, Faculty of Medicine, Universidad Nacional de Colombia, Bogotá, Colombia; d Colombian Group of Psoriasis and Psoriatic Arthritis, Bogotá, Colombia; e Private practice, Buenos Aires, Argentina; f Postgraduate Program of Dermatology, Faculty of Medicine, Universidad CES, Medellín, Colombia; g Service of Dermatology, CLIPSO-HELPHARMA, Medellín, Colombia; h Service of Dermatology, Dermatologico Country, Guadalajara, Mexico; i Service of Dermatology, Hospital Municipal Dr. Bernardo Houssay, Buenos Aires, Argentina; j Clinical Research Area, Psoriahue, Buenos Aires, Argentina; k Department of Dermatology, Faculty of Medicine, Universidad del Salvador, Buenos Aires, Argentina; l Service of Dermatology, DERMOS Centro de Dermatología, Guatemala City, Guatemala; m Department of Dermatology, Faculty of Medicine, Universidad El Bosque, Bogotá, Colombia; n Department of Dermatology, Faculty of Medicine, Universidad ICESI, Cali, Colombia; o Service of Dermatology, Hospital Universitario La Samaritana, Bogotá, Colombia; p Service of Dermatology, Virrey Solis IPS, Bogotá, Colombia; q Department of Dermatology, Faculty of Medical Sciences, Universidad Católica de Cuyo, San Luis, Argentina; r Department of Dermatology and Pediatric, Faculty of Biomedical Sciences, Universidad Austral, Buenos Aires, Argentina; s Department of Dermatology, Faculty of Medicine, Universidade Estadual de Campinas, Campinas, Brazil; t Epidemiology Department, EpiThink Health Consulting, Bogotá, Colombia

**Keywords:** Biological products, Biosimilar pharmaceuticals, Drug therapy, Latin America, Practice guidelines, Psoriasis, Treatment outcome

## Abstract

**Background:**

Psoriasis care in Latin America is heterogeneous, and access to systemic agents, biologics, phototherapy, and monitoring resources varies widely across countries.

**Objective:**

To develop a regional, evidence-based clinical practice guideline for psoriasis management using GRADE methodology.

**Methods:**

The Latin American Psoriasis Society (SOLAPSO) convened a guideline development group, an extended expert panel, patients, and an independent methodological team. We framed clinical questions using PICO, conducted systematic literature reviews, rated certainty of evidence with GRADE, and formulated recommendations through structured Evidence-to-Decision deliberations incorporating benefits and harms, patient values, feasibility, equity, and resource use. Independent external reviewers assessed clarity and methodological quality.

**Results:**

The guideline provides measurable therapeutic targets for plaque psoriasis and key phenotypes, including nail psoriasis, generalized pustular psoriasis, and other special forms. It recommends first-line conventional systemic options (methotrexate, cyclosporine, acitretin) and phototherapy, and it defines indications for escalation to biologic agents or small molecules after inadequate response, intolerance, or contraindications to conventional therapy. It addresses selection across biologic classes, use of biosimilars, and strategies for primary and secondary therapeutic failure. Additional recommendations cover high-impact areas (scalp, nails, palmoplantar, inverse, genital) and special situations (pregnancy, cancer, chronic viral infections, HIV, latent tuberculosis, pediatric psoriasis), plus nonpharmacologic interventions (weight reduction and structured psychological support).

**Study limitations:**

Limitations include heterogeneity in the availability of regional evidence, indirect evidence for some special populations, and variability in access to therapies across Latin American health systems.

**Conclusions:**

This guideline offers an actionable framework for comprehensive psoriasis care in Latin America, balancing best available evidence with regional constraints to support equitable, patient-centered implementation in routine clinical practice.

## Introduction

Psoriasis is a chronic immune-mediated inflammatory disease with clinical, functional, and psychosocial consequences that extend beyond skin involvement.[1], [2] Global prevalence is commonly estimated at approximately 2% to 3%, with variation across regions, age groups, and data sources.[3], [4] A systematic review of studies from Latin America and the Caribbean found no population-based studies and showed that most available estimates were derived from hospital registries or clinical databases, limiting reliable cross-country comparisons.^5^ Colombian registry and multicenter data provide country-specific information, but comparable population-level estimates are still sparse for many countries in the region.[6], [7]

Psoriasis is associated with psoriatic arthritis, cardiometabolic disease, and impaired health-related quality of life, which together increase the clinical and economic burden of care.[1], [4], [8] Multi-country analyses from Argentina, Brazil, Colombia, and Mexico support that psoriasis generates a relevant humanistic and economic burden in Latin America, including health care use, productivity loss, and out-of-pocket costs.^9^ Studies from Brazil and regional surveys further show unmet therapeutic needs and access gaps that affect timely treatment initiation and continuity of care.[10], [11], [12]

Advances in biologic therapies and oral small molecules have changed the management of moderate-to-severe psoriasis. In Latin America, access to these treatments remains uneven across health systems, as shown by studies describing gaps in treatment availability, reimbursement, and unmet therapeutic needs in Brazil, Chile, and other countries in the region.[11], [12] Some public systems reimburse selected advanced therapies, whereas other settings require private coverage, out-of-pocket payment, administrative authorization, or legal coverage pathways for medicines that are not routinely financed. Because availability and affordability vary by country, conventional systemic agents such as methotrexate, acitretin, and cyclosporine, as well as phototherapy when available, remain clinically relevant within regional treatment algorithms.

Although several countries in the region have updated or published national psoriasis guidance ‒ including the 2024 Brazilian Consensus on Psoriasis and Treatment Algorithm of the Sociedade Brasileira de Dermatologia ‒ harmonized regional standards remain needed.^13^ Latin American health systems differ markedly in regulatory approval processes, reimbursement policies, biologic access, and availability of phototherapy and conventional systemic agents, which underscores the need for regionally adapted guidance.

## Methods

This clinical practice guideline was developed in accordance with international standards for guideline development from the Guidelines International Network (GIN) and by following the GRADE (Grading of Recommendations Assessment, Development and Evaluation) methodology.[14], [15] The process was structured to support transparency and applicability of recommendations across Latin American countries by incorporating acceptability and feasibility through the Evidence to Decision (EtD) framework for decision-making.

### Participants

The guideline development group consisted of 13 dermatology specialists with expertise in psoriasis, representing different countries in the region. Methodological coordination was led by EpiThink Health Consulting, an independent consulting group with experience in guideline development. Patient representatives from Brazil, Mexico, Argentina, Guatemala, Colombia, and Chile contributed their perspectives on the disease, on the topics addressed in the guideline, and on the acceptability of the proposed recommendations. Their input informed the Evidence-to-Decision deliberations, particularly with respect to patient values and treatment preferences. All participants declared potential conflicts of interest, which were reviewed in accordance with international standards.^16^ Details on guideline participants are available in Supplementary material 1.

### Formulation of clinical questions

Clinical questions included in the guideline were defined by consensus among clinical experts, prioritizing therapeutic targets; conventional systemic and biologic treatment, including biosimilars; scenarios of treatment failure; management of psoriasis in high-impact areas and other clinical forms; management in special situations; and, finally, the use of nonpharmacologic therapies. Clinical questions were structured using the PICO format (population, intervention, comparator, and outcome).^17^

### Evidence search and appraisal

Systematic reviews were conducted according to the Cochrane manual,^18^ with searches in MEDLINE, Embase, and the Cochrane Library through May 2025, and updated to January 2026. Structured searches were complemented by free-text searches in Google Scholar. Publications in English and Spanish were considered. Two reviewers independently screened retrieved references and selected those meeting eligibility criteria. Disagreements were resolved by consensus or by a third reviewer.

The process followed PRISMA (Preferred Reporting Items for Systematic Reviews and Meta-Analyses) guidance (20). Methodological quality was assessed using validated tools: AGREE II for clinical guidelines.^19^ AMSTAR for systematic reviews^20^ the Cochrane tool for clinical trials^21^ and JBI instruments for other study designs.^22^ Recent studies with higher methodological quality were prioritized. Details of evidence selection and appraisal are presented in Supplementary material 2. Certainty of evidence was rated with GRADE as high, moderate, low, or very low based on risk of bias, precision, consistency, directness, and potential publication bias.^23^

### Formulation of recommendations

Information extracted from the search was synthesized into evidence summaries and GRADE tables for each available intervention and comparison. The panel discussed the evidence in virtual sessions using the GRADE Evidence to Decision framework,^24^ considering benefits, harms, certainty of evidence, patient values and preferences, acceptability, impact on health resource use, and feasibility. The EtD analysis is presented in Supplementary material 3.

Once recommendations were drafted, they were graded for strength and direction. Recommendations can be strong or conditional, depending on the certainty of evidence and the expected magnitude of effect. In some exceptional situations, a strong recommendation may be based on low-quality evidence, particularly when the intervention is safe, low cost, and omission could lead to serious consequences, or when there is certainty about harm from an intervention with uncertain benefit. Interpretation of recommendation grading is presented in Table 1.

**Table 1:** Strength and direction of recommendations: clinical implications.

Classification	Definition	Implication
Strongly for	Benefits clearly outweigh harms.	Most patients should receive the intervention.
Strongly against	Harms clearly outweigh benefits.	Most patients should not receive the intervention.
Conditionally for	Benefits probably outweigh harms, but uncertainty remains.	Individualized decision based on context and preferences.
Conditionally against	Harms probably outweigh benefits, but uncertainty remains.	Do not use routinely; consider only in specific contexts.

In addition, good practice statements were developed for situations where a favorable balance was clear and clinical consensus was explicit.[25], [26] In settings where evidence was limited or absent, expert consensus was used.

**Table:** 

**Recommendations**
**General principles for psoriasis management**
1 Apply a patient-centered approach in psoriasis care that incorporates clinical features, comorbidities, sociocultural context, and psychosocial impact through shared, informed decision-making. 2 Prioritize equity and continuity of access to therapies based on health system capacity and local availability. 3 Select therapies using a comprehensive assessment of skin involvement and quality-of-life impact, integrating coordinated interdisciplinary management of metabolic, cardiovascular, musculoskeletal, and psychiatric comorbidities. 4 Use a stepwise treatment approach, choosing interventions based on severity, response, safety, and cost-effectiveness, and adjusting therapy using periodic assessments of disease control. 5 Maintain safety as a cross-cutting component of care through routine monitoring and adverse event reporting within active pharmacovigilance programs. 6 Avoid drugs with absolute contraindications when treating special populations (pregnant patients, people who are breastfeeding, children, older adults, and patients with relevant comorbidities) to reduce adverse event risk. 7 Provide patient education to support adherence and self-care, including strategies to address triggers and support healthy habits. 8 Adapt therapeutic decisions to the regional socioeconomic and health system context to support rational resource use and contextual adaptation of international recommendations.

**Fig. 1:**
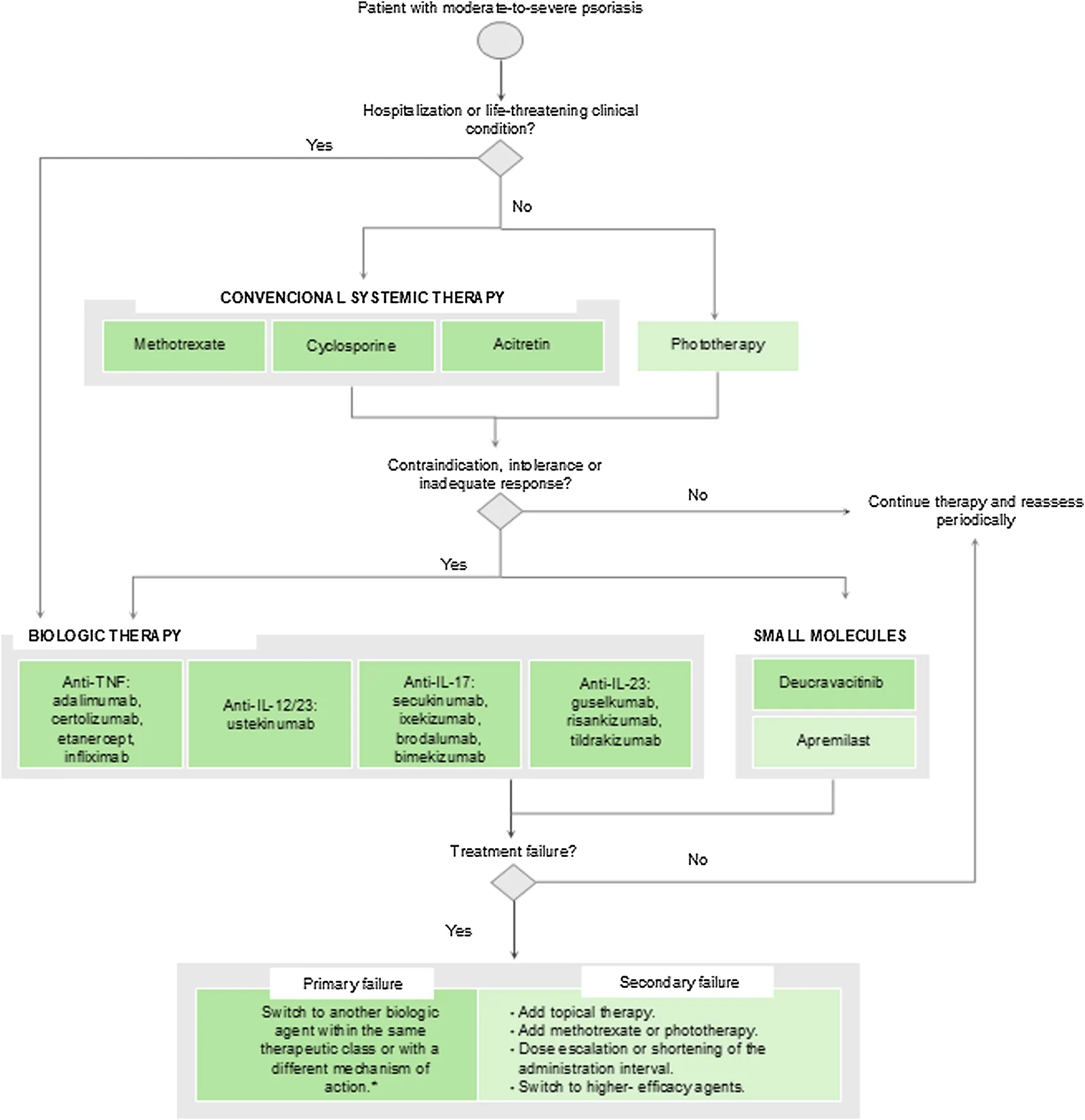
Psoriasis treatment algorithm. IL, Interleukin; TNF, Tumor Necrosis Factor. Notes: Green intensity denotes strong recommendations (darker green) or conditional recommendations (lighter green). * In cases of primary failure to an anti-TNF agent, switching to another drug within the same therapeutic class is not recommended.

Fig. 1 describes the overall psoriasis treatment algorithm based on the evidence and expert panel deliberations regarding currently available interventions.

### Therapeutic goals

**Table:** 

**Recommendation 1**
In patients with plaque psoriasis, we recommend establishing the following treatment targets: Absolute PASI < 3, or PASI90 response, or PGA 0/1, and DLQI < 5*
*If DLQI measurement is not available, consider objective measures only.
Strong recommendation for, moderate-certainty evidence
**Recommendation 2**
In patients with nail psoriasis, we suggest establishing modified NAPSI < 10 as the treatment target.
Conditional recommendation for, low-certainty evidence
**Recommendation 3**
In patients with generalized pustular psoriasis, we suggest establishing GPPASI < 3 as the treatment target.
Conditional recommendation for, very low-certainty evidence
**Recommendation 4**
In patients with other forms of psoriasis (guttate, erythrodermic, inverse, palmoplantar, scalp), we suggest establishing PGA 0/1 as the treatment target.
Conditional recommendation for, very low-certainty evidence

DLQI, Dermatology Life Quality Index; GPPASI, Generalized Pustular Psoriasis Area and Severity Index; NAPSI, Nail Psoriasis Severity Index; PASI, Psoriasis Area and Severity Index; PGA, Physician’s Global Assessment.

### Evidence synthesis

The available evidence on therapeutic targets in psoriasis comes primarily from post hoc analyses and observational studies and is considered low to moderate quality. There is growing consensus that achieving a 90% or greater response on the Psoriasis Area and Severity Index (PASI) should be the optimal treatment target in patients with moderate-to-severe psoriasis. This is supported by recent studies of biologic agents such as bimekizumab, brodalumab, guselkumab, ixekizumab, risankizumab, and secukinumab, which show a higher likelihood of complete or near-complete clearance of cutaneous lesions with a favorable impact on health-related quality of life. In addition, patients who achieve PASI90 or PASI100 report greater treatment satisfaction, less interference with daily activities, and meaningful improvement in symptoms such as pruritus and pain.[27], [28], [29], [30], [31], [32], [33], [34], [35], [36], [37], [38], [39], [40], [41], [42], [43], [44], [45]

A strong correlation has also been demonstrated between absolute PASI < 3 and a PASI90 response, supporting its use as an alternative marker of optimal disease control. Complementary evidence from studies evaluating Body Surface Area (BSA) suggests that maintaining BSA ≤ 1% or 3% is associated with a quality of life comparable to that of the general population and could be considered a reasonable clinical target.

### From evidence to decision

The panel recognized that complete clearance is an ideal goal but is difficult to achieve in routine practice; therapeutic targets such as absolute PASI < 3, PASI90, or Physician’s Global Assessment (PGA) are realistic and reflect adequate disease control. The Dermatology Life Quality Index (DLQI) is central to assessment, recognizing that a subset of patients may show results that are discordant with clinical findings or not evaluable. BSA ≤ 1%, despite more limited use and evidence, can be considered an alternative when PASI or PGA are not available.

The modified Nail Psoriasis Severity Index (modified NAPSI), the Generalized Pustular Psoriasis Area and Severity Index (GPPASI), and PGA were considered appropriate tools to evaluate therapeutic success in nail psoriasis, generalized pustular psoriasis, and other psoriasis subtypes, respectively.

### Conventional nonbiologic treatment

**Table:** 

**Recommendation 5**
In patients with moderate-to-severe plaque psoriasis, we recommend methotrexate as first-line systemic therapy, generally starting at 7.5–15 mg/week and titrating according to clinical response and tolerability, with usual target doses of 15–20 mg/week.
*Strong recommendation for, moderate-certainty evidence*
**Recommendation 6**
In patients with moderate-to-severe plaque psoriasis, we recommend cyclosporine (3–5 mg/kg/day) as a first-line systemic therapy option.
*Observation: Use cyclosporine for the shortest duration possible and discontinue once the treatment target is achieved. Ideally, continuous treatment should not exceed 1 year.*
*Strong recommendation for, moderate-certainty evidence*
**Recommendation 7**
In patients with moderate-to-severe plaque psoriasis, we recommend acitretin (0.3–0.7 mg/kg/day) as a first-line systemic therapy option.
*Observation: Acitretin is contraindicated during pregnancy and should be avoided in patients planning pregnancy. In women of reproductive potential, effective contraception must be ensured during treatment and for up to 3 years after discontinuation because of prolonged teratogenic risk.*
*Strong recommendation for, moderate-certainty evidence*
**Recommendation 8**
In patients with moderate-to-severe plaque psoriasis, we suggest phototherapy (NB-UVB or PUVA) as a first-line treatment option.
*Conditional recommendation for, low-certainty evidence*

NB-UVB, Narrowband Ultraviolet B; PUVA, Psoralen Plus Ultraviolet A.

### Evidence synthesis

Evidence on methotrexate use in moderate-to-severe psoriasis is of moderate quality, with PGA 0–1 and PASI75 response rates up to 74%.^46^ Hepatotoxicity has been described in some cases of prolonged use; no established threshold currently exists for treatment duration. Typical starting doses range from 7.5–15 mg/week, with titration according to clinical response and tolerability; usual target doses are 15–20 mg/week.^47^ Cyclosporine has shown superiority over placebo, with PASI75 response rates between 10% and 40%, greater efficacy at 5 mg/kg/day, and results comparable to methotrexate with lower discontinuation rates.^48^

Acitretin has moderate-quality evidence, with PASI75 response rates between 47% and 69% at 12 weeks, particularly at 25 mg/day. At higher doses (50 mg/day), an increase in minor adverse events was reported, including scaling, pruritus, alopecia, rhinitis, spasms, and erythema.^46^ Combined use of acitretin and methotrexate, a practice that is uncommon, has shown higher efficacy and lower hepatotoxicity (except in alcohol users), with more favorable cutaneous effects and no increase in hepatic fibrosis.^49^

Phototherapy remains an effective alternative, with no meaningful difference in PASI75 between NB-UVB and PUVA (minimum 20–30 sessions, depending on joules), although clearance and recurrence rates are variable.[46], [50] Combining phototherapy with methotrexate or acitretin improves clinical response, accelerates clearance, and allows lower cumulative dosing and reduced hepatotoxicity. Clinical studies report PASI75 rates of 80%–90% with methotrexate plus NB-UVB combinations, and greater efficacy of acitretin plus phototherapy compared with monotherapy.^51^

### From evidence to decision

The expert panel agreed that methotrexate remains a first-line treatment option for plaque psoriasis in Latin America due to its efficacy, safety, and low cost. Although comparative evidence between oral and subcutaneous routes is limited, clinical experience suggests a faster therapeutic response with subcutaneous administration, highlighting the need for further studies. Subcutaneous administration may therefore be considered for patients requiring dose escalation within accepted dosing ranges or in those experiencing nausea or intolerance to oral therapy. Cyclosporine is primarily recommended for acute flares or severe disease, with its use limited to a maximum of 12-months because of the risk of nephrotoxicity. Selection of conventional systemic therapy should be based on disease severity, contraindications, and intolerance to methotrexate.

As systemic alternatives, acitretin has been effective, although access is restricted in some countries. In women of reproductive potential, effective contraception must be ensured during acitretin therapy and for up to 3-years afterward because of teratogenic risk. Phototherapy use depends on local equipment availability, limiting application in remote areas. Methotrexate plus NB-UVB, or acitretin plus PUVA, are useful options in selected cases, especially when other alternatives are not available.

### Biologic therapy and small molecules

**Table:** 

**Recommendation 9**
In patients with moderate-to-severe plaque psoriasis, we recommend biologic agents or small molecules when there is a contraindication, intolerance, or lack of response to at least one conventional systemic drug or phototherapy, used at adequate doses for a minimum of 12–16 weeks.
*Strong recommendation for, high-certainty evidence*
**Recommendation 10**
In patients with severe plaque psoriasis who require hospitalization or whose clinical condition is life-threatening, we recommend biologic agents as the first therapeutic option, given the need for a rapid disease response.
*Strong recommendation for, moderate-certainty evidence*
**Recommendation 11**
In patients with moderate-to-severe plaque psoriasis, we recommend the use of any of the following biologic therapies, with no preferred order: anti-TNF agents (adalimumab, certolizumab), anti-IL-12/23 (ustekinumab), anti-IL-17 (bimekizumab, brodalumab, ixekizumab, secukinumab), or anti-IL-23 (guselkumab, risankizumab, tildrakizumab).
*Strong recommendation for, high-certainty evidence*
**Good Practice Statement 1**
In patients with psoriasis, including high-impact areas and different clinical forms, treatment selection should be based on an individualized assessment that considers the patient’s clinical and comorbidity profile, clinical judgment, costs, and drug availability in each country. There is no pre-established stepwise escalation scheme within this therapeutic group.
**Recommendation 12**
In patients with moderate-to-severe plaque psoriasis, we recommend deucravacitinib as a therapeutic option.
*Strong recommendation for, moderate-certainty evidence*
**Recommendation 13**
In patients with moderate-to-severe plaque psoriasis, we suggest apremilast as a nonpreferred therapeutic option when biologic therapies or higher-efficacy oral options are contraindicated, unavailable, or not feasible.
*Conditional recommendation for, low-certainty evidence*
**Recommendation 14**
In patients with moderate-to-severe plaque psoriasis who are candidates for biologic therapy, we suggest considering approved anti-TNF and anti-IL-12/23 biosimilars, applying the same indications and guidance as the innovator products, according to local regulatory approval, interchangeability rules, and pharmacovigilance requirements.
*Conditional recommendation for, low-certainty evidence*

IL, Interleukin; TNF, Tumor Necrosis Factor.

### Evidence synthesis

Evidence on biologic agents in moderate-to-severe psoriasis shows that IL-17, IL-23, IL-12/23, and anti-TNF inhibitors are superior to placebo, with higher PASI75 and PASI90 response rates and improved quality of life, without an increase in serious adverse events at 24 weeks.^52^ Studies with follow-up to 56 weeks report variable but sustained efficacy, adherence, and persistence by agent, while therapeutic survival beyond 5 years has been less well documented.[53], [54], [55]

For small molecules, a recent systematic review found that deucravacitinib (6 mg/day) improved PASI90, DLQI, and PGA.^56^ Its safety profile was favorable, with predominantly mild adverse events (nausea, headache, infections) and no increase in serious adverse events or discontinuations. Apremilast has shown clinical efficacy for PASI75 and PPPGA 0/1 compared with placebo, without an increase in serious adverse events.^57^ Moderate-quality evidence supports small molecules as effective and safe alternatives in patients with access limitations or contraindications to biologic therapy.

In the setting of biologic need, evidence on biosimilars in psoriasis has increased steadily. Anti-TNF and ustekinumab biosimilars have shown clinical equivalence to reference products for efficacy (PASI, DLQI) and safety, with moderate certainty. Some studies report no meaningful differences between adalimumab biosimilars and the innovator for PASI 50/75/90/100 responses or adverse events at week 51, supporting equivalence when initiating or switching therapy.[58], [59] For infliximab, a 6 month follow-up study supports clinical interchangeability.^60^ Although infliximab was not included among the preferred biologic options for routine plaque psoriasis management in this guideline, this evidence was retained because infliximab may still be used in selected settings or specific clinical scenarios where other recommended biologics are unavailable. Overall, the evidence supports considering anti-TNF and IL-12/23 inhibitor biosimilars for the same indications and guidance as their reference biologics, subject to local regulation and pharmacovigilance requirements.

### From evidence to decision

The panel considered biologics a key option for moderate-to-severe plaque psoriasis, particularly in patients with contraindication, intolerance, or failure of conventional therapies or phototherapy. Use is also justified in severe disease or life-threatening presentations. The risk-benefit balance should be assessed carefully before initiating biologic therapy, particularly regarding latent tuberculosis infection. All candidates for biologic treatment should undergo screening for latent tuberculosis using tuberculin skin testing and/or an interferon-gamma release assay, according to local guidance, before therapy is started. Reactivation risk is highest with anti-TNF agents; in patients with confirmed latent tuberculosis, preventive treatment should be initiated according to national protocols and should begin at least 1-month before anti-TNF therapy or cyclosporine. For IL-17 and IL-23 inhibitors, current observational and meta-analytic evidence suggests a very low reactivation risk, supporting concurrent initiation of latent tuberculosis treatment and psoriasis therapy when clinically appropriate.[61], [62] Based on their mechanisms of action and available safety data, the same approach is considered applicable to IL-12/23 inhibitors and deucravacitinib, as specified in the good practice statements for the latent tuberculosis section. In patients with active tuberculosis, biologic therapy should be deferred until at least the initial phase of antituberculous treatment has been completed.

Specifically recommended biologics include adalimumab, bimekizumab, brodalumab, certolizumab, guselkumab, ixekizumab, risankizumab, secukinumab, tildrakizumab, and ustekinumab. Selection should be individualized based on clinical profile, availability, and clinician judgment, without a pre-established escalation pathway. In some countries where recommended biologics are not available, etanercept and infliximab may be options, although closing access gaps to higher-efficacy and safer treatments remains a goal. High biologic costs may be offset by preventing comorbidities and long-term complications such as psoriatic arthritis and may also be reduced through biosimilar use.

In Latin America, use of biosimilars (adalimumab, etanercept, infliximab, ustekinumab) varies based on national regulations regarding interchangeability with reference biologics. Overall, biosimilars have shown favorable outcomes and improved affordability, while ongoing active pharmacovigilance is needed to ensure sustained effectiveness and safety.

For small molecules, apremilast use has declined because of lower efficacy, while deucravacitinib shows effectiveness comparable to anti-TNF agents and safety similar to IL-23 inhibitors, emerging as a therapeutic alternative.

### Therapeutic failure

**Table:** 

**Recommendation 15**
In patients with moderate-to-severe plaque psoriasis who have failed anti-TNF therapy, we recommend switching to a biologic agent with a different mechanism of action (anti-IL-17, anti-IL-23, or anti-IL-12/23).
*Strong recommendation for, moderate-certainty evidence*
**Recommendation 16**
In patients with moderate-to-severe plaque psoriasis who have failed anti-IL-17, anti-IL-23, or anti-IL-12/23 therapy, we suggest switching to another biologic agent within the same class or with a different mechanism of action.
*Conditional recommendation for, moderate-certainty evidence*
**Recommendation 17**
In patients with moderate-to-severe plaque psoriasis who have failed after a partial response to biologic therapy (PASI 3–10, DLQI > 5, or high-impact area involvement), we suggest the following options based on availability, risk-benefit assessment, and costs: Add topical therapy; Add systemic therapy (methotrexate or phototherapy); Consider a dose increase or shortening the dosing interval; Consider switching to higher-efficacy molecules while maintaining rational resource use.
*Conditional recommendation for, moderate-certainty evidence*

IL, Interleukin; TNF, Tumor Necrosis Factor.

### Evidence synthesis

Evidence on management of therapeutic failure in psoriasis is heterogeneous. In patients treated with anti-TNF agents, switching to a biologic with a different mechanism of action is generally preferred because IL-17 and IL-23 inhibitors have shown higher efficacy in controlled and observational studies. Switching from an IL-12/23 inhibitor to guselkumab because of inadequate response showed clinical benefit in the randomized, double-blind, phase III NAVIGATE trial, which included 871 patients with inadequate response to ustekinumab.^63^ Switching within a biologic class can be effective in secondary loss of response, particularly for IL-17 and IL-23 inhibitors.

Treatment sequencing should reflect the type of failure: switching to a different class is favored when there is no meaningful initial response, whereas switching within a class can be considered after secondary loss of response to preserve future therapeutic options. Selection should also account for psoriatic arthritis involvement. Because psoriatic arthritis was not formulated as a separate clinical question in this guideline, this statement should be interpreted as a contextual consideration rather than a formal recommendation. When axial disease is present, IL-17 inhibitors may be favored; for peripheral disease, either IL-17 or IL-23 inhibitors may be considered, according to the clinical profile, availability, and local regulatory approval.^35^

### From evidence to decision

The guideline group considered that several literature-supported strategies can be used after therapeutic failure, with careful risk–benefit assessment. There is no single ideal escalation scheme for biologic use; class switching is favored for primary failure, while options for secondary failure can be selected based on therapeutic convenience. Adding topical or systemic therapy, dose adjustment, or switching agents are options that can be considered based on individual patient needs.

### High-impact areas and special forms

**Table:** 

**Recommendation 18**
In patients with scalp psoriasis, we recommend any of the following options without a preferred order: conventional systemic therapies, biologics (adalimumab, etanercept, guselkumab, infliximab, ixekizumab, risankizumab, secukinumab, ustekinumab), or small molecules (apremilast or deucravacitinib), alone or combined with topical treatments.
*Strong recommendation for, moderate-certainty evidence*
**Recommendation 19**
In patients with nail psoriasis, we recommend any of the following options without a preferred order: adalimumab, etanercept, guselkumab, infliximab, ixekizumab, risankizumab, secukinumab, or ustekinumab.
*Strong recommendation for, moderate-certainty evidence*
**Recommendation 20**
In patients with palmoplantar psoriasis, we recommend any biologic therapy without a preferred order, alone or combined with topical treatments.
*Strong recommendation for, moderate-certainty evidence*
**Recommendation 21**
In patients with inverse psoriasis, we suggest biologic therapies or small molecules without a preferred order.
*Conditional recommendation for, low-certainty evidence*
**Recommendation 22**
In patients with genital psoriasis, we suggest any biologic therapy without a preferred order.
*Conditional recommendation for, low-certainty evidence*
**Recommendation 23**
In patients with erythrodermic psoriasis, we recommend any of the following high-efficacy biologics without a preferred order: bimekizumab, guselkumab, infliximab, ixekizumab, risankizumab, or secukinumab.
*Strong recommendation for, moderate-certainty evidence*
**Recommendation 24**
In patients with guttate psoriasis, we suggest any biologic therapy without a preferred order.
*Conditional recommendation for, low-certainty evidence*
**Recommendation 25**
In patients with generalized pustular psoriasis, we recommend spesolimab as the first treatment option.
*Strong recommendation for, high-certainty evidence*
**Recommendation 26**
In patients with generalized pustular psoriasis, if spesolimab is contraindicated or not available, we suggest any of the following therapies without a preferred order: bimekizumab, cyclosporine, guselkumab, infliximab, ixekizumab, risankizumab, or secukinumab.
*Observation: Acitretin and methotrexate remain contextual options for selected patients with generalized pustular psoriasis when spesolimab and biologic therapy are unavailable, contraindicated, or not feasible. Their use should be individualized because methotrexate has a slower onset of action and requires laboratory monitoring, whereas acitretin carries prolonged teratogenic risk after discontinuation. These agents should not be interpreted as equivalent substitutes for rapid-acting targeted therapy during severe generalized pustular psoriasis flares when such treatment is available.*
*Conditional recommendation for, low-certainty evidence*

IL, Interleukin; TNF, Tumor Necrosis Factor.

### Evidence synthesis

In scalp psoriasis, multiple systemic agents have demonstrated efficacy, with heterogeneity across study designs and outcome definitions. Biologics, particularly IL-17 and IL-23 inhibitors, have shown favorable clinical and quality-of-life outcomes in patients with scalp involvement.[64], [65] Among oral small molecules, deucravacitinib has demonstrated efficacy and tolerability in moderate-to-severe scalp psoriasis in a phase 3b/4 randomized trial, and the broader evidence for TYK2 and PDE-4 inhibitors in scalp disease has been summarized in recent systematic reviews.[66], [67] No direct comparative trials establish a single preferred systemic agent for scalp psoriasis; treatment choice should therefore be individualized according to severity, comorbidities, patient preference, and local access.

In nail psoriasis, targeted systemic therapies, including anti-TNF, IL-17, IL-23, and IL-12/23 inhibitors, have shown meaningful improvement across nail-specific outcomes, although comparative certainty remains limited by indirect comparisons and heterogeneous endpoints.[68], [69] Reported adverse events are generally consistent with the known safety profiles of each drug class.

In genital and inverse psoriasis, available evidence is limited and mostly derived from small studies, trials in selected high-impact areas, and systematic reviews. Topical corticosteroids, calcineurin inhibitors, and vitamin D analogs can be effective, although tolerability may be limited by local irritation in sensitive skin areas. Biologic therapy is appropriate for severe or recalcitrant disease, with ixekizumab and selected IL-23 inhibitors supported by clinical studies in genital psoriasis or high-impact areas.[70], [71]

In palmoplantar psoriasis and palmoplantar pustulosis, biologic agents have shown efficacy across outcomes such as Palmoplantar Physician Global Assessment and Palmoplantar Psoriasis Area and Severity Index responses, with no single class clearly preferred based on the available comparative evidence.[72], [73], [74] Evidence for small molecules remains more limited, and the role of Janus kinase inhibitors is still uncertain.

In generalized pustular psoriasis, available evidence remains limited and varies across drug classes. Spesolimab has randomized trial evidence supporting rapid disease control and is preferred when available and appropriate. IL-17, IL-23, and anti-TNF inhibitors have also shown efficacy in observational studies and smaller clinical series. Conventional systemic agents, including acitretin, cyclosporine, and methotrexate, have historically been used in generalized pustular psoriasis, particularly in settings where access to targeted therapies is restricted. Real-world data show that acitretin and methotrexate continue to be used during generalized pustular psoriasis flares, although the evidence base is less direct than for newer targeted agents and treatment choice must account for onset of action, reproductive safety, comorbidities, and monitoring requirements.^75^

In erythrodermic psoriasis, systemic therapies ‒ both biologic and oral ‒ can produce rapid improvement and high PASI 90/100 response rates.[76], [77] IL-17 inhibitors stand out for their rapid onset of action, even in cases with uncertain etiology. Clinical benefit has also been reported with other biologic classes, including anti-TNF and anti-IL-23 agents;^78^ however, the lack of comparative trials and the predominance of case series preclude the designation of a single first-line therapy.

In guttate psoriasis, topical therapies (corticosteroids, calcipotriol) and phototherapy with NB-UVB or PUVA are highly effective and safe. Favorable responses have been reported with IL-17, IL-23, and IL-12/23 inhibitors, although evidence comes from observational studies and case reports.^79^

### From evidence to decision

Experts recognize that evidence for managing psoriasis in high-impact areas remains limited. In all settings, selection should be individualized based on the needed speed of response, comorbidities, and therapy availability. Clinical trials in genital psoriasis have demonstrated meaningful benefits with ixekizumab and guselkumab. Agents supported by these data are preferred; still, given variability in biologic availability across the region, other agents such as adalimumab, ustekinumab, risankizumab and secukinumab have also been used in practice with favorable outcomes.

For erythrodermic psoriasis and generalized pustular psoriasis, evidence and experience with systemic and biologic therapy support multiple options, prioritizing treatments with stronger documented efficacy: IL-17 inhibitors, IL-23 inhibitors, and anti-TNF agents. If preferred therapies are not available (see specific recommendations), use of adalimumab, certolizumab, etanercept, or ustekinumab is suggested.

### Special situations

#### Psoriasis and pregnancy

**Table:** 

**Recommendation 27**
In pregnant patients with mild-to-moderate psoriasis, we recommend topical therapy (emollients, mid-potency topical corticosteroids, or calcipotriol) as first-line treatment.
*Strong recommendation for, very low-certainty evidence*
**Recommendation 28**
In pregnant patients with moderate-to-severe psoriasis, we recommend UVB phototherapy as systemic treatment.
*Strong recommendation for, very low-certainty evidence*
**Recommendation 29**
In pregnant patients with moderate-to-severe psoriasis, we suggest cyclosporine as second-line systemic management.
*Conditional recommendation for, very low-certainty evidence*
**Recommendation 30**
In pregnant patients with psoriasis who require biologic therapy, we suggest certolizumab pegol as the first option.
*Conditional recommendation for, very low-certainty evidence*
**Recommendation 31**
In pregnant patients for whom certolizumab pegol is not available, is contraindicated, or is ineffective, we suggest considering secukinumab, ixekizumab, ustekinumab, risankizumab, guselkumab, tildrakizumab, brodalumab, or bimekizumab in exceptional circumstances and after an individualized risk-benefit assessment.
*Conditional recommendation for, very low-certainty evidence*
**Good practice statement 2**
In women with psoriasis who are planning pregnancy, are pregnant, or are postpartum, comprehensive risk management should be performed, including counseling on safe contraception, available therapeutic options, and potential treatment risks.
**Good practice statement 3**
The decision to start or continue biologic therapy during pregnancy or the postpartum period should be based on an individualized assessment performed jointly by dermatology, obstetrics, and, when needed, maternal–fetal medicine.

UVB, Ultraviolet B.

### Evidence synthesis

In psoriasis management during pregnancy, topical therapies and UVB phototherapy are considered safe options, especially in the first trimester. Cyclosporine can be used as a second-line alternative because of low placental transfer.^80^ In contrast, acitretin and methotrexate are contraindicated during pregnancy and should be avoided in patients planning pregnancy. For biologic therapies, available analyses have not identified meaningful differences in obstetric outcomes among agents; however, the certainty of evidence remains low due to heterogeneity and small sample sizes, underscoring the need for individualized risk–benefit assessment.[81], [82]

#### From evidence to decision

In women with psoriasis who are planning pregnancy, are pregnant, or are postpartum, we recommend offering counseling on safe contraception, available treatment options, potential risks, and individualized planning for discontinuation or reintroduction of immunomodulatory drugs. Use of topical corticosteroids in the third trimester should be evaluated individually based on the risk-benefit balance. Decisions to start or continue biologic therapy and, when required, to switch to certolizumab-based management should be grounded in a comprehensive risk-benefit assessment with dermatology and obstetrics/gynecology involvement.

#### Psoriasis and cancer

**Table:** 

**Good practice statement 4**
Before starting biologic therapy and during its use, evaluate personal cancer history and oncologic risk factors. In patients with a recent history of cancer, the decision to use biologic therapy should be based on an individualized risk–benefit assessment in coordination with oncology.
**Recommendation 32**
In patients with active malignancies, we suggest topical therapies, phototherapy (except in those at high risk for skin cancer), and acitretin.
*Conditional recommendation for, low-certainty evidence*
**Recommendation 33**
In patients with a history of cancer, we suggest considering apremilast, deucravacitinib, IL-17 inhibitors, IL-23 inhibitors, or IL-12/23 inhibitors. In patients receiving anti-TNF therapy, evaluate switching to one of these options.
*Conditional recommendation for, low-certainty evidence*

IL, Interleukin; TNF, Tumor Necrosis Factor.

### Evidence synthesis

IL-17 and IL-23 inhibitors have shown favorable efficacy and safety profiles in patients with prior cancer or concomitant cancer in real-world studies.^83^ Recurrence rates or new tumor rates are reported as similar between patients not receiving immunosuppressants and those treated with anti-TNF agents, immunomodulators, or combinations, although the available evidence is low quality.^84^ JAK inhibitors have been associated with a higher incidence of malignancies, although oncologic events are uncommon across all groups.^85^

### From evidence to decision

In patients with a history of cancer, treatment choice should incorporate cancer history, malignancy status (active, remission, or palliative care), and individual risk factors. In recent malignancy, joint assessment with oncology is recommended. Although available biologics generally have favorable safety profiles, avoiding anti-TNF agents in this subgroup and considering alternatives with lower immunosuppressive potential is suggested.

#### Psoriasis and chronic infections (Hepatitis, HIV)

**Table:** 

**Good practice statement 5**
Management of patients with psoriasis and comorbid chronic hepatitis or HIV infection should be interdisciplinary, led by dermatology with support from hepatology, gastroenterology, and infectious diseases as appropriate.
**Good practice statement 6**
In patients with chronic viral infections (hepatitis B, hepatitis C, HIV), ensure adequate treatment of the underlying infection, aiming for an undetectable viral load to reduce reactivation risk and related complications.
**Good practice statement 7**
In patients with psoriasis who are candidates for systemic therapy, implement comprehensive risk management through hepatitis B and C screening, hepatitis B vaccination in nonexposed or unvaccinated individuals when indicated, antiviral prophylaxis in hepatitis B virus carriers who will receive high-risk immunosuppressants, and referral for confirmatory testing and antiviral management in patients with hepatitis C infection.
**Recommendation 34**
In patients with psoriasis and hepatitis C infection, we recommend UVB phototherapy as a therapeutic option.
*Strong recommendation for, low-certainty evidence*
**Recommendation 35**
In patients with active hepatitis B, we suggest not initiating immunosuppressive systemic therapy until the viral infection has been treated.
*Conditional recommendation against, low-certainty evidence*
**Recommendation 36**
In patients with psoriasis and HIV infection receiving antiretroviral therapy, we recommend phototherapy, preferably NB-UVB, as first-line treatment.
*Strong recommendation for, very low-certainty evidence*
**Recommendation 37**
In patients with psoriasis and HIV infection, we suggest acitretin as second-line therapy.
*Conditional recommendation for, very low-certainty evidence*
**Recommendation 38**
In patients with psoriasis and HIV infection who do not respond to conventional therapy, we suggest considering biologic therapy with IL-23, IL-12/23, or IL-17 inhibitors.
*Conditional recommendation for, very low-certainty evidence*

IL, Interleukin; UVB, Ultraviolet B; HIV, Human Immunodeficiency Virus.

### Evidence synthesis

Evidence on psoriasis treatment in patients with hepatitis is low quality, limited by small case numbers and retrospective designs. Hepatitis B reactivation is a clinically meaningful risk in patients treated with biologics, particularly anti-TNF agents, while IL-17 and IL-23 inhibitors have a more favorable safety profile in this context.^86^ Patients who are Hepatitis B surface Antigen (HBsAg) positive have a high reactivation risk, supporting preventive antiviral prophylaxis. In HBsAg-negative/anti-HBc-positive individuals, the risk of reactivation is lower, although potential outcomes may be more severe, supporting the need for close monitoring. Although serious events are uncommon, this evidence supports universal hepatitis B and C screening before biologic initiation.[87], [88]

Evidence on psoriasis management in people with HIV is limited. Phototherapy or acitretin is preferred given their more favorable safety profile, whereas methotrexate and cyclosporine carry relative contraindications due to the potential to worsen immunosuppression.

In refractory cases, IL-23, IL-12/23, or IL-17 inhibitors have demonstrated efficacy and safety, with no evidence of viral reactivation or serious adverse events;^89^ and favorable outcomes with risankizumab have been reported with follow-up of up to two years.^90^ Overall, biologic therapy has not been associated with an increased risk of infectious complications, and viral reactivation associated with biologic use appears to be low (0.12; 95% CI 0.00–0.40), with few severe cases reported.[87], [89] Regular CD4+ monitoring and coordinated infectious disease care are recommended to support treatment safety.

### From evidence to decision

In candidates for systemic immunosuppressive or biologic therapy, hepatitis B and C screening should be performed before treatment initiation. Hepatitis B vaccination should be offered to nonexposed or unvaccinated individuals when indicated. For hepatitis C, patients with positive screening should be referred for confirmatory testing, antiviral management, and coordinated follow-up. Interdisciplinary management among dermatology, gastroenterology, hepatology, and infectious diseases is central in patients with chronic hepatitis or HIV infection to optimize safety and treatment efficacy.

#### Psoriasis and latent tuberculosis

**Table:** 

**Good practice statement 8**
Evaluation for latent tuberculosis in candidates for methotrexate should be individualized based on risk profile.
**Good practice statement 9**
Treatment for latent tuberculosis should be started at least 1 month before initiating anti-TNF therapy or cyclosporine.
**Good practice statement 10**
In patients with latent tuberculosis who are candidates for IL-12/23 inhibitors, IL-23 inhibitors, IL-17 inhibitors, or deucravacitinib, anti-tuberculosis treatment can be given concurrently.
**Recommendation 39**
In patients with psoriasis and latent tuberculosis, when biologic therapy is indicated, we recommend preferential use of IL-17 or IL-23 inhibitors because of a lower reactivation risk.
*Strong recommendation for, low-certainty evidence*
**Recommendation 40**
In patients with psoriasis and latent tuberculosis, we suggest considering acitretin, apremilast, or phototherapy as safe systemic treatment options.
*Conditional recommendation for, low-certainty evidence*

### Evidence synthesis

Phototherapy, acitretin, and apremilast are considered safe systemic options for patients with psoriasis and latent tuberculosis. Evidence on biologics shows an overall reactivation rate that is close to zero (0.0%; 95% CI 0%–0.21%), although risk is higher with anti-TNF agents (1.3%).^91^ Anti-TNF agents and ustekinumab should be avoided or discontinued in active tuberculosis and restarted only after clinical control with close monitoring. Current recommendations prioritize IL-17 or IL-23 inhibitors, particularly in individuals with high exposure risk or limited access to prophylaxis.

Universal screening for latent tuberculosis infection and prophylaxis per local guidance are mandatory before biologic initiation. Cohort studies show that adherence to these strategies reduces active tuberculosis incidence by up to ten-fold in patients treated with anti-TNF agents.^92^

### From evidence to decision

Experts emphasized that the high prevalence of latent and active tuberculosis in Latin America poses a safety challenge for biologic therapy. The highest reactivation risk is seen with anti-TNF agents and cyclosporine; initiating latent tuberculosis treatment at least 1 month before starting these agents is recommended. For IL-12/23, IL-23, IL-17 inhibitors, or deucravacitinib, treatment of latent infection can be administered concomitantly. For patients with active tuberculosis, biologic therapy should be stopped until the first phase of treatment has been completed.

### Pediatric psoriasis

**Table:** 

**Recommendation 41**
In pediatric patients with mild psoriasis requiring systemic therapy, we suggest methotrexate at 10–15 mg/m^2^ body surface area per week, corresponding approximately to 0.2–0.4 mg/kg/week in most pediatric patients, with a maximum dose of 25 mg per week.
*Observations: The weight-based dose is provided to facilitate clinical use and should be interpreted as an approximate equivalent of body surface area-based dosing. Add folic acid supplementation and perform periodic monitoring for hepatotoxicity and myelosuppression. Subcutaneous administration may be considered in patients with gastrointestinal intolerance to oral therapy.*
*Conditional recommendation for, very low-certainty evidence*
**Recommendation 42**
In pediatric patients with psoriasis with acute exacerbations, pustular psoriasis, or methotrexate failure or contraindication, we suggest cyclosporine (2.5–5 mg/kg/day).
*Observations: Monitor renal and hepatic function and avoid treatment beyond 12 months because of toxicity risk.*
*Conditional recommendation for, very low-certainty evidence*
**Recommendation 43**
In pediatric patients aged 6–17 years with moderate-to-severe psoriasis, or with failure of or contraindications to conventional treatments, we recommend etanercept (0.8 mg/kg, maximum 50 mg, administered subcutaneously once weekly). Treatment response should be assessed at 12 weeks.
*Strong recommendation for, high-certainty evidence*
**Recommendation 44**
In pediatric patients aged 4–17 years with moderate-to-severe psoriasis, or with failure of or contraindications to conventional treatments, we recommend adalimumab (20 mg every 2 weeks in patients weighing 15 to < 30 kg; 40 mg every 2 weeks in patients weighing ≥ 30 kg). Treatment response should be assessed at 16 weeks.
*Strong recommendation for, moderate-certainty evidence*
**Recommendation 45**
In pediatric patients aged 6 years or older with moderate-to-severe psoriasis, or with failure of or contraindications to conventional treatments, we recommend ixekizumab (40 mg in patients weighing < 25 kg, 80 mg in patients weighing 25–50 kg, and a 160 mg loading dose followed by 80 mg maintenance dosing in patients weighing > 50 kg) or secukinumab (75 mg in patients weighing < 25 kg and 150 mg in patients weighing ≥ 25 kg). Treatment response should be assessed at 16 weeks.
*Contingent on approval of this indication in the country.
*Strong recommendation for, moderate-certainty evidence*
**Recommendation 46**
In pediatric patients aged 4 years or older with moderate-to-severe psoriasis, or with failure of or contraindications to conventional treatments, we recommend ustekinumab (0.75 mg/kg in patients weighing <60 kg; 45 mg in patients weighing 60–100 kg; 90 mg in patients weighing >100 kg, administered at weeks 0 and 4, then every 12 weeks). Treatment response should be assessed at 16 weeks.
*Contingent on approval of this indication in the country.
*Strong recommendation for, low-certainty evidence*
**Recommendation 47**
In pediatric patients with pediatric pustular psoriasis or other exceptional cases as third-line therapy, we suggest considering acitretin (0.5–1 mg/kg/day) with strict clinical and laboratory monitoring.
*Conditional recommendation for, very low-certainty evidence*

### Evidence synthesis

Evidence on conventional therapies in pediatric psoriasis is limited. Methotrexate is recommended as the first systemic option, while cyclosporine is reserved for acute flares, pustular psoriasis, or methotrexate failure/contraindication, and should be limited to courses under 1 year because of toxicity. Acitretin can be considered a third-line option for pediatric pustular psoriasis or refractory pediatric psoriasis when methotrexate and cyclosporine have failed, are contraindicated, or are unsuitable. This statement is based on limited evidence and expert consensus. The best available pediatric evidence comes from case series in generalized pustular psoriasis, where low-dose acitretin was associated with clinical response, but no randomized controlled trials are available in this population.^93^ Close monitoring for mucocutaneous adverse effects, lipid abnormalities, liver function, and potential effects on bone growth is required. In girls of early reproductive potential, the prolonged teratogenic risk of acitretin requires careful counseling and consideration of alternative systemic agents.

Evidence on biologics and small molecules in pediatric populations shows sustained efficacy and acceptable safety profiles. Among anti-TNF agents, adalimumab was superior to methotrexate (PASI75: 58% at week 16), and etanercept achieved PASI75 in 55%–57% versus 11% with placebo at week 12, with quality-of-life improvement and responses maintained for up to 5 years.^94^ Among IL-17 and IL-23 inhibitors, secukinumab achieved PASI75 responses above 90% and PASI90 near 70% at week 12, with efficacy maintained through 104 weeks and good tolerability across age groups.^95^ Ustekinumab achieved PASI75 in about 80% and PGA 0/1 in 69% of adolescents at week 12, while ixekizumab showed high response rates (PASI75: 90%, PASI90: 78%, PASI100: 50%) maintained through week 108. Apremilast showed more modest efficacy (PASI75: 45% vs. 16% with placebo at week 16) with a favorable safety profile.

Consensus guidance on methotrexate for pediatric inflammatory skin disease supports weight-based dosing and monitoring in children, including patients treated for psoriasis.^96^

### From evidence to decision

Recommendations for pediatric psoriasis are constrained by limited studies in this age group. Experts agreed that methotrexate and cyclosporine can be used safely and effectively based on clinical experience. Cyclosporine should be used as bridge therapy and for periods under 1 year. Acitretin, supported by case series, does not appear to affect growth, but requires special caution in girls of early reproductive potential. Biologics are the only therapies specifically approved for pediatric psoriasis beginning at age 4 or 6 years; access in the region remains limited by regulatory constraints and administrative barriers.

### Nonpharmacologic treatment

**Table:** 

**Recommendation 48**
In patients with psoriasis who are overweight or obese, we recommend a supervised caloric reduction plan to reduce disease activity and enhance response to systemic therapies.
*Strong recommendation for, moderate-certainty evidence*
**Recommendation 49**
In patients with psoriasis, we recommend referral to structured psychological support, given the impact of emotional burden on adherence and illness perception.
*Strong recommendation for, moderate-certainty evidence*

### Evidence synthesis

Nutritional assessment and a supervised caloric reduction plan in overweight or obese patients are recommended nonpharmacologic interventions in psoriasis management. These measures support improved control of comorbidities and reduced disease activity.^97^ Moderate-quality evidence also supports structured, sustained psychological interventions as useful adjuncts to reduce anxiety, decrease depressive symptoms, and improve quality of life in patients with psoriasis.[98], [99]

For interventions such as the Mediterranean diet, probiotics, fatty acids, and other micronutrients, the evidence is limited and methodologically heterogeneous,[97], [100] with a predominance of observational studies, small sample sizes, or inconsistent results.

### From evidence to decision

Experts recommended incorporating nutritional assessment and psychological support as integral components of psoriasis care, given the effect of excess weight and emotional burden on disease control. A multidisciplinary approach that supports adherence and reduces risk factors that can increase flare frequency or reduce medication effectiveness is needed. In this context, nonpharmacologic interventions should be treated as complementary strategies in psoriasis care.

Although anti-inflammatory dietary patterns may be associated with lower clinical severity and may have adjunct effects on cutaneous inflammation and metabolic comorbidities, the current evidence base is inconsistent, and disparities in access to certain foods across Latin American populations prevent strong recommendations.

## Guideline implementation considerations

### Patient perspective

Patients with psoriasis commonly describe a profound impact of the disease on emotional, social, and psychological well-being. The visibility of cutaneous lesions, especially during childhood and adolescence, affects self-image and can lead to the pursuit of treatments without scientific support. Limited general awareness of the disease contributes to stigma, social isolation, and discrimination, which worsen mental health and quality of life.

Education of the community and healthcare personnel reduces stigma, strengthens self-care, and supports adherence. Education targeted to patients and caregivers is needed, with clear, accurate, and culturally adapted information on the chronic and autoimmune nature of psoriasis, disease course, comorbidities, and triggers of acute flares to support empowerment in disease control. Patient groups are highly valued as spaces for education, guidance, and emotional support, facilitating acceptance of the disease and adaptation to daily life.

In care delivery, patients particularly value the possibility of remission or visible improvement that allows them to regain a sense of control. Treatment efficacy and safety play a central role in quality-of-life improvement. Clear explanation of available treatment options, risks and benefits, potential adverse effects, and the possibility of therapeutic failure supports realistic expectations for each intervention. Limited availability of treatments in some countries, out-of-pocket costs, administrative delays, and shortages of dermatologists or specialized centers create barriers to successful care.

In this context, patients advocate for guaranteed access to effective therapies and trained professionals, within an interdisciplinary model that integrates ongoing psychological support and health education as core components. Patients also emphasize strengthening psoriasis support networks as a strategy to improve adherence and reduce the emotional, psychological, and social burden associated with the disease.

### Barriers and facilitators

Implementation of this guideline faces challenges related to access barriers, particularly for medications such as acitretin and biologic agents, reflecting differences in commercialization, health system organization, and resource allocation across Latin American countries. Inclusion of biologics in health coverage for patients who truly need them, along with rational use of biosimilars, can support adoption and sustainability of the recommendations, provided bio-comparability is ensured and active pharmacovigilance is maintained to monitor ongoing efficacy and safety.

Management of psoriasis in special situations requires a multidisciplinary approach that integrates comorbidity and risk management, including screening, prophylaxis, and vaccination for infectious diseases. Availability and accessibility of therapies for these conditions must be ensured so they do not interfere with the efficacy or safety of psoriasis-specific treatment.

Regionally generated evidence is needed to strengthen clinical decision-making, support implementation, and inform future updates of psoriasis recommendations in Latin America. Studies should prioritize psoriasis in high-impact areas, pediatric populations, special clinical situations, treatment sequencing, biosimilar use, long-term safety, patient-reported outcomes, and real-world effectiveness of biologic and oral targeted therapies.

This regional psoriasis guideline supports comprehensive care while recognizing that implementation depends on national formularies, reimbursement pathways, drug availability, phototherapy access, patient preferences, and local regulatory requirements. This implementation perspective is consistent with the need to interpret regional recommendations alongside country-specific guidance. The 2024 Brazilian Consensus on Psoriasis and Treatment Algorithm of the Sociedade Brasileira de Dermatologia, for example, similarly includes spesolimab and cyclosporine as first-line treatments for generalized pustular psoriasis.^13^ The main difference is the role of acitretin in plaque psoriasis: the Brazilian algorithm no longer includes it in the treatment sequence, whereas SOLAPSO retains acitretin as a first-line systemic option for selected patients because it remains available and affordable in several Latin American settings. This divergence reflects the distinction between a country-specific algorithm and a regional guideline developed for health systems with variable access to advanced therapies.

Monitoring implementation of clinical guidance for psoriasis treatment should be done using specific indicators. Supplementary Material 4 presents a general matrix that should be reviewed and adapted for each country.

## Research data availability

All data supporting this guideline are derived from published sources identified through the systematic searches described in Supplementary material 2. Evidence summaries and EtD frameworks are available in Supplementary material 3.

## Authors' contributions

Fernando Valenzuela: Approval of the final version of the manuscript; manuscript critical review; preparation and writing of the manuscript; study conception and planning.

Juan Raúl Castro Ayarza: Approval of the final version of the manuscript; manuscript critical review; preparation and writing of the manuscript; study conception and planning.

Débora Kaplan: Approval of the final version of the manuscript; manuscript critical review; preparation and writing of the manuscript; study conception and planning.

Angela María Londoño-García: Approval of the final version of the manuscript; manuscript critical review; preparation and writing of the manuscript; study conception and planning.

Karla del Rocío Macías García: Approval of the final version of the manuscript; manuscript critical review; preparation and writing of the manuscript; study conception and planning.

Gabriel Magariños: Approval of the final version of the manuscript; manuscript critical review; preparation and writing of the manuscript; study conception and planning.

Enrique Salvador Rivas Záldivar: Approval of the final version of the manuscript; manuscript critical review; preparation and writing of the manuscript; study conception and planning.

Manuel Franco: Approval of the final version of the manuscript; manuscript critical review; preparation and writing of the manuscript; study conception and planning.

Sara Cano: Approval of the final version of the manuscript; manuscript critical review; preparation and writing of the manuscript; study conception and planning.

Carolina Cortés: Approval of the final version of the manuscript; manuscript critical review; preparation and writing of the manuscript; study conception and planning.

Paola Jimena Cárdenas: Approval of the final version of the manuscript; manuscript critical review; preparation and writing of the manuscript; study conception and planning.

Carla Castro: Approval of the final version of the manuscript; manuscript critical review; preparation and writing of the manuscript; study conception and planning.

Renata Ferreira: Approval of the final version of the manuscript; manuscript critical review; preparation and writing of the manuscript; study conception and planning.

Susan Martínez: Approval of the final version of the manuscript; data collection, analysis and interpretation; manuscript critical review; preparation and writing of the manuscript.

Linda Ibatá Bernal: Approval of the final version of the manuscript; data collection, analysis and interpretation; manuscript critical review; preparation and writing of the manuscript.

Julieth Carolina Castillo: Approval of the final version of the manuscript; data collection, analysis and interpretation; manuscript critical review; preparation and writing of the manuscript.

Jorge Ospina: Approval of the final version of the manuscript; data collection, analysis and interpretation; manuscript critical review; preparation and writing of the manuscript.

## Financial support

The development of this clinical practice guideline was financially supported by Eli Lilly Mexico, 10.13039/100004331Johnson & Johnson Colombia, 10.13039/100004336Novartis Colombia, and UCB Biopharma SRL. Scientific and academic work was led independently by SOLAPSO and EpiThink Health Consulting. Funders had no role in, and did not influence, the guideline’s development, content, deliberations, decisions, or final text.

## Declaration of competing interest

All participants completed detailed declarations of interest (available in the Supplementary Material). A centralized review determined that no conflicts of interest limited participation in decision-making.

All authors completed disclosures of financial and personal relationships that could be perceived as influencing this work. The complete disclosures are available in Supplementary material 1. The centralized review determined that no conflicts of interest restricted participation in guideline decisions.
