# Supplementary material for: Clinical practice guideline for psoriasis management in Latin America
Source: An Bras Dermatol. 2026 Aug 29;101(5):501449. doi: 10.1016/j.abd.2026.501449 (PMC13545376; doi:10.1016/j.abd.2026.501449)
Supplement: Supplementary file 1 [file mmc1.docx]

**CLINICAL PRACTICE GUIDELINE FOR PSORIASIS MANAGEMENT IN LATIN AMERICA**

**Latin American Psoriasis Society – SOLAPSO**

**Supplementary material 1. Conflict of interest disclosure**

**PARTICIPANTS**

**Development Group**

**Fernando Valenzuela, MD.** Specialist in dermatology and venereology, psoriasis fellow, clinical teaching. Centro Internacional de Estudios Clínicos, Universidad de Chile. Santiago, Chile.

**Juan Raúl Castro Ayarza, MD.** Dermatology specialist. Specialist in university teaching, master’s degree in dermatologic oncology, master’s degree in epidemiology. Medicarte IPS, Clínica del Country, Universidad Nacional de Colombia, COLPSOR, Colombia.

**Débora Kaplan, MD.** Dermatology specialist. Private practice. Buenos Aires, Argentina. Former president of the Argentine Psoriasis Society.

**Angela María Londoño García, MD.** Dermatology specialist, master’s degree in epidemiology, master’s degree in autoimmunity. CLIPSO, HELPHARMA, Universidad CES. Medellín, Colombia.

**Karla del Rocío Macías García, MD.** Dermatology specialist. Dermatológico Country. Guadalajara, Mexico.

**Gabriel Magariños, MD.** Dermatology specialist, dermatopathology. Hospital Municipal Dr. Bernardo Houssay, Psoriahue, Universidad del Salvador. Buenos Aires, Argentina.

**Enrique Salvador Rivas Záldivar, MD.** Dermatology specialist. DERMOS Centro de Dermatología, Guatemala

**Extended Expert Panel**

**Manuel Franco, MD.** Dermatology specialist. Universidad El Bosque, Medicarte IPS. Colombia.

**Sara Cano, MD.** Dermatology specialist. Master’s degree in trichology. Universidad ICESI, Cali, Colombia.

**Carolina Cortés, MD.** Dermatology specialist. Hospital Universitario La Samaritana. Bogotá D.C., Colombia.

**Paola Jimena Cárdenas, MD.** Dermatology specialist. Master’s degree in dermatologic oncology. Master’s degree in public health. Virrey Solís IPS. Bogotá D.C., Colombia.

**Carla Castro, MD.** Dermatology specialist. Specialist in pediatric dermatology. Universidad Católica de Cuyo, Hospital Universitario Austral. Argentina.

**Renata Ferreira, MD.** Dermatology specialist. Universidade Estadual de Campinas. Brazil.

**Patient Representatives**

Simone Canelada (Brazil).

Jorge Espinosa (Mexico).

Silvia Fernández Barrios (Argentina).

Pável Ernesto García (Guatemala).

Guillermo Gutiérrez (Colombia).

Sarai Ponce (Chile).

**Methodological Team**

Susan Martínez, MD. Epidemiology specialist. Master’s degree in public health. EpiThink Health Consulting.

Linda Ibatá Bernal, MD. Epidemiology specialist. Master’s degree in public health. EpiThink Health Consulting.

Julieth Carolina Castillo, RN. Epidemiology specialist. Master’s degree in public health. EpiThink Health Consulting.

Jorge Ospina, MD. Master’s degree in pharmacology. EpiThink Health Consulting.

**DECLARATION OF INTERESTS**

Participants in the development of this clinical practice guideline completed a declaration of interests using a pre-established format, following the Manual de procesos participativos of the Instituto de Evaluación Tecnológica en Salud (IETS).¹ The results of the conflict-of-interest assessment and the decisions regarding unrestricted or limited participation are described below:

**Conflicts of interest and participation review**

| Member | Disclosure | Participation decision |
| --- | --- | --- |
| Clinical experts | | |
| Fernando Valenzuela | Has served as a speaker for Pfizer, AbbVie, Janssen, and Eli Lilly. | Unrestricted participation |
| Juan Raúl Castro Ayarza | Has served as a speaker for AbbVie, Eli Lilly, Pharmalab, Novartis, and Janssen. | Unrestricted participation |
| Débora Kaplan | Has served as a speaker for AbbVie, Novartis, BMS, Eli Lilly, RAFFO, Fortbenton, and Janssen. | Unrestricted participation |
| Angela María Londoño García | Has served as a speaker for Lilly, AbbVie, and Janssen. | Unrestricted participation |
| Karla del Rocío Macías García | Has served as a speaker for Eli Lilly, AbbVie, UCB Pharma, Novartis, BMS, Pfizer, and Leo Pharma. | Unrestricted participation |
| Gabriel Alejandro Magariños | Has participated in clinical research for Bristol, Takeda, Novartis, Sanofi, Eli Lilly, and Janssen. | Unrestricted participation |
| Enrique Salvador Rivas Záldivar | Has served as a speaker for Novartis and AbbVie. | Unrestricted participation |
| Extended panel | | |
| Manuel Franco | Has served as a speaker for AbbVie, Eli Lilly, Pfizer, Novartis, Johnson & Johnson, BMS, and Boehringer Ingelheim. | Unrestricted participation |
| Sara Cano | Has served as a speaker for Argen, AbbVie, Biopas, Boehringer Ingelheim, Pfizer, Eli Lilly, BMS, Janssen, and Novartis. | Unrestricted participation |
| Carolina Cortés | Has served as a speaker for Sanofi and Pfizer. | Unrestricted participation |
| Paola Jimena Cárdenas | Has served as a speaker for Janssen, AbbVie, Eli Lilly, and Novartis. | Unrestricted participation |
| Carla Castro | Has served as a speaker for Novartis, BMS, AbbVie, Janssen, and Eli Lilly. | Unrestricted participation |
| Renata Ferreira | Has served as a speaker for AbbVie, Pfizer, Novartis, Eli Lilly, Boehringer Ingelheim, Sanofi, BMS, UCB, and Galderma. | Unrestricted participation |
| Methodological experts (EpiThink Health Consulting) | | |
| Susan Martínez | EpiThink received payment from SOLAPSO for the development of this guideline. EpiThink has provided consulting services to multiple pharmaceutical companies and scientific societies. | Unrestricted participation |
| Linda Ibatá Bernal | EpiThink received payment from SOLAPSO for the development of this guideline. EpiThink has provided consulting services to multiple pharmaceutical companies and scientific societies. | Unrestricted participation |
| Julieth Carolina Castillo | EpiThink received payment from SOLAPSO for the development of this guideline. EpiThink has provided consulting services to multiple pharmaceutical companies and scientific societies. | Unrestricted participation |
| Jorge Ospina | EpiThink received payment from SOLAPSO for the development of this guideline. EpiThink has provided consulting services to multiple pharmaceutical companies and scientific societies. | Unrestricted participation |
| Patient representatives | | |
| Patient representatives invited to participate are or have been members of organizations such as AEPSO (Asociación Civil para el Enfermo de Psoriasis), IFPA (International Federation of Psoriasis Associations), Fundapso, and Fundación Padece. Their participation in this consensus is in a personal capacity as individuals with psoriasis, and their opinions do not represent the position of those organizations. | | |
