# Supplementary material for: Clinical practice guideline for psoriasis management in Latin America
Source: An Bras Dermatol. 2026 Aug 29;101(5):501449. doi: 10.1016/j.abd.2026.501449 (PMC13545376; doi:10.1016/j.abd.2026.501449)
Supplement: Supplementary file 2 [file mmc2.docx]

**CLINICAL PRACTICE GUIDELINE FOR PSORIASIS MANAGEMENT IN LATIN AMERICA**

**Latin American Psoriasis Society – SOLAPSO**

**Supplementary Material 2. Search strategies, evidence selection, and evidence synthesis**

# SYSTEMATIC SEARCHES AND LITERATURE SELECTION

## THERAPEUTIC TARGETS

Evidence search strategies

Database: Medline

Platform: Ovid

Search date: 06/24

Search strategy:

#1, ('psoriasis' or 'psoriasiform dermatitis' or 'psoriasiform dermatosis' or 'psoriasiform lesion' or 'psoriasiform rash' or 'psoriasiform skin rash' or 'psoriasis' or 'psoriatic epidermis' or 'psoriatic skin' or 'pustular psoriasis' or 'psoriasis pustulosa' or 'pustular psoriasis' or 'pustulous psoriasis' or 'pustulosis palmoplantaris' or 'chronic palmoplantar pustular psoriasis' or 'palmar plantar psoriasis' or 'palmo-plantar psoriasis' or 'palmoplantar psoriasis' or 'palmoplantar pustular psoriasis').ti,ab,kw., 31

#2, (('psoriasis area and severity index') or 'pasi' or 'psoriasis area severity index' or 'severity index' or 'body surface' or 'area, body surface' or 'body surface area' or 'surface, body' or `bsa` or 'physician global assessment' or 'physician global assessment score' or 'physician global assessment of disease activity' or 'physician global assessment scale' or `pga` or 'psoriasis symptom inventory' or `psi` or 'dermatology life quality index' or 'dlqi' or 'dermatologic life quality index' or 'dermatological life quality index' or 'dermatology quality of life index' or 'dermatology life quality index questionnaire' or 'dermatology life quality index score' or 'investigator global assessment' or 'investigator global assessment score' or 'investigator global assessment scale' or napsi or 'nail psoriasis severity index' or 'nail psoriasis severity index score').ti,ab,kw., 48

#3, 1 and 2, 12

Database: Embase

Platform: Embase

Search date: 06/24

Search strategy (results) #1,’'psoriasis'/mj OR 'psoriasiform dermatitis' OR 'psoriasiform dermatosis' OR 'psoriasiform lesion' OR 'psoriasiform rash' OR 'psoriasiform skin rash' OR 'psoriasis' OR 'psoriatic epidermis' OR 'psoriatic skin' OR 'pustular psoriasis'/mj OR 'psoriasis pustulosa' OR 'pustular psoriasis' OR 'pustulous psoriasis' OR 'pustulosis palmoplantaris'/mj OR 'chronic palmoplantar pustular psoriasis' OR 'palmar plantar psoriasis' OR 'palmo-plantar psoriasis' OR 'palmoplantar psoriasis' OR 'palmoplantar pustular psoriasis'‘,114973

#2,’'psoriasis area and severity index'/mj OR 'pasi (psoriasis area severity index)' OR 'psoriasis area severity index' OR `pasi` OR 'severity index'/mj OR 'body surface'/mj OR 'area, body surface' OR 'body surface area' OR 'surface, body' OR `bsa` OR 'physician global assessment'/mj OR 'physician global assessment score'/mj OR 'physician global assessment of disease activity'/mj OR 'physician global assessment scale'/mj OR `pga` OR 'psoriasis symptom inventory'/mj OR `psi` OR 'dermatology life quality index'/mj OR 'dlqi' OR 'dermatologic life quality index' OR 'dermatological life quality index' OR 'dermatology quality of life index' OR 'dermatology life quality index questionnaire'/mj OR 'dermatology life quality index score'/mj OR 'investigator global assessment'/mj OR 'investigator global assessment score'/mj OR 'investigator global assessment scale'/mj OR napsi OR 'nail psoriasis severity index'/mj OR 'nail psoriasis severity index score'/mj’,123160

#3,’'goal setting'/mj OR 'therapeutic goal' OR 'treatment target' OR 'clinical goals'‘,11320

#4,’#2 AND #3’,127

#5,’#1 AND #4’,59

Database: Cochrane Database of Systematic Reviews – Database of Abstracts of Reviews of Effects

Platform: PubMed

Search date: 06/24

Search strategy:

#1,’(‘‘Psoriasis’’[Mesh]) OR ('psoriasis' or 'psoriasiform dermatitis' or 'psoriasiform dermatosis' or 'psoriasiform lesion' or 'psoriasiform rash' or 'psoriasiform skin rash' or 'psoriasis' or 'psoriatic epidermis' or 'psoriatic skin' or 'pustular psoriasis' or 'psoriasis pustulosa' or 'pustular psoriasis' or 'pustulous psoriasis' or 'pustulosis palmoplantaris' or 'chronic palmoplantar pustular psoriasis' or 'palmar plantar psoriasis' or 'palmo-plantar psoriasis' or 'palmoplantar psoriasis' or 'palmoplantar pustular psoriasis')’,’68,718’

#2,’'psoriasis area and severity index' or 'pasi' or 'psoriasis area severity index' or 'severity index' or ‘‘Body Surface Area’’[Mesh] or 'body surface' or 'area, body surface' or 'body surface area' or 'surface, body' or `bsa` or 'physician global assessment' or 'physician global assessment score' or 'physician global assessment of disease activity' or 'physician global assessment scale' or `pga` or 'psoriasis symptom inventory' or `psi` or 'dermatology life quality index' or 'dlqi' or 'dermatologic life quality index' or 'dermatological life quality index' or 'dermatology quality of life index' or 'dermatology life quality index questionnaire' or 'dermatology life quality index score' or 'investigator global assessment' or 'investigator global assessment score' or 'investigator global assessment scale' or napsi or 'nail psoriasis severity index' or 'nail psoriasis severity index score'‘,’607,412’

#3,'goal setting' OR 'therapeutic goal' OR 'treatment target' OR 'clinical goals',’1,288,994’

#4, 2 AND 3,’27,348’

#5, 1 AND 4,’1,184’

#6, 5 AND (review [Filter] OR systematic review [Filter])’, 277

#7, 6 AND (y_5[Filter]),108

## CONVENTIONAL SYSTEMIC TREATMENT

Evidence search strategies

Database: Medline

Platform: Ovid

Search date: 02/25

Search strategy:

#1, ('psoriasis' or 'psoriasiform dermatitis' or 'psoriasiform dermatosis' or 'psoriasiform lesion' or 'psoriasiform rash' or 'psoriasiform skin rash' or 'psoriasis' or 'psoriatic epidermis' or 'psoriatic skin' or 'pustular psoriasis' or 'psoriasis pustulosa' or 'pustular psoriasis' or 'pustulous psoriasis' or 'pustulosis palmoplantaris' or 'chronic palmoplantar pustular psoriasis' or 'palmar plantar psoriasis' or 'palmo-plantar psoriasis' or 'palmoplantar psoriasis' or 'palmoplantar pustular psoriasis').ti,ab,kw., 31

#2, ('acitren' or 'acitretin' or 'acitretina' or 'acitretine' or 'acitretinoin').ti,ab,kw.,

#3, ('cyclosporine' or 'ciclosporin' or 'ciclosporin a' or 'ciclosporine' or 'cyclosporin' or 'cyclosporin a' or 'cyclosporine' or 'cyclosporine a').ti,ab,kw., 73

#4, ('methotrexate' or 'mtx' or 'methohexate' or 'methotrate' or 'methotrexat' or 'methotrexat ebewe' or 'methotrexate' or 'methotrexate disodium' or 'methotrexate lpf' or 'methotrexate sodium' or 'methotrexate sodium preservative free' or 'methotrexate-lde' or 'methotrexato' or 'methoxtrexate' or 'methrotrexate' or 'metothrexate' or 'metothrexate sodium' or 'metotrexat' or 'metotrexate').ti,ab,kw., 84

#5, 1 or 2 or 3 or 4, 161

Database: Embase

Platform: Embase

Search date: 02/25

Search strategy:

#1,’'psoriasis'/mj OR 'psoriasiform dermatitis' OR 'psoriasiform dermatosis' OR 'psoriasiform lesion' OR 'psoriasiform rash' OR 'psoriasiform skin rash' OR 'psoriasis' OR 'psoriatic epidermis' OR 'psoriatic skin' OR 'pustular psoriasis'/mj OR 'psoriasis pustulosa' OR 'pustular psoriasis' OR 'pustulous psoriasis' OR 'pustulosis palmoplantaris'/mj OR 'chronic palmoplantar pustular psoriasis' OR 'palmar plantar psoriasis' OR 'palmo-plantar psoriasis' OR 'palmoplantar psoriasis' OR 'palmoplantar pustular psoriasis'‘,116292

#2,’'acitretin'/mj OR 'acitren' OR 'acitretin' OR 'acitretina' OR 'acitretine' OR 'acitretinoin'‘,9218

#3,’'cyclosporine'/mj OR 'ciclosporin' OR 'ciclosporin a' OR 'ciclosporine' OR 'cyclosporin' OR 'cyclosporin a' OR 'cyclosporine' OR 'cyclosporine a'‘,187011

#4,’'methotrexate'/mj OR 'mtx' OR 'methohexate' OR 'methotrate' OR 'methotrexat' OR 'methotrexat ebewe' OR 'methotrexate' OR 'methotrexate disodium' OR 'methotrexate lpf' OR 'methotrexate sodium' OR 'methotrexate sodium preservative free' OR 'methotrexate-lde' OR 'methotrexato' OR 'methoxtrexate' OR 'methrotrexate' OR 'metothrexate' OR 'metothrexate sodium' OR 'metotrexat' OR 'metotrexate'‘,236551

#5,’#2 OR #3 OR #4’,389605

#6,’#1 AND #5’,19923

#7,’#1 AND #5 AND ([cochrane review]/lim OR [systematic review]/lim OR [meta analysis]/lim) AND [2019-2025]/py’,422

Database: Cochrane Database of Systematic Reviews - Database of Abstracts of Reviews of Effects

Platform: Pubmed

Search date: 02/25

Search strategy:

#1,’(‘‘Psoriasis’’[Mesh]) OR ('psoriasis' or 'psoriasiform dermatitis' or 'psoriasiform dermatosis' or 'psoriasiform lesion' or 'psoriasiform rash' or 'psoriasiform skin rash' or 'psoriasis' or 'psoriatic epidermis' or 'psoriatic skin' or 'pustular psoriasis' or 'psoriasis pustulosa' or 'pustular psoriasis' or 'pustulous psoriasis' or 'pustulosis palmoplantaris' or 'chronic palmoplantar pustular psoriasis' or 'palmar plantar psoriasis' or 'palmo-plantar psoriasis' or 'palmoplantar psoriasis' or 'palmoplantar pustular psoriasis')’,’68,786’

#2, (‘Acitretin’[Mesh]) OR ('acitretin'[Title/Abstract] OR 'acitretina'[Title/Abstract] OR 'acitretine'[Title/Abstract]), 2198

#3, (‘Cyclosporine’[Mesh]) OR ('ciclosporin'[Title/Abstract] OR 'ciclosporin a'[Title/Abstract] OR 'ciclosporine'[Title/Abstract] OR 'cyclosporin'[Title/Abstract] OR 'cyclosporin a'[Title/Abstract] OR 'cyclosporine'[Title/Abstract] OR 'cyclosporine a'[Title/Abstract]), 61295

#4, (‘Methotrexate’[Mesh]) OR ('mtx' OR 'methotrexat' OR 'methotrexat ebewe' OR 'methotrexate' OR 'methotrexate disodium' OR 'methotrexate lpf' OR 'methotrexate sodium' OR 'methotrexate sodium preservative free' OR 'methotrexate lde' OR 'methotrexato' OR 'methoxtrexate' OR 'methrotrexate' OR 'metothrexate' OR 'metothrexate sodium' OR 'metotrexat' OR 'metotrexate'), 65176

#5, ‘#2 OR #3 OR #4’, 124055

#6,’#1 AND #5’, 5657

#7, #6 Filters: in the last 5 years, Meta-Analysis, Systematic Review, 69

## TREATMENT WITH BIOLOGIC AGENTS AND SMALL MOLECULES

Evidence search strategies

Database: Medline

Platform: Ovid

Search date: 02/25

Search strategy:

#1, ('psoriasis' or 'psoriasiform dermatitis' or 'psoriasiform dermatosis' or 'psoriasiform lesion' or 'psoriasiform rash' or 'psoriasiform skin rash' or 'psoriasis' or 'psoriatic epidermis' or 'psoriatic skin' or 'pustular psoriasis' or 'psoriasis pustulosa' or 'pustular psoriasis' or 'pustulous psoriasis' or 'pustulosis palmoplantaris' or 'chronic palmoplantar pustular psoriasis' or 'palmar plantar psoriasis' or 'palmo-plantar psoriasis' or 'palmoplantar psoriasis' or 'palmoplantar pustular psoriasis').ti,ab,kw., 31

#2, ('adalimumab' or 'apremilast' or 'bimekizumab' or 'brodalumab' or 'certolizumab pegol' or 'pegylated tumor necrosis factor alpha antibody fab fragment' or 'pegylated tumour necrosis factor alpha antibody fab fragment' or 'deucravacitinib' or 'etanercept' or 'recombinant tumor necrosis factor receptor fc fusion protein' or 'recombinant tumour necrosis factor receptor fc fusion protein' or 'tumor necrosis factor receptor fc fusion protein' or 'tumour necrosis factor receptor fc fusion protein' or 'golimumab' or 'guselkumab' or 'infliximab' or 'ixekizumab' or 'risankizumab' or 'secukinumab' or 'spesolimab' or 'ustekinumab').ti,ab,kw., 68

#3, 1 AND 2, 93

Database: Embase

Platform: Embase

Search date: 02/25

Search strategy:

#1,’'psoriasis'/mj OR 'psoriasiform dermatitis' OR 'psoriasiform dermatosis' OR 'psoriasiform lesion' OR 'psoriasiform rash' OR 'psoriasiform skin rash' OR 'psoriasis' OR 'psoriatic epidermis' OR 'psoriatic skin' OR 'pustular psoriasis'/mj OR 'psoriasis pustulosa' OR 'pustular psoriasis' OR 'pustulous psoriasis' OR 'pustulosis palmoplantaris'/mj OR 'chronic palmoplantar pustular psoriasis' OR 'palmar plantar psoriasis' OR 'palmo-plantar psoriasis' OR 'palmoplantar psoriasis' OR 'palmoplantar pustular psoriasis'‘,117291

#2,’'adalimumab'/mj OR 'adalimumab' OR 'apremilast'/mj OR 'apremilast' OR 'bimekizumab'/mj OR 'bimekizumab' OR 'brodalumab'/mj OR 'brodalumab' OR 'certolizumab pegol'/mj OR 'certolizumab pegol' OR 'pegylated tumor necrosis factor alpha antibody fab fragment' OR 'pegylated tumour necrosis factor alpha antibody fab fragment' OR 'deucravacitinib'/mj OR 'deucravacitinib' OR 'etanercept'/mj OR 'recombinant tumor necrosis factor receptor fc fusion protein' OR 'recombinant tumour necrosis factor receptor fc fusion protein' OR 'tumor necrosis factor receptor fc fusion protein' OR 'tumour necrosis factor receptor fc fusion protein' OR 'golimumab'/mj OR 'golimumab' OR 'guselkumab'/mj OR 'guselkumab' OR 'infliximab'/mj OR 'infliximab' OR 'ixekizumab'/mj OR 'ixekizumab' OR 'risankizumab'/mj OR 'risankizumab' OR 'secukinumab'/mj OR 'secukinumab' OR 'spesolimab'/mj OR 'spesolimab' OR 'ustekinumab'/mj OR 'ustekinumab'‘,111847

#3,’#1 AND #2’,22869

#4,’#1 AND #2 AND ([cochrane review]/lim OR [systematic review]/lim OR [meta analysis]/lim)’,1435

#5,’#1 AND #2 AND ([cochrane review]/lim OR [systematic review]/lim OR [meta analysis]/lim) AND [2020-2025]/py’,713

Database: Cochrane Database of Systematic Reviews – Database of Abstracts of Reviews of Effects

Platform: PubMed

Search date: 02/25

Search strategy:

#1,(‘Psoriasis’[Mesh]) OR ('psoriasis'[Title/Abstract] OR 'psoriasiform dermatitis'[Title/Abstract] OR 'psoriasiform dermatosis'[Title/Abstract] OR 'psoriasiform lesion'[Title/Abstract] OR 'psoriasiform rash'[Title/Abstract] OR 'psoriasiform skin rash'[Title/Abstract] OR 'psoriasis'[Title/Abstract] OR 'psoriatic epidermis'[Title/Abstract] OR 'psoriatic skin'[Title/Abstract] OR 'pustular psoriasis'[Title/Abstract] OR 'psoriasis pustulosa'[Title/Abstract] OR 'pustular psoriasis'[Title/Abstract] OR 'pustulous psoriasis'[Title/Abstract] OR 'pustulosis palmoplantaris'[Title/Abstract] OR 'chronic palmoplantar pustular psoriasis'[Title/Abstract] OR 'palmar plantar psoriasis'[Title/Abstract] OR 'palmo-plantar psoriasis'[Title/Abstract] OR 'palmoplantar psoriasis'[Title/Abstract] OR 'palmoplantar pustular psoriasis'[Title/Abstract]), 68209

#2, ‘Adalimumab’[MeSH Terms] OR ‘apremilast’[Supplementary Concept] OR ‘bimekizumab’[Supplementary Concept] OR ‘brodalumab’[Supplementary Concept] OR ‘Certolizumab Pegol’[MeSH Terms] OR ‘deucravacitinib’[Supplementary Concept] OR ‘Etanercept’[MeSH Terms] OR ‘golimumab’[Supplementary Concept] OR ‘guselkumab’[Supplementary Concept] OR ‘Infliximab’[MeSH Terms] OR ‘ixekizumab’[Supplementary Concept] OR ‘risankizumab’[Supplementary Concept] OR ‘secukinumab’[Supplementary Concept] OR ‘spesolimab’[Supplementary Concept] OR ‘Ustekinumab’[MeSH Terms], 25767

#3, 'adalimumab'[Title/Abstract] OR 'apremilast'[Title/Abstract] OR 'bimekizumab'[Title/Abstract] OR 'brodalumab'[Title/Abstract] OR 'certolizumab pegol'[Title/Abstract] OR 'pegylated tumor necrosis factor alpha antibody fab fragment'[Title/Abstract] OR 'pegylated tumour necrosis factor alpha antibody fab fragment'[Title/Abstract] OR 'deucravacitinib'[Title/Abstract] OR 'etanercept'[Title/Abstract] OR 'recombinant tumor necrosis factor receptor fc fusion protein'[Title/Abstract] OR 'recombinant tumour necrosis factor receptor fc fusion protein'[Title/Abstract] OR 'tumor necrosis factor receptor fc fusion protein'[Title/Abstract] OR 'tumour necrosis factor receptor fc fusion protein'[Title/Abstract] OR 'golimumab'[Title/Abstract] OR 'guselkumab'[Title/Abstract] OR 'infliximab'[Title/Abstract] OR 'ixekizumab'[Title/Abstract] OR 'risankizumab'[Title/Abstract] OR 'secukinumab'[Title/Abstract] OR 'spesolimab'[Title/Abstract] OR 'ustekinumab'[Title/Abstract], 33284

#4, #2 OR #3, 37807

#5, #1 AND #4, 9163

#6, #5 Filters: in the last 5 years, Meta-Analysis, Systematic Review, 210

## BIOLOGIC TREATMENT FAILURE

Evidence search strategies

Database: Medline

Platform: Ovid

Search date: 02/25

Search strategy:

#1 ('plaque psoriasis' or 'psoriasis vulgaris' or 'chronic plaque psoriasis' or 'psoriasis, plaque' or 'plaque-type psoriasis' or 'psoriasis of the skin' or 'cutaneous psoriasis').ti,ab,kw.

#2 ('adalimumab' or 'etanercept' or 'infliximab' or 'certolizumab pegol' or 'golimumab' or

'ustekinumab' or 'secukinumab' or 'ixekizumab' or 'brodalumab' or 'bimekizumab' or

'guselkumab' or 'risankizumab' or 'tildrakizumab' or 'deucravacitinib' or 'apremilast' or

'tumor necrosis factor alpha inhibitor' or 'tumour necrosis factor alpha inhibitor' or 'TNF inhibitor' or 'interleukin 17 inhibitor' or 'IL-17 inhibitor' or 'interleukin 23 inhibitor' or 'IL-23 inhibitor' or 'interleukin 12 23 inhibitor' or 'IL-12/23 inhibitor' or 'biologic therapy' or 'biological therapy' or 'biologic agent' or 'biological agent' or 'biosimilar').ti,ab,kw.

#3 ('treatment failure' or 'therapeutic failure' or 'primary failure' or 'primary nonresponse' or 'primary non-response' or 'secondary failure' or 'secondary nonresponse' or 'secondary non-response' or 'loss of response' or 'loss of efficacy' or 'inadequate response' or 'insufficient response' or 'suboptimal response' or 'nonresponder' or 'non-responder' or

'refractory' or 'treatment discontinuation' or 'discontinuation' or 'switch' or 'switching' or 'treatment switch' or 'therapy switch' or 'escalation' or 'dose escalation' or 'dose increase' or

'optimization' or 'optimisation' or 'dose optimization' or 'dose adjustment' or 'rescue therapy').ti,ab,kw.

#4 (#1 and #2 and #3) 72

Database: Embase

Platform: Embase

Search date: 02/25

Search strategy:

#1, ('plaque psoriasis'/exp OR 'psoriasis vulgaris'/exp OR 'chronic plaque psoriasis':ti,ab,kw OR 'plaque psoriasis':ti,ab,kw OR 'psoriasis vulgaris':ti,ab,kw OR 'chronic plaque psoriasis':ti,ab,kw OR 'psoriasis, plaque':ti,ab,kw).ti,ab,kw.

#2, ('adalimumab'/exp OR 'etanercept'/exp OR 'infliximab'/exp OR 'certolizumab pegol'/exp OR 'golimumab'/exp OR 'ustekinumab'/exp OR 'secukinumab'/exp OR 'ixekizumab'/exp OR 'brodalumab'/exp OR 'bimekizumab'/exp OR 'guselkumab'/exp OR 'risankizumab'/exp OR 'tildrakizumab'/exp OR 'deucravacitinib'/exp OR 'apremilast'/exp OR 'tumor necrosis factor inhibitor'/exp OR 'interleukin 17 inhibitor'/exp OR 'interleukin 23 inhibitor'/exp OR 'interleukin 12 23 inhibitor'/exp OR 'biological therapy'/exp OR 'biosimilar'/exp OR adalimumab:ti,ab,kw OR etanercept:ti,ab,kw OR infliximab:ti,ab,kw OR 'certolizumab pegol':ti,ab,kw OR golimumab:ti,ab,kw OR ustekinumab:ti,ab,kw OR secukinumab:ti,ab,kw OR ixekizumab:ti,ab,kw OR brodalumab:ti,ab,kw OR bimekizumab:ti,ab,kw OR guselkumab:ti,ab,kw OR risankizumab:ti,ab,kw OR tildrakizumab:ti,ab,kw OR deucravacitinib:ti,ab,kw OR apremilast:ti,ab,kw OR ('TNF inhibitor*' OR 'IL-17 inhibitor*' OR 'IL17 inhibitor*' OR 'IL-23 inhibitor*' OR 'IL23 inhibitor*' OR 'IL-12/23 inhibitor*' OR biologic* OR biological* OR biosimilar*):ti,ab,kw).ti,ab,kw.

#3, ('treatment failure'/exp OR 'drug resistance'/exp OR 'therapy failure':ti,ab,kw OR 'treatment failure':ti,ab,kw OR 'therapeutic failure':ti,ab,kw OR 'primary failure':ti,ab,kw OR 'primary nonresponse':ti,ab,kw OR 'primary non-response':ti,ab,kw OR 'secondary failure':ti,ab,kw OR 'secondary nonresponse':ti,ab,kw OR 'secondary non-response':ti,ab,kw OR 'loss of response':ti,ab,kw OR 'loss of efficacy':ti,ab,kw OR 'inadequate response':ti,ab,kw OR 'insufficient response':ti,ab,kw OR 'suboptimal response':ti,ab,kw OR nonresponder*:ti,ab,kw OR refractory:ti,ab,kw OR recalcitrant:ti,ab,kw OR 'difficult to treat':ti,ab,kw OR switch*:ti,ab,kw OR 'treatment switch':ti,ab,kw OR 'therapy switch':ti,ab,kw OR escalation:ti,ab,kw OR 'dose escalation':ti,ab,kw OR 'dose optimization':ti,ab,kw OR 'dose adjustment':ti,ab,kw OR 'rescue therapy':ti,ab,kw OR 'salvage therapy':ti,ab,kw).ti,ab,kw.

#4, ('systematic review'/exp OR 'meta analysis'/exp OR (systematic NEAR/1 review):ti,ab,kw OR (meta NEAR/1 analys*):ti,ab,kw OR metaanalys*:ti,ab,kw).ti,ab,kw.

#5, #1 AND #2 AND #3 AND #4 282

Database: Cochrane Database of Systematic Reviews (CDSR) – Database of Abstracts of Reviews of Effects (DARE)

Platform: PubMed

Search date: 02/25

Search strategy:

#1, (‘plaque psoriasis’ or ‘psoriasis vulgaris’ or ‘chronic plaque psoriasis’ or ‘plaque-type #1, (‘plaque psoriasis’ OR ‘psoriasis vulgaris’ OR ‘chronic plaque psoriasis’ OR ‘plaque-type psoriasis’)

#2, (adalimumab OR apremilast OR bimekizumab OR brodalumab OR ‘certolizumab pegol’ OR deucravacitinib OR etanercept OR golimumab OR guselkumab OR infliximab OR ixekizumab OR risankizumab OR secukinumab OR tildrakizumab OR ustekinumab OR biologic* OR biological* OR biosimilar* OR ‘TNF inhibitor*’ OR ‘tumor necrosis factor inhibitor*’ OR ‘interleukin 17 inhibitor*’ OR ‘IL-17 inhibitor*’ OR ‘interleukin 23 inhibitor*’ OR ‘IL-23 inhibitor*’ OR ‘interleukin 12 23 inhibitor*’ OR ‘IL-12/23 inhibitor*’)

#3, (‘treatment failure’ OR ‘therapeutic failure’ OR ‘primary failure’ OR ‘primary nonresponse’ OR ‘primary non-response’ OR ‘secondary failure’ OR ‘secondary nonresponse’ OR ‘secondary non-response’ OR ‘loss of response’ OR ‘loss of efficacy’ OR ‘inadequate response’ OR ‘insufficient response’ OR ‘suboptimal response’ OR nonresponder* OR refractory OR recalcitrant OR ‘difficult to treat’ OR switch* OR ‘treatment switch’ OR ‘therapy switch’ OR cycling OR ‘biologic cycling’ OR escalation OR ‘dose escalation’ OR ‘dose optimization’ OR ‘dose adjustment’ OR ‘combination therapy’ OR ‘combination treatment’ OR ‘add-on’ OR ‘adjunctive therapy’ OR ‘rescue therapy’ OR ‘salvage therapy’)

#4 (#1 AND #2 AND #3) 111

## HIGH-IMPACT AREAS AND SPECIAL FORMS

Evidence search strategies

Database: Medline

Platform: Ovid

Search date: 02/25

Search strategy:

#1, ('psoriasis' or 'psoriasis vulgaris' or 'plaque psoriasis' or 'psoriatic skin' or 'psoriasiform dermatitis' or 'psoriasiform dermatosis' or 'guttate psoriasis' or 'psoriasis guttata' or 'pustular psoriasis' or 'psoriasis pustulosa' or 'generalized pustular psoriasis' or 'GPP' or 'palmoplantar pustulosis' or 'pustulosis palmoplantaris' or 'palmoplantar psoriasis' or 'palmar plantar psoriasis' or 'erythrodermic psoriasis' or 'psoriatic erythroderma' or 'scalp psoriasis' or 'psoriasis of the scalp' or 'nail psoriasis' or 'psoriatic nail' or 'genital psoriasis' or 'inverse psoriasis' or 'flexural psoriasis').ti,ab,kw. 76,325

#2, ('treatment' or 'therapy' or 'therapeutics' or 'management' or 'drug therapy' or 'topical therapy' or 'topical treatment' or 'systemic therapy' or 'systemic treatment' or 'biologic therapy' or 'biological therapy' or 'phototherapy' or 'PUVA' or 'narrow band UVB' or 'NB-UVB' or 'ultraviolet b' or 'ultraviolet therapy' or 'laser therapy' or 'excimer laser').ti,ab,kw. 16,901,541

#3, ('adalimumab' or 'etanercept' or 'infliximab' or 'certolizumab pegol' or 'golimumab' or 'ustekinumab' or 'secukinumab' or 'ixekizumab' or 'brodalumab' or 'bimekizumab' or 'guselkumab' or 'risankizumab' or 'tildrakizumab' or 'spesolimab' or 'apremilast' or 'deucravacitinib' or 'tofacitinib' or 'baricitinib' or 'upadacitinib' or 'methotrexate' or 'cyclosporine' or 'ciclosporin' or 'acitretin' or 'retinoid' or 'fumaric acid esters' or 'dimethyl fumarate' or 'calcipotriol' or 'calcipotriene' or 'vitamin d analogue' or 'corticosteroid' or 'topical corticosteroid' or 'tacrolimus' or 'pimecrolimus' or 'coal tar' or 'anthralin' or 'dithranol').ti,ab,kw. 614,512

#4, 2 or 3	11,538,373

#5, 1 and 4. 53,481

#6, #5 AND Filters	165

Database: Embase

Platform: Embase

Search date: 02/25

Search date range: 10 years

Search strategy:

#1,’'psoriasis'/mj OR 'psoriasis vulgaris' OR 'plaque psoriasis' OR 'psoriatic skin' OR 'psoriasiform dermatitis' OR 'psoriasiform dermatosis' OR 'guttate psoriasis'/mj OR 'psoriasis guttata' OR 'pustular psoriasis'/mj OR 'generalized pustular psoriasis'/mj OR 'palmoplantar pustulosis'/mj OR 'pustulosis palmoplantaris'/mj OR 'palmoplantar psoriasis'/mj OR 'palmar plantar psoriasis' OR 'erythrodermic psoriasis'/mj OR 'psoriatic erythroderma' OR 'scalp psoriasis'/mj OR 'psoriasis of the scalp' OR 'nail psoriasis'/mj OR 'psoriatic nail' OR 'genital psoriasis'/mj OR 'inverse psoriasis'/mj OR 'flexural psoriasis'‘,[n]

#2,’'therapy'/mj OR 'treatment'/mj OR 'drug therapy'/mj OR 'topical therapy'/mj OR 'systemic therapy'/mj OR 'biological therapy'/mj OR 'phototherapy'/mj OR 'ultraviolet therapy'/mj OR 'puva'/mj OR 'narrow band uvb' OR 'NB-UVB' OR 'excimer laser'/mj’, [n]

#3,’'adalimumab'/mj OR 'adalimumab' OR 'etanercept'/mj OR 'etanercept' OR 'infliximab'/mj OR 'infliximab' OR 'certolizumab pegol'/mj OR 'certolizumab pegol' OR 'golimumab'/mj OR 'golimumab' OR 'ustekinumab'/mj OR 'ustekinumab' OR 'secukinumab'/mj OR 'secukinumab' OR 'ixekizumab'/mj OR 'ixekizumab' OR 'brodalumab'/mj OR 'brodalumab' OR 'bimekizumab'/mj OR 'bimekizumab' OR 'guselkumab'/mj OR 'guselkumab' OR 'risankizumab'/mj OR 'risankizumab' OR 'tildrakizumab'/mj OR 'tildrakizumab' OR 'spesolimab'/mj OR 'spesolimab' OR 'apremilast'/mj OR 'apremilast' OR 'deucravacitinib'/mj OR 'deucravacitinib' OR 'methotrexate'/mj OR 'methotrexate' OR 'cyclosporin'/mj OR 'ciclosporin' OR 'acitretin'/mj OR 'acitretin' OR 'dimethyl fumarate'/mj OR 'fumaric acid ester'/mj OR 'calcipotriol'/mj OR 'calcipotriol' OR 'corticosteroid'/mj OR 'topical corticosteroid' OR 'tacrolimus'/mj OR 'tacrolimus' OR 'pimecrolimus'/mj OR 'pimecrolimus' OR 'coal tar'/mj OR 'anthralin'/mj OR 'dithranol'‘,[n]

#4,’#2 OR #3’,[n]

#5,’#1 AND #4’,[n]

#6,’#5 AND [2016-2026]/py’,[n]. 330

Database: Cochrane Database of Systematic Reviews – Database of Abstracts of Reviews of Effects

Platform: PubMed

Search date: 02/25

Search strategy:

#1, (‘Psoriasis’[Mesh] OR ‘Psoriasis, Pustular’[Mesh] OR ‘Psoriasis, Erythrodermic’[Mesh] OR (‘psoriasis’[Title/Abstract] OR ‘plaque psoriasis’[Title/Abstract] OR ‘psoriasis vulgaris’[Title/Abstract] OR ‘guttate psoriasis’[Title/Abstract] OR ‘psoriasis guttata’[Title/Abstract] OR ‘pustular psoriasis’[Title/Abstract] OR ‘generalized pustular psoriasis’[Title/Abstract] OR ‘palmoplantar pustulosis’[Title/Abstract] OR ‘palmoplantar psoriasis’[Title/Abstract] OR ‘erythrodermic psoriasis’[Title/Abstract] OR ‘scalp psoriasis’[Title/Abstract] OR ‘nail psoriasis’[Title/Abstract] OR ‘genital psoriasis’[Title/Abstract] OR ‘inverse psoriasis’[Title/Abstract] OR ‘flexural psoriasis’[Title/Abstract]))

#2, (‘Therapeutics’[Mesh] OR ‘Drug Therapy’[Mesh] OR ‘Phototherapy’[Mesh] OR ‘Ultraviolet Therapy’[Mesh] OR (‘treatment’[Title/Abstract] OR ‘therapy’[Title/Abstract] OR ‘management’[Title/Abstract] OR ‘topical therapy’[Title/Abstract] OR ‘systemic therapy’[Title/Abstract] OR ‘biologic therapy’[Title/Abstract] OR ‘phototherapy’[Title/Abstract] OR ‘PUVA’[Title/Abstract] OR ‘narrow band UVB’[Title/Abstract] OR ‘excimer laser’[Title/Abstract]))

#3, (‘adalimumab’[MeSH Terms] OR ‘Etanercept’[MeSH Terms] OR ‘Infliximab’[MeSH Terms] OR ‘Certolizumab Pegol’[MeSH Terms] OR ‘Ustekinumab’[MeSH Terms] OR (‘adalimumab’[Title/Abstract] OR ‘etanercept’[Title/Abstract] OR ‘infliximab’[Title/Abstract] OR ‘certolizumab pegol’[Title/Abstract] OR ‘golimumab’[Title/Abstract] OR ‘ustekinumab’[Title/Abstract] OR ‘secukinumab’[Title/Abstract] OR ‘ixekizumab’[Title/Abstract] OR ‘brodalumab’[Title/Abstract] OR ‘bimekizumab’[Title/Abstract] OR ‘guselkumab’[Title/Abstract] OR ‘risankizumab’[Title/Abstract] OR ‘tildrakizumab’[Title/Abstract] OR ‘spesolimab’[Title/Abstract] OR ‘apremilast’[Title/Abstract] OR ‘deucravacitinib’[Title/Abstract] OR ‘methotrexate’[Title/Abstract] OR ‘cyclosporine’[Title/Abstract] OR ‘ciclosporin’[Title/Abstract] OR ‘acitretin’[Title/Abstract] OR ‘dimethyl fumarate’[Title/Abstract] OR ‘calcipotriol’[Title/Abstract] OR ‘calcipotriene’[Title/Abstract] OR ‘tacrolimus’[Title/Abstract] OR ‘pimecrolimus’[Title/Abstract]))

#4, #2 OR #3

#5, #1 AND #4

#6, #5 Filters: in the last 10 years, Systematic Review. 192

## SPECIAL SITUATIONS

Evidence search strategies

Database: Medline

Platform: PubMed

Search date: 02/25

Search date range: 10 years

Search strategy:

#1, ‘Psoriasis’[MeSH Terms] OR ‘plaque psoriasis’[Title/Abstract] OR ‘psoriasis vulgaris’[Title/Abstract] OR ‘chronic plaque psoriasis’[Title/Abstract] OR ‘plaque-type psoriasis’[Title/Abstract] 53,271

#2, ‘Therapeutics’[MeSH Terms] OR ‘Drug Therapy’[MeSH Terms] OR ‘Phototherapy’[MeSH Terms] OR ‘Ultraviolet Therapy’[MeSH Terms] OR ‘Biological Products’[MeSH Terms] OR ‘Antibodies, Monoclonal’[MeSH Terms] OR ‘Antirheumatic Agents’[MeSH Terms] OR (treatment[Title/Abstract] OR therapy[Title/Abstract] OR management[Title/Abstract] OR ‘drug therapy’[Title/Abstract] OR ‘topical therapy’[Title/Abstract] OR ‘systemic therapy’[Title/Abstract] OR ‘biologic therapy’[Title/Abstract] OR ‘biological therapy’[Title/Abstract] OR phototherapy[Title/Abstract] OR PUVA[Title/Abstract] OR ‘narrow band UVB’[Title/Abstract] OR NB-UVB[Title/Abstract] OR ‘excimer laser’[Title/Abstract] OR methotrexate[Title/Abstract] OR cyclosporine[Title/Abstract] OR ciclosporin[Title/Abstract] OR acitretin[Title/Abstract] OR apremilast[Title/Abstract] OR deucravacitinib[Title/Abstract] OR adalimumab[Title/Abstract] OR etanercept[Title/Abstract] OR infliximab[Title/Abstract] OR ‘certolizumab pegol’[Title/Abstract] OR golimumab[Title/Abstract] OR ustekinumab[Title/Abstract] OR secukinumab[Title/Abstract] OR ixekizumab[Title/Abstract] OR brodalumab[Title/Abstract] OR bimekizumab[Title/Abstract] OR guselkumab[Title/Abstract] OR risankizumab[Title/Abstract] OR tildrakizumab[Title/Abstract] OR spesolimab[Title/Abstract]

#3, (‘Child’[MeSH Terms] OR ‘Infant’[MeSH Terms] OR ‘Adolescent’[MeSH Terms] OR (child*[Title/Abstract] OR pediatric*[Title/Abstract] OR paediatric*[Title/Abstract] OR infant*[Title/Abstract] OR adolescen*[Title/Abstract] OR teen*[Title/Abstract] OR youth*[Title/Abstract] OR neonat*[Title/Abstract]))

#4, (‘Pregnancy’[MeSH Terms] OR ‘Lactation’[MeSH Terms] OR ‘Breast Feeding’[MeSH Terms] OR (pregnan*[Title/Abstract] OR gestation*[Title/Abstract] OR prenatal[Title/Abstract] OR antenatal[Title/Abstract] OR perinatal[Title/Abstract] OR postnatal[Title/Abstract] OR postpartum[Title/Abstract] OR lactat*[Title/Abstract] OR breastfeeding[Title/Abstract] OR ‘breast feeding’[Title/Abstract] OR ‘nursing mother*’[Title/Abstract]))

#5, (‘Neoplasms’[MeSH Terms] OR (cancer*[Title/Abstract] OR neoplasm*[Title/Abstract] OR malignan*[Title/Abstract] OR carcinoma*[Title/Abstract] OR lymphoma*[Title/Abstract] OR leukemia*[Title/Abstract] OR tumour*[Title/Abstract] OR tumor*[Title/Abstract]))

#6, (‘Hepatitis’[MeSH Terms] OR ‘Hepatitis B’[MeSH Terms] OR ‘Hepatitis C’[MeSH Terms] OR ‘Hepatitis, Viral, Human’[MeSH Terms] OR ‘Liver Diseases’[MeSH Terms] OR hepatitis[Title/Abstract] OR ‘hepatitis b’[Title/Abstract] OR HBV[Title/Abstract] OR ‘hepatitis c’[Title/Abstract] OR HCV[Title/Abstract] OR ‘viral hepatitis’[Title/Abstract] OR ‘chronic hepatitis’[Title/Abstract] OR cirrhosis[Title/Abstract] OR ‘liver disease’[Title/Abstract])

#7, (‘HIV Infections’[MeSH Terms] OR ‘HIV’[MeSH Terms] OR ‘Acquired Immunodeficiency Syndrome’[MeSH Terms] OR (HIV[Title/Abstract] OR ‘human immunodeficiency virus’[Title/Abstract] OR AIDS[Title/Abstract] OR ‘acquired immunodeficiency syndrome’[Title/Abstract])

#8, (‘Tuberculosis’[MeSH Terms] OR ‘Mycobacterium tuberculosis’[MeSH Terms] OR ‘Latent Tuberculosis’[MeSH Terms] OR tuberculosis[Title/Abstract] OR TB[Title/Abstract] OR ‘mycobacterium tuberculosis’[Title/Abstract] OR ‘latent tuberculosis’[Title/Abstract] OR LTBI[Title/Abstract])

#9, #3 OR #4 OR #5 OR #6 OR #7 OR #8. 10,819,460

#10, #1 AND #2 AND #9. 1,619,817

#11, #10 Filters: in the last 10 years. 513,664

#12, #11 Filters 166

Database: Embase

Platform: Embase

Search date: 02/25

Search date range: 10 years

Search strategy:

#1, ‘'psoriasis'/mj AND ('psoriasis vulgaris'/mj OR 'plaque psoriasis':ti,ab,kw OR 'psoriasis vulgaris':ti,ab,kw OR 'chronic plaque psoriasis':ti,ab,kw OR 'plaque-type psoriasis':ti,ab,kw)’,[n]

#2, ‘'therapy'/mj OR 'treatment'/mj OR 'drug therapy'/mj OR 'topical therapy'/mj OR 'systemic therapy'/mj OR 'biological therapy'/mj OR 'phototherapy'/mj OR 'ultraviolet therapy'/mj OR 'puva'/mj OR 'methotrexate'/mj OR 'cyclosporin'/mj OR 'acitretin'/mj OR 'apremilast'/mj OR 'deucravacitinib'/mj OR 'adalimumab'/mj OR 'etanercept'/mj OR 'infliximab'/mj OR 'certolizumab pegol'/mj OR 'golimumab'/mj OR 'ustekinumab'/mj OR 'secukinumab'/mj OR 'ixekizumab'/mj OR 'brodalumab'/mj OR 'bimekizumab'/mj OR 'guselkumab'/mj OR 'risankizumab'/mj OR 'tildrakizumab'/mj OR 'spesolimab'/mj OR treatment:ti,ab,kw OR therapy:ti,ab,kw OR management:ti,ab,kw’,[n]

#3, ‘'child'/mj OR 'infant'/mj OR 'adolescent'/mj OR child*:ti,ab,kw OR pediatric*:ti,ab,kw OR paediatric*:ti,ab,kw OR infant*:ti,ab,kw OR adolescen*:ti,ab,kw OR teen*:ti,ab,kw’,[n]

#4, ‘'pregnancy'/mj OR 'lactation'/mj OR 'breast feeding'/mj OR pregnan*:ti,ab,kw OR gestation*:ti,ab,kw OR postpartum:ti,ab,kw OR lactat*:ti,ab,kw OR breastfeeding:ti,ab,kw’,[n]

#5, ‘'neoplasm'/mj OR 'cancer'/mj OR cancer*:ti,ab,kw OR neoplasm*:ti,ab,kw OR malignan*:ti,ab,kw OR carcinoma*:ti,ab,kw OR lymphoma*:ti,ab,kw OR leukemia*:ti,ab,kw’,[n]

#6, ‘'hepatitis'/mj OR 'hepatitis b'/mj OR 'hepatitis c'/mj OR 'liver disease'/mj OR hepatitis:ti,ab,kw OR HBV:ti,ab,kw OR HCV:ti,ab,kw OR cirrhosis:ti,ab,kw’,[n]

#7, ‘'human immunodeficiency virus infection'/mj OR 'acquired immune deficiency syndrome'/mj OR HIV:ti,ab,kw OR AIDS:ti,ab,kw’,[n]

#8, ‘'tuberculosis'/mj OR 'latent tuberculosis'/mj OR tuberculosis:ti,ab,kw OR TB:ti,ab,kw OR LTBI:ti,ab,kw’,[n]

#9, ‘#3 OR #4 OR #5 OR #6 OR #7 OR #8’,[n]

#10, ‘#1 AND #2 AND #9’,[n]

#11, ‘#10 AND [2016-2026]/py’,[n]. 292

Database: Cochrane Database of Systematic Reviews – Database of Abstracts of Reviews of Effects

Platform: PubMed

Search date: 02/25

Search strategy:

#1, (‘Psoriasis’[MeSH Terms] AND (‘Psoriasis Vulgaris’[MeSH Terms] OR ‘plaque psoriasis’[Title/Abstract] OR ‘psoriasis vulgaris’[Title/Abstract] OR ‘chronic plaque psoriasis’[Title/Abstract] OR ‘plaque-type psoriasis’[Title/Abstract]))

#2, (‘Therapeutics’[MeSH Terms] OR ‘Drug Therapy’[MeSH Terms] OR ‘Phototherapy’[MeSH Terms] OR ‘Ultraviolet Therapy’[MeSH Terms] OR ‘Biological Products’[MeSH Terms] OR ‘Antibodies, Monoclonal’[MeSH Terms] OR (treatment[Title/Abstract] OR therapy[Title/Abstract] OR management[Title/Abstract] OR methotrexate[Title/Abstract] OR cyclosporine[Title/Abstract] OR acitretin[Title/Abstract] OR apremilast[Title/Abstract] OR deucravacitinib[Title/Abstract] OR adalimumab[Title/Abstract] OR etanercept[Title/Abstract] OR infliximab[Title/Abstract] OR ‘certolizumab pegol’[Title/Abstract] OR ustekinumab[Title/Abstract] OR secukinumab[Title/Abstract] OR ixekizumab[Title/Abstract] OR brodalumab[Title/Abstract] OR bimekizumab[Title/Abstract] OR guselkumab[Title/Abstract] OR risankizumab[Title/Abstract] OR tildrakizumab[Title/Abstract] OR spesolimab[Title/Abstract]))

#3, (child*[Title/Abstract] OR pediatric*[Title/Abstract] OR paediatric*[Title/Abstract] OR infant*[Title/Abstract] OR adolescen*[Title/Abstract] OR pregnan*[Title/Abstract] OR lactat*[Title/Abstract] OR breastfeeding[Title/Abstract] OR cancer*[Title/Abstract] OR neoplasm*[Title/Abstract] OR hepatitis[Title/Abstract] OR HBV[Title/Abstract] OR HCV[Title/Abstract] OR HIV[Title/Abstract] OR AIDS[Title/Abstract] OR tuberculosis[Title/Abstract] OR TB[Title/Abstract] OR LTBI[Title/Abstract])

#4, #1 AND #2 AND #3

#5, #4 Filters: in the last 10 years, Systematic Review 125

## BIOSIMILARS

Evidence search strategies

Database: Medline

Platform: PubMed

Search date: 02/25

Search strategy:

#1, ‘Psoriasis’[MeSH Terms] OR ‘plaque psoriasis’ OR ‘psoriasis vulgaris’ OR ‘chronic plaque psoriasis’OR ‘plaque-type psoriasis’ OR 'psoriasiform dermatitis' or 'psoriasiform dermatosis' or 'psoriasiform lesion' or 'psoriasiform rash' or 'psoriasiform skin rash' or 'psoriasis' or 'psoriatic epidermis' or 'psoriatic skin'

#2, ‘Biological Products’[MeSH Terms] OR ‘Antibodies, Monoclonal’[MeSH Terms] OR ‘Tumor Necrosis Factor Inhibitors’[MeSH Terms] OR biologic*[Title/Abstract] OR biological*[Title/Abstract] OR ‘biologic therapy’[Title/Abstract] OR ‘biological therapy’[Title/Abstract] OR ‘TNF inhibitor*’ OR ‘interleukin 17 inhibitor*’ OR ‘interleukin 23 inhibitor*’ OR ‘interleukin 12 23 inhibitor*’ OR 'adalimumab' or 'apremilast' or 'bimekizumab' or 'brodalumab' or 'certolizumab pegol' or 'pegylated tumor necrosis factor alpha antibody fab fragment' or 'pegylated tumour necrosis factor alpha antibody fab fragment' or 'deucravacitinib' or 'etanercept' or 'recombinant tumor necrosis factor receptor fc fusion protein' or 'recombinant tumour necrosis factor receptor fc fusion protein' or 'tumor necrosis factor receptor fc fusion protein' or 'tumour necrosis factor receptor fc fusion protein' or 'golimumab' or 'guselkumab' or 'infliximab' or 'ixekizumab' or 'risankizumab' or 'secukinumab' or 'spesolimab' or 'ustekinumab' or rituximab OR CT-P13 OR SB2 OR SB4 OR SB5 OR ABP 501 OR ABP501 OR GP2017 OR PF-06438179 OR PF06438179 OR MSB11022 OR FKB327 OR BI 695501 OR 'HD203' OR 'AVT02' OR 'adalimumab biosimilar' OR 'etanercept biosimilar' OR 'infliximab biosimilar' OR 'ustekinumab biosimilar'

#3, ‘Biosimilar Pharmaceuticals’[MeSH Terms] OR biosimilar* OR biosimilarity OR ‘biosimilar product* OR ‘biosimilar agent*’ OR ‘follow-on biologic*’ OR ‘follow on biologic*’ OR ‘subsequent entry biologic*’ OR ‘similar biotherapeutic product*’ OR ‘similar biotherapeutic*’ OR SBP

#4, #2 AND #3

#5, #1 AND #4

#6, #4 Filters: in the last 10 years. 117

Database: Embase

Platform: Embase

Search date: 02/25

Search strategy:

#1, ‘'psoriasis'/mj AND ('psoriasis vulgaris'/mj OR 'plaque psoriasis':ti,ab,kw OR 'psoriasis vulgaris':ti,ab,kw OR 'chronic plaque psoriasis':ti,ab,kw OR 'plaque-type psoriasis':ti,ab,kw)’,[n]

#2, ‘'biological therapy'/mj OR 'biological product'/mj OR 'monoclonal antibody'/mj OR 'tumor necrosis factor inhibitor'/mj OR biologic*:ti,ab,kw OR biological*:ti,ab,kw OR 'TNF inhibitor*':ti,ab,kw OR 'interleukin 17 inhibitor*':ti,ab,kw OR 'interleukin 23 inhibitor*':ti,ab,kw OR 'interleukin 12 23 inhibitor*':ti,ab,kw’,[n]

#3, ‘'biosimilar'/mj OR biosimilar*:ti,ab,kw OR biosimilarity:ti,ab,kw OR 'follow on biologic*':ti,ab,kw OR 'subsequent entry biologic*':ti,ab,kw OR 'similar biotherapeutic*':ti,ab,kw OR SBP:ti,ab,kw’,[n]

#4, ‘'adalimumab'/mj OR 'etanercept'/mj OR 'infliximab'/mj OR 'ustekinumab'/mj OR adalimumab:ti,ab,kw OR etanercept:ti,ab,kw OR infliximab:ti,ab,kw OR ustekinumab:ti,ab,kw OR 'CT-P13':ti,ab,kw OR SB2:ti,ab,kw OR SB4:ti,ab,kw OR SB5:ti,ab,kw OR 'ABP 501':ti,ab,kw OR ABP501:ti,ab,kw OR GP2017:ti,ab,kw OR 'PF-06438179':ti,ab,kw OR PF06438179:ti,ab,kw OR MSB11022:ti,ab,kw OR FKB327:ti,ab,kw OR 'BI 695501':ti,ab,kw OR BI695501:ti,ab,kw OR HD203:ti,ab,kw OR AVT02:ti,ab,kw’,[n]

#5, ‘#2 AND (#3 OR #4)’,[n]

#6, ‘#1 AND #5’,[n]

#7, ‘#6 AND [2016-2026]/py’,[n] 162

Database: Cochrane Database of Systematic Reviews – Database of Abstracts of Reviews of Effects

Platform: PubMed

Search date: 02/25

Search strategy:

#1, (‘Psoriasis’[MeSH Terms] AND (‘Psoriasis Vulgaris’[MeSH Terms] OR ‘plaque psoriasis’[Title/Abstract] OR ‘psoriasis vulgaris’[Title/Abstract] OR ‘chronic plaque psoriasis’[Title/Abstract] OR ‘plaque-type psoriasis’[Title/Abstract]))

#2, (‘Biological Products’[MeSH Terms] OR ‘Antibodies, Monoclonal’[MeSH Terms] OR ‘Tumor Necrosis Factor Inhibitors’[MeSH Terms] OR (biologic*[Title/Abstract] OR biological*[Title/Abstract] OR ‘TNF inhibitor*’[Title/Abstract] OR ‘interleukin 17 inhibitor*’[Title/Abstract] OR ‘interleukin 23 inhibitor*’[Title/Abstract] OR ‘interleukin 12 23 inhibitor*’[Title/Abstract]))

#3, (‘Biosimilar Pharmaceuticals’[MeSH Terms] OR biosimilar*[Title/Abstract] OR biosimilarity[Title/Abstract] OR ‘follow-on biologic*’[Title/Abstract] OR ‘follow on biologic*’[Title/Abstract] OR ‘subsequent entry biologic*’[Title/Abstract] OR ‘similar biotherapeutic*’[Title/Abstract] OR SBP[Title/Abstract] OR ‘adalimumab biosimilar’[Title/Abstract] OR ‘etanercept biosimilar’[Title/Abstract] OR ‘infliximab biosimilar’[Title/Abstract] OR ‘ustekinumab biosimilar’[Title/Abstract] OR CT-P13[Title/Abstract] OR SB2[Title/Abstract] OR SB4[Title/Abstract] OR SB5[Title/Abstract] OR ABP501[Title/Abstract] OR ‘ABP 501’[Title/Abstract] OR GP2017[Title/Abstract] OR PF-06438179[Title/Abstract] OR PF06438179[Title/Abstract] OR MSB11022[Title/Abstract] OR FKB327[Title/Abstract] OR BI695501[Title/Abstract] OR ‘BI 695501’[Title/Abstract] OR HD203[Title/Abstract] OR AVT02[Title/Abstract])

#4, #1 AND #2 AND #3

#5, #4 Filters: in the last 10 years, Systematic Review 104

## NON-PHARMACOLOGICAL MANAGEMENT

Evidence search strategies

Database: Medline

Platform: PubMed

Search date: 02/25

Search strategy:

#1, ‘Psoriasis’[MeSH Terms] OR ‘plaque psoriasis’ OR ‘psoriasis vulgaris’ OR ‘chronic plaque psoriasis’OR ‘plaque-type psoriasis’ OR 'psoriasiform dermatitis' or 'psoriasiform dermatosis' or 'psoriasiform lesion' or 'psoriasiform rash' or 'psoriasiform skin rash' or 'psoriasis' or 'psoriatic epidermis' or 'psoriatic skin'

#2, "Life Style"[MeSH Terms] OR "Diet"[MeSH Terms] OR "Diet Therapy"[MeSH Terms] OR "Nutrition Therapy"[MeSH Terms] OR "Weight Reduction Programs"[MeSH Terms] OR "Weight Loss"[MeSH Terms] OR "Diet, Reducing"[MeSH Terms] OR "Diet, Anti-Inflammatory" OR "Dietary Supplements"[MeSH Terms] OR "Fatty Acids, Omega-3"[MeSH Terms] OR "Fish Oils"[MeSH Terms] OR "Vitamins"[MeSH Terms] OR "Vitamin D"[MeSH Terms] OR "Probiotics"[MeSH Terms] OR "Prebiotics"[MeSH Terms] OR "Dietary Fiber"[MeSH Terms] OR "Fasting"[MeSH Terms] OR nutrition*[Title/Abstract] OR "nutritional intervention*"[Title/Abstract]

#3, "Psychotherapy"[MeSH Terms] OR "Cognitive Behavioral Therapy"[MeSH Terms] OR "Mindfulness"[MeSH Terms] OR "Meditation"[MeSH Terms] OR "Stress, Psychological"[MeSH Terms] OR "Stress Management"OR "Relaxation Therapy"[MeSH Terms] OR "Behavior Therapy"[MeSH Terms] OR "Support Groups" OR "Patient Education as Topic"[MeSH Terms] OR psycholog*[Title/Abstract] OR psychotherapy[Title/Abstract] OR CBT[Title/Abstract] OR "support group*"[Title/Abstract] OR "supportive therapy"[Title/Abstract] OR psychoeducation[Title/Abstract] OR "patient education"[Title/Abstract]

#4, (#2 OR #3)

#5, #1 AND #4. 112

Database: Embase

Platform: Embase

Search date: 02/25

Search strategy:

#1, "'psoriasis'/mj OR psoriasis:ti,ab,kw OR 'plaque psoriasis':ti,ab,kw OR 'psoriasis vulgaris':ti,ab,kw",[n]

#2, "'lifestyle'/mj OR 'diet'/mj OR 'diet therapy'/mj OR 'nutrition'/mj OR 'nutrition therapy'/mj OR 'weight reduction'/mj OR 'weight loss'/mj OR 'mediterranean diet'/mj OR 'dietary supplement'/mj OR 'omega 3 fatty acid'/mj OR 'fish oil'/mj OR 'vitamin d'/mj OR 'probiotic'/mj OR 'prebiotic agent'/mj OR 'gluten free diet'/mj OR 'fasting'/mj OR diet:ti,ab,kw OR nutrition*:ti,ab,kw OR 'diet therapy':ti,ab,kw OR 'nutrition therapy':ti,ab,kw OR 'mediterranean diet':ti,ab,kw OR 'anti-inflammatory diet':ti,ab,kw OR 'gluten free':ti,ab,kw OR 'gluten-free':ti,ab,kw OR fasting:ti,ab,kw OR 'intermittent fasting':ti,ab,kw OR 'weight loss':ti,ab,kw OR 'weight reduction':ti,ab,kw OR obesity:ti,ab,kw OR omega-3:ti,ab,kw OR 'fish oil':ti,ab,kw OR probiotic*:ti,ab,kw OR prebiotic*:ti,ab,kw OR synbiotic*:ti,ab,kw OR 'vitamin d':ti,ab,kw OR supplement*:ti,ab,kw",[n]

#3, "'psychotherapy'/mj OR 'cognitive therapy'/mj OR 'cognitive behavioral therapy'/mj OR 'mindfulness'/mj OR 'meditation'/mj OR 'stress'/mj OR 'stress management'/mj OR 'relaxation'/mj OR 'behavior therapy'/mj OR 'support group'/mj OR 'patient education'/mj OR psycholog*:ti,ab,kw OR psychotherapy:ti,ab,kw OR 'cognitive behavioral therapy':ti,ab,kw OR CBT:ti,ab,kw OR mindfulness:ti,ab,kw OR meditation:ti,ab,kw OR 'stress management':ti,ab,kw OR relaxation:ti,ab,kw OR counselling:ti,ab,kw OR counseling:ti,ab,kw OR 'support group*':ti,ab,kw OR psychoeducation:ti,ab,kw OR 'patient education':ti,ab,kw",[n]

#4, "#2 OR #3",[n]

#5, "#1 AND #4",[n]

#6, "#5 AND [2016-2026]/py",[n] 153

Database: Cochrane Database of Systematic Reviews – Database of Abstracts of Reviews of Effects

Platform: PubMed

Search date: 02/25

Search strategy:

#1, ("Psoriasis"[MeSH Terms] OR psoriasis[Title/Abstract] OR "plaque psoriasis"[Title/Abstract] OR "psoriasis vulgaris"[Title/Abstract])

#2, ("Life Style"[MeSH Terms] OR "Diet"[MeSH Terms] OR "Diet Therapy"[MeSH Terms] OR "Nutrition Therapy"[MeSH Terms] OR "Weight Loss"[MeSH Terms] OR "Mediterranean Diet"[MeSH Terms] OR "Dietary Supplements"[MeSH Terms] OR "Fatty Acids, Omega-3"[MeSH Terms] OR "Fish Oils"[MeSH Terms] OR "Vitamin D"[MeSH Terms] OR "Probiotics"[MeSH Terms] OR "Gluten-Free Diet"[MeSH Terms] OR "Fasting"[MeSH Terms] OR diet[Title/Abstract] OR nutrition*[Title/Abstract] OR "weight loss"[Title/Abstract] OR "mediterranean diet"[Title/Abstract] OR omega-3[Title/Abstract] OR "fish oil"[Title/Abstract] OR probiotic*[Title/Abstract] OR "vitamin d"[Title/Abstract] OR supplement*[Title/Abstract] OR fasting[Title/Abstract])

#3, ("Psychotherapy"[MeSH Terms] OR "Cognitive Behavioral Therapy"[MeSH Terms] OR "Mindfulness"[MeSH Terms] OR "Meditation"[MeSH Terms] OR "Stress Management"[MeSH Terms] OR "Relaxation Therapy"[MeSH Terms] OR "Support Groups"[MeSH Terms] OR psychotherapy[Title/Abstract] OR "cognitive behavioral therapy"[Title/Abstract] OR CBT[Title/Abstract] OR mindfulness[Title/Abstract] OR meditation[Title/Abstract] OR "stress management"[Title/Abstract] OR relaxation[Title/Abstract] OR counseling[Title/Abstract] OR counselling[Title/Abstract] OR psychoeducation[Title/Abstract] OR "support group"[Title/Abstract])

#4, #1 AND (#2 OR #3)

#5, #4 Filters: in the last 10 years, Systematic Review. 109

# SYNTHESIS AND EVIDENCE APPRAISAL

## Question 1. In patients with moderate-to-severe plaque psoriasis, what should the index-based treatment target be?

Evidence from 20 studies—mainly post-hoc analyses and observational studies—supports the use of more stringent therapeutic targets in moderate-to-severe psoriasis. Achieving PASI90 or PASI100 is currently considered a key goal, given its association with complete or near-complete remission of disease and a clinically meaningful improvement in quality of life. Newer biologic therapies, such as risankizumab, ixekizumab, and secukinumab, show a higher likelihood of achieving these responses, with greater patient satisfaction and reduced impact of disease on daily life, including itch and pain. Additionally, there is a good correlation between absolute PASI ≤2 and PASI90. In patients who do not reach at least PASI75, studies suggest reassessing the therapeutic strategy.

**Quality of evidence:** Low to moderate

Description of studies involving therapeutic targets in moderate-to-severe psoriasis

*Secondary sources: reviews and consensus statements*

| **Author** | **Year** | **Study design** | **Population** | **Indices** | **Results** |
| --- | --- | --- | --- | --- | --- |
| **Yasmeen et al.** **[1]** | 2022 | Systematic review and network meta-analysis | Patients with moderate-to-severe psoriasis treated with targeted therapies. | PASI75; PASI90; PASI100 | Over 1 year, targeted therapies achieved PASI75, PASI90, and PASI100 with varying success rates by treatment. Newer biologics, including IL-17 and IL-23 inhibitors, showed higher PASI90 and PASI100 rates than anti-TNF agents. Secukinumab and ixekizumab had the highest PASI100 rates at 1 year, with 50–60% of patients achieving complete remission and 70–80% achieving PASI90. |
| **Belinchón Romero et al.** **[2]** | 2021 | Expert consensus | Patients with moderate-to-severe psoriasis | PASI75; PASI90; PASI100; PGA; DLQI | PASI90 is considered a realistic therapeutic target for most patients with moderate-to-severe psoriasis, while PASI100 is the ideal target. PASI75 is viewed as the minimum acceptable response to avoid treatment failure. A change in treatment strategy is suggested if PASI75 is not achieved or if absolute PASI remains ≤3. Quality of life (DLQI) improves meaningfully when PASI90 or higher is achieved and is a key factor in clinical decision-making. |
| **Galluzzo et al.** **[3]** | 2021 | Narrative review | Patients with moderate-to-severe plaque psoriasis | PASI90; DLQI; PSD | Secukinumab has positioned PASI90 as the new standard therapeutic target in moderate-to-severe psoriasis, and achieving PASI90 correlates strongly with DLQI 0/1. Across studies, 75–93% of patients achieved PASI75 and 60–76% achieved PASI90 at 52 weeks. Longer-term outcomes (up to 5 years) were also favorable, with 59% achieving PASI90. Clinically relevant improvements were reported in quality of life and patient-perceived symptoms (pruritus, pain, scaling), assessed using the PSD. |
| **Rusk et al.****[4]** | 2021 | Narrative review | Patients with nail psoriasis | NAPSI; PASI90 | Patients who achieve PASI75 or PASI90 tend to show nail improvement measured by NAPSI. For example, in biologic studies, up to 75% of patients who reach PASI90 show reduced nail psoriasis severity at 24 weeks. Nail response is typically slower than skin response: ~40% show substantial NAPSI improvement before week 16, increasing gradually to 60–70% by week 24 or later. |
| **Belinchón Romero et al.** **[5]** | 2023 | Systematic literature review | Patients with moderate-to-severe psoriasis treated with TNF-α inhibitors | PASI50; PASI75; DLQI; PGA | Primary nonresponse to TNF-α inhibitors is defined as failure to achieve PASI50 after 12–16 weeks of treatment. DLQI and PGA are also discussed as tools to assess overall response and quality-of-life impact. |
| **Gisondi et al.** **[6]** | 2015 | Review article and expert consensus | Patients with moderate-to-severe plaque psoriasis | PASI; DLQI | Patients with moderate-to-severe psoriasis should achieve at least PASI75 to continue systemic or biologic therapy. Newer treatments, including IL-17 and IL-23 inhibitors, are raising response expectations toward PASI90/100. |
| **Puig et al.** **[7]** | 2014 | Narrative review | Patients with moderate-to-severe plaque psoriasis | PASI75; PASI90; DLQI | A 90% PASI improvement is proposed as the new benchmark for therapeutic success in moderate-to-severe psoriasis. PASI improvement correlates strongly with DLQI, particularly among patients achieving PASI90 or better, who show higher rates of DLQI 0–1. Newer therapies—especially IL-17 biologics—have demonstrated high PASI90 rates in clinical trials, with up to 75% of patients reaching PASI90 by 12 weeks. |

*Primary sources: observational studies and post hoc analyses of clinical trials*

| **Author** | **Year** | **Study design** | **Population** | **Indices** | **Results** |
| --- | --- | --- | --- | --- | --- |
| **Boswell et al.** **[8]** | 2024 | Observational, analytic, retrospective cohort | Patients with moderate-to-severe psoriasis and concomitant psoriatic arthritis | PASI; DLQI | At week 12, 42.2% achieved PASI90 and 25.2% achieved PASI75–89. Patients achieving PASI90–100 at week 12 had greater improvement in quality of life (DLQI 0/1) than those achieving PASI75–89. Median time to DLQI 0/1 was 8 weeks for PASI90 responders vs 12 weeks for PASI75–89 responders. At week 52, 74% of PASI90 responders achieved DLQI 0/1 vs 57% of PASI75–89 responders. |
| **Thaçi et al.** **[9]** | 2023 | Post hoc analysis of phase 3 reSURFACE 1 and 2 trials | 443 patients with moderate-to-severe psoriasis | PASI; DLQI | Over 5 years, patients treated with tildrakizumab maintained a PASI <3 response. Among those receiving 100 mg and 200 mg, 76.1% and 81.7% maintained PASI <3 for at least 90% of follow-up. PASI <3 was maintained at all visits through 244 weeks in 58.9% (100 mg) and 70% (200 mg). Patients who achieved PASI <3 reported marked quality-of-life improvement, with DLQI 0/1. |
| **Imafuku et al.** **[10]** | 2022 | Prospective, multicenter case series | 73 patients with moderate-to-severe plaque psoriasis treated with brodalumab | PASI; BSA; PtGA; TSQM-9 | Brodalumab produced a marked PASI reduction from baseline (median 12.1) to 0 at weeks 12 and 48 (p<0.0001). Ninety percent of patients reported effective treatment on PtGA at weeks 12 and 48. TSQM-9 scores improved for overall satisfaction, convenience, and effectiveness (p<0.0001). PASI ≤2 was proposed as a practical target to improve satisfaction, although achieving this threshold alone may not ensure complete long-term satisfaction. |
| **Reich et al.** **[11]** | 2021 | Pooled analysis of 4 phase 3 and 4 trials (ESTEEM 1 and 2, LIBERATE, UNVEIL) | 1062 patients with moderate-to-severe plaque psoriasis (PASI ≥12 and BSA ≥10%) | PASI; DLQI; ScPGA; NAPSI | At 32 weeks, 29.5% achieved PASI ≤2, with higher rates among those with lower baseline severity. Patients in the lowest baseline PASI quartiles (PASI 2.4–13.1) achieved PASI ≤2 in 43.5%, whereas only 18.4% with higher baseline PASI (>20.1) did so. Among those achieving PASI ≤2, 83.6% achieved DLQI ≤5. Shorter disease duration (<5 years) and no prior systemic therapy were associated with higher success rates. |
| **Campanati et al.** **[12]** | 2021 | Post hoc analysis of the SUPREME study | 433 patients with moderate-to-severe plaque psoriasis | PASI; DLQI; BSA; IGA | At weeks 16 and 24, 65% and 70% achieved MDA-1 (PASI90 and DLQI 0/1). At weeks 16 and 24, 76% and 83% achieved MDA-2 (PASI ≤1 or BSA <3%). Age, weight, and BMI influenced MDA attainment; younger and lower-weight patients achieved MDA more often. |
| **Lebwohl, et al.** **[13]** | 2021 | Post hoc analysis of 4 phase III trials | 2101 patients with moderate-to-severe plaque psoriasis | PASI90 | The study assessed duration of clinical remission as time maintaining PASI90 and compared risankizumab with adalimumab and ustekinumab at 1 year. Risankizumab monotherapy yielded the longest duration of PASI90 (mean 245.7 days [67.3%]) vs adalimumab and risankizumab (183.8 days [50.4%]). These findings suggest longer maintenance of clinical remission with risankizumab vs comparator regimens. |
| **Puig et al.** **[14]** | 2020 | Post hoc analysis of a long-term clinical trial (phase IIIB, 52 weeks) | Patients with moderate-to-severe plaque psoriasis (PASI ≥10) | PASI50; PASI75; PASI90; PASI100; DLQI | Patients treated with ixekizumab reached higher skin clearance thresholds (PASI75/90/100) earlier than those treated with ustekinumab. Early response milestones (eg, PASI75 at week 4 and PASI90 at week 12) predicted sustained quality-of-life improvement (DLQI 0/1) at 52 weeks. With early target attainment, up to 87% of ixekizumab-treated patients and 71% of ustekinumab-treated patients achieved DLQI 0/1 at week 52. |
| **Puig et al.** **[15]** | 2019 | Post hoc analysis of phase 3 UNCOVER-2 and UNCOVER-3 trials | 2567 adults with moderate-to-severe plaque psoriasis (PASI ≥12) | PASI; DLQI; Itch Numeric Rating Scale; PtGA | At week 12, 54.6% of ixekizumab-treated patients achieved PASI ≤1 vs 13.4% with etanercept and 0.8% with placebo. Patients achieving PASI ≤2 also reported major quality-of-life gains: 81.8% achieved DLQI (0,1) and 96% achieved PtGA (0,1). |
| **Merola et al.** **[16]** | 2018 | Pooled analysis of three randomized, double-blind, placebo-controlled trials (abstract) | 3866 patients with moderate-to-severe plaque psoriasis (PASI ≥12, BSA ≥10%) | PASI; BSA; sPGA | Correlations between BSA × sPGA and PASI were strong, with 93.8% concordance for PASI75 and 88.2% for PASI90, suggesting BSA × sPGA may substitute for PASI in clinical practice. A 66% change in BSA predicted PASI75, and a 94% change in BSA × sPGA predicted PASI90. These findings support BSA and BSA × sPGA as practical tools producing results comparable to PASI. |
| **Elewski et al.** **[17]** | 2017 | Data analysis from two phase 3 studies (ERASURE and FIXTURE) | Patients with moderate-to-severe plaque psoriasis | PASI; DLQI; IGA | At week 12, 69.4% of PASI90 responders achieved DLQI 0/1 vs 47.1% of those achieving PASI75–89. At week 52, 74% of PASI90 responders achieved DLQI 0/1 vs 56.7% of PASI75–89 responders. Median time to DLQI 0/1 was 8 weeks for PASI90 responders and 12 weeks for PASI75–89 responders. |

**References**

[1]	Yasmeen N, Sawyer LM, Malottki K, et al. Targeted therapies for patients with moderate-to-severe psoriasis: a systematic review and network meta-analysis of PASI response at 1 year. J Dermatol Treat. 2022;33(1):204–218.

[2]	Belinchón Romero I, Dauden E, Ferrándiz Foraster C, et al. Therapeutic goals and treatment response evaluation in moderate to severe psoriasis: an experts opinion document. Ann Med. 2021;53(1):1727–1736.

[3]	Galluzzo M, Talamonti M, Manfreda V, et al. Impact of secukinumab on patient-reported outcomes in moderate to severe plaque psoriasis: a review of clinical studies. Expert Opin Biol Ther. 2021;21(2):191–200.

[4]	Rusk AM, Fleischer AB. In psoriasis treatment, greater improvement in skin severity predicts greater improvement in nail severity. J Dermatol Treat. 2021;32(8):894–897.

[5]	Belinchon Romero I, Mateu Puchades A, Ribera Pibernat M, et al. Criteria used to define tumor necrosis factor-alpha inhibitors failure in patients with moderate-to-severe psoriasis: a systematic literature review. Ann Med. 2023;55(1):1335–1345.

[6]	Gisondi P, Di Mercurio M, Idolazzi L, et al. Concept of Remission in Chronic Plaque Psoriasis. J Rheumatol Suppl. 2015;93(0):57–60.

[7]	Puig L. PASI 90 response: the new standard in therapeutic efficacy for psoriasis. J Eur Acad Dermatol Venereol. 2015;29(4):645–648.

[8]	Boswell ND, Singla S, Gordon KB. Sequencing of Targeted Therapy in Psoriasis: Does it Matter? Am J Clin Dermatol. 2024;25(5):795–810.

[9]	Thaçi D, Piaserico S, Gaarn Du Jardin K, et al. Long‐term stability of PASI <3 response to tildrakizumab: Pooled analyses from reSURFACE 1 and reSURFACE 2 phase 3 trials through 5 years. J Eur Acad Dermatol Venereol [Internet]. 2023 [cited 2024 Oct 4];37(4).

[10]	Imafuku S, Ohata C, Okubo Y, et al. Effectiveness of brodalumab in achieving treatment satisfaction for patients with plaque psoriasis: The ProLOGUE study. J Dermatol Sci. 2022;105(3):176–184.

[11]	Reich K, Mrowietz U, Menter A, et al. Effect of baseline disease severity on achievement of treatment target with apremilast: results from a pooled analysis. J Eur Acad Dermatol Venereol. 2021;35(12):2409–2414.

[12]	Campanati A, Diotallevi F, Radi G, et al. Psoriatic patients treated with secukinumab reach high levels of minimal disease activity: results from the SUPREME study. Eur J Dermatol. 2021;31(5):630–637.

[13]	Lebwohl MG, Soliman AM, Yang H, et al. 25572 Impact of risankizumab on the duration of PASI90 achievement: A post hoc analysis of 4 phase III clinical trials. J Am Acad Dermatol. 2021;85(3):AB62.

[14]	Puig L, Zhu B, Burge R, et al. Early Treatment Targets for Predicting Long-term Dermatology Life Quality Index Response in Patients with Moderate-to-Severe Plaque Psoriasis: A Post-hoc Analysis from a Long-term Clinical Study. J Clin Aesthetic Dermatol. 2020;13(10):18–22.

[15]	Puig L, Dossenbach M, Berggren L, et al. Absolute and Relative Psoriasis Area and Severity Indices (PASI) for Comparison of the Efficacy of Ixekizumab to Etanercept and Placebo in Patients with Moderate-to-severe Plaque Psoriasis: An Integrated Analysis of UNCOVER-2 and UNCOVER-3 Outcomes. Acta Derm Venereol. 2019;99(11):971–977.

[16]	Merola JF, Amato DA, See K, et al. Evaluation of sPGA × BSA as an Outcome Measure and Treatment Target for Clinical Practice. J Invest Dermatol. 2018;138(9):1955–1961.

[17]	Elewski BE, Puig L, Mordin M, et al. Psoriasis patients with psoriasis Area and Severity Index (PASI) 90 response achieve greater health-related quality-of-life improvements than those with PASI 75–89 response: results from two phase 3 studies of secukinumab. J Dermatol Treat. 2017;28(6):492–499.

[18]	Schmitt J, Rosumeck S, Thomaschewski G, et al. Efficacy and safety of systemic treatments for moderate-to-severe psoriasis: meta-analysis of randomized controlled trials. Br J Dermatol. 2014;170(2):274–303.

[19]	Li Z, Pei B, Lei T, et al. Clinical features and effects of cyclosporine in the treatment of psoriasis: a systemic review and meta-analysis. Arch Dermatol Res. 2024;316(10):705.

[20]	Kogan N, Raimondo N, Gusis SE, et al. Latin American clinical practice guidelines on the systemic treatment of psoriasis SOLAPSO–Sociedad Latinoamericana de Psoriasis (Latin American Psoriasis Society). Int J Dermatol. 2019;58:4–28.

[21]	van Huizen AM, Sikkel R, Caron AG, et al. Methotrexate dosing regimen for plaque-type psoriasis: an update of a systematic review. J Dermatol Treat. 2022;33(8):3104–3118.

[22]	Hsieh T-S, Tsai T-F. Combination therapy for psoriasis with methotrexate and other oral disease-modifying antirheumatic drugs: a systematic review. Dermatol Ther. 2023;13(4):891–909.

[23]	Cheng Y, Liu GJ, Zhang M. Narrow‐band ultraviolet B phototherapy versus broad‐band ultraviolet B or psoralen‐ultraviolet A photochemotherapy for psoriasis. Cochrane Database Syst Rev. 2013;(10):CD009481.

[24]	Sbidian E, Chaimani A, Garcia-Doval I, et al. Systemic pharmacological treatments for chronic plaque psoriasis: a network meta-analysis. Cochrane Database Syst Rev. 2022;5(5):CD011535.

[25]	Mahmoud A, Ahmed AAS, Naeem A, et al. Tyrosine Kinase-2 Inhibitor (Deucravacitinib) for Psoriasis: A Systematic Review and Network Meta-Analysis of Randomized Controlled Trials. Int J Dermatol Venereol. 2024;7(4):216.

[26]	Armstrong AW, Soliman AM, Betts KA, et al. Long-term benefit–risk profiles of treatments for moderate-to-severe plaque psoriasis: a network meta-analysis. Dermatol Ther. 2022;12(1):167–184.

[27]	Piragine E, Petri D, Martelli A, et al. Adherence and persistence to biological drugs for psoriasis: systematic review with meta-analysis. J Clin Med. 2022;11(6):1506.

[28]	Gottlieb AB, Kalb RE, Blauvelt A, et al. The efficacy and safety of infliximab in patients with plaque psoriasis who had an inadequate response to etanercept: results of a prospective, multicenter, open-label study. J Am Acad Dermatol. 2012;67(4):642–650.

[29]	Yamauchi PS, Bissonnette R, Teixeira HD, et al. Systematic review of efficacy of anti-tumor necrosis factor (TNF) therapy in patients with psoriasis previously treated with a different anti-TNF agent. J Am Acad Dermatol. 2016;75(3):612-618.e6.

[30]	Piaserico S, Cazzaniga S, Chimenti S, et al. Efficacy of switching between tumor necrosis factor-alfa inhibitors in psoriasis: results from the Italian Psocare registry. J Am Acad Dermatol. 2014;70(2):257-262.e3.

[31]	Boswell ND, Singla S, Gordon KB. Sequencing of Targeted Therapy in Psoriasis: Does it Matter? Am J Clin Dermatol. 2024;25(5):795–810.

[32]	Warren RB, Blauvelt A, Poulin Y, et al. Efficacy and safety of risankizumab vs. secukinumab in patients with moderate-to-severe plaque psoriasis (IMMerge): results from a phase III, randomized, open-label, efficacy-assessor-blinded clinical trial. Br J Dermatol. 2021;184(1):50–59.

[33]	Blauvelt A, Papp KA, Griffiths CEM, et al. Efficacy and safety of guselkumab, an anti-interleukin-23 monoclonal antibody, compared with adalimumab for the continuous treatment of patients with moderate to severe psoriasis: Results from the phase III, double-blinded, placebo- and active comparator-controlled VOYAGE 1 trial. J Am Acad Dermatol. 2017;76(3):405–417.

[34]	Reich K, Armstrong AW, Foley P, et al. Efficacy and safety of guselkumab, an anti-interleukin-23 monoclonal antibody, compared with adalimumab for the treatment of patients with moderate to severe psoriasis with randomized withdrawal and retreatment: Results from the phase III, double-blind, placebo- and active comparator-controlled VOYAGE 2 trial. J Am Acad Dermatol. 2017;76(3):418–431.

[35]	Reich K, Gooderham M, Thaçi D, et al. Risankizumab compared with adalimumab in patients with moderate-to-severe plaque psoriasis (IMMvent): a randomised, double-blind, active-comparator-controlled phase 3 trial. Lancet Lond Engl. 2019;394(10198):576–586.

[36]	Warren RB, Blauvelt A, Bagel J, et al. Bimekizumab versus Adalimumab in Plaque Psoriasis. N Engl J Med. 2021;385(2):130–141.

[37]	Warren RB, Barker JNWB, Finlay AY, et al. Secukinumab for patients failing previous tumour necrosis factor-α inhibitor therapy: results of a randomized open-label study (SIGNATURE). Br J Dermatol. 2020;183(1):60–70.

[38]	Strober B, Paul C, Blauvelt A, et al. Bimekizumab efficacy and safety in patients with moderate to severe plaque psoriasis: Two-year interim results from the open-label extension of the randomized BE RADIANT phase 3b trial. J Am Acad Dermatol. 2023;89(3):486–495.

[39]	Loft N, Halling A-S, Egeberg A, et al. Efficacy of a second interleukin 17 inhibitor in patients with psoriasis: A systematic review and meta-analysis. J Am Acad Dermatol. 2021;84(1):130–138.

[40]	Loft N, Egeberg A, Isufi D, et al. Response to Interleukin-17A Inhibitors According to Prior Biologic Exposures: A Danish Nationwide Study. Acta Derm Venereol. 2023;103:adv12616.

[41]	Papp K, Blauvelt A, Sullivan J, et al. Ixekizumab—An Effective and Safe Treatment for Moderate-to-Severe Plaque Psoriasis in Patients Previously Treated With Other IL-17 Inhibitors: Results From IXORA-P. J Psoriasis Psoriatic Arthritis®. 2019;4(4):180–185.

[42]	Reddy R, Pannu S, Fiumara K, et al. Efficacy of in-class interleukin-23 inhibitor switching: risankizumab following guselkumab failure in moderate-to-severe psoriasis treatment. Br J Dermatol. 2021;184(3):559–561.

[43]	Gasslitter I, Kirsten N, Augustin M, et al. Successful intra-class switching among IL-17 antagonists: a multicentre, multinational, retrospective study. Arch Dermatol Res. 2019;311(5):421–424.

[44]	Daniele SG, Eldirany SA, Damiani G, et al. Structural Basis for p19 Targeting by Anti-IL-23 Biologics: Correlations with Short- and Long-Term Efficacy in Psoriasis. JID Innov Skin Sci Mol Popul Health. 2024;4(2):100261.

[45]	Camela E, Ocampo‐Garza SS, Cinelli E, et al. Therapeutic update of biologics and small molecules for scalp psoriasis: a systematic review. Dermatol Ther. 2021;34(2):e14857.

[46]	Mosca M, Hong J, Hadeler E, et al. Scalp psoriasis: a literature review of effective therapies and updated recommendations for practical management. Dermatol Ther. 2021;11(3):769–797.

[47]	Tsiogkas SG, Karamitrou EK, Grammatikopoulou MG, et al. Efficacy of tyrosine-kinase-2 and phosphodiesterase-4 inhibitors for scalp psoriasis: a systematic review and meta-analysis. Curr Med Res Opin. 2024;40(2):155–163.

[48]	Duffin KC, Griffiths CE, Hoffmann M, et al. Deucravacitinib, an Oral Selective Tyrosine Kinase 2 (TYK2) Inhibitor, in Patients with Moderate to Severe Scalp Psoriasis: Efficacy and Safety Results of a Phase 3b/4, Multicenter, Randomized, Double-blinded, Placebo-controlled Trial (PSORIATYK SCALP). SKIN J Cutan Med. 2024;8(6):s420–s420.

[49]	Khan M, Wallace CE, Ahmed F, et al. Assessing Comparative Efficacy of Biologics for the Treatment of Psoriasis With Nail Involvement: A Systematic Review. J Psoriasis Psoriatic Arthritis®. 2024;9(2):61–68.

[50]	Hwang JK, Ricardo JW, Lipner SR. Efficacy and safety of nail psoriasis targeted therapies: a systematic review. Am J Clin Dermatol. 2023;24(5):695–720.

[51]	Hong JJ, Mosca ML, Hadeler EK, et al. Genital and inverse/intertriginous psoriasis: an updated review of therapies and recommendations for practical management. Dermatol Ther. 2021;11(3):833–844.

[52]	Beck KM, Yang EJ, Sanchez IM, et al. Treatment of Genital Psoriasis: A Systematic Review. Dermatol Ther. 2018;8(4):509–525.

[53]	Tsiogkas SG, Grammatikopoulou MG, Kontouli K-M, et al. Efficacy of biologic agents for palmoplantar psoriasis: a systematic review and network meta-analysis. Expert Rev Clin Immunol. 2023;19(12):1485–1498.

[54]	Huang I-H, Wu P-C, Chiu H-Y, et al. Small-molecule inhibitors and biologics for palmoplantar psoriasis and palmoplantar pustulosis: a systematic review and network meta-analysis. Am J Clin Dermatol. 2024;25(3):347–358.

[55]	Xia R, Diao Z, Chen D, et al. Efficacy of anti-interleukin-17A biological agents for palmoplantar psoriasis and palmoplantar pustulosis: A network meta-analysis. Int Immunopharmacol. 2024;130:111716.

[56]	Reynolds KA, Pithadia DJ, Lee EB, et al. A systematic review of treatment strategies for erythrodermic psoriasis. J Dermatol Treat. 2021;32(1):49–55.

[57]	Lo Y, Tsai T-F. Updates on the treatment of erythrodermic psoriasis. Psoriasis Targets Ther. 2021;59–73.

[58]	Zhou T, Koussiouris J, Kim L, et al. Management of guttate psoriasis: a systematic review. J Cutan Med Surg. 2024;28(6):577–584.

[59]	Rega F, Trovato F, Bortone G, et al. Therapeutic potential of Spesolimab-Sbzo in the management of generalized pustular psoriasis flares in adults: evidence to date. Psoriasis Targets Ther. 2024;23–27.

[60]	Puig L, Fujita H, Thaçi D, et al. Current treatments for generalized pustular psoriasis: a narrative summary of a systematic literature search. Dermatol Ther. 2024;14(9):2331–2378.

[61]	Chen B, Liu Q, Dong X, et al. Biologics for generalized pustular psoriasis: a systematic review and single-arm meta-analysis. Front Immunol. 2024;15:1462158.

[62]	Kearns DG, Chat VS, Zang PD, et al. Review of treatments for generalized pustular psoriasis. J Dermatol Treat. 2021;32(5):492–494.

[63]	Sánchez‐García V, Hernández‐Quiles R, de‐Miguel‐Balsa E, et al. Exposure to biologic therapy before and during pregnancy in patients with psoriasis: systematic review and meta‐analysis. J Eur Acad Dermatol Venereol. 2023;37(10):1971–1990.

[64]	Owczarek W, Walecka I, Lesiak A, et al. The use of biological drugs in psoriasis patients prior to pregnancy, during pregnancy and lactation: a review of current clinical guidelines. Adv Dermatol Allergol Dermatol Alergol. 2020;37(6):821–830.

[65]	Jeong YD, Jo H, Cho H, et al. Biologics use for psoriasis during pregnancy and its related adverse outcomes in pregnant women and newborns: Findings from WHO pharmacovigilance study. Int Arch Allergy Immunol. 2025;186(6):579–593.

[66]	Battista T, Gallo L, Martora F, et al. Biological therapy for psoriasis in cancer patients: an 8-year retrospective real-life study. J Clin Med. 2024;13(7):1940.

[67]	Gupta A, Peyrin-Biroulet L, Ananthakrishnan AN. Risk of cancer recurrence in patients with immune-mediated diseases with use of immunosuppressive therapies: an updated systematic review and meta-analysis. Clin Gastroenterol Hepatol. 2024;22(3):499–512.

[68]	Russell MD, Stovin C, Alveyn E, et al. JAK inhibitors and the risk of malignancy: a meta-analysis across disease indications. Ann Rheum Dis. 2023;82(8):1059–1067.

[69]	Alhalabi MM, Almokdad R. The incidence of hepatitis B reactivation in patients receiving ustekinumab: a systematic review and proportional meta-analysis. Eur J Gastroenterol Hepatol. 2025;37(1):1–9.

[70]	Kuo MH, Ko P-H, Wang S-T, et al. Incidence of HBV Reactivation in Psoriasis Patients Undergoing Cytokine Inhibitor Therapy: A Single-Center Study and Systematic Review with a Meta-Analysis. Viruses. 2024;17(1):42.

[71]	Li L, Jiang X, Fu L, et al. Reactivation rates of hepatitis B or C or HIV in patients with psoriasis using biological therapies: a systematic review and meta-analysis. Clin Exp Med. 2023;23(3):701–715.

[72]	Megna M, Lauletta G, Tommasino N, et al. Management of psoriasis patients with serious infectious diseases. Adv Ther. 2024;41(6):2099–2111.

[73]	Zhu X, Pan X, Da M, et al. Risk for latent tuberculosis infection reactivation among patients with psoriasis on biologics treatment: a meta-analysis. J Infect. 2024;89(3).

[74]	Mateu-Arrom L, Puig L. Infection Risk and Vaccination in the Management of Psoriasis: Considerations for Biologic Therapy. Psoriasis Targets Ther. 2025;127–144.

[75]	Jafarzadeh A, Behrangi E, Khosravi M, et al. Emerging treatments for dermatologic diseases in infants, children, and adolescents: a systematic review of clinical trials on biologics and small molecule inhibitors. Inflammopharmacology. 2025;1–56.

[76]	Sticherling M, Nikkels AF, Hamza AM, et al. Secukinumab in pediatric patients with plaque psoriasis: pooled safety analysis from two phase 3 randomized clinical trials. Am J Clin Dermatol. 2023;24(5):821–835.

[77]	Li C, Sunhe Y, Zhou H, et al. Efficacy and safety evaluations of adalimumab biosimilars in the treatment of psoriasis. J Dermatol Treat. 2023;34(1):2249145.

[78]	Phan DB, Elyoussfi S, Stevenson M, et al. Biosimilars for the treatment of psoriasis: a systematic review of clinical trials and observational studies. JAMA Dermatol. 2023;159(7):763–771.

[79]	Macaluso FS, Cummings JF, Atreya R, et al. A systematic review on infliximab biosimilar SB2: from pre-clinical data to real-world evidence. Expert Opin Biol Ther. 2022;22(2):203–223.

[80]	Wang Q, Wang J, Sun X, et al. Evidence-based dietary recommendations for patients with psoriasis: A systematic review. Clin Nutr. 2025;47:68–82.

[81]	Wei K, Liao X, Yang T, et al. Efficacy of probiotic supplementation in the treatment of psoriasis—A systematic review and meta‐analysis. J Cosmet Dermatol. 2024;23(7):2361–2367.

[82]	Lu L, Xu Y, Shi M, et al. Psychosocial interventions for psoriasis: a Bayesian network meta-analysis. J Dermatol Treat. 2025;36(1):2427321.

[83]	Wei W, Zhang B, Liu T, et al. Effect of psychological intervention on quality of life among patients with psoriasis: a meta-analysis. Int J Behav Med. 2024;31(6):911–922.

## QUESTION 2. In patients with moderate-to-severe plaque psoriasis, what are the efficacy and safety of cyclosporine?

Two relevant systematic reviews were identified on cyclosporine use in psoriasis. One SR [18] including 10 clinical trials found cyclosporine to be superior to placebo, with higher PASI75 response rates, and reported greater efficacy with higher doses (5 mg/kg) than with lower doses. In head-to-head comparisons, cyclosporine was superior to methotrexate at 7.5 mg and had similar efficacy to methotrexate at 15 mg; it was also associated with a lower discontinuation rate than methotrexate. A more recent SR conducted in China [19], which included 12 randomized controlled trials, confirmed cyclosporine efficacy versus control groups, with improvements in response rate, recurrence, PASI, and several clinical outcomes. Benefit was greater in patients younger than 40 years and without nail involvement. Adverse reaction rates were comparable to those of other treatments, and findings remained stable in sensitivity analyses.

Efficacy and safety of cyclosporine in psoriasis based on Li et al. [18]

| **Outcomes** | **№ of participants (studies)** | **Certainty of evidence (GRADE)** | **Relative effect (95% CI)** | **Relative effect (95% CI)** | |
| --- | --- | --- | --- | --- | --- |
|  |  |  |  | **Risk with control** | **Risk difference with cyclosporine** |
| Response rate | 1016 (12 randomized controlled trials [RCTs]) | ⨁⨁⨁◯ Moderateᵃ | RR 1.09 (1.01 to 1.17) | 837 per 1,000 | 75 more per 1,000 (8 more to 142 more) |
| Recurrence rate | 601 (8 randomized controlled trials [RCTs]) | ⨁⨁⨁⨁ High | RR 0.62 (0.40 to 0.96) | 156 per 1,000 | 59 fewer per 1,000 (94 fewer to 6 fewer) |
| Adverse event rate | 500 (7 randomized controlled trials [RCTs]) | ⨁⨁⨁⨁ High | RR 0.92 (0.77 to 1.09) | 467 per 1,000 | 37 fewer per 1,000 (107 fewer to 42 more) |
| PASI score | 274 (3 randomized controlled trials [RCTs]) | ⨁⨁⨁⨁ High | — | — | SMD 1.21 SD lower (1.47 lower to 0.95 lower) |
| **The risk in the intervention group** (and its 95% confidence interval) is based on the assumed risk in the comparison group and the relative effect of the intervention (and its 95% confidence interval).  **CI:** confidence interval; **RR**: risk ratio; **SMD**: standardized mean difference. | | | | | |
| **GRADE Working Group grades of evidence**  **High certainty:** We are very confident that the true effect is close to the estimate of the effect.  **Moderate certainty:** We have moderate confidence in the effect estimate; the true effect is likely close to the estimate, but it could be materially different.  **Low certainty:** Our confidence in the effect estimate is limited; the true effect may be materially different from the estimate.  **Very low certainty:** We have very little confidence in the effect estimate; the true effect is likely materially different from the estimate. | | | | | |

Explanations

ᵃ Results show wide variability or unexplained heterogeneity.

Efficacy and safety of cyclosporine compared with methotrexate for moderate-to-severe psoriasis

Table adapted from: Pathirana, D., A. D. Ormerod, P. Saiag, C. Smith, P. I. Spuls, A. Nast, J. Barker, et al. ‘European S3-Guidelines on the Systemic Treatment of Psoriasis Vulgaris.’ Journal of the European Academy of Dermatology and Venereology: JEADV 23 Suppl 2 (October 2009): 1–70. doi:10.1111/j.1468-3083.2009.03389.x.

| **Outcome No. of participants (studies)** | **Relative effect (95% CI)** | **Relative effect (95% CI)** | | | **Quality** |
| --- | --- | --- | --- | --- | --- |
|  |  | **Without CSA** | **With CSA** | **Difference** |  |
| PASI 75% (induction)  153 (2 randomized controlled trials [RCTs]) | RR 1.59 (0.78 to 3.26) | Observado | | | ⨁◯◯◯ Very lowᵃ,ᵇ,ᶜ |
|  |  | 43,8% | 696 por 1000  (342 a 1000) | 258 más por 1000  (96 menos a 990 más) |  |
| PGA 0–1 (induction)  114 (1 RCT) | RR 1.37 (0.44 to 4.28) | Observado | | | ⨁◯◯◯ Very lowᵃ,ᵇ,ᶜ |
|  |  | 26,2% | 359 por 1000  (115 a 1000) | 97 más por 1000  (147 menos a 859 más) |  |
| PGA 0–1 (induction)  114 (1 RCT) | RR 1.01 (0.19 to 5.23) | Observado | | | ^⨁^◯◯◯ Very lowᵃ,ᶜ |
|  |  | 31,0% | 313 por 1000  (59 a 1000) | 3 más por 1000  (251 menos a 1311 más) |  |
| Serious adverse events 153  (2 randomized controlled trials [RCTs]) | No events in either arm | | | |  |

***CI: confidence interval; RR: risk ratio.***

***Explanations***

1. Lack of blinding

2. Unexplained differences in results across both studies

3. 95% CI includes both benefit and harm

***References***

Flytstrom I, Stenberg B, Svensson A, Bergbrant IM. Methotrexate vs. ciclosporin in psoriasis: effectiveness, quality of life and safety. A randomized controlled trial. Br J Dermatol. 2008;158(1):116-21.

## QUESTION 3. In patients with moderate-to-severe plaque psoriasis, what are the efficacy and safety of methotrexate?

**Evidence summary**

The evidence considered in the prior version of the guideline [19] included three studies evaluating methotrexate efficacy and safety in adults with moderate-to-severe psoriasis. Results suggest methotrexate is associated with a higher likelihood of achieving clinically relevant responses, including PASI75 and PGA 0–1. In the CHAMPION study and other placebo-controlled comparative trials, methotrexate showed greater efficacy, with larger PASI reductions at 16 weeks and at 6 months. No major adverse events were identified in these psoriasis studies. In contrast, a large meta-analysis in patients with rheumatologic diseases found an increased risk of hepatic adverse events associated with methotrexate, without a statistically significant increase in liver failure, cirrhosis, or mortality..

**Quality of evidence:** Moderate

**Methotrexate compared with placebo for adults with moderate-to-severe psoriasis**

Table adapted from: Pathirana, D., A. D. Ormerod, P. Saiag, C. Smith, P. I. Spuls, A. Nast, J. Barker, et al. ‘European S3-Guidelines on the Systemic Treatment of Psoriasis Vulgaris.’ Journal of the European Academy of Dermatology and Venereology: JEADV 23 Suppl 2 (October 2009): 1–70. doi:10.1111/j.1468-3083.2009.03389.x.)

| **Outcome (participants; studies)** | **Relative effect (95% CI)** | **Without MTX** | **With MTX** | **Difference** | **Quality** | **Interpretation** |
| --- | --- | --- | --- | --- | --- | --- |
| PASI 75 (induction) (n=163; 1 RCT) | RR 1.88 (1.02–3.47) | Observed: 18.9% | 355/1,000 (193–656) | 166 more/1,000 (4 more to 467 more) | ⨁⨁⨁◯ MODERATE¹ | Likely increase in PASI 75 |
| PASI 90 (induction) (n=163; 1 RCT) | RR 1.20 (0.50–2.93) | Low: 11.3% | 136/1,000 (57–331) | 23 more/1,000 (57 fewer to 218 more) | ⨁⨁◯◯ LOW² | Possible increase in PASI 90 |
| PGA 0–1 (n=163; 1 RCT) | RR 2.65 (1.18–5.93) | Observed: 11.3% | 299/1,000 (133–670) | 186 more/1,000 (20 more to 557 more) | ⨁⨁⨁◯ MODERATE¹ | Likely increase in PGA 0–1 |
| PASI 75 (long-term) (n=36; 1 RCT) | RR 3.58 (1.21–10.57) | Observed: 17.6% | 630/1,000 (213–1,000) | 454 more/1,000 (37–1,684) | ⨁⨁⨁◯ MODERATE³ | Likely increase in the probability of PGA 0–1 |
| Serious adverse events (n=163; 1 RCT) | RR 0.48 (0.03–7.50) | Observed: 1.9% | 9/1,000 (1–143) | 10 fewer/1,000 (18 fewer to 124 more) | ⨁⨁◯◯ LOW² | Possible absence of major adverse events |
| Any hepatic adverse event (n=13,177; 32 RCTs) | RR 2.19 (1.73–2.77) | 80/1,000 | 175/1,000 (138–221) | 95 more/1,000 (58 more to 141 more) | ⨁⨁⨁⨁ HIGH | MTX increases hepatic adverse events |
| Serious hepatic adverse events (n=13,177; 32 RCTs) | RR 0.12 (0.01–1.09) | 48/100,000 | 6/100,000 (0–52) | 42 fewer/100,000 (47 fewer to 4 more) | ⨁◯◯◯ VERY LOW²,⁴ | Uncertainty about the effect of MTX on this long-term outcome |
| Clinically relevant fibrosis (n=460; 3 observational studies) | Risk difference pre- vs post-MTX: 9% increase (3 fewer to 20) | — | — | — | ⨁◯◯◯ VERY LOW²,⁵ | Uncertainty about the effect of MTX on this long-term outcome |

CI: confidence interval; RR: risk ratio.

GRADE Working Group grades of evidence

High certainty: We are very confident that the true effect is close to the estimate of the effect

Moderate certainty: We have moderate confidence in the effect estimate; the true effect is likely close to the estimate, but it could be materially different

Low certainty: Our confidence in the effect estimate is limited; the true effect may be materially different from the estimate

Very low certainty: We have very little confidence in the effect estimate; the true effect is likely materially different from the estimate

Explanations

1. 95% CI includes no meaningful benefit

2. 95% CI includes both meaningful benefit and harm

3. Optimal sample size not reached

4. Follow-up in RCTs may have been insufficient to detect this outcome

5. Inadequate design

***References***

1. Ho SG, Yeung CK, Chan HH. Methotrexate versus traditional Chinese medicine in psoriasis: a randomized, placebo-controlled trial to determine
2. efficacy, safety and quality of life. Clin Exp Dermatol. 2010;35(7):717-22.
3. Revicki D, Willian MK, Saurat JH et al. Impact of adalimumab treatment on health-related quality of life and other patient-reported outcomes:
4. results from a 16-week randomized controlled trial in patients with moderate to severe plaque psoriasis. Br J Dermatol. 2008;158(3):549-57.
5. Saurat JH, Stingl G, Dubertret L et al. Efficacy and safety results from the randomized controlled comparative study of adalimumab vs.
6. methotrexate vs. placebo in patients with psoriasis (CHAMPION). Br J Dermatol. 2008;158(3):558-66.
7. Conway, Richard, Candice Low, Robert J. Coughlan, Martin J. O’Donnell, and John J. Carey. ‘Risk of Liver Injury among Methotrexate Users: A
8. Meta-Analysis of Randomised Controlled Trials.’ Seminars in Arthritis and Rheumatism 45, no. 2 (October 2015): 156–62.
9. doi:10.1016/j.semarthrit.2015.05.003.
10. Maybury C.M., Jabbar-Lopez Z.K., Wong T., Dhillon A.P., Barker J.N., and Smith C.H. ‘Methotrexate and Liver Fibrosis in People with Psoriasis: A
11. Systematic Review of Observational Studies.’ British Journal of Dermatology 171, no. 1 (2014): 17–29.

A systematic review [20] synthesized RCTs and other aggregated evidence on MTX dosing (oral, subcutaneous, or intramuscular) in patients with psoriasis. Data were heterogeneous. In RCTs, MTX was generally prescribed at a starting dose of 7.5 mg/week. In other aggregated evidence, MTX was more commonly started at 15 mg/week. Some studies recommended test dosing; other sources did not mention test dosing or even discouraged it.

**Methotrexate 2.5 mg/day compared with MTX 15 mg/week for adults with moderate-to-severe psoriasis**

| **Outcome (participants; studies)** | **Relative effect (95% CI)** | **Without MTX 2.5 mg/day (per 1,000)** | **With MTX 2.5 mg/day (per 1,000)** | **Absolute difference** | **Certainty of evidence (GRADE)** | **Interpretation of results** |
| --- | --- | --- | --- | --- | --- | --- |
| PASI >25 (n=202; 1 RCT) | RR 0.83 (0.76 to 0.93) | 941 per 1,000 | 781 per 1,000 (715 to 875) | 160 fewer per 1,000 (226 fewer to 66 fewer) | ⊕⊕◯◯ Low¹,² | Effectiveness with 2.5 mg/day is possibly lower |
| PASI >75 (n=202; 1 RCT) | RR 0.74 (0.61 to 0.90) | 802 per 1,000 | 593 per 1,000 (489 to 722) | 209 fewer per 1,000 (313 fewer to 80 fewer) | ⊕⊕◯◯ Low¹,² | Effectiveness with 2.5 mg/day is possibly lower |
| Serious adverse events (n=202; 1 RCT) | No serious adverse events were identified at 1 year of follow-up. Nausea, vomiting, and fatigue were less frequent with MTX 2.5 mg/day (4% vs 30%). | | | | ⊕⊕◯◯ Low¹,² |  |

CI: confidence interval; RR: risk ratio.

GRADE Working Group grades of evidence

High certainty: We are very confident that the true effect is close to the estimate of the effect

Moderate certainty: We have moderate confidence in the effect estimate; the true effect is likely close to the estimate, but it could be materially different

Low certainty: Our confidence in the effect estimate is limited; the true effect may be materially different from the estimate

Very low certainty: We have very little confidence in the effect estimate; the true effect is likely materially different from the estimate

**Explanations**

1. Substantial loss to follow-up

2. Optimal sample size not reached

***References***

Radmanesh, Mohammad, Behnam Rafiei, Zahra-Beigum Moosavi, and Niloofar Sina. ‘Weekly vs. Daily Administration of Oral Methotrexate (MTX) for Generalized Plaque Psoriasis: A Randomized Controlled Clinical Trial.’ International Journal of Dermatology 50, no. 10 (October 2011): 1291–93. doi:10.1111/j.1365-4632.2011.04967. x.

**Subcutaneous MTX compared with oral MTX for adults with moderate-to-severe psoriasis**

| **Outcome (participants; studies)** | **Relative effect (95% CI)** | **Anticipated absolute effects** | | | **Quality** | **Interpretation of results** |
| --- | --- | --- | --- | --- | --- | --- |
|  |  | **Without SC MTX** | **With SC MTX** | **Absolute difference** |  |  |
| ACR20 at 24 weeks (n=375; 1 RCT) | RR 1.11 (0.98 to 1.26) | Observed: 70.0% | 777 per 1,000 (686 to 882) | 77 more per 1,000 (14 fewer to 182 more) | ⨁⨁◯◯ Low¹,² | Possible improvement in symptoms |
| ACR70 at 24 weeks (n=381; 1 RCT) | RR 1.23 (0.93 to 1.63) | Observed: 33.0% | 406 per 1,000 (307 to 538) | 76 more per 1,000 (23 fewer to 208 more) | ⨁⨁◯◯ Low¹ | Possible improvement in symptoms |
| Serious adverse events (n=375; 1 RCT) | RR 1.3 (0.5 to 3.5) | Observed: 4.3% | 56 per 1,000 (22 to 151) | 13 more per 1,000 (22 fewer to 107 more) | ⨁⨁◯◯ Low² | Possible absence of meaningful differences in serious adverse events |
| Adverse events leading to discontinuation (n=375; 1 RCT) | RR 2.10 (0.92 to 5.40) | Observed: 4.3% | 90 per 1,000 (40 to 232) | 47 more per 1,000 (3 fewer to 189 more) | ⨁⨁◯◯ Low² | Possible increase in adverse events leading to discontinuation |

CI: confidence interval; RR: risk ratio.

GRADE Working Group grades of evidence

High certainty: We are very confident that the true effect is close to the estimate of the effect

Moderate certainty: We have moderate confidence in the effect estimate; the true effect is likely close to the estimate, but it could be materially different

Low certainty: Our confidence in the effect estimate is limited; the true effect may be materially different from the estimate

Very low certainty: We have very little confidence in the effect estimate; the true effect is likely materially different from the estimate

Explanations

1. Rheumatoid arthritis population; no psoriasis studies.

2. CI includes no benefit.

3. CI includes both meaningful benefit and harm.

***References***

Braun, J., P. Kästner, P. Flaxenberg, J. Währisch, P. Hanke, W. Demary, U. von Hinüber, et al. ‘Comparison of the Clinical Efficacy and Safety of Subcutaneous versus Oral Administration of Methotrexate in Patients with Active Rheumatoid Arthritis: Results of a Six-Month, Multicenter, Randomized, Double-Blind, Controlled, Phase IV Trial.’ Arthritis and Rheumatism 58, no. 1 (January 2008): 73–81. doi:10.1002/art.23144.

## QUESTION 4. In adults with moderate-to-severe plaque psoriasis, what are the efficacy and safety of acitretin?

**Evidence summary**

The evidence considered in the prior version of the guideline [20] included two RCTs (n=194) showing a higher likelihood of achieving PASI75 with acitretin versus placebo. At week 12, 47% to 69% of patients achieved PASI75, with better responses at higher doses. Acitretin was associated with frequent minor adverse events, including scaling, pruritus, alopecia, rhinitis, spasms, and erythema; incidence increased with higher dosing (50 vs 25 mg/kg/day). Other effects, such as cheilitis, dry mouth, and xerophthalmia, were similar across doses.

**Quality of evidence:** Moderate

In the evidence update, one systematic literature review evaluated acitretin combined with methotrexate [21]. Although earlier experience with etretinate plus methotrexate led to this combination being contraindicated due to hepatitis risk, the available evidence suggests combination therapy may increase efficacy without a meaningful rise in hepatotoxicity, except among patients who consume alcohol. Additional studies and real-world data show better skin outcomes with the combination and a similar incidence of liver fibrosis compared with methotrexate monotherapy.

**Quality of evidence:** Very low

Eficacia y seguridad de acitretina comparada con placebo para adultos con psoriasis moderada a severa

| **Outcome (participants; studies)** | **Relative effect (95% CI)** | **Anticipated absolute effects** | | | **Quality** | **Interpretation of results** |
| --- | --- | --- | --- | --- | --- | --- |
|  |  | **Without acitretin** | **With acitretin** | **Absolute difference** |  |  |
| 75% improvement (PASI, global score, or number of plaques)  194 (2 randomized controlled trials [RCTs]) | RR 2.21 (1.40 to 3.50) | 176 per 1,000 | 389 per 1,000 (246 to 616) | 213 more per 1,000 (70 more to 440 more) | ⨁⨁⨁◯ Moderateᵃ | Likely increases the probability of achieving PASI75 |
| Adverse events  n= 504 (2 randomized controlled trials [RCTs]) | Low-dose incidence: cheilitis 70%, scaling 30%, pruritus 26%, alopecia 13%, rhinitis 17%, spasms 13%, erythema 4%, dry mouth 13%, xerophthalmia 13%. High-dose incidence: cheilitis 68%, scaling 64%, pruritus 43%, alopecia 46%, rhinitis 39%, spasms 43%, erythema 18%, dry mouth 18%, xerophthalmia 18%. | | | | ⨁⨁⨁⨁ High | Increased risk of adverse events |

CI: confidence interval; RR: risk ratio.

GRADE Working Group grades of evidence

High certainty: We are very confident that the true effect is close to the estimate of the effect

Moderate certainty: We have moderate confidence in the effect estimate; the true effect is likely close to the estimate, but it could be materially different

Low certainty: Our confidence in the effect estimate is limited; the true effect may be materially different from the estimate

Very low certainty: We have very little confidence in the effect estimate; the true effect is likely materially different from the estimate

Explanations

1. Too few events to reach the optimal sample size.

***References***

Lassus, A., and J. M. Geiger. ‘Acitretin and Etretinate in the Treatment of Palmoplantar Pustulosis: A Double-Blind Comparative Trial.’ The British Journal of Dermatology 119, no. 6 (December 1988): 755–59.

Goldfarb, M. T., C. N. Ellis, A. K. Gupta, T. Tincoff, T. A. Hamilton, and J. J. Voorhees. ‘Acitretin Improves Psoriasis in a Dose-Dependent Fashion.’ Journal of the American Academy of Dermatology 18, no. 4 Pt 1 (April 1988): 655–62.

Pearce, Daniel J., Stephen Klinger, Kristin K. Ziel, Emma J. Murad, Richard Rowell, and Steven R. Feldman. ‘Low-Dose Acitretin Is Associated with Fewer Adverse Events than High-Dose Acitretin in the Treatment of Psoriasis.’ Archives of Dermatology 142, no. 8 (August 2006): 1000–1004. doi:10.1001/archderm.142.8.1000.

Efficacy and safety of acitretin compared with etanercept (ETN) for adults with moderate-to-severe psoriasis

Table adapted from: Pathirana, D., A. D. Ormerod, P. Saiag, C. Smith, P. I. Spuls, A. Nast, J. Barker, et al. ‘European S3-Guidelines on the Systemic Treatment of Psoriasis Vulgaris.’ Journal of the European Academy of Dermatology and Venereology: JEADV 23 Suppl 2 (October 2009): 1–70. [doi:10.1111/j.1468-3083.2009.03389.x.](https://doi.org/10.1111/j.1468-3083.2009.03389.x.)

| **Outcome (participants; studies)** | **Relative effect (95% CI)** | **Anticipated absolute effects** | | | **Quality** | **Interpretation of results** |
| --- | --- | --- | --- | --- | --- | --- |
|  |  | **Without ETN** | **With ETN** | **Absolute difference** |  |  |
| PASI 75% (induction)  102 (2 randomized controlled trials [RCTs]) | RR 0.47 (0.25 to 0.86) | Observed: 42.3% | 199 per 1,000 (106 to 364) | 224 fewer per 1,000 (317 fewer to 59 fewer) | ⨁⨁◯◯ Lowᵃ,ᵇ | ETN may be more effective than acitretin for achieving PASI75 |
| PASI 75 (long-term)  42 (1 randomized controlled trial [RCT]) | RR 0.66 (0.29 to 1.49) | Low: 45.5% | 300 per 1,000 (132 to 678) | 155 fewer per 1,000 (323 fewer to 223 more) | ⨁◯◯◯ Very lowᵃ,ᵇ,ᶜ | Uncertainty about the effect on this outcome |
| Serious adverse events or discontinuations due to adverse events.  102 (2 randomized controlled trials [RCTs]) | — | — | — | No events observed | — | — |

CI: confidence interval; RR: risk ratio.

**Explanations**

a. Lack of blinding

b. Baseline PASI was low

c. 95% CI includes both benefit and harm

***References***

Caproni M, Antiga E, Melani L, Volpi W, Del Bianco E, Fabbri P. Serum levels of IL-17 and IL-22 are reduced by etanercept, but not by acitretin, in patients with psoriasis: a randomized- controlled trial. J Clin Immunol. 2009;29(2):210-4.

Dogra S, Jain A, Kanwar AJ. Efficacy and safety of acitretin in three fixed doses of 25, 35 and 50 mg in adult patients with severe plaque type psoriasis: A randomized, double blind, parallel group, dose ranging study. J Eur Acad Dermatol Venereol. 2013;27(3):e305-e11

## QUESTION 5. In adults with moderate-to-severe plaque psoriasis, what are the efficacy and safety of phototherapy?

**Evidence summary**

The evidence from the prior version of the guideline [20] included one RCT (n=46) comparing narrowband UVB (NB-UVB) phototherapy versus placebo, showing uncertainty about benefit and no clear reduction in recurrences. In addition, a Cochrane systematic review [23] including 13 RCTs (n=662) compared NB-UVB with broadband UVB and PUVA. In one RCT versus oral PUVA, there were no meaningful differences in PASI75; across other studies, clearance outcomes were inconsistent versus both oral PUVA and bath PUVA.

**Quality of evidence:** Low

Efficacy and safety of NB-UVB vs placebo for adults with moderate-to-severe psoriasis

| **Outcome (participants; studies)** | **Relative effect (95% CI)** | **Anticipated absolute effects** | | | **Quality** |
| --- | --- | --- | --- | --- | --- |
|  |  | **Without NBUBVB** | **With NBUBVB** | **Absolute difference** |  |
| PASI 75% (maintenance); follow-up: 1 year 46 (1 RCT) | RR 2.18 (0.99 to 4.82) | 333 per 1,000 | 727 per 1,000 (330 to 1,000) | 393 more per 1,000 (3 fewer to 1,273 more) | ⨁◯◯◯ Very low¹,² |
| Recurrence; follow-up: 1 year 46 (1 RCT) | RR 1.09 (0.61 to 1.90) | 542 per 1,000 | 590 per 1,000 (330 to 1,000) | 49 more per 1,000 (211 fewer to 487 more) | ⨁⨁◯◯ Low¹,² |
| Serious adverse events 46 (1 RCT) | — | — | In the NB-UVB arm, 8% developed painful erythema | — | ⨁⨁⨁◯ Moderate¹ |

CI: confidence interval; RR: relative risk.

***Explanations***

1. Method for allocation concealment not reported

2. Few events and few patients

***References***

Boztepe, Gonca, Ayşen Karaduman, Sedef Sahin, Murat Hayran, and Fikret Kölemen. ‘The Effect of Maintenance Narrow-Band Ultraviolet B Therapy on the Duration of Remission for Psoriasis: A Prospective Randomized Clinical Trial.’ International Journal of Dermatology45, no. 3 º(March 2006): 245–50. doi:10.1111/j.1365-4632.2004.02398.x.

Efficacy and safety of NB-UVB vs oral PUVA for plaque psoriasis

| **Outcome** | **Assumed risk (oral PUVA)** | **Corresponding risk (NB-UVB)** | **Relative effect (95% CI)** | **No. of participants (studies)** | **Certainty of evidence (GRADE)** | **Comments** |
| --- | --- | --- | --- | --- | --- | --- |
| Proportion achieving PASI 75 | 720 per 1,000 | 655 per 1,000 (454 to 950) | RR 0.91 (0.63 to 1.32) | 51 (1 study) | ⊕⊕◯◯ Low¹,² | Intention-to-treat (ITT) analysis |
| Withdrawal due to adverse effects | 32 per 1,000 | 50 per 1,000 (7 to 82) | RR 0.71 (0.20 to 2.54) | 247 (3 studies) | ⊕⊕◯◯ Low¹ | Intention-to-treat (ITT) analysis |
| Clearance rate | Study population | See comment | Not estimable | 0 (0) | See comment | Results from 3 small RCTs are inconsistent. Due to marked statistical heterogeneity, data were not pooled. |

CI: confidence interval; RR: relative risk.

***Explanations***

1. Small sample size

2. High risk of bias

3. The 3 studies were small and at high risk of bias; the outcome was based on fewer than 300 participants.

***References***

Cheng, Y., Liu, G. J., & Zhang, M. (2013). Narrow‐band ultraviolet B phototherapy versus broad‐band ultraviolet B or psoralen‐ultraviolet A photochemotherapy for psoriasis. *Cochrane Database of Systematic Reviews*, (10).

Efficacy and safety of NB-UVB vs bath PUVA for plaque psoriasis

| **Outcome** | **Comparative effect (95% CI)** | | **Relative effect (95% CI)** | **No. of participants (studies)** | **Certainty of evidence (GRADE)** | **Comments** |
| --- | --- | --- | --- | --- | --- | --- |
|  | **Assumed risk** | **Corresponding risk** |  |  |  |  |
|  | **Bath PUVA (assumed risk)** | **NB-UVB (corresponding risk)** |  |  |  |  |
| Clearance rate | 348 per 1,000 | 623 per 1,000 (160 to 1,000) | RR 1.79 (0.46 to 6.91) | 92 (2 studies) | ⨁⨁◯◯ Low¹ | 1) Based on studies using left–right body comparisons. 2) ITT analysis. |
| Clearance rate | 611 per 1,000 | 110 per 1,000 (31 to 434) | RR 0.18 (0.05 to 0.71) | 36 (1 study) | ⨁⨁◯◯ Low²,³ | 1) Based on the study using between-participant comparisons. 2) ITT analysis. |

CI: confidence interval; RR: relative risk.

***Explanations***

1. Both studies were small and at high risk of bias; the outcome was based on fewer than 300 participants.

2. High risk of bias.

3. Small sample size; the outcome was based on fewer than 300 participants.

***References***

Cheng, Y., Liu, G. J., & Zhang, M. (2013). Narrow‐band ultraviolet B phototherapy versus broad‐band ultraviolet B or psoralen‐ultraviolet A photochemotherapy for psoriasis. *Cochrane Database of Systematic Reviews*, (10).

Efficacy and safety of NB-UVB compared with selective BB-UVB for chronic plaque psoriasis

| **Outcome** | **Comparative effect (95% CI)** | | **Relative effect (95% CI)** | **No. of participants (studies)** | **Certainty of evidence (GRADE)** | **Comments** |
| --- | --- | --- | --- | --- | --- | --- |
|  | **Assumed risk** | **Corresponding risk** |  |  |  |  |
|  | **BB-UVB selective** | **NB-UVB** |  |  |  |  |
| Withdrawal due to adverse effects | 20 per 1,000 | 60 per 1,000 (6 to 557) | RR 3.00 (0.32 to 27.87) | 100 (1 study) | ⊕⊕◯◯ Low¹,² | ITT analysis |
| Clearance rate | 400 per 1,000 | 560 per 1,000 (368 to 852) | RR 1.40 (0.92 to 2.13) | 100 (1 study) | ⊕⊕◯◯ Low¹,² | ITT analysis |

IC: Intervalo de confianza; RR: Riesgo relativo

CI: confidence interval; RR: relative risk.

***Explanations***

*1. Unclear risk of bias.*

*2. Small sample size; the outcome was based on fewer than 300 participants.*

***References***

Cheng, Y., Liu, G. J., & Zhang, M. (2013). Narrow‐band ultraviolet B phototherapy versus broad‐band ultraviolet B or psoralen‐ultraviolet A photochemotherapy for psoriasis. *Cochrane Database of Systematic Reviews*, (10).

**References**

1.	Yasmeen N, Sawyer LM, Malottki K, Levin LÅ, Didriksen Apol E, Jemec GB. Targeted therapies for patients with moderate-to-severe psoriasis: a systematic review and network meta-analysis of PASI response at 1 year. J Dermatol Treat. 2022 Jan 2;33(1):204–18.

2.	Belinchón Romero I, Dauden E, Ferrándiz Foraster C, González-Cantero Á, Carrascosa Carrillo JM. Therapeutic goals and treatment response evaluation in moderate to severe psoriasis: an experts opinion document. Ann Med. 2021 Jan;53(1):1727–36.

3.	Galluzzo M, Talamonti M, Manfreda V, Silvaggio D, Tartaglia C, Roberto D, et al. Impact of secukinumab on patient-reported outcomes in moderate to severe plaque psoriasis: a review of clinical studies. Expert Opin Biol Ther. 2021 Feb 1;21(2):191–200.

4.	Rusk AM, Fleischer AB. In psoriasis treatment, greater improvement in skin severity predicts greater improvement in nail severity. J Dermatol Treat. 2021 Nov 17;32(8):894–7.

5.	Belinchon Romero I, Mateu Puchades A, Ribera Pibernat M, Ruiz Genao DianaP, De La Cueva Dobao P, Carrascosa JM. Criteria used to define tumor necrosis factor-alpha inhibitors failure in patients with moderate-to-severe psoriasis: a systematic literature review. Ann Med. 2023 Dec 12;55(1):1335–45.

6.	Gisondi P, Di Mercurio M, Idolazzi L, Girolomoni G. Concept of Remission in Chronic Plaque Psoriasis. J Rheumatol Suppl. 2015 Nov 1;93(0):57–60.

7.	Puig L. PASI 90 response: the new standard in therapeutic efficacy for psoriasis. J Eur Acad Dermatol Venereol. 2015 Apr;29(4):645–8.

8.	Boswell ND, Singla S, Gordon KB. Sequencing of Targeted Therapy in Psoriasis: Does it Matter? Am J Clin Dermatol. 2024 Sept;25(5):795–810.

9.	Thaçi D, Piaserico S, Gaarn Du Jardin K, Warren RB. Long‐term stability of PASI <3 response to tildrakizumab: Pooled analyses from reSURFACE 1 and reSURFACE 2 phase 3 trials through 5 years. J Eur Acad Dermatol Venereol [Internet]. 2023 Apr [cited 2024 Oct 4];37(4). Available from: https://onlinelibrary.wiley.com/doi/10.1111/jdv.18783

10.	Imafuku S, Ohata C, Okubo Y, Tobita R, Saeki H, Mabuchi T, et al. Effectiveness of brodalumab in achieving treatment satisfaction for patients with plaque psoriasis: The ProLOGUE study. J Dermatol Sci. 2022 Mar;105(3):176–84.

11.	Reich K, Mrowietz U, Menter A, Griffiths CEM, Bagel J, Strober B, et al. Effect of baseline disease severity on achievement of treatment target with apremilast: results from a pooled analysis. J Eur Acad Dermatol Venereol. 2021 Dec;35(12):2409–14.

12.	Campanati A, Diotallevi F, Radi G, Molinelli E, Brisigotti V, Martina E, et al. Psoriatic patients treated with secukinumab reach high levels of minimal disease activity: results from the SUPREME study. Eur J Dermatol. 2021 Oct;31(5):630–7.

13.	Lebwohl MG, Soliman AM, Yang H, Wang J, Koenigsberg SH, Anderson A, et al. 25572 Impact of risankizumab on the duration of PASI90 achievement: A post hoc analysis of 4 phase III clinical trials. J Am Acad Dermatol. 2021 Sept;85(3):AB62.

14.	Puig L, Zhu B, Burge R, Shrom D, Dong Y, Shen W, et al. Early Treatment Targets for Predicting Long-term Dermatology Life Quality Index Response in Patients with Moderate-to-Severe Plaque Psoriasis: A Post-hoc Analysis from a Long-term Clinical Study. J Clin Aesthetic Dermatol. 2020 Oct;13(10):18–22.

15.	Puig L, Dossenbach M, Berggren L, Ljungberg A, Zachariae C. Absolute and Relative Psoriasis Area and Severity Indices (PASI) for Comparison of the Efficacy of Ixekizumab to Etanercept and Placebo in Patients with Moderate-to-severe Plaque Psoriasis: An Integrated Analysis of UNCOVER-2 and UNCOVER-3 Outcomes. Acta Derm Venereol. 2019;99(11):971–7.

16.	Merola JF, Amato DA, See K, Burge R, Mallinckrodt C, Ojeh CK, et al. Evaluation of sPGA × BSA as an Outcome Measure and Treatment Target for Clinical Practice. J Invest Dermatol. 2018 Sept;138(9):1955–61.

17.	Elewski BE, Puig L, Mordin M, Gilloteau I, Sherif B, Fox T, et al. Psoriasis patients with psoriasis Area and Severity Index (PASI) 90 response achieve greater health-related quality-of-life improvements than those with PASI 75–89 response: results from two phase 3 studies of secukinumab. J Dermatol Treat. 2017 Aug 18;28(6):492–9.

18.	Schmitt J, Rosumeck S, Thomaschewski G, Sporbeck B, Haufe E, Nast A. Efficacy and safety of systemic treatments for moderate-to-severe psoriasis: meta-analysis of randomized controlled trials. Br J Dermatol. 2014 Feb;170(2):274–303.

19.	Li Z, Pei B, Lei T, Yao M, Niu Y, Zhou P, et al. Clinical features and effects of cyclosporine in the treatment of psoriasis: a systemic review and meta-analysis. Arch Dermatol Res. 2024 Oct 26;316(10):705.

20.	Kogan N, Raimondo N, Gusis SE, Izcovich A, Abarca Duran JA, Barahona‐Torres L, et al. Latin American clinical practice guidelines on the systemic treatment of psoriasis SOLAPSO–Sociedad Latinoamericana de Psoriasis (Latin American Psoriasis Society). Int J Dermatol. 2019;58:4–28.

21.	van Huizen AM, Sikkel R, Caron AG, Menting SP, Spuls PI. Methotrexate dosing regimen for plaque-type psoriasis: an update of a systematic review. J Dermatol Treat. 2022;33(8):3104–18.

22.	Hsieh TS, Tsai TF. Combination therapy for psoriasis with methotrexate and other oral disease-modifying antirheumatic drugs: a systematic review. Dermatol Ther. 2023;13(4):891–909.

23.	Cheng Y, Liu GJ, Zhang M. Narrow‐band ultraviolet B phototherapy versus broad‐band ultraviolet B or psoralen‐ultraviolet A photochemotherapy for psoriasis. Cochrane Database Syst Rev. 2013;(10):CD009481.

24.	Sbidian E, Chaimani A, Garcia-Doval I, Doney L, Dressler C, Hua C, et al. Systemic pharmacological treatments for chronic plaque psoriasis: a network meta-analysis. Cochrane Database Syst Rev. 2022 May 23;5(5):CD011535.

25.	Mahmoud A, Ahmed AAS, Naeem A, Abuelazm M, Elshinawy M, Hassan AR, et al. Tyrosine Kinase-2 Inhibitor (Deucravacitinib) for Psoriasis: A Systematic Review and Network Meta-Analysis of Randomized Controlled Trials. Int J Dermatol Venereol. 2024 Dec;7(4):216.

26.	Armstrong AW, Soliman AM, Betts KA, Wang Y, Gao Y, Stakias V, et al. Long-term benefit–risk profiles of treatments for moderate-to-severe plaque psoriasis: a network meta-analysis. Dermatol Ther. 2022;12(1):167–84.

27.	Piragine E, Petri D, Martelli A, Janowska A, Dini V, Romanelli M, et al. Adherence and persistence to biological drugs for psoriasis: systematic review with meta-analysis. J Clin Med. 2022;11(6):1506.

28.	Gottlieb AB, Kalb RE, Blauvelt A, Heffernan MP, Sofen HL, Ferris LK, et al. The efficacy and safety of infliximab in patients with plaque psoriasis who had an inadequate response to etanercept: results of a prospective, multicenter, open-label study. J Am Acad Dermatol. 2012 Oct;67(4):642–50.

29.	Yamauchi PS, Bissonnette R, Teixeira HD, Valdecantos WC. Systematic review of efficacy of anti-tumor necrosis factor (TNF) therapy in patients with psoriasis previously treated with a different anti-TNF agent. J Am Acad Dermatol. 2016 Sept;75(3):612-618.e6.

30.	Piaserico S, Cazzaniga S, Chimenti S, Giannetti A, Maccarone M, Picardo M, et al. Efficacy of switching between tumor necrosis factor-alfa inhibitors in psoriasis: results from the Italian Psocare registry. J Am Acad Dermatol. 2014 Feb;70(2):257-262.e3.

31.	Boswell ND, Singla S, Gordon KB. Sequencing of Targeted Therapy in Psoriasis: Does it Matter? Am J Clin Dermatol. 2024 Sept;25(5):795–810.

32.	Warren RB, Blauvelt A, Poulin Y, Beeck S, Kelly M, Wu T, et al. Efficacy and safety of risankizumab vs. secukinumab in patients with moderate-to-severe plaque psoriasis (IMMerge): results from a phase III, randomized, open-label, efficacy-assessor-blinded clinical trial. Br J Dermatol. 2021 Jan;184(1):50–9.

33.	Blauvelt A, Papp KA, Griffiths CEM, Randazzo B, Wasfi Y, Shen YK, et al. Efficacy and safety of guselkumab, an anti-interleukin-23 monoclonal antibody, compared with adalimumab for the continuous treatment of patients with moderate to severe psoriasis: Results from the phase III, double-blinded, placebo- and active comparator-controlled VOYAGE 1 trial. J Am Acad Dermatol. 2017 Mar;76(3):405–17.

34.	Reich K, Armstrong AW, Foley P, Song M, Wasfi Y, Randazzo B, et al. Efficacy and safety of guselkumab, an anti-interleukin-23 monoclonal antibody, compared with adalimumab for the treatment of patients with moderate to severe psoriasis with randomized withdrawal and retreatment: Results from the phase III, double-blind, placebo- and active comparator-controlled VOYAGE 2 trial. J Am Acad Dermatol. 2017 Mar;76(3):418–31.

35.	Reich K, Gooderham M, Thaçi D, Crowley JJ, Ryan C, Krueger JG, et al. Risankizumab compared with adalimumab in patients with moderate-to-severe plaque psoriasis (IMMvent): a randomised, double-blind, active-comparator-controlled phase 3 trial. Lancet Lond Engl. 2019 Aug 17;394(10198):576–86.

36.	Warren RB, Blauvelt A, Bagel J, Papp KA, Yamauchi P, Armstrong A, et al. Bimekizumab versus Adalimumab in Plaque Psoriasis. N Engl J Med. 2021 July 8;385(2):130–41.

37.	Warren RB, Barker JNWB, Finlay AY, Burden AD, Kirby B, Armendariz Y, et al. Secukinumab for patients failing previous tumour necrosis factor-α inhibitor therapy: results of a randomized open-label study (SIGNATURE). Br J Dermatol. 2020 July;183(1):60–70.

38.	Strober B, Paul C, Blauvelt A, Thaçi D, Puig L, Lebwohl M, et al. Bimekizumab efficacy and safety in patients with moderate to severe plaque psoriasis: Two-year interim results from the open-label extension of the randomized BE RADIANT phase 3b trial. J Am Acad Dermatol. 2023 Sept;89(3):486–95.

39.	Loft N, Halling AS, Egeberg A, Skov L. Efficacy of a second interleukin 17 inhibitor in patients with psoriasis: A systematic review and meta-analysis. J Am Acad Dermatol. 2021 Jan;84(1):130–8.

40.	Loft N, Egeberg A, Isufi D, Rasmussen MK, Bryld LE, Dam TN, et al. Response to Interleukin-17A Inhibitors According to Prior Biologic Exposures: A Danish Nationwide Study. Acta Derm Venereol. 2023 Nov 21;103:adv12616.

41.	Papp K, Blauvelt A, Sullivan J, Tada Y, Polzer P, Mallbris L, et al. Ixekizumab—An Effective and Safe Treatment for Moderate-to-Severe Plaque Psoriasis in Patients Previously Treated With Other IL-17 Inhibitors: Results From IXORA-P. J Psoriasis Psoriatic Arthritis®. 2019 Oct 1;4(4):180–5.

42.	Reddy R, Pannu S, Fiumara K, Kahn J, Rosmarin D. Efficacy of in-class interleukin-23 inhibitor switching: risankizumab following guselkumab failure in moderate-to-severe psoriasis treatment. Br J Dermatol. 2021 Mar;184(3):559–61.

43.	Gasslitter I, Kirsten N, Augustin M, Torz K, Mrowietz U, Eyerich K, et al. Successful intra-class switching among IL-17 antagonists: a multicentre, multinational, retrospective study. Arch Dermatol Res. 2019 July;311(5):421–4.

44.	Daniele SG, Eldirany SA, Damiani G, Ho M, Bunick CG. Structural Basis for p19 Targeting by Anti-IL-23 Biologics: Correlations with Short- and Long-Term Efficacy in Psoriasis. JID Innov Skin Sci Mol Popul Health. 2024 Mar;4(2):100261.

45.	Camela E, Ocampo‐Garza SS, Cinelli E, Villani A, Fabbrocini G, Megna M. Therapeutic update of biologics and small molecules for scalp psoriasis: a systematic review. Dermatol Ther. 2021;34(2):e14857.

46.	Mosca M, Hong J, Hadeler E, Brownstone N, Bhutani T, Liao W. Scalp psoriasis: a literature review of effective therapies and updated recommendations for practical management. Dermatol Ther. 2021;11(3):769–97.

47.	Tsiogkas SG, Karamitrou EK, Grammatikopoulou MG, Zafiriou E, Bogdanos DP. Efficacy of tyrosine-kinase-2 and phosphodiesterase-4 inhibitors for scalp psoriasis: a systematic review and meta-analysis. Curr Med Res Opin. 2024;40(2):155–63.

48.	Duffin KC, Griffiths CE, Hoffmann M, Blauvelt A, Balagula E, Napoli A, et al. Deucravacitinib, an Oral Selective Tyrosine Kinase 2 (TYK2) Inhibitor, in Patients with Moderate to Severe Scalp Psoriasis: Efficacy and Safety Results of a Phase 3b/4, Multicenter, Randomized, Double-blinded, Placebo-controlled Trial (PSORIATYK SCALP). SKIN J Cutan Med. 2024;8(6):s420–s420.

49.	Khan M, Wallace CE, Ahmed F, Rahman SM, Memon N, Haque A. Assessing Comparative Efficacy of Biologics for the Treatment of Psoriasis With Nail Involvement: A Systematic Review. J Psoriasis Psoriatic Arthritis®. 2024;9(2):61–8.

50.	Hwang JK, Ricardo JW, Lipner SR. Efficacy and safety of nail psoriasis targeted therapies: a systematic review. Am J Clin Dermatol. 2023;24(5):695–720.

51.	Hong JJ, Mosca ML, Hadeler EK, Brownstone ND, Bhutani T, Liao WJ. Genital and inverse/intertriginous psoriasis: an updated review of therapies and recommendations for practical management. Dermatol Ther. 2021;11(3):833–44.

52.	Beck KM, Yang EJ, Sanchez IM, Liao W. Treatment of Genital Psoriasis: A Systematic Review. Dermatol Ther. 2018 Dec;8(4):509–25.

53.	Tsiogkas SG, Grammatikopoulou MG, Kontouli KM, Minopoulou I, Goulis DD, Zafiriou E, et al. Efficacy of biologic agents for palmoplantar psoriasis: a systematic review and network meta-analysis. Expert Rev Clin Immunol. 2023;19(12):1485–98.

54.	Huang IH, Wu PC, Chiu HY, Huang YH. Small-molecule inhibitors and biologics for palmoplantar psoriasis and palmoplantar pustulosis: a systematic review and network meta-analysis. Am J Clin Dermatol. 2024;25(3):347–58.

55.	Xia R, Diao Z, Chen D, Wang Y, Zhou C, Gao Y, et al. Efficacy of anti-interleukin-17A biological agents for palmoplantar psoriasis and palmoplantar pustulosis: A network meta-analysis. Int Immunopharmacol. 2024;130:111716.

56.	Reynolds KA, Pithadia DJ, Lee EB, Liao W, Wu JJ. A systematic review of treatment strategies for erythrodermic psoriasis. J Dermatol Treat. 2021 Feb;32(1):49–55.

57.	Lo Y, Tsai TF. Updates on the treatment of erythrodermic psoriasis. Psoriasis Targets Ther. 2021;59–73.

58.	Zhou T, Koussiouris J, Kim L, Vender R. Management of guttate psoriasis: a systematic review. J Cutan Med Surg. 2024;28(6):577–84.

59.	Rega F, Trovato F, Bortone G, Pellacani G, Richetta AG, Dattola A. Therapeutic potential of Spesolimab-Sbzo in the management of generalized pustular psoriasis flares in adults: evidence to date. Psoriasis Targets Ther. 2024;23–7.

60.	Puig L, Fujita H, Thaçi D, Zheng M, Hernandez Daly AC, Leonardi C, et al. Current treatments for generalized pustular psoriasis: a narrative summary of a systematic literature search. Dermatol Ther. 2024;14(9):2331–78.

61.	Chen B lin, Liu Q wei, Dong X wan, Bai Y ping. Biologics for generalized pustular psoriasis: a systematic review and single-arm meta-analysis. Front Immunol. 2024;15:1462158.

62.	Kearns DG, Chat VS, Zang PD, Han G, Wu JJ. Review of treatments for generalized pustular psoriasis. J Dermatol Treat. 2021 July 4;32(5):492–4.

63.	Sánchez‐García V, Hernández‐Quiles R, de‐Miguel‐Balsa E, Giménez‐Richarte Á, Ramos‐Rincón J, Belinchón‐Romero I. Exposure to biologic therapy before and during pregnancy in patients with psoriasis: systematic review and meta‐analysis. J Eur Acad Dermatol Venereol. 2023;37(10):1971–90.

64.	Owczarek W, Walecka I, Lesiak A, Czajkowski R, Reich A, Zerda I, et al. The use of biological drugs in psoriasis patients prior to pregnancy, during pregnancy and lactation: a review of current clinical guidelines. Adv Dermatol Allergol Dermatol Alergol. 2020;37(6):821–30.

65.	Jeong YD, Jo H, Cho H, Jang W, Park J, Lee S, et al. Biologics use for psoriasis during pregnancy and its related adverse outcomes in pregnant women and newborns: Findings from WHO pharmacovigilance study. Int Arch Allergy Immunol. 2025;186(6):579–93.

66.	Battista T, Gallo L, Martora F, Fattore D, Potestio L, Cacciapuoti S, et al. Biological therapy for psoriasis in cancer patients: an 8-year retrospective real-life study. J Clin Med. 2024;13(7):1940.

67.	Gupta A, Peyrin-Biroulet L, Ananthakrishnan AN. Risk of cancer recurrence in patients with immune-mediated diseases with use of immunosuppressive therapies: an updated systematic review and meta-analysis. Clin Gastroenterol Hepatol. 2024;22(3):499–512.

68.	Russell MD, Stovin C, Alveyn E, Adeyemi O, Chan CKD, Patel V, et al. JAK inhibitors and the risk of malignancy: a meta-analysis across disease indications. Ann Rheum Dis. 2023;82(8):1059–67.

69.	Alhalabi MM, Almokdad R. The incidence of hepatitis B reactivation in patients receiving ustekinumab: a systematic review and proportional meta-analysis. Eur J Gastroenterol Hepatol. 2025;37(1):1–9.

70.	Kuo MH, Ko PH, Wang ST, Tseng CW. Incidence of HBV Reactivation in Psoriasis Patients Undergoing Cytokine Inhibitor Therapy: A Single-Center Study and Systematic Review with a Meta-Analysis. Viruses. 2024;17(1):42.

71.	Li L, Jiang X, Fu L, Zhang L, Feng Y. Reactivation rates of hepatitis B or C or HIV in patients with psoriasis using biological therapies: a systematic review and meta-analysis. Clin Exp Med. 2023;23(3):701–15.

72.	Megna M, Lauletta G, Tommasino N, Salsano A, Battista T, Ruggiero A, et al. Management of psoriasis patients with serious infectious diseases. Adv Ther. 2024;41(6):2099–111.

73.	Zhu X, Pan X, Da M, Wang F, Dong Z. Risk for latent tuberculosis infection reactivation among patients with psoriasis on biologics treatment: a meta-analysis. J Infect. 2024;89(3).

74.	Mateu-Arrom L, Puig L. Infection Risk and Vaccination in the Management of Psoriasis: Considerations for Biologic Therapy. Psoriasis Targets Ther. 2025;127–44.

75.	Jafarzadeh A, Behrangi E, Khosravi M, Falakeh S, Amiri JK, Goodarzi A. Emerging treatments for dermatologic diseases in infants, children, and adolescents: a systematic review of clinical trials on biologics and small molecule inhibitors. Inflammopharmacology. 2025;1–56.

76.	Sticherling M, Nikkels AF, Hamza AM, Kwong P, Szepietowski JC, El Sayed M, et al. Secukinumab in pediatric patients with plaque psoriasis: pooled safety analysis from two phase 3 randomized clinical trials. Am J Clin Dermatol. 2023;24(5):821–35.

77.	Li C, Sunhe Y, Zhou H, Dong W. Efficacy and safety evaluations of adalimumab biosimilars in the treatment of psoriasis. J Dermatol Treat. 2023;34(1):2249145.

78.	Phan DB, Elyoussfi S, Stevenson M, Lunt M, Warren RB, Yiu ZZ. Biosimilars for the treatment of psoriasis: a systematic review of clinical trials and observational studies. JAMA Dermatol. 2023;159(7):763–71.

79.	Macaluso FS, Cummings JF, Atreya R, Choi J, Orlando A. A systematic review on infliximab biosimilar SB2: from pre-clinical data to real-world evidence. Expert Opin Biol Ther. 2022;22(2):203–23.

80.	Wang Q, Wang J, Sun X, Liu L, Zhang M, Yu Y, et al. Evidence-based dietary recommendations for patients with psoriasis: A systematic review. Clin Nutr. 2025;47:68–82.

81.	Wei K, Liao X, Yang T, He X, Yang D, Lai L, et al. Efficacy of probiotic supplementation in the treatment of psoriasis—A systematic review and meta‐analysis. J Cosmet Dermatol. 2024;23(7):2361–7.

82.	Lu L, Xu Y, Shi M, Liu A. Psychosocial interventions for psoriasis: a Bayesian network meta-analysis. J Dermatol Treat. 2025;36(1):2427321.

83.	Wei W, Zhang B, Liu T, Lu T. Effect of psychological intervention on quality of life among patients with psoriasis: a meta-analysis. Int J Behav Med. 2024;31(6):911–22.

## QUESTION 6. In adults with moderate-to-severe plaque psoriasis, what are the efficacy and safety of anti-TNF agents?

A network meta-analysis systematic review was identified as the primary evidence source. Results showed anti-TNF agents are effective versus placebo, with a higher likelihood of achieving PASI90 and a clinically relevant improvement in quality of life. For safety, there were no meaningful differences in serious adverse events compared with placebo.

**Efficacy and safety of etanercept for psoriasis based on Sbidian et al.****[22]**

| **Outcome** | **No. of participants (studies)** | **Certainty of evidence (GRADE)** | **Relative effect (95% CI)** | **Anticipated absolute effects** | |
| --- | --- | --- | --- | --- | --- |
|  |  |  |  | **Risk with placebo** | **Risk difference with etanercept** |
| Efficacy assessed as: PASI 90 response | 5650 (14 randomized controlled trials [RCTs]) | ⊕⊕⊕⊕ High | RR 11.68 (8.14 to 16.75) | 15 per 1,000 | 158 more per 1,000 (106 more to 234 more) |
| Quality of life assessed as: validated quality-of-life scale | 3246 (8 randomized controlled trials [RCTs]) | ⊕⊕⊕⊕ High | — | — | SMD 1.11 SD lower (1.34 lower to 0.88 lower) |
| Serious adverse events | 4265 (13 randomized controlled trials [RCTs]) | ⊕⊕⊕⊕ High | RR 0.92 (0.53 to 1.60) | 18 per 1,000 | 1 fewer per 1,000 (9 fewer to 11 more) |

The risk in the intervention group (and its 95% confidence interval) is based on the assumed risk in the comparison group and the relative effect of the intervention (and its 95% confidence interval).

CI: confidence interval; RR: risk ratio; SMD: standardized mean difference.

**Efficacy and safety of adalimumab for psoriasis based on Sbidian et al.****[22]**

| **Outcome** | **No. of participants (studies)** | **Certainty of evidence (GRADE)** | **Relative effect (95% CI)** | **Anticipated absolute effects** | |
| --- | --- | --- | --- | --- | --- |
|  |  |  |  | **Risk with placebo** | **Risk difference with adalimumab** |
| Efficacy assessed as: PASI90 response | 3421 (9 randomized controlled trials [RCTs]) | ⊕⊕⊕⊕ High | RR 13.13 (8.01 to 21.53) | 31 per 1,000 | 372 more per 1,000 (215 more to 629 more) |
| Quality of life assessed as: validated quality-of-life scale | 3055 (9 randomized controlled trials [RCTs]) | ⊕⊕⊕⊕ High | — | — | SMD 0.98 SD lower (1.11 lower to 0.85 lower) |
| Safety assessed as: serious adverse events | 3485 (10 randomized controlled trials [RCTs]) | ⊕⊕⊕⊕ High | RR 1.15 (0.72 to 1.84) | 20 per 1,000 | 3 more per 1,000 (6 fewer to 17 more) |

The risk in the intervention group (and its 95% confidence interval) is based on the assumed risk in the comparison group and the relative effect of the intervention (and its 95% confidence interval).

CI: confidence interval; RR: risk ratio; SMD: standardized mean difference.

**Efficacy and safety of certolizumab for psoriasis based on Sbidian et al.****[22]**

| **Outcome** | **No. of participants (studies)** | **Certainty of evidence (GRADE)** | **Relative effect (95% CI)** | **Anticipated absolute effects** | |
| --- | --- | --- | --- | --- | --- |
|  |  |  |  | **Risk with placebo** | **Risk difference with certolizumab** |
| Efficacy assessed as: PASI90 response | 1153 (5 randomized controlled trials [RCTs]) | ⊕⊕⊕⊕ High | RR 19.77 (8.29 to 47.12) | 17 per 1,000 | 312 more per 1,000 (121 more to 765 more) |
| Quality of life assessed as: validated quality-of-life scale | 588 (3 randomized controlled trials [RCTs]) | ⊕⊕⊕⊕ High | — | — | SMD 0.92 SD lower (1.13 lower to 0.72 lower) |
| Safety assessed as: serious adverse events | 1153 (5 randomized controlled trials [RCTs]) | ⊕⊕⊕⊕ High | RR 0.97 (0.22 to 4.24) | 33 per 1,000 | 1 fewer per 1,000 (26 fewer to 108 more) |

The risk in the intervention group (and its 95% confidence interval) is based on the assumed risk in the comparison group and the relative effect of the intervention (and its 95% confidence interval).

CI: confidence interval; RR: risk ratio; SMD: standardized mean difference.

**Efficacy and safety of infliximab for psoriasis based on Sbidian et al.****[22]**

| **Outcome** | **No. of participants (studies)** | **Certainty of evidence (GRADE)** | **Relative effect (95% CI)** | **Anticipated absolute effects** | |
| --- | --- | --- | --- | --- | --- |
|  |  |  |  | **Risk with placebo** | **Risk difference with infliximab** |
| Efficacy assessed as: PASI 90 response | 1645 (5 randomized controlled trials [RCTs]) | ⊕⊕⊕⊕ High | RR 27.71 (12.52 to 61.30) | 13 per 1,000 | 334 more per 1,000 (144 more to 754 more) |
| Quality of life assessed as: quality-of-life scale | 1645 (5 randomized controlled trials [RCTs]) | ⊕⊕⊕⊕ High | — | — | SMD 1.29 SD lower (1.48 lower to 1.1 lower) |
| Safety assessed as: serious adverse events | 1678 (6 randomized controlled trials [RCTs]) | ⊕⊕⊕⊕ High | RR 1.99 (0.82 to 4.78) | 12 per 1,000 | 12 more per 1,000 (2 fewer to 46 more) |

The risk in the intervention group (and its 95% confidence interval) is based on the assumed risk in the comparison group and the relative effect of the intervention (and its 95% confidence interval).

CI: confidence interval; RR: risk ratio; SMD: standardized mean difference.

## QUESTION 7. In adults with moderate-to-severe plaque psoriasis, what are the efficacy and safety of anti–IL-12/23 agents?

A network meta-analysis systematic review was identified as the primary evidence source. Direct evidence showed the anti–IL-12/23 agent was more effective than placebo for achieving PASI90 and improving quality of life. No relevant differences were observed in serious adverse events versus placebo.

**Efficacy and safety of ustekinumab for psoriasis based on Sbidian et al.****[22]**

| **Outcome** | **No. of participants (studies)** | **Certainty of evidence (GRADE)** | **Relative effect (95% CI)** | **Anticipated absolute effects** | |
| --- | --- | --- | --- | --- | --- |
|  |  |  |  | **Risk with placebo** | **Risk difference with ustekinumab** |
| Efficacy assessed as: PASI90 response | 4520 (11 randomized controlled trials [RCTs]) | ⊕⊕⊕⊕ High | RR 18.37 (12.56 to 26.85) | 21 per 1,000 | 367 more per 1,000 (245 more to 547 more) |
| Quality of life | 3359 (9 randomized controlled trials [RCTs]) | ⊕⊕⊕⊕ High | — | — | SMD 1.35 SD lower (1.54 lower to 1.16 lower) |
| Safety assessed as: serious adverse events | 4842 (12 randomized controlled trials [RCTs]) | ⊕⊕⊕⊕ High | RR 0.98 (0.62 to 1.55) | 17 per 1,000 | 0 fewer per 1,000 (7 fewer to 9 more) |

The risk in the intervention group (and its 95% confidence interval) is based on the assumed risk in the comparison group and the relative effect of the intervention (and its 95% confidence interval).

CI: confidence interval; RR: risk ratio; SMD: standardized mean difference.

## QUESTION 8. In adults with moderate-to-severe plaque psoriasis, what are the efficacy and safety of anti–IL-17 agents?

A Cochrane systematic review evaluating therapies for moderate-to-severe psoriasis was identified. Anti–IL-17 agents were clearly more effective than placebo for achieving PASI responses and improving quality of life, with no meaningful differences in serious adverse events.

**Efficacy and safety of secukinumab for psoriasis based on Sbidian et al.****[22]**

| **Outcome** | **No. of participants (studies)** | **Certainty of evidence (GRADE)** | **Relative effect (95% CI)** | **Anticipated absolute effects** | |
| --- | --- | --- | --- | --- | --- |
|  |  |  |  | **Risk with placebo** | **Risk difference with secukinumab** |
| Efficacy assessed as: PASI response | 4622 (15 randomized controlled trials [RCTs]) | ⊕⊕⊕⊕ High | RR 22.70 (15.53 to 33.19) | 17 per 1,000 | 372 more per 1,000 (249 more to 552 more) |
| Quality of life assessed as: standardized mean difference on QoL questionnaire* | 213 (2 randomized controlled trials [RCTs]) | ⊕⊕⊕⊕ High | — | — | SMD 1.4 SD lower (1.71 lower to 1.09 lower) |
| Safety assessed as: serious adverse events | 4229 (15 randomized controlled trials [RCTs]) | ⊕⊕⊕⊕ High | RR 1.04 (0.64 to 1.69) | 18 per 1,000 | 1 more per 1,000 (7 fewer to 13 more) |

The risk in the intervention group (and its 95% confidence interval) is based on the assumed risk in the comparison group and the relative effect of the intervention (and its 95% confidence interval).

CI: confidence interval; RR: risk ratio; SMD: standardized mean difference.

**Eficacia y seguridad del ixekinumab en el tratamiento de la psoriasis a partir de los resultados de Sbidian et al.****[22]**

| **Outcome** | **No. of participants (studies)** | **Certainty of evidence (GRADE)** | **Relative effect (95% CI)** | **Anticipated absolute effects** | |
| --- | --- | --- | --- | --- | --- |
|  |  |  |  | **Risk with placebo** | **Risk difference with ixekinumab** |
| Efficacy assessed as: participants achieving PASI90 response | 3706 (5 randomized controlled trials [RCTs]) | ⊕⊕⊕⊕ High | RR 47.03 (18.81 to 117.59) | 12 per 1,000 | 558 more per 1,000 (216 more to 1,414 more) |
| Quality of life assessed as: standardized mean difference on QoL questionnaire* | 3564 (4 randomized controlled trials [RCTs]) | ⊕⊕⊕⊕ High | — | — | SMD 1.85 SD lower (2.14 lower to 1.55 lower) |
| Safety assessed as: participants with serious adverse events | 3706 (5 randomized controlled trials [RCTs]) | ⊕⊕⊕⊕ High | RR 0.90 (0.45 to 1.80) | 18 per 1,000 | 2 fewer per 1,000 (10 fewer to 14 more) |

The risk in the intervention group (and its 95% confidence interval) is based on the assumed risk in the comparison group and the relative effect of the intervention (and its 95% confidence interval).

CI: confidence interval; RR: risk ratio; SMD: standardized mean difference.

**Efficacy and safety of brodalumab for psoriasis based on Sbidian et al.****[22]**

| **Outcome** | **No. of participants (studies)** | **Certainty of evidence (GRADE)** | **Relative effect (95% CI)** | **Anticipated absolute effects** | |
| --- | --- | --- | --- | --- | --- |
|  |  |  |  | **Risk with placebo** | **Risk difference with brodalumab** |
| Efficacy assessed as: PASI response | 4109 (5 randomized controlled trials [RCTs]) | ⊕⊕⊕◯ Moderateᵃ | RR 26.33 (16.77 to 41.33) | 20 per 1,000 | 496 more per 1,000 (309 more to 789 more) |
| Quality of life assessed as: standardized mean difference on QoL questionnaire* | 349 (2 randomized controlled trials [RCTs]) | ⊕⊕⊕◯ Moderateᵇ | — | — | SMD 0.96 SD lower (1.44 lower to 0.47 lower) |
| Safety assessed as: serious adverse events | 4109 (5 randomized controlled trials [RCTs]) | ⊕⊕⊕⊕ High | RR 0.92 (0.52 to 1.61) | 17 per 1,000 | 1 fewer per 1,000 (8 fewer to 11 more) |

The risk in the intervention group (and its 95% confidence interval) is based on the assumed risk in the comparison group and the relative effect of the intervention (and its 95% confidence interval).

CI: confidence interval; RR: risk ratio; SMD: standardized mean difference.

**Efficacy and safety of bimekizumab for psoriasis based on Sbidian et al.****[22]**

| **Outcome** | **No. of participants (studies)** | **Certainty of evidence (GRADE)** | **Relative effect (95% CI)** | **Anticipated absolute effects** | |
| --- | --- | --- | --- | --- | --- |
|  |  |  |  | **Risk with placebo** | **Risk difference with bimekizumab** |
| Efficacy assessed as: PASI response | 1089 (3 randomized controlled trials [RCTs]) | ⊕⊕⊕⊕ High | RR 29.43 (10.30 to 84.15) | 24 per 1,000 | 674 more per 1,000 (220 more to 1,970 more) |
| Safety assessed as: serious adverse events | 1089 (3 randomized controlled trials [RCTs]) | ⊕⊕⊕⊕ High | RR 0.58 (0.20 to 1.65) | 24 per 1,000 | 10 fewer per 1,000 (19 fewer to 15 more) |

The risk in the intervention group (and its 95% confidence interval) is based on the assumed risk in the comparison group and the relative effect of the intervention (and its 95% confidence interval).

CI: confidence interval; RR: risk ratio.

## QUESTION 9. In adults with moderate-to-severe plaque psoriasis, what are the efficacy and safety of anti–IL-23 agents?

A network meta-analysis systematic review was identified as the evidence source. Anti–IL-23 agents were more effective than placebo for achieving PASI90 and improving quality of life, with no relevant differences in serious adverse events.

**Efficacy and safety of guselkumab for psoriasis based on Sbidian et al.****[22]**

| **Outcome** | **No. of participants (studies)** | **Certainty of evidence (GRADE)** | **Relative effect (95% CI)** | **Anticipated absolute effects** | |
| --- | --- | --- | --- | --- | --- |
|  |  |  |  | **Risk with placebo** | **Risk difference with guselkumab** |
| Efficacy assessed as: PASI90 response | 1767 (5 randomized controlled trials [RCTs]) | ⊕⊕⊕⊕ High | RR 27.79 (16.23 to 47.60) | 22 per 1,000 | 591 more per 1,000 (336 more to 1,028 more) |
| Quality of life assessed as: validated quality-of-life scale | 1444 (3 randomized controlled trials [RCTs]) | ⊕⊕⊕⊕ High | — | — | SMD 1.36 SD lower (1.54 lower to 1.18 lower) |
| Safety assessed as: serious adverse events | 1767 (5 randomized controlled trials [RCTs]) | ⊕⊕⊕⊕ High | RR 1.07 (0.50 to 2.28) | 17 per 1,000 | 1 more per 1,000 (8 fewer to 21 more) |

The risk in the intervention group (and its 95% confidence interval) is based on the assumed risk in the comparison group and the relative effect of the intervention (and its 95% confidence interval).

CI: confidence interval; RR: risk ratio; SMD: standardized mean difference.

**Efficacy and safety of tildrakizumab for psoriasis based on Sbidian et al.****[22]**

| **Outcome** | **No. of participants (studies)** | **Certainty of evidence (GRADE)** | **Relative effect (95% CI)** | **Anticipated absolute effects** | |
| --- | --- | --- | --- | --- | --- |
|  |  |  |  | **Risk with placebo** | **Risk difference with tildrakizumab** |
| Efficacy assessed as: PASI 90 response | 1903 (3 randomized controlled trials [RCTs]) | ⊕⊕⊕⊕ High | RR 17,26 (8,27 to 36,05) | 20 per 1,000 | 319 more per 1,000 (143 more to 687 more) |
| Quality of life assessed as: validated quality-of-life scale | 1904 (3 RCTs) | ⊕⊕⊕⊕ High | — | — | SMD −1,36 SD — / 1,36 SD lower (1,48 lower to 1,23 lower) |
| Safety assessed as: serious adverse events | 1904 (3 RCTs) | ⊕⊕⊕⊕ High | RR 1,01 (0,37 to 2,77) | 14 per 1,000 | 0 fewer per 1,000 (9 fewer to 25 more) |

The risk in the intervention group (and its 95% CI) is based on the assumed risk in the comparison group and the relative effect of the intervention.

CI: confidence interval; RR: risk ratio; SMD (DME): standardized mean difference.

**Efficacy and safety of risankizumab for psoriasis based on Sbidian et al.****[22]**

| **Outcome** | **No. of participants (studies)** | **Certainty of evidence (GRADE)** | **Relative effect (95% CI)** | **Anticipated absolute effects** | |
| --- | --- | --- | --- | --- | --- |
|  |  |  |  | **Risk with placebo** | **Risk difference with risankizumab** |
| Efficacy assessed as: PASI 90 response | 1633 (5 randomized controlled trials [RCTs]) | ⊕⊕⊕⊕ High | RR 22,51 (12,90 to 39,30) | 29 per 1,000 | 630 more per 1,000 (348 more to 1,121 more) |
| Quality of life assessed as: validated quality-of-life scale | 798 (2 RCTs) | ⊕⊕⊕⊕ High | — | — | SMD 1,82 SD lower (2,04 lower to 1,6 lower) |
| Safety assessed as: serious adverse events | 1633 (5 RCTs) | ⊕⊕⊕⊕ High | RR 0,72 (0,28 to 1,90) | 32 per 1,000 | 9 fewer per 1,000 (23 fewer to 29 more) |

The risk in the intervention group (and its 95% CI) is based on the assumed risk in the comparison group and the relative effect of the intervention.

CI: confidence interval; RR: risk ratio; SMD: standardized mean difference.

## QUESTION 10. In adults with moderate-to-severe plaque psoriasis, what are the efficacy and safety of small molecules?

A network meta-analysis systematic review was identified as the evidence source. Small molecules were more effective than placebo for achieving PASI90 and improving quality of life, with no meaningful differences in serious adverse events.

**Efficacy and safety of apremilast for psoriasis based on Sbidian et al.****[22]**

| **Outcome** | **No. of participants (studies)** | **Certainty of evidence (GRADE)** | **Relative effect (95% CI)** | **Anticipated absolute effects** | |
| --- | --- | --- | --- | --- | --- |
|  |  |  |  | **Risk with placebo** | **Risk difference with apremilast** |
| Efficacy assessed as: PASI 90 response | 2029 (5 randomized controlled trials [RCTs]) | ⊕⊕⊕⊕ High | RR 6,94 (3,37 to 14,30) | 12 per 1,000 | 70 more per 1,000 (28 more to 158 more) |
| Quality of life assessed as: validated quality-of-life scale | 2166 (5 randomized controlled trials [RCTs]) | ⊕⊕⊕⊕ High | — | — | SMD 0,59 SD lower (0,7 lower to 0,47 lower) |
| Safety assessed as: serious adverse events | 2593 (7 randomized controlled trials [RCTs]) | ⊕⊕⊕⊕ High | RR 0,85 (0,48 to 1,52) | 21 per 1,000 | 3 fewer per 1,000 (11 fewer to 11 more) |

The risk in the intervention group (and its 95% CI) is based on the assumed risk in the comparison group and the relative effect of the intervention.

CI: confidence interval; RR: risk ratio; SMD: standardized mean difference.

Evidence on deucravacitinib comes from a systematic review[23] including four RCTs (n=2,156). Treatment was associated with improvements in psoriasis severity, symptoms, physician global assessment, and quality of life. Compared with apremilast, deucravacitinib showed greater efficacy for PASI, DLQI, and overall clinical assessments, including scalp assessments. For safety, there were more mild-to-moderate adverse events than with placebo, with no relevant differences in serious adverse events or discontinuation rates.

**Efficacy and safety of deucravacitinib for psoriasis based on Mahmoud et al.****[23]**

| **Outcome** | **No. of participants (studies)** | **Certainty of evidence (GRADE)** | **Relative effect (95% CI)** | **Anticipated absolute effects** | |
| --- | --- | --- | --- | --- | --- |
|  |  |  |  | **Risk with placebo** | **Risk difference with deucravacitinib** |
| Efficacy assessed as: PASI 90 response | 2029 (2 randomized controlled trials [RCTs]) | ⊕⊕⊕⊕ Highᵃ,ᵇ | RR 9,69 (6,18 to 15,20) | 30 per 1,000 | 261 more per 1,000 (156 more to 427 more) |
| Safety assessed as: serious adverse events | 967 (3 randomized controlled trials [RCTs]) | ⊕⊕◯◯ Lowᵃ,ᵇ | RR 0,39 (0,16 to 0,91) | 40 per 1,000 | 24 fewer per 1,000 (33 fewer to 4 fewer) |

The risk in the intervention group (and its 95% CI) is based on the assumed risk in the comparison group and the relative effect of the intervention.

CI: confidence interval; RR: risk ratio.

**Summary of long-term evidence with biologics**

A network meta-analysis systematic review evaluated long-term efficacy and safety (weeks 48–56) of therapies for moderate-to-severe plaque psoriasis[24]. In the efficacy analysis, anti-TNF agents generally showed lower PASI75/90/100 responses than anti-interleukin biologics.

***Summary of long-term efficacy outcomes of biologics in psoriasis (48 to 52 weeks)***

| **Molecule** | **PASI75, % (95% CI)** | **PASI90, % (95% CI)** | **PASI100, % (95% CI)** |
| --- | --- | --- | --- |
| Risankizumab | 92,9  (90,5, 94,9) | 84,9  (80,9, 88,3) | 65,4  (59,4, 71,0) |
| Bimekizumab | 90,8  (87,7, 93,3) | 81,3  (76,5, 85,4) | 59,9  (53,3, 66,2) |
| Brodalumab | 89,1  (85,8, 91,7) | 78,6  (73,8, 82,8) | 56,1  (49,9, 62,2) |
| Guselkumab | 88,2  (84,3, 91,4) | 77,3  (71,6, 82,3) | 54,4  (47,4, 61,4) |
| Ixekizumab | 84,6  (77,6, 90,0) | 72,0  (62,6, 80,0) | 47,8  (37,5, 58,1) |
| Secukinumab | 80,4  (77,2, 83,3) | 66,2  (62,2, 70,1) | 41,3  (37,1, 45,7) |
| Ustekinumab | 71,4  (69,3, 73,4) | 55,1  (52,9, 57,2) | 30,5  (28,4, 32,7) |
| Adalimumab | 67,7  (58,4, 75,9) | 50,8  (41,2, 60,4) | 26,9  (19,4, 35,5) |
| Etanercept | 54,7  (46,6, 62,6) | 37,4  (30,1, 45,3) | 16,9  (12,3, 22,5) |

Source: constructed from Armstrong AW, et al. Ther. 2022;12(1):167-84.

***Summary of long-term safety outcomes of biologics in psoriasis (48 to 52 weeks)***

| **Molecule** | **Any adverse event,**  **median % (95% CI)** | **Serious adverse event,**  **median % (95% CI)** | **Adverse event leading to discontinuation,**  **median % (95% CI)** |
| --- | --- | --- | --- |
| Risankizumab | 67,5  (57,8, 75,5) | 4,4  (2,4, 8,0) | 0,9  (0,2, 4,0) |
| Guselkumab | 72,2  (63,6, 79,5) | 5,9  (3,1, 11,0) | 2,5  (0,9, 7,1) |
| Adalimumab | 72,9  (61,3, 82,0) | 5,4  (2,1, 13,4) | 3,4  (0,8, 12,6) |
| Secukinumab | 76,6  (71,7, 80,9) | 6,9  (4,4, 10,6) | 3,2  (1,7, 6,1) |
| Ustekinumab | 76,9  (74,5, 79,2) | 5,7  (4,5, 7,1) | 2,2  (1,5, 3,2) |
| Ixekizumab | 80,9  (68,7, 89,3) | 10,5  (3,8, 27,2) | 4,3  (0,6, 28,9) |
| Bimekizumab | 82,3  (74,4, 88,2) | 7,2  (3,4, 14,5) | 4,1  (1,4, 11,5) |

Source: constructed from Armstrong AW, et al. Ther. 2022;12(1):167-84.

A systematic review of observational studies assessed adherence and persistence with biologic therapy in psoriasis[25] showing moderate overall proportions and marked variability across agents.

***Summary of pooled comparative survival analyses of biologics in psoriasis***

| ***Pairwise comparisons*** | **SEC vs. ADA** | **SEC vs. ETA** | **SEC vs. INF** | **SEC vs. IXE** | **SEC vs. UST** |  |
| --- | --- | --- | --- | --- | --- | --- |
| ***Follow-up: 1 year*** |  |  |  |  |  |  |
| *Pooled HR* | 0,99 (0,70–1,40) | 1,10 (0,90–1,35) | 1,07 (0,85–1,36) | 2,01 (1,09–3,71) | 0,62 (0,47–0,81) |  |
| ***Pairwise comparisons*** | **UST vs. ADA** | **UST vs. ETA** | **UST vs. INF** | **ADA vs. ETA** | **ADA vs. INF** | **ETA vs. INF** |
| ***Follow-up: 2 years*** |  |  |  |  |  |  |
| *Pooled HR* | 1,92 (1,61–2,29) | 2,28 (1,92–2,70) | 2,24 (1,92–2,60) | 1,13 (0,99–1,30) | 1,32 (1,06–1,63) | 1,19 (0,98–1,45) |
| ***Follow-up: 5 years*** |  |  |  |  |  |  |
| *Pooled HR* | **1,48 (1,33–1,65)** | **1,97 (1,68–2,31)** | **2,04 (1,75–2,38)** | **1,31 (1,12–1,54)** | **1,72 (1,52–2,02)** | **1,40 (1,17–1,69)** |

Analyses were performed when sufficient data were available. UST, ustekinumab; ADA, adalimumab; INF, infliximab; HR, hazard ratio.

**References**

1.	Yasmeen N, Sawyer LM, Malottki K, Levin LÅ, Didriksen Apol E, Jemec GB. Targeted therapies for patients with moderate-to-severe psoriasis: a systematic review and network meta-analysis of PASI response at 1 year. J Dermatol Treat. 2022 Jan 2;33(1):204–18.

2.	Belinchón Romero I, Dauden E, Ferrándiz Foraster C, González-Cantero Á, Carrascosa Carrillo JM. Therapeutic goals and treatment response evaluation in moderate to severe psoriasis: an experts opinion document. Ann Med. 2021 Jan;53(1):1727–36.

3.	Galluzzo M, Talamonti M, Manfreda V, Silvaggio D, Tartaglia C, Roberto D, et al. Impact of secukinumab on patient-reported outcomes in moderate to severe plaque psoriasis: a review of clinical studies. Expert Opin Biol Ther. 2021 Feb 1;21(2):191–200.

4.	Rusk AM, Fleischer AB. In psoriasis treatment, greater improvement in skin severity predicts greater improvement in nail severity. J Dermatol Treat. 2021 Nov 17;32(8):894–7.

5.	Belinchon Romero I, Mateu Puchades A, Ribera Pibernat M, Ruiz Genao DianaP, De La Cueva Dobao P, Carrascosa JM. Criteria used to define tumor necrosis factor-alpha inhibitors failure in patients with moderate-to-severe psoriasis: a systematic literature review. Ann Med. 2023 Dec 12;55(1):1335–45.

6.	Gisondi P, Di Mercurio M, Idolazzi L, Girolomoni G. Concept of Remission in Chronic Plaque Psoriasis. J Rheumatol Suppl. 2015 Nov 1;93(0):57–60.

7.	Puig L. PASI 90 response: the new standard in therapeutic efficacy for psoriasis. J Eur Acad Dermatol Venereol. 2015 Apr;29(4):645–8.

8.	Boswell ND, Singla S, Gordon KB. Sequencing of Targeted Therapy in Psoriasis: Does it Matter? Am J Clin Dermatol. 2024 Sept;25(5):795–810.

9.	Thaçi D, Piaserico S, Gaarn Du Jardin K, Warren RB. Long‐term stability of PASI <3 response to tildrakizumab: Pooled analyses from reSURFACE 1 and reSURFACE 2 phase 3 trials through 5 years. J Eur Acad Dermatol Venereol [Internet]. 2023 Apr [cited 2024 Oct 4];37(4). Available from: https://onlinelibrary.wiley.com/doi/10.1111/jdv.18783

10.	Imafuku S, Ohata C, Okubo Y, Tobita R, Saeki H, Mabuchi T, et al. Effectiveness of brodalumab in achieving treatment satisfaction for patients with plaque psoriasis: The ProLOGUE study. J Dermatol Sci. 2022 Mar;105(3):176–84.

11.	Reich K, Mrowietz U, Menter A, Griffiths CEM, Bagel J, Strober B, et al. Effect of baseline disease severity on achievement of treatment target with apremilast: results from a pooled analysis. J Eur Acad Dermatol Venereol. 2021 Dec;35(12):2409–14.

12.	Campanati A, Diotallevi F, Radi G, Molinelli E, Brisigotti V, Martina E, et al. Psoriatic patients treated with secukinumab reach high levels of minimal disease activity: results from the SUPREME study. Eur J Dermatol. 2021 Oct;31(5):630–7.

13.	Lebwohl MG, Soliman AM, Yang H, Wang J, Koenigsberg SH, Anderson A, et al. 25572 Impact of risankizumab on the duration of PASI90 achievement: A post hoc analysis of 4 phase III clinical trials. J Am Acad Dermatol. 2021 Sept;85(3):AB62.

14.	Puig L, Zhu B, Burge R, Shrom D, Dong Y, Shen W, et al. Early Treatment Targets for Predicting Long-term Dermatology Life Quality Index Response in Patients with Moderate-to-Severe Plaque Psoriasis: A Post-hoc Analysis from a Long-term Clinical Study. J Clin Aesthetic Dermatol. 2020 Oct;13(10):18–22.

15.	Puig L, Dossenbach M, Berggren L, Ljungberg A, Zachariae C. Absolute and Relative Psoriasis Area and Severity Indices (PASI) for Comparison of the Efficacy of Ixekizumab to Etanercept and Placebo in Patients with Moderate-to-severe Plaque Psoriasis: An Integrated Analysis of UNCOVER-2 and UNCOVER-3 Outcomes. Acta Derm Venereol. 2019;99(11):971–7.

16.	Merola JF, Amato DA, See K, Burge R, Mallinckrodt C, Ojeh CK, et al. Evaluation of sPGA × BSA as an Outcome Measure and Treatment Target for Clinical Practice. J Invest Dermatol. 2018 Sept;138(9):1955–61.

17.	Elewski BE, Puig L, Mordin M, Gilloteau I, Sherif B, Fox T, et al. Psoriasis patients with psoriasis Area and Severity Index (PASI) 90 response achieve greater health-related quality-of-life improvements than those with PASI 75–89 response: results from two phase 3 studies of secukinumab. J Dermatol Treat. 2017 Aug 18;28(6):492–9.

18.	Schmitt J, Rosumeck S, Thomaschewski G, Sporbeck B, Haufe E, Nast A. Efficacy and safety of systemic treatments for moderate-to-severe psoriasis: meta-analysis of randomized controlled trials. Br J Dermatol. 2014 Feb;170(2):274–303.

19.	Li Z, Pei B, Lei T, Yao M, Niu Y, Zhou P, et al. Clinical features and effects of cyclosporine in the treatment of psoriasis: a systemic review and meta-analysis. Arch Dermatol Res. 2024 Oct 26;316(10):705.

20.	Kogan N, Raimondo N, Gusis SE, Izcovich A, Abarca Duran JA, Barahona‐Torres L, et al. Latin American clinical practice guidelines on the systemic treatment of psoriasis SOLAPSO–Sociedad Latinoamericana de Psoriasis (Latin American Psoriasis Society). Int J Dermatol. 2019;58:4–28.

21.	van Huizen AM, Sikkel R, Caron AG, Menting SP, Spuls PI. Methotrexate dosing regimen for plaque-type psoriasis: an update of a systematic review. J Dermatol Treat. 2022;33(8):3104–18.

22.	Hsieh TS, Tsai TF. Combination therapy for psoriasis with methotrexate and other oral disease-modifying antirheumatic drugs: a systematic review. Dermatol Ther. 2023;13(4):891–909.

23.	Cheng Y, Liu GJ, Zhang M. Narrow‐band ultraviolet B phototherapy versus broad‐band ultraviolet B or psoralen‐ultraviolet A photochemotherapy for psoriasis. Cochrane Database Syst Rev. 2013;(10):CD009481.

24.	Sbidian E, Chaimani A, Garcia-Doval I, Doney L, Dressler C, Hua C, et al. Systemic pharmacological treatments for chronic plaque psoriasis: a network meta-analysis. Cochrane Database Syst Rev. 2022 May 23;5(5):CD011535.

25.	Mahmoud A, Ahmed AAS, Naeem A, Abuelazm M, Elshinawy M, Hassan AR, et al. Tyrosine Kinase-2 Inhibitor (Deucravacitinib) for Psoriasis: A Systematic Review and Network Meta-Analysis of Randomized Controlled Trials. Int J Dermatol Venereol. 2024 Dec;7(4):216.

26.	Armstrong AW, Soliman AM, Betts KA, Wang Y, Gao Y, Stakias V, et al. Long-term benefit–risk profiles of treatments for moderate-to-severe plaque psoriasis: a network meta-analysis. Dermatol Ther. 2022;12(1):167–84.

27.	Piragine E, Petri D, Martelli A, Janowska A, Dini V, Romanelli M, et al. Adherence and persistence to biological drugs for psoriasis: systematic review with meta-analysis. J Clin Med. 2022;11(6):1506.

28.	Gottlieb AB, Kalb RE, Blauvelt A, Heffernan MP, Sofen HL, Ferris LK, et al. The efficacy and safety of infliximab in patients with plaque psoriasis who had an inadequate response to etanercept: results of a prospective, multicenter, open-label study. J Am Acad Dermatol. 2012 Oct;67(4):642–50.

29.	Yamauchi PS, Bissonnette R, Teixeira HD, Valdecantos WC. Systematic review of efficacy of anti-tumor necrosis factor (TNF) therapy in patients with psoriasis previously treated with a different anti-TNF agent. J Am Acad Dermatol. 2016 Sept;75(3):612-618.e6.

30.	Piaserico S, Cazzaniga S, Chimenti S, Giannetti A, Maccarone M, Picardo M, et al. Efficacy of switching between tumor necrosis factor-alfa inhibitors in psoriasis: results from the Italian Psocare registry. J Am Acad Dermatol. 2014 Feb;70(2):257-262.e3.

31.	Boswell ND, Singla S, Gordon KB. Sequencing of Targeted Therapy in Psoriasis: Does it Matter? Am J Clin Dermatol. 2024 Sept;25(5):795–810.

32.	Warren RB, Blauvelt A, Poulin Y, Beeck S, Kelly M, Wu T, et al. Efficacy and safety of risankizumab vs. secukinumab in patients with moderate-to-severe plaque psoriasis (IMMerge): results from a phase III, randomized, open-label, efficacy-assessor-blinded clinical trial. Br J Dermatol. 2021 Jan;184(1):50–9.

33.	Blauvelt A, Papp KA, Griffiths CEM, Randazzo B, Wasfi Y, Shen YK, et al. Efficacy and safety of guselkumab, an anti-interleukin-23 monoclonal antibody, compared with adalimumab for the continuous treatment of patients with moderate to severe psoriasis: Results from the phase III, double-blinded, placebo- and active comparator-controlled VOYAGE 1 trial. J Am Acad Dermatol. 2017 Mar;76(3):405–17.

34.	Reich K, Armstrong AW, Foley P, Song M, Wasfi Y, Randazzo B, et al. Efficacy and safety of guselkumab, an anti-interleukin-23 monoclonal antibody, compared with adalimumab for the treatment of patients with moderate to severe psoriasis with randomized withdrawal and retreatment: Results from the phase III, double-blind, placebo- and active comparator-controlled VOYAGE 2 trial. J Am Acad Dermatol. 2017 Mar;76(3):418–31.

35.	Reich K, Gooderham M, Thaçi D, Crowley JJ, Ryan C, Krueger JG, et al. Risankizumab compared with adalimumab in patients with moderate-to-severe plaque psoriasis (IMMvent): a randomised, double-blind, active-comparator-controlled phase 3 trial. Lancet Lond Engl. 2019 Aug 17;394(10198):576–86.

36.	Warren RB, Blauvelt A, Bagel J, Papp KA, Yamauchi P, Armstrong A, et al. Bimekizumab versus Adalimumab in Plaque Psoriasis. N Engl J Med. 2021 July 8;385(2):130–41.

37.	Warren RB, Barker JNWB, Finlay AY, Burden AD, Kirby B, Armendariz Y, et al. Secukinumab for patients failing previous tumour necrosis factor-α inhibitor therapy: results of a randomized open-label study (SIGNATURE). Br J Dermatol. 2020 July;183(1):60–70.

38.	Strober B, Paul C, Blauvelt A, Thaçi D, Puig L, Lebwohl M, et al. Bimekizumab efficacy and safety in patients with moderate to severe plaque psoriasis: Two-year interim results from the open-label extension of the randomized BE RADIANT phase 3b trial. J Am Acad Dermatol. 2023 Sept;89(3):486–95.

39.	Loft N, Halling AS, Egeberg A, Skov L. Efficacy of a second interleukin 17 inhibitor in patients with psoriasis: A systematic review and meta-analysis. J Am Acad Dermatol. 2021 Jan;84(1):130–8.

40.	Loft N, Egeberg A, Isufi D, Rasmussen MK, Bryld LE, Dam TN, et al. Response to Interleukin-17A Inhibitors According to Prior Biologic Exposures: A Danish Nationwide Study. Acta Derm Venereol. 2023 Nov 21;103:adv12616.

41.	Papp K, Blauvelt A, Sullivan J, Tada Y, Polzer P, Mallbris L, et al. Ixekizumab—An Effective and Safe Treatment for Moderate-to-Severe Plaque Psoriasis in Patients Previously Treated With Other IL-17 Inhibitors: Results From IXORA-P. J Psoriasis Psoriatic Arthritis®. 2019 Oct 1;4(4):180–5.

42.	Reddy R, Pannu S, Fiumara K, Kahn J, Rosmarin D. Efficacy of in-class interleukin-23 inhibitor switching: risankizumab following guselkumab failure in moderate-to-severe psoriasis treatment. Br J Dermatol. 2021 Mar;184(3):559–61.

43.	Gasslitter I, Kirsten N, Augustin M, Torz K, Mrowietz U, Eyerich K, et al. Successful intra-class switching among IL-17 antagonists: a multicentre, multinational, retrospective study. Arch Dermatol Res. 2019 July;311(5):421–4.

44.	Daniele SG, Eldirany SA, Damiani G, Ho M, Bunick CG. Structural Basis for p19 Targeting by Anti-IL-23 Biologics: Correlations with Short- and Long-Term Efficacy in Psoriasis. JID Innov Skin Sci Mol Popul Health. 2024 Mar;4(2):100261.

45.	Camela E, Ocampo‐Garza SS, Cinelli E, Villani A, Fabbrocini G, Megna M. Therapeutic update of biologics and small molecules for scalp psoriasis: a systematic review. Dermatol Ther. 2021;34(2):e14857.

46.	Mosca M, Hong J, Hadeler E, Brownstone N, Bhutani T, Liao W. Scalp psoriasis: a literature review of effective therapies and updated recommendations for practical management. Dermatol Ther. 2021;11(3):769–97.

47.	Tsiogkas SG, Karamitrou EK, Grammatikopoulou MG, Zafiriou E, Bogdanos DP. Efficacy of tyrosine-kinase-2 and phosphodiesterase-4 inhibitors for scalp psoriasis: a systematic review and meta-analysis. Curr Med Res Opin. 2024;40(2):155–63.

48.	Duffin KC, Griffiths CE, Hoffmann M, Blauvelt A, Balagula E, Napoli A, et al. Deucravacitinib, an Oral Selective Tyrosine Kinase 2 (TYK2) Inhibitor, in Patients with Moderate to Severe Scalp Psoriasis: Efficacy and Safety Results of a Phase 3b/4, Multicenter, Randomized, Double-blinded, Placebo-controlled Trial (PSORIATYK SCALP). SKIN J Cutan Med. 2024;8(6):s420–s420.

49.	Khan M, Wallace CE, Ahmed F, Rahman SM, Memon N, Haque A. Assessing Comparative Efficacy of Biologics for the Treatment of Psoriasis With Nail Involvement: A Systematic Review. J Psoriasis Psoriatic Arthritis®. 2024;9(2):61–8.

50.	Hwang JK, Ricardo JW, Lipner SR. Efficacy and safety of nail psoriasis targeted therapies: a systematic review. Am J Clin Dermatol. 2023;24(5):695–720.

51.	Hong JJ, Mosca ML, Hadeler EK, Brownstone ND, Bhutani T, Liao WJ. Genital and inverse/intertriginous psoriasis: an updated review of therapies and recommendations for practical management. Dermatol Ther. 2021;11(3):833–44.

52.	Beck KM, Yang EJ, Sanchez IM, Liao W. Treatment of Genital Psoriasis: A Systematic Review. Dermatol Ther. 2018 Dec;8(4):509–25.

53.	Tsiogkas SG, Grammatikopoulou MG, Kontouli KM, Minopoulou I, Goulis DD, Zafiriou E, et al. Efficacy of biologic agents for palmoplantar psoriasis: a systematic review and network meta-analysis. Expert Rev Clin Immunol. 2023;19(12):1485–98.

54.	Huang IH, Wu PC, Chiu HY, Huang YH. Small-molecule inhibitors and biologics for palmoplantar psoriasis and palmoplantar pustulosis: a systematic review and network meta-analysis. Am J Clin Dermatol. 2024;25(3):347–58.

55.	Xia R, Diao Z, Chen D, Wang Y, Zhou C, Gao Y, et al. Efficacy of anti-interleukin-17A biological agents for palmoplantar psoriasis and palmoplantar pustulosis: A network meta-analysis. Int Immunopharmacol. 2024;130:111716.

56.	Reynolds KA, Pithadia DJ, Lee EB, Liao W, Wu JJ. A systematic review of treatment strategies for erythrodermic psoriasis. J Dermatol Treat. 2021 Feb;32(1):49–55.

57.	Lo Y, Tsai TF. Updates on the treatment of erythrodermic psoriasis. Psoriasis Targets Ther. 2021;59–73.

58.	Zhou T, Koussiouris J, Kim L, Vender R. Management of guttate psoriasis: a systematic review. J Cutan Med Surg. 2024;28(6):577–84.

59.	Rega F, Trovato F, Bortone G, Pellacani G, Richetta AG, Dattola A. Therapeutic potential of Spesolimab-Sbzo in the management of generalized pustular psoriasis flares in adults: evidence to date. Psoriasis Targets Ther. 2024;23–7.

60.	Puig L, Fujita H, Thaçi D, Zheng M, Hernandez Daly AC, Leonardi C, et al. Current treatments for generalized pustular psoriasis: a narrative summary of a systematic literature search. Dermatol Ther. 2024;14(9):2331–78.

61.	Chen B lin, Liu Q wei, Dong X wan, Bai Y ping. Biologics for generalized pustular psoriasis: a systematic review and single-arm meta-analysis. Front Immunol. 2024;15:1462158.

62.	Kearns DG, Chat VS, Zang PD, Han G, Wu JJ. Review of treatments for generalized pustular psoriasis. J Dermatol Treat. 2021 July 4;32(5):492–4.

63.	Sánchez‐García V, Hernández‐Quiles R, de‐Miguel‐Balsa E, Giménez‐Richarte Á, Ramos‐Rincón J, Belinchón‐Romero I. Exposure to biologic therapy before and during pregnancy in patients with psoriasis: systematic review and meta‐analysis. J Eur Acad Dermatol Venereol. 2023;37(10):1971–90.

64.	Owczarek W, Walecka I, Lesiak A, Czajkowski R, Reich A, Zerda I, et al. The use of biological drugs in psoriasis patients prior to pregnancy, during pregnancy and lactation: a review of current clinical guidelines. Adv Dermatol Allergol Dermatol Alergol. 2020;37(6):821–30.

65.	Jeong YD, Jo H, Cho H, Jang W, Park J, Lee S, et al. Biologics use for psoriasis during pregnancy and its related adverse outcomes in pregnant women and newborns: Findings from WHO pharmacovigilance study. Int Arch Allergy Immunol. 2025;186(6):579–93.

66.	Battista T, Gallo L, Martora F, Fattore D, Potestio L, Cacciapuoti S, et al. Biological therapy for psoriasis in cancer patients: an 8-year retrospective real-life study. J Clin Med. 2024;13(7):1940.

67.	Gupta A, Peyrin-Biroulet L, Ananthakrishnan AN. Risk of cancer recurrence in patients with immune-mediated diseases with use of immunosuppressive therapies: an updated systematic review and meta-analysis. Clin Gastroenterol Hepatol. 2024;22(3):499–512.

68.	Russell MD, Stovin C, Alveyn E, Adeyemi O, Chan CKD, Patel V, et al. JAK inhibitors and the risk of malignancy: a meta-analysis across disease indications. Ann Rheum Dis. 2023;82(8):1059–67.

69.	Alhalabi MM, Almokdad R. The incidence of hepatitis B reactivation in patients receiving ustekinumab: a systematic review and proportional meta-analysis. Eur J Gastroenterol Hepatol. 2025;37(1):1–9.

70.	Kuo MH, Ko PH, Wang ST, Tseng CW. Incidence of HBV Reactivation in Psoriasis Patients Undergoing Cytokine Inhibitor Therapy: A Single-Center Study and Systematic Review with a Meta-Analysis. Viruses. 2024;17(1):42.

71.	Li L, Jiang X, Fu L, Zhang L, Feng Y. Reactivation rates of hepatitis B or C or HIV in patients with psoriasis using biological therapies: a systematic review and meta-analysis. Clin Exp Med. 2023;23(3):701–15.

72.	Megna M, Lauletta G, Tommasino N, Salsano A, Battista T, Ruggiero A, et al. Management of psoriasis patients with serious infectious diseases. Adv Ther. 2024;41(6):2099–111.

73.	Zhu X, Pan X, Da M, Wang F, Dong Z. Risk for latent tuberculosis infection reactivation among patients with psoriasis on biologics treatment: a meta-analysis. J Infect. 2024;89(3).

74.	Mateu-Arrom L, Puig L. Infection Risk and Vaccination in the Management of Psoriasis: Considerations for Biologic Therapy. Psoriasis Targets Ther. 2025;127–44.

75.	Jafarzadeh A, Behrangi E, Khosravi M, Falakeh S, Amiri JK, Goodarzi A. Emerging treatments for dermatologic diseases in infants, children, and adolescents: a systematic review of clinical trials on biologics and small molecule inhibitors. Inflammopharmacology. 2025;1–56.

76.	Sticherling M, Nikkels AF, Hamza AM, Kwong P, Szepietowski JC, El Sayed M, et al. Secukinumab in pediatric patients with plaque psoriasis: pooled safety analysis from two phase 3 randomized clinical trials. Am J Clin Dermatol. 2023;24(5):821–35.

77.	Li C, Sunhe Y, Zhou H, Dong W. Efficacy and safety evaluations of adalimumab biosimilars in the treatment of psoriasis. J Dermatol Treat. 2023;34(1):2249145.

78.	Phan DB, Elyoussfi S, Stevenson M, Lunt M, Warren RB, Yiu ZZ. Biosimilars for the treatment of psoriasis: a systematic review of clinical trials and observational studies. JAMA Dermatol. 2023;159(7):763–71.

79.	Macaluso FS, Cummings JF, Atreya R, Choi J, Orlando A. A systematic review on infliximab biosimilar SB2: from pre-clinical data to real-world evidence. Expert Opin Biol Ther. 2022;22(2):203–23.

80.	Wang Q, Wang J, Sun X, Liu L, Zhang M, Yu Y, et al. Evidence-based dietary recommendations for patients with psoriasis: A systematic review. Clin Nutr. 2025;47:68–82.

81.	Wei K, Liao X, Yang T, He X, Yang D, Lai L, et al. Efficacy of probiotic supplementation in the treatment of psoriasis—A systematic review and meta‐analysis. J Cosmet Dermatol. 2024;23(7):2361–7.

82.	Lu L, Xu Y, Shi M, Liu A. Psychosocial interventions for psoriasis: a Bayesian network meta-analysis. J Dermatol Treat. 2025;36(1):2427321.

83.	Wei W, Zhang B, Liu T, Lu T. Effect of psychological intervention on quality of life among patients with psoriasis: a meta-analysis. Int J Behav Med. 2024;31(6):911–22.

## QUESTION 11. What are the efficacy and safety of switching between anti-TNF agents after treatment failure?

[26–28]. Systematic reviews and registry data show PASI75 response rates of 40% to 60%, mainly in secondary loss of response. These rates are lower than those achieved with IL-17 or IL-23 inhibitors, so within-class anti-TNF switching is generally considered a second-line alternative when a class switch is not feasible in primary failure[29]. o relevant increases in serious adverse events have been reported, and overall tolerability remains acceptable, although limited durability of response constrains this strategy.

**Efficacy and safety of switching from etanercept to infliximab in psoriasis (PSUNRISE, Gottlieb et al., 2012)****[26]**

| **Outcome** | **Population / Follow-up** | **Study type** | **Certainty of evidence (GRADE)** | **Main result** | **Clinical comment / Interpretation** |
| --- | --- | --- | --- | --- | --- |
| Efficacy (PASI 75) | 211 patients, 26 weeks | Open-label, multicenter, prospective study | Low ⨁⨁◯◯ | 51.7% (60/211) at week 10; 54.5% at week 26 | Clinically meaningful improvement in >50% of patients |
| Physician global response (PGA 0/1) | 211 (week 10), 199 (week 26) | — | Moderate ⨁⨁⨁◯ | 65.4% at week 10 (95% CI 58.6–71.8); 61.3% at week 26 | Sustained response in roughly two-thirds of patients |
| Safety (serious adverse events) | 215 | Prospective observational | Moderate ⨁⨁⨁◯ | 3.7% (8 patients) | No increase in risk compared with primary infliximab use |

**Efficacy and safety of switching between anti-TNF agents after treatment failure, based on the Psocare registry (Piaserico et al., 2014)****[28]**

| **Outcome** | **Population / Follow-up** | **Study type** | **Certainty of evidence (GRADE)** | **Main result** | **Clinical comment / Interpretation** |
| --- | --- | --- | --- | --- | --- |
| Efficacy (PASI 75) | >2,000 patients switching anti-TNF agents | Prospective observational | Low ⨁⨁◯◯ | 40%–50% achieved PASI 75 after switching | Clinically meaningful improvement |
| Safety (serious adverse events) | Not stratified by switch type | Prospective observational | Moderate ⨁⨁⨁◯ | No meaningful increase | Acceptable safety profile maintained |

## QUESTION 12. What are the efficacy and safety of switching from an anti-TNF agent to an IL-17 or IL-23 inhibitor after treatment failure?

[30–33] shows that switching from an anti-TNF agent to an IL-17 or IL-23 inhibitor is highly effective in patients with moderate-to-severe plaque psoriasis with prior treatment failure. Phase III trials report higher clinical response rates after switching, including among patients with inadequate response to adalimumab. Trials such as VOYAGE, IMMvent, and IMMerge show clinically relevant gains in PASI90 after switching to guselkumab or risankizumab, while data from SIGNATURE and BE SURE with secukinumab and bimekizumab support the effectiveness of IL-17 inhibition[34,35]. This approach is prioritized for both primary and secondary anti-TNF failure given higher efficacy and a favorable safety profile[29].

**Efficacy and safety of guselkumab after adalimumab failure (VOYAGE 2)****[32]**

| **Outcome** | **Population / Follow-up** | **Study type** | **Certainty of evidence (GRADE)** | **Main result** | **Clinical comment / Interpretation** |
| --- | --- | --- | --- | --- | --- |
| Efficacy (PASI 90 at week 48) in adalimumab nonresponders who switched to guselkumab | 112 (VOYAGE 2 subgroup) / 20 weeks post-switch (week 28 to 48) | Randomized trial (subgroup without re-randomization) | Moderate ⨁⨁⨁◯ | Absolute response 66.1% | 66.1% achieved PASI 90 at week 48 after switching from adalimumab to guselkumab |
| Efficacy (PASI 100 at week 48) | 112 / 20 weeks | — | Moderate ⨁⨁⨁◯ | Absolute response 28.6% | 28.6% achieved complete clearance (PASI 100) |
| Safety (serious adverse events) | 112 / 20 weeks | Trial with active safety monitoring | Moderate ⨁⨁⨁◯ | No serious or unexpected treatment-related SAEs reported in the subgroup | Low adverse event rate; no SAEs, deaths, or serious infections |

**Efficacy and safety of risankizumab after partial response to adalimumab (IMMvent)****[33]**

| **Outcome** | **Population / Follow-up** | **Study type** | **Certainty of evidence (GRADE)** | **Main result** | **Clinical comment / Interpretation** |
| --- | --- | --- | --- | --- | --- |
| Efficacy (PASI 90 at week 44) in intermediate responders to adalimumab who switched to risankizumab | 109 (IMMvent, Part B) / 28 weeks (week 16 to 44) | Randomized, double-blind trial | High ⨁⨁⨁⨁ | RR not reported; adjusted absolute difference 45.0% (95% CI: 28.9 to 61.1) | 66% with risankizumab vs 21% continuing adalimumab |
| Efficacy (PASI 100 at week 44) | 109 / 28 weeks | — | High ⨁⨁⨁⨁ | Adjusted absolute difference 32.8% (95% CI: 18.8 to 46.9) | 40% with risankizumab vs 7% with adalimumab |
| Safety (serious adverse events) | 109 / 28 weeks | Trial with active safety monitoring | High ⨁⨁⨁⨁ | No relevant differences reported | SAEs in 6% (risankizumab) vs 4% (adalimumab); no new safety profile differences |

**Efficacy and safety of secukinumab after anti-TNF failure (SIGNATURE)****[35]**

| **Outcome** | **Population / Follow-up** | **Study type** | **Certainty of evidence (GRADE)** | **Main result** | **Clinical comment / Interpretation** |
| --- | --- | --- | --- | --- | --- |
| Efficacy (PASI 75 at week 16) | 233 patients with prior anti-TNF failure / 16 weeks | Open-label, randomized, noncomparative trial | Moderate ⨁⨁⨁◯ | 65.3% (300 mg) and 44.3% (150 mg) achieved PASI 75 (P < 0.0001) | Clinically meaningful response after mechanism switch; higher efficacy with 300 mg |
| Efficacy (PASI 75 at week 72) | 70 patients (subgroup remaining on 300 mg) / 72 weeks | Open-label trial | Moderate ⨁⨁⨁◯ | 77% maintained PASI 75 | Durable long-term effect, consistent with sustained response after switching to IL-17 inhibition |
| Safety (serious adverse events) | 233 patients / 72 weeks | Trial with active safety monitoring | Moderate ⨁⨁⨁◯ | Adverse events consistent with known profile; higher frequency of candidiasis | No new safety signals. Acceptable safety. Some increase in mild Candida infections. |

## QUESTION 13. What is the efficacy of switching within the same biologic class (IL-17 or IL-23) after treatment failure?

Within-class switching, particularly after secondary failure, is supported by observational evidence and open-label trials. For IL-17 inhibitors, switching among agents such as secukinumab, ixekizumab, or bimekizumab can restore clinical response, with PASI90 rates above 70% in several studies[36–39]. For IL-23 inhibitors, evidence is more limited and responses are variable, with better outcomes among patients without prior primary failure[40]. In this setting, within-class switching can be considered after secondary failure before switching classes, with the goal of optimizing and preserving future therapeutic options, while emphasizing careful patient selection and close follow-up[29].

**Efficacy and safety of bimekizumab after switching from secukinumab (BE RADIANT)****[36]**

| **Outcome** | **Population / Follow-up** | **Study type** | **Certainty of evidence (GRADE)** | **Main result** | **Clinical comment / Interpretation** |
| --- | --- | --- | --- | --- | --- |
| Efficacy (PASI 100 at week 96) in patients who switched from secukinumab to bimekizumab | BE RADIANT subgroup / 48 weeks post-switch (week 48 to 96) | Randomized trial (with open-label extension) | Moderate ⨁⨁⨁◯ | PASI 100 in 76.6% at end of follow-up | After switching from secukinumab to bimekizumab, complete clearance was regained in nearly 8 of 10 patients |
| Efficacy (PASI 100 at week 48) in patients treated with bimekizumab from baseline | 373 patients / 48 weeks | Randomized, double-blind trial | High ⨁⨁⨁⨁ | 74.8% achieved PASI 100 vs 52.8% with secukinumab | Bimekizumab showed superiority versus secukinumab during the first treatment year |
| Safety (common and serious adverse events) | Entire cohort / through week 96 | Trial with active safety monitoring | High ⨁⨁⨁⨁ | Common events: nasopharyngitis, oral candidiasis, UTI; no new safety signals | Safety profile consistent with prior studies; candidiasis more frequent but manageable |

**Efficacy and safety of ixekizumab after prior exposure to another IL-17 inhibitor (IXORA-P)****[39]**

| **Outcome** | **Population / Follow-up** | **Study type** | **Certainty of evidence (GRADE)** | **Main result** | **Clinical comment / Interpretation** |
| --- | --- | --- | --- | --- | --- |
| Efficacy (PASI 90 at week 52) in patients previously exposed to another IL-17 agent | Subgroup of 288 patients (IL-17–experienced) / 52 weeks | Randomized, double-blind trial | Moderate ⨁⨁⨁◯ | PASI 90 in 82% (Q2W), 67% (Q4W), 75% (Q4W/Q2W adjustment) | Prior IL-17 exposure did not reduce ixekizumab effectiveness; outcomes similar to IL-17–naïve patients |
| Efficacy (PASI 100 at week 52) in patients previously exposed | 288 / 52 weeks | Randomized, double-blind trial | Moderate ⨁⨁⨁◯ | PASI 100 in 59% (Q2W), 42% (Q4W), 52% (Q4W/Q2W) | Sustained complete response in IL-17–experienced patients, comparable with IL-17–naïve |
| Safety (common and serious adverse events) | Entire cohort / 52 weeks | Trial with active safety monitoring | Moderate ⨁⨁⨁◯ | Similar safety profile in IL-17–naïve vs previously treated patients | No new safety signals; consistent with the known ixekizumab profile |

**Efficacy and safety of switching from guselkumab to risankizumab in moderate-to-severe psoriasis (Reddy et al., 2021)****[40]**

| **Outcome** | **Population / Follow-up** | **Study design** | **Certainty of evidence (GRADE)** | **Main result** | **Clinical comment / Interpretation** |
| --- | --- | --- | --- | --- | --- |
| Mean reduction in PGA×BSA among patients who switched from guselkumab to risankizumab | 23 patients / mean 5.4 months on risankizumab | Retrospective observational study | Low ⨁⨁◯◯ | Mean 79.3% reduction in PGA×BSA (28.5 → 5.9) | Clinically meaningful improvement after switching, with better response in secondary failure (90% reduction) than in primary failure (57.6%). |
| Comparison with IL-23–naïve patients | 18 guselkumab-naïve patients / 5.4 months on risankizumab | Comparative observational study | Low ⨁⨁◯◯ | 84.3% reduction in PGA×BSA (49.8 → 7.8) | Similar response to those who switched from guselkumab, suggesting preserved effectiveness after an in-class switch. |
| Safety (common and serious adverse events) | 41 patients total / short mean follow-up | Observational chart review | Low ⨁⨁◯◯ | No related serious adverse events reported | Safety consistent with the expected risankizumab profile, without new clinically relevant findings. |

## QUESTION 14. What are the recommendations for optimal biologic sequencing based on failure type (primary vs secondary)?

Management of adults with moderate-to-severe plaque psoriasis who have had prior biologic failure requires an individualized approach driven by the pattern of treatment failure. Available evidence indicates that switching between anti-TNF agents can be useful mainly in secondary failure, with PASI75 response rates of 40%–60%, but with lower effectiveness than switching to a different biologic class; in primary failure, a class switch is generally recommended[26–28].

Switching from an anti-TNF agent to an IL-17 or IL-23 inhibitor shows consistently higher effectiveness, with high PASI90 rates even among patients previously exposed to biologics [32]^,^^[29,34]^. In-class switching—particularly among IL-17 inhibitors and in the setting of secondary loss of response—can restore disease control, with PASI90 response rates around 70%–80% in selected patients [36,39–41], For IL-23 inhibitors, outcomes after in-class switching are more variable and appear to depend on the type of prior failure.

According to Boswell et al., primary failure should prompt a switch in mechanism of action, whereas secondary failure allows consideration of an in-class switch first to preserve future options [29]. Concomitant psoriatic arthritis also influences sequencing, generally favoring IL-17 inhibitors for axial involvement and IL-23 inhibitors for predominantly peripheral disease. Structural differences among agents within the same class may help explain variability in response and support a more personalized approach when considering in-class switching[42].

Figure 1. Optimal sequencing algorithm for biologic therapies in psoriasis by type of treatment failure (adapted from Boswell et al.) [29].

**References**

1.	Yasmeen N, Sawyer LM, Malottki K, Levin LÅ, Didriksen Apol E, Jemec GB. Targeted therapies for patients with moderate-to-severe psoriasis: a systematic review and network meta-analysis of PASI response at 1 year. J Dermatol Treat. 2022 Jan 2;33(1):204–18.

2.	Belinchón Romero I, Dauden E, Ferrándiz Foraster C, González-Cantero Á, Carrascosa Carrillo JM. Therapeutic goals and treatment response evaluation in moderate to severe psoriasis: an experts opinion document. Ann Med. 2021 Jan;53(1):1727–36.

3.	Galluzzo M, Talamonti M, Manfreda V, Silvaggio D, Tartaglia C, Roberto D, et al. Impact of secukinumab on patient-reported outcomes in moderate to severe plaque psoriasis: a review of clinical studies. Expert Opin Biol Ther. 2021 Feb 1;21(2):191–200.

4.	Rusk AM, Fleischer AB. In psoriasis treatment, greater improvement in skin severity predicts greater improvement in nail severity. J Dermatol Treat. 2021 Nov 17;32(8):894–7.

5.	Belinchon Romero I, Mateu Puchades A, Ribera Pibernat M, Ruiz Genao DianaP, De La Cueva Dobao P, Carrascosa JM. Criteria used to define tumor necrosis factor-alpha inhibitors failure in patients with moderate-to-severe psoriasis: a systematic literature review. Ann Med. 2023 Dec 12;55(1):1335–45.

6.	Gisondi P, Di Mercurio M, Idolazzi L, Girolomoni G. Concept of Remission in Chronic Plaque Psoriasis. J Rheumatol Suppl. 2015 Nov 1;93(0):57–60.

7.	Puig L. PASI 90 response: the new standard in therapeutic efficacy for psoriasis. J Eur Acad Dermatol Venereol. 2015 Apr;29(4):645–8.

8.	Boswell ND, Singla S, Gordon KB. Sequencing of Targeted Therapy in Psoriasis: Does it Matter? Am J Clin Dermatol. 2024 Sept;25(5):795–810.

9.	Thaçi D, Piaserico S, Gaarn Du Jardin K, Warren RB. Long‐term stability of PASI <3 response to tildrakizumab: Pooled analyses from reSURFACE 1 and reSURFACE 2 phase 3 trials through 5 years. J Eur Acad Dermatol Venereol [Internet]. 2023 Apr [cited 2024 Oct 4];37(4). Available from: https://onlinelibrary.wiley.com/doi/10.1111/jdv.18783

10.	Imafuku S, Ohata C, Okubo Y, Tobita R, Saeki H, Mabuchi T, et al. Effectiveness of brodalumab in achieving treatment satisfaction for patients with plaque psoriasis: The ProLOGUE study. J Dermatol Sci. 2022 Mar;105(3):176–84.

11.	Reich K, Mrowietz U, Menter A, Griffiths CEM, Bagel J, Strober B, et al. Effect of baseline disease severity on achievement of treatment target with apremilast: results from a pooled analysis. J Eur Acad Dermatol Venereol. 2021 Dec;35(12):2409–14.

12.	Campanati A, Diotallevi F, Radi G, Molinelli E, Brisigotti V, Martina E, et al. Psoriatic patients treated with secukinumab reach high levels of minimal disease activity: results from the SUPREME study. Eur J Dermatol. 2021 Oct;31(5):630–7.

13.	Lebwohl MG, Soliman AM, Yang H, Wang J, Koenigsberg SH, Anderson A, et al. 25572 Impact of risankizumab on the duration of PASI90 achievement: A post hoc analysis of 4 phase III clinical trials. J Am Acad Dermatol. 2021 Sept;85(3):AB62.

14.	Puig L, Zhu B, Burge R, Shrom D, Dong Y, Shen W, et al. Early Treatment Targets for Predicting Long-term Dermatology Life Quality Index Response in Patients with Moderate-to-Severe Plaque Psoriasis: A Post-hoc Analysis from a Long-term Clinical Study. J Clin Aesthetic Dermatol. 2020 Oct;13(10):18–22.

15.	Puig L, Dossenbach M, Berggren L, Ljungberg A, Zachariae C. Absolute and Relative Psoriasis Area and Severity Indices (PASI) for Comparison of the Efficacy of Ixekizumab to Etanercept and Placebo in Patients with Moderate-to-severe Plaque Psoriasis: An Integrated Analysis of UNCOVER-2 and UNCOVER-3 Outcomes. Acta Derm Venereol. 2019;99(11):971–7.

16.	Merola JF, Amato DA, See K, Burge R, Mallinckrodt C, Ojeh CK, et al. Evaluation of sPGA × BSA as an Outcome Measure and Treatment Target for Clinical Practice. J Invest Dermatol. 2018 Sept;138(9):1955–61.

17.	Elewski BE, Puig L, Mordin M, Gilloteau I, Sherif B, Fox T, et al. Psoriasis patients with psoriasis Area and Severity Index (PASI) 90 response achieve greater health-related quality-of-life improvements than those with PASI 75–89 response: results from two phase 3 studies of secukinumab. J Dermatol Treat. 2017 Aug 18;28(6):492–9.

18.	Schmitt J, Rosumeck S, Thomaschewski G, Sporbeck B, Haufe E, Nast A. Efficacy and safety of systemic treatments for moderate-to-severe psoriasis: meta-analysis of randomized controlled trials. Br J Dermatol. 2014 Feb;170(2):274–303.

19.	Li Z, Pei B, Lei T, Yao M, Niu Y, Zhou P, et al. Clinical features and effects of cyclosporine in the treatment of psoriasis: a systemic review and meta-analysis. Arch Dermatol Res. 2024 Oct 26;316(10):705.

20.	Kogan N, Raimondo N, Gusis SE, Izcovich A, Abarca Duran JA, Barahona‐Torres L, et al. Latin American clinical practice guidelines on the systemic treatment of psoriasis SOLAPSO–Sociedad Latinoamericana de Psoriasis (Latin American Psoriasis Society). Int J Dermatol. 2019;58:4–28.

21.	van Huizen AM, Sikkel R, Caron AG, Menting SP, Spuls PI. Methotrexate dosing regimen for plaque-type psoriasis: an update of a systematic review. J Dermatol Treat. 2022;33(8):3104–18.

22.	Hsieh TS, Tsai TF. Combination therapy for psoriasis with methotrexate and other oral disease-modifying antirheumatic drugs: a systematic review. Dermatol Ther. 2023;13(4):891–909.

23.	Cheng Y, Liu GJ, Zhang M. Narrow‐band ultraviolet B phototherapy versus broad‐band ultraviolet B or psoralen‐ultraviolet A photochemotherapy for psoriasis. Cochrane Database Syst Rev. 2013;(10):CD009481.

24.	Sbidian E, Chaimani A, Garcia-Doval I, Doney L, Dressler C, Hua C, et al. Systemic pharmacological treatments for chronic plaque psoriasis: a network meta-analysis. Cochrane Database Syst Rev. 2022 May 23;5(5):CD011535.

25.	Mahmoud A, Ahmed AAS, Naeem A, Abuelazm M, Elshinawy M, Hassan AR, et al. Tyrosine Kinase-2 Inhibitor (Deucravacitinib) for Psoriasis: A Systematic Review and Network Meta-Analysis of Randomized Controlled Trials. Int J Dermatol Venereol. 2024 Dec;7(4):216.

26.	Armstrong AW, Soliman AM, Betts KA, Wang Y, Gao Y, Stakias V, et al. Long-term benefit–risk profiles of treatments for moderate-to-severe plaque psoriasis: a network meta-analysis. Dermatol Ther. 2022;12(1):167–84.

27.	Piragine E, Petri D, Martelli A, Janowska A, Dini V, Romanelli M, et al. Adherence and persistence to biological drugs for psoriasis: systematic review with meta-analysis. J Clin Med. 2022;11(6):1506.

28.	Gottlieb AB, Kalb RE, Blauvelt A, Heffernan MP, Sofen HL, Ferris LK, et al. The efficacy and safety of infliximab in patients with plaque psoriasis who had an inadequate response to etanercept: results of a prospective, multicenter, open-label study. J Am Acad Dermatol. 2012 Oct;67(4):642–50.

29.	Yamauchi PS, Bissonnette R, Teixeira HD, Valdecantos WC. Systematic review of efficacy of anti-tumor necrosis factor (TNF) therapy in patients with psoriasis previously treated with a different anti-TNF agent. J Am Acad Dermatol. 2016 Sept;75(3):612-618.e6.

30.	Piaserico S, Cazzaniga S, Chimenti S, Giannetti A, Maccarone M, Picardo M, et al. Efficacy of switching between tumor necrosis factor-alfa inhibitors in psoriasis: results from the Italian Psocare registry. J Am Acad Dermatol. 2014 Feb;70(2):257-262.e3.

31.	Boswell ND, Singla S, Gordon KB. Sequencing of Targeted Therapy in Psoriasis: Does it Matter? Am J Clin Dermatol. 2024 Sept;25(5):795–810.

32.	Warren RB, Blauvelt A, Poulin Y, Beeck S, Kelly M, Wu T, et al. Efficacy and safety of risankizumab vs. secukinumab in patients with moderate-to-severe plaque psoriasis (IMMerge): results from a phase III, randomized, open-label, efficacy-assessor-blinded clinical trial. Br J Dermatol. 2021 Jan;184(1):50–9.

33.	Blauvelt A, Papp KA, Griffiths CEM, Randazzo B, Wasfi Y, Shen YK, et al. Efficacy and safety of guselkumab, an anti-interleukin-23 monoclonal antibody, compared with adalimumab for the continuous treatment of patients with moderate to severe psoriasis: Results from the phase III, double-blinded, placebo- and active comparator-controlled VOYAGE 1 trial. J Am Acad Dermatol. 2017 Mar;76(3):405–17.

34.	Reich K, Armstrong AW, Foley P, Song M, Wasfi Y, Randazzo B, et al. Efficacy and safety of guselkumab, an anti-interleukin-23 monoclonal antibody, compared with adalimumab for the treatment of patients with moderate to severe psoriasis with randomized withdrawal and retreatment: Results from the phase III, double-blind, placebo- and active comparator-controlled VOYAGE 2 trial. J Am Acad Dermatol. 2017 Mar;76(3):418–31.

35.	Reich K, Gooderham M, Thaçi D, Crowley JJ, Ryan C, Krueger JG, et al. Risankizumab compared with adalimumab in patients with moderate-to-severe plaque psoriasis (IMMvent): a randomised, double-blind, active-comparator-controlled phase 3 trial. Lancet Lond Engl. 2019 Aug 17;394(10198):576–86.

36.	Warren RB, Blauvelt A, Bagel J, Papp KA, Yamauchi P, Armstrong A, et al. Bimekizumab versus Adalimumab in Plaque Psoriasis. N Engl J Med. 2021 July 8;385(2):130–41.

37.	Warren RB, Barker JNWB, Finlay AY, Burden AD, Kirby B, Armendariz Y, et al. Secukinumab for patients failing previous tumour necrosis factor-α inhibitor therapy: results of a randomized open-label study (SIGNATURE). Br J Dermatol. 2020 July;183(1):60–70.

38.	Strober B, Paul C, Blauvelt A, Thaçi D, Puig L, Lebwohl M, et al. Bimekizumab efficacy and safety in patients with moderate to severe plaque psoriasis: Two-year interim results from the open-label extension of the randomized BE RADIANT phase 3b trial. J Am Acad Dermatol. 2023 Sept;89(3):486–95.

39.	Loft N, Halling AS, Egeberg A, Skov L. Efficacy of a second interleukin 17 inhibitor in patients with psoriasis: A systematic review and meta-analysis. J Am Acad Dermatol. 2021 Jan;84(1):130–8.

40.	Loft N, Egeberg A, Isufi D, Rasmussen MK, Bryld LE, Dam TN, et al. Response to Interleukin-17A Inhibitors According to Prior Biologic Exposures: A Danish Nationwide Study. Acta Derm Venereol. 2023 Nov 21;103:adv12616.

41.	Papp K, Blauvelt A, Sullivan J, Tada Y, Polzer P, Mallbris L, et al. Ixekizumab—An Effective and Safe Treatment for Moderate-to-Severe Plaque Psoriasis in Patients Previously Treated With Other IL-17 Inhibitors: Results From IXORA-P. J Psoriasis Psoriatic Arthritis®. 2019 Oct 1;4(4):180–5.

42.	Reddy R, Pannu S, Fiumara K, Kahn J, Rosmarin D. Efficacy of in-class interleukin-23 inhibitor switching: risankizumab following guselkumab failure in moderate-to-severe psoriasis treatment. Br J Dermatol. 2021 Mar;184(3):559–61.

43.	Gasslitter I, Kirsten N, Augustin M, Torz K, Mrowietz U, Eyerich K, et al. Successful intra-class switching among IL-17 antagonists: a multicentre, multinational, retrospective study. Arch Dermatol Res. 2019 July;311(5):421–4.

44.	Daniele SG, Eldirany SA, Damiani G, Ho M, Bunick CG. Structural Basis for p19 Targeting by Anti-IL-23 Biologics: Correlations with Short- and Long-Term Efficacy in Psoriasis. JID Innov Skin Sci Mol Popul Health. 2024 Mar;4(2):100261.

45.	Camela E, Ocampo‐Garza SS, Cinelli E, Villani A, Fabbrocini G, Megna M. Therapeutic update of biologics and small molecules for scalp psoriasis: a systematic review. Dermatol Ther. 2021;34(2):e14857.

46.	Mosca M, Hong J, Hadeler E, Brownstone N, Bhutani T, Liao W. Scalp psoriasis: a literature review of effective therapies and updated recommendations for practical management. Dermatol Ther. 2021;11(3):769–97.

47.	Tsiogkas SG, Karamitrou EK, Grammatikopoulou MG, Zafiriou E, Bogdanos DP. Efficacy of tyrosine-kinase-2 and phosphodiesterase-4 inhibitors for scalp psoriasis: a systematic review and meta-analysis. Curr Med Res Opin. 2024;40(2):155–63.

48.	Duffin KC, Griffiths CE, Hoffmann M, Blauvelt A, Balagula E, Napoli A, et al. Deucravacitinib, an Oral Selective Tyrosine Kinase 2 (TYK2) Inhibitor, in Patients with Moderate to Severe Scalp Psoriasis: Efficacy and Safety Results of a Phase 3b/4, Multicenter, Randomized, Double-blinded, Placebo-controlled Trial (PSORIATYK SCALP). SKIN J Cutan Med. 2024;8(6):s420–s420.

49.	Khan M, Wallace CE, Ahmed F, Rahman SM, Memon N, Haque A. Assessing Comparative Efficacy of Biologics for the Treatment of Psoriasis With Nail Involvement: A Systematic Review. J Psoriasis Psoriatic Arthritis®. 2024;9(2):61–8.

50.	Hwang JK, Ricardo JW, Lipner SR. Efficacy and safety of nail psoriasis targeted therapies: a systematic review. Am J Clin Dermatol. 2023;24(5):695–720.

51.	Hong JJ, Mosca ML, Hadeler EK, Brownstone ND, Bhutani T, Liao WJ. Genital and inverse/intertriginous psoriasis: an updated review of therapies and recommendations for practical management. Dermatol Ther. 2021;11(3):833–44.

52.	Beck KM, Yang EJ, Sanchez IM, Liao W. Treatment of Genital Psoriasis: A Systematic Review. Dermatol Ther. 2018 Dec;8(4):509–25.

53.	Tsiogkas SG, Grammatikopoulou MG, Kontouli KM, Minopoulou I, Goulis DD, Zafiriou E, et al. Efficacy of biologic agents for palmoplantar psoriasis: a systematic review and network meta-analysis. Expert Rev Clin Immunol. 2023;19(12):1485–98.

54.	Huang IH, Wu PC, Chiu HY, Huang YH. Small-molecule inhibitors and biologics for palmoplantar psoriasis and palmoplantar pustulosis: a systematic review and network meta-analysis. Am J Clin Dermatol. 2024;25(3):347–58.

55.	Xia R, Diao Z, Chen D, Wang Y, Zhou C, Gao Y, et al. Efficacy of anti-interleukin-17A biological agents for palmoplantar psoriasis and palmoplantar pustulosis: A network meta-analysis. Int Immunopharmacol. 2024;130:111716.

56.	Reynolds KA, Pithadia DJ, Lee EB, Liao W, Wu JJ. A systematic review of treatment strategies for erythrodermic psoriasis. J Dermatol Treat. 2021 Feb;32(1):49–55.

57.	Lo Y, Tsai TF. Updates on the treatment of erythrodermic psoriasis. Psoriasis Targets Ther. 2021;59–73.

58.	Zhou T, Koussiouris J, Kim L, Vender R. Management of guttate psoriasis: a systematic review. J Cutan Med Surg. 2024;28(6):577–84.

59.	Rega F, Trovato F, Bortone G, Pellacani G, Richetta AG, Dattola A. Therapeutic potential of Spesolimab-Sbzo in the management of generalized pustular psoriasis flares in adults: evidence to date. Psoriasis Targets Ther. 2024;23–7.

60.	Puig L, Fujita H, Thaçi D, Zheng M, Hernandez Daly AC, Leonardi C, et al. Current treatments for generalized pustular psoriasis: a narrative summary of a systematic literature search. Dermatol Ther. 2024;14(9):2331–78.

61.	Chen B lin, Liu Q wei, Dong X wan, Bai Y ping. Biologics for generalized pustular psoriasis: a systematic review and single-arm meta-analysis. Front Immunol. 2024;15:1462158.

62.	Kearns DG, Chat VS, Zang PD, Han G, Wu JJ. Review of treatments for generalized pustular psoriasis. J Dermatol Treat. 2021 July 4;32(5):492–4.

63.	Sánchez‐García V, Hernández‐Quiles R, de‐Miguel‐Balsa E, Giménez‐Richarte Á, Ramos‐Rincón J, Belinchón‐Romero I. Exposure to biologic therapy before and during pregnancy in patients with psoriasis: systematic review and meta‐analysis. J Eur Acad Dermatol Venereol. 2023;37(10):1971–90.

64.	Owczarek W, Walecka I, Lesiak A, Czajkowski R, Reich A, Zerda I, et al. The use of biological drugs in psoriasis patients prior to pregnancy, during pregnancy and lactation: a review of current clinical guidelines. Adv Dermatol Allergol Dermatol Alergol. 2020;37(6):821–30.

65.	Jeong YD, Jo H, Cho H, Jang W, Park J, Lee S, et al. Biologics use for psoriasis during pregnancy and its related adverse outcomes in pregnant women and newborns: Findings from WHO pharmacovigilance study. Int Arch Allergy Immunol. 2025;186(6):579–93.

66.	Battista T, Gallo L, Martora F, Fattore D, Potestio L, Cacciapuoti S, et al. Biological therapy for psoriasis in cancer patients: an 8-year retrospective real-life study. J Clin Med. 2024;13(7):1940.

67.	Gupta A, Peyrin-Biroulet L, Ananthakrishnan AN. Risk of cancer recurrence in patients with immune-mediated diseases with use of immunosuppressive therapies: an updated systematic review and meta-analysis. Clin Gastroenterol Hepatol. 2024;22(3):499–512.

68.	Russell MD, Stovin C, Alveyn E, Adeyemi O, Chan CKD, Patel V, et al. JAK inhibitors and the risk of malignancy: a meta-analysis across disease indications. Ann Rheum Dis. 2023;82(8):1059–67.

69.	Alhalabi MM, Almokdad R. The incidence of hepatitis B reactivation in patients receiving ustekinumab: a systematic review and proportional meta-analysis. Eur J Gastroenterol Hepatol. 2025;37(1):1–9.

70.	Kuo MH, Ko PH, Wang ST, Tseng CW. Incidence of HBV Reactivation in Psoriasis Patients Undergoing Cytokine Inhibitor Therapy: A Single-Center Study and Systematic Review with a Meta-Analysis. Viruses. 2024;17(1):42.

71.	Li L, Jiang X, Fu L, Zhang L, Feng Y. Reactivation rates of hepatitis B or C or HIV in patients with psoriasis using biological therapies: a systematic review and meta-analysis. Clin Exp Med. 2023;23(3):701–15.

72.	Megna M, Lauletta G, Tommasino N, Salsano A, Battista T, Ruggiero A, et al. Management of psoriasis patients with serious infectious diseases. Adv Ther. 2024;41(6):2099–111.

73.	Zhu X, Pan X, Da M, Wang F, Dong Z. Risk for latent tuberculosis infection reactivation among patients with psoriasis on biologics treatment: a meta-analysis. J Infect. 2024;89(3).

74.	Mateu-Arrom L, Puig L. Infection Risk and Vaccination in the Management of Psoriasis: Considerations for Biologic Therapy. Psoriasis Targets Ther. 2025;127–44.

75.	Jafarzadeh A, Behrangi E, Khosravi M, Falakeh S, Amiri JK, Goodarzi A. Emerging treatments for dermatologic diseases in infants, children, and adolescents: a systematic review of clinical trials on biologics and small molecule inhibitors. Inflammopharmacology. 2025;1–56.

76.	Sticherling M, Nikkels AF, Hamza AM, Kwong P, Szepietowski JC, El Sayed M, et al. Secukinumab in pediatric patients with plaque psoriasis: pooled safety analysis from two phase 3 randomized clinical trials. Am J Clin Dermatol. 2023;24(5):821–35.

77.	Li C, Sunhe Y, Zhou H, Dong W. Efficacy and safety evaluations of adalimumab biosimilars in the treatment of psoriasis. J Dermatol Treat. 2023;34(1):2249145.

78.	Phan DB, Elyoussfi S, Stevenson M, Lunt M, Warren RB, Yiu ZZ. Biosimilars for the treatment of psoriasis: a systematic review of clinical trials and observational studies. JAMA Dermatol. 2023;159(7):763–71.

79.	Macaluso FS, Cummings JF, Atreya R, Choi J, Orlando A. A systematic review on infliximab biosimilar SB2: from pre-clinical data to real-world evidence. Expert Opin Biol Ther. 2022;22(2):203–23.

80.	Wang Q, Wang J, Sun X, Liu L, Zhang M, Yu Y, et al. Evidence-based dietary recommendations for patients with psoriasis: A systematic review. Clin Nutr. 2025;47:68–82.

81.	Wei K, Liao X, Yang T, He X, Yang D, Lai L, et al. Efficacy of probiotic supplementation in the treatment of psoriasis—A systematic review and meta‐analysis. J Cosmet Dermatol. 2024;23(7):2361–7.

82.	Lu L, Xu Y, Shi M, Liu A. Psychosocial interventions for psoriasis: a Bayesian network meta-analysis. J Dermatol Treat. 2025;36(1):2427321.

83.	Wei W, Zhang B, Liu T, Lu T. Effect of psychological intervention on quality of life among patients with psoriasis: a meta-analysis. Int J Behav Med. 2024;31(6):911–22.

## QUESTION 15. In patients with moderate-to-severe plaque psoriasis, what are the efficacy and safety of biologic therapies for psoriasis in high-impact areas?

**Scalp psoriasis**

[43,44], one systematic review of small molecules[45] and a recent clinical trial of deucravacitinib[46]. The available evidence indicates that several systemic therapies are effective for scalp psoriasis, including anti–TNF agents, IL-17 and IL-23 inhibitors, anti–IL-12/23 therapy, and small molecules such as apremilast and deucravacitinib. Direct, head-to-head comparisons are limited by heterogeneity in outcome measures, so results are presented descriptively by intervention.

***Efficacy of biologics in patients with scalp psoriasis***

| **Outcome** | **Population / Follow-up** | **Study design** | **Certainty of evidence (GRADE)** | **Main result** | **Interpretation** |
| --- | --- | --- | --- | --- | --- |
| Efficacy – Psoriasis Scalp Severity Index (PSSI) | Etanercept 50 mg twice weekly | Randomized clinical trial | Moderate ⨁⨁⨁◯ | SMD 1.59 (1.40–1.78) | More effective than placebo |
| Efficacy – Psoriasis Scalp Severity Index (PSSI) | Ixekizumab 160 mg every 4 weeks | Randomized clinical trial | Moderate ⨁⨁⨁◯ | SMD 2.10 (1.90–2.30) | More effective than placebo |
| Efficacy – Psoriasis Scalp Severity Index (PSSI) | Secukinumab 300 mg weekly for 4 weeks, then every 4 weeks | Randomized clinical trial | Moderate ⨁⨁⨁◯ | SMD 3.09 (2.45–3.73) | More effective than placebo |
| Efficacy – Psoriasis Scalp Severity Index (PSSI) | Brodalumab 210 mg weekly for 3 weeks, then every 2 weeks | Randomized clinical trial | Moderate ⨁⨁⨁◯ | SMD 1.67 (1.39–1.94) | More effective than placebo |
| Efficacy – scalp-specific Investigator Global Assessment (IGA) | Adalimumab 80 mg at week 0, 40 mg at week 1, then every 2 weeks | Randomized clinical trial | Moderate ⨁⨁⨁◯ | SMD −1.22 (−1.93 to −0.52) | More effective than placebo |
| Efficacy – scalp-specific IGA 0/1 | Guselkumab 100 mg every 8 weeks | Randomized clinical trial vs adalimumab | Moderate ⨁⨁⨁◯ | Week 16: GUS 81.8% vs ADA 69.0% • Week 24: GUS 85% vs ADA 68.5% | More effective than adalimumab |
| Efficacy – scalp-specific Investigator Global Assessment (IGA) | Ustekinumab 90 mg every 12 weeks | Randomized clinical trial | Moderate ⨁⨁⨁◯ | SMD −1.78 (−2.77 to −0.80) | More effective than placebo |
| Efficacy – Mean percent improvement in PSSI | Risankizumab 90 mg/180 mg at weeks 0, 4, and 16 | Randomized clinical trial vs ustekinumab | Moderate ⨁⨁⨁◯ | 94% vs 82% | More effective than ustekinumab |
| Efficacy – Achievement of PSSI 90 | Infliximab 5 mg/kg at weeks 0, 2, 6, 14, and 22 | Randomized clinical trial vs adalimumab | Moderate ⨁⨁⨁◯ | Week 16: IFX 70.0% vs ADA 45.5% • Week 24: IFX 70.0% vs ADA 54.5% | More effective than adalimumab |

***Explanations***

1. All studies had at least one domain at high or unclear risk of bias, primarily related to allocation concealment, selective reporting, and other biases.

***Efficacy of small molecules in patients with scalp psoriasis***

| **Outcome** | **Population / Follow-up** | **Study design** | **Certainty of evidence (GRADE)** | **Main result** | **Interpretation** |
| --- | --- | --- | --- | --- | --- |
| Skin clear or almost clear at 16 weeks | Apremilast 623/1464; placebo 181/1043 | Randomized clinical trial (9) | High ⨁⨁⨁⨁ | RR 2.41 (2.08–2.79) | More effective than placebo |
| Skin clear or almost clear at 16 weeks | Deucravacitinib 395/619; placebo 56/345 | Randomized clinical trial (3) | High ⨁⨁⨁⨁ | RR 3.86 (3.02–4.94) | More effective than placebo |

The PSORIATYK SCALP trial [46] showed that deucravacitinib is effective and well tolerated in patients with moderate-to-severe scalp psoriasis, including those with limited overall skin involvement. The safety profile was consistent with prior experience, and no new safety signals were identified. Overall, newer biologics—particularly IL-17 and IL-23 inhibitors—appear to be associated with better clinical outcomes for scalp disease and its quality-of-life impact. In the absence of direct head-to-head comparisons across interventions, treatment selection should be individualized based on patient characteristics and preferences.

**Nail psoriasis**

Two systematic reviews [47,48] encompassing 68 studies and 15 targeted therapies for nail psoriasis were included. The evidence indicates that multiple systemic and biologic therapies—including TNF-α, IL-17, IL-23, and IL-12/23 inhibitors, as well as PDE-4, CD11a, and JAK inhibitors—produce clinically meaningful improvements in nail involvement. Across studies, treatments generally showed acceptable safety profiles, with adverse events mostly mild and consistent with known drug effects.

**Efficacy of biologics in patients with nail psoriasis at week 24**

| **Biologic** | **Mean percent improvement in NAPSI at week 24** | **Mean percent improvement in mNAPSI** | **NAPSI = 0 at week 24 (%)** |
| --- | --- | --- | --- |
| Adalimumab | 57.2%ᵃ | NR | 44.6% |
| Guselkumab | 52.4% | NR | NR |
| Ustekinumab | 55.7% | NR | 19.4% |
| Ixekizumab | 70.5% | NR | 41% |
| Infliximab | 57.2% | NR | 26.2% |
| Etanercept | 74% | NR | 31% |
| Brodalumab | 76.9% | NR | 31.6% |
| Golimumab | 44% | NR | NR |
| Risankizumab | NR | 54.1% | NR |
| Secukinumab | 41.6%ᵇ | NR | NR |
| Certolizumab pegol | NR | 55.2% | NR |
| Placebo | 4.5% | 33% | 7.8% |

*NR: Not reported.*

*^a.^ The mean includes one 26-week study (if that study is excluded: 57.4%).*

*^b.^ Derived from a 16-week study; the 32-week result from the same study was 57.9%*.

**Efficacy of biologics in patients with nail psoriasis at week 52**

| **Biologic** | **Mean percent improvement in NAPSI at week 52** | **NAPSI = 0 at week 52 (%)** |
| --- | --- | --- |
| Brodalumab | 83.1% | 63.80% |
| Ustekinumab | 69% | 33.85% |
| Guselkumab | 68.1%ᵃ | NR |
| Ixekizumab | 83.3% | 57.70% |
| Adalimumab | 70.2%ᵇ | 58.80% |
| Placebo | −45% | NR |

*NR: Not reported.*

*ᵃ Derived from a 48-week study.*

*ᵇ The mean includes one 48-week study (mean if that study is excluded: 79%).*

**Genital psoriasis**

Evidence on the treatment of genital psoriasis is derived primarily from narrative literature reviews [49]^,^ [50] of open-label studies and case series. Topical corticosteroids show good tolerability and effectiveness, while nonsteroidal topical options—such as calcineurin inhibitors and vitamin D analogs—are also effective but carry a higher risk of local irritation. In severe or refractory disease, systemic therapy is suggested. Among biologics, ixekizumab has demonstrated efficacy in a placebo-controlled trial. Overall, the available evidence remains limited, and dedicated controlled studies are needed to better define optimal treatment strategies.

**Palmoplantar psoriasis**

Evidence for palmoplantar psoriasis and palmoplantar pustulosis comes from three systematic reviews [51–53] with meta-analyses that primarily evaluated biologics and, in one case, small molecules. Biologics demonstrated efficacy in lesion clearance and improvements in PPASI, with generally favorable safety profiles. The certainty of evidence is limited because these outcomes are commonly secondary endpoints in trials. Evidence for small molecules is sparse, and the role of newer classes, such as JAK inhibitors, remains uncertain.

**Efficacy of biologics and small molecules in patients with palmoplantar psoriasis**

| **Outcome** | **Population / Follow-up** | **Study design** | **Certainty of evidence (GRADE)** | **Main result** | **Interpretation** |
| --- | --- | --- | --- | --- | --- |
| Efficacy – PPPGA 0/1 | Secukinumab 300 mg every 4 weeks | Randomized clinical trial vs placebo | Moderate ⨁⨁⨁◯ | RR 22.67 (3.08–166.58) | More effective than placebo |
| Efficacy – PPPGA 0/1 | Ustekinumab | Randomized clinical trial (1) vs placebo | Moderate ⨁⨁⨁◯ | RR 3.23 (2.06–5.07) | More effective than placebo |
| Efficacy – PPPGA 0/1 | Guselkumab | Randomized clinical trial (2) vs placebo | Moderate ⨁⨁⨁◯ | RR 3.16 (2.20–4.53) | More effective than placebo |
| Efficacy – PPPGA 0/1 | Bimekizumab | Randomized clinical trial (2) vs placebo | Moderate ⨁⨁⨁◯ | RR 3.13 (2.20–4.45) | More effective than placebo |
| Efficacy – PPPGA 0/1 | Ixekizumab | Randomized clinical trial vs placebo | Moderate ⨁⨁⨁◯ | RR 2.88 (2.15–3.87) | More effective than placebo |
| Efficacy – PPPGA 0/1 | Infliximab | Randomized clinical trial vs placebo | Moderate ⨁⨁⨁◯ | RR 3.00 (0.35–25.40) | More effective than placebo |
| Efficacy – PPPGA 0/1 | Adalimumab | Randomized clinical trial (3) vs placebo | Moderate ⨁⨁⨁◯ | RR 2.67 (1.90–3.76) | More effective than placebo |
| Efficacy – PPPGA 0/1 | Etanercept | Randomized clinical trial (2) vs placebo | Moderate ⨁⨁⨁◯ | RR 2.30 (1.64–3.24) | More effective than placebo |

***Explanations***

1. Several studies had unclear risk of bias in two or more domains, mainly due to incomplete outcome data and other biases.

## QUESTION 16. What are the efficacy and safety of biologic therapies in special forms of psoriasis?

**Erythrodermic psoriasis**

Evidence for erythrodermic psoriasis management comes from two systematic literature reviews [54,55]. The review by Reynolds et al. included 23 studies with more than 200 patients. Biologics—including TNF inhibitors (infliximab [n = 4], etanercept [n = 2], and adalimumab [n = 1]), IL-17 inhibitors (secukinumab [n = 3], ixekizumab [n = 2], and brodalumab [n = 1]), ustekinumab (n = 4), and guselkumab (n = 1)—were associated with rapid clinical improvement in erythrodermic psoriasis. Efficacy was also reported with conventional systemic agents such as cyclosporine (n = 4), etretinate (n = 3), and methotrexate (n = 1).

Across reports, both biologic and oral systemic therapies produced rapid symptom improvement, with high PASI 90 and PASI 100 response rates among patients treated with biologics. There is growing support for biologics, particularly IL-17 inhibitors, given their rapid onset even when the trigger for erythroderma is uncertain. The lack of randomized, double-blind comparative trials prevents identification of a clear first-line option.

**Guttate psoriasis**

The systematic review by Zhou et al. (2024)[56] assessed evidence for guttate psoriasis treatments, largely based on observational studies and only five randomized clinical trials. These RCTs did not demonstrate meaningful benefit of antibiotics or maintenance phototherapy compared with control. Most evidence comes from case reports and series, where topical therapy and phototherapy—especially narrow-band UVB and PUVA—showed high response rates and acceptable safety. Favorable reports also exist for anti–IL-17, anti–IL-23, and anti–IL-12/23 biologics, although evidence is limited and observational. Between 17.5% and 38.9% of patients progress to chronic plaque psoriasis. Overall certainty of evidence is low; in extensive or refractory disease, systemic therapy or biologics can be considered when other options have failed.

**Pustular psoriasis**

Evidence for pustular psoriasis management comes primarily from systematic reviews [57–59] and observational studies[60], with overall low certainty due to small sample sizes and pathophysiologic heterogeneity across pustular presentations. Traditional systemic therapies—acitretin, cyclosporine, and methotrexate—remain first-line options. Oral acitretin at 0.75 to 1 mg/kg/day has been reported to be effective, with response in 7 to 10 days and complete clearance within 2 weeks. To prevent recurrence, maintenance dosing of 0.125 to 0.25 mg/kg/day for months has been recommended. Cyclosporine has shown efficacy in GPP, with case reports describing resolution of pustules and lymphadenopathy within 2 to 4 weeks at 2 mg/kg/day. Methotrexate has also shown effectiveness, with two retrospective studies reporting response rates of 76.2% and 80%, respectively. Typical dosing ranges from 7.5 to 25 mg weekly, with regular monitoring for hematologic and hepatic abnormalities.

More recent evidence highlights biologics, particularly IL-36 inhibitors. Meta-analyses and recent reviews indicate that these agents achieve faster and more clinically meaningful responses than other biologics, with an acceptable safety profile. Spesolimab has the strongest support, as the only agent with positive results in randomized controlled trials, demonstrating rapid pustule clearance and a reduction in relapse frequency. In Effisayil™ 1, a single intravenous bolus of spesolimab achieved complete pustule clearance (GPPGA subscore = 0) by the end of week 1 in 54% of patients, compared with 6% in the placebo group. Improvement in overall GPPGA (0 or 1) was also greater in the active-treatment group. Although anti-spesolimab antibodies were detected in a substantial proportion of patients and some infections were reported, the overall safety profile was acceptable. In Effisayil™ 2 (123 patients), higher-dose regimens reduced relapse risk by 84% compared with placebo (HR = 0.16; p = 0.0005). Infection rates were similar across groups, and no deaths were reported.

IL-17 inhibitors, IL-23 inhibitors, and TNF inhibitors have also shown effectiveness. Overall, current data support biologic therapy—particularly spesolimab—while underscoring the need for additional RCTs with standardized outcomes to better define optimal management.

**Efficacy of biologics in patients with pustular psoriasis**

| **Outcome** | **Population / Follow-up** | **Study design** | **Certainty of evidence (GRADE)** | **Proportion (95% CI)** |
| --- | --- | --- | --- | --- |
| Efficacy – Proportion achieving a GPPASI75 response | TNF inhibitors (anti-TNF) | Prospective cohort (2) | Low ⨁⨁◯◯ | 0.06 (0.00–0.37) |
| Efficacy – Proportion achieving a GPPASI75 response | IL-17 inhibitors (anti–IL-17) | Prospective cohort (4); Case series (1); Randomized trial (1) | Low ⨁⨁◯◯ | 0.11 (0.00–0.34) |
| Efficacy – Proportion achieving a GPPASI75 response | IL-23 inhibitors (anti–IL-23) | Randomized clinical trial (2) vs placebo | Low ⨁⨁◯◯ | 0.00 (0.00–0.07) |
| Efficacy – Proportion achieving a GPPASI75 response | IL-36 inhibitors (anti–IL-36) | Prospective cohort (2) | Low ⨁⨁◯◯ | 0.40 (0.27–0.54) |

***Explanations***

Several studies had moderate or high risk of bias in two or more domains, mainly due to confounding, participant selection, and outcome measurement.

**References**

1.	Yasmeen N, Sawyer LM, Malottki K, Levin LÅ, Didriksen Apol E, Jemec GB. Targeted therapies for patients with moderate-to-severe psoriasis: a systematic review and network meta-analysis of PASI response at 1 year. J Dermatol Treat. 2022 Jan 2;33(1):204–18.

2.	Belinchón Romero I, Dauden E, Ferrándiz Foraster C, González-Cantero Á, Carrascosa Carrillo JM. Therapeutic goals and treatment response evaluation in moderate to severe psoriasis: an experts opinion document. Ann Med. 2021 Jan;53(1):1727–36.

3.	Galluzzo M, Talamonti M, Manfreda V, Silvaggio D, Tartaglia C, Roberto D, et al. Impact of secukinumab on patient-reported outcomes in moderate to severe plaque psoriasis: a review of clinical studies. Expert Opin Biol Ther. 2021 Feb 1;21(2):191–200.

4.	Rusk AM, Fleischer AB. In psoriasis treatment, greater improvement in skin severity predicts greater improvement in nail severity. J Dermatol Treat. 2021 Nov 17;32(8):894–7.

5.	Belinchon Romero I, Mateu Puchades A, Ribera Pibernat M, Ruiz Genao DianaP, De La Cueva Dobao P, Carrascosa JM. Criteria used to define tumor necrosis factor-alpha inhibitors failure in patients with moderate-to-severe psoriasis: a systematic literature review. Ann Med. 2023 Dec 12;55(1):1335–45.

6.	Gisondi P, Di Mercurio M, Idolazzi L, Girolomoni G. Concept of Remission in Chronic Plaque Psoriasis. J Rheumatol Suppl. 2015 Nov 1;93(0):57–60.

7.	Puig L. PASI 90 response: the new standard in therapeutic efficacy for psoriasis. J Eur Acad Dermatol Venereol. 2015 Apr;29(4):645–8.

8.	Boswell ND, Singla S, Gordon KB. Sequencing of Targeted Therapy in Psoriasis: Does it Matter? Am J Clin Dermatol. 2024 Sept;25(5):795–810.

9.	Thaçi D, Piaserico S, Gaarn Du Jardin K, Warren RB. Long‐term stability of PASI <3 response to tildrakizumab: Pooled analyses from reSURFACE 1 and reSURFACE 2 phase 3 trials through 5 years. J Eur Acad Dermatol Venereol [Internet]. 2023 Apr [cited 2024 Oct 4];37(4). Available from: https://onlinelibrary.wiley.com/doi/10.1111/jdv.18783

10.	Imafuku S, Ohata C, Okubo Y, Tobita R, Saeki H, Mabuchi T, et al. Effectiveness of brodalumab in achieving treatment satisfaction for patients with plaque psoriasis: The ProLOGUE study. J Dermatol Sci. 2022 Mar;105(3):176–84.

11.	Reich K, Mrowietz U, Menter A, Griffiths CEM, Bagel J, Strober B, et al. Effect of baseline disease severity on achievement of treatment target with apremilast: results from a pooled analysis. J Eur Acad Dermatol Venereol. 2021 Dec;35(12):2409–14.

12.	Campanati A, Diotallevi F, Radi G, Molinelli E, Brisigotti V, Martina E, et al. Psoriatic patients treated with secukinumab reach high levels of minimal disease activity: results from the SUPREME study. Eur J Dermatol. 2021 Oct;31(5):630–7.

13.	Lebwohl MG, Soliman AM, Yang H, Wang J, Koenigsberg SH, Anderson A, et al. 25572 Impact of risankizumab on the duration of PASI90 achievement: A post hoc analysis of 4 phase III clinical trials. J Am Acad Dermatol. 2021 Sept;85(3):AB62.

14.	Puig L, Zhu B, Burge R, Shrom D, Dong Y, Shen W, et al. Early Treatment Targets for Predicting Long-term Dermatology Life Quality Index Response in Patients with Moderate-to-Severe Plaque Psoriasis: A Post-hoc Analysis from a Long-term Clinical Study. J Clin Aesthetic Dermatol. 2020 Oct;13(10):18–22.

15.	Puig L, Dossenbach M, Berggren L, Ljungberg A, Zachariae C. Absolute and Relative Psoriasis Area and Severity Indices (PASI) for Comparison of the Efficacy of Ixekizumab to Etanercept and Placebo in Patients with Moderate-to-severe Plaque Psoriasis: An Integrated Analysis of UNCOVER-2 and UNCOVER-3 Outcomes. Acta Derm Venereol. 2019;99(11):971–7.

16.	Merola JF, Amato DA, See K, Burge R, Mallinckrodt C, Ojeh CK, et al. Evaluation of sPGA × BSA as an Outcome Measure and Treatment Target for Clinical Practice. J Invest Dermatol. 2018 Sept;138(9):1955–61.

17.	Elewski BE, Puig L, Mordin M, Gilloteau I, Sherif B, Fox T, et al. Psoriasis patients with psoriasis Area and Severity Index (PASI) 90 response achieve greater health-related quality-of-life improvements than those with PASI 75–89 response: results from two phase 3 studies of secukinumab. J Dermatol Treat. 2017 Aug 18;28(6):492–9.

18.	Schmitt J, Rosumeck S, Thomaschewski G, Sporbeck B, Haufe E, Nast A. Efficacy and safety of systemic treatments for moderate-to-severe psoriasis: meta-analysis of randomized controlled trials. Br J Dermatol. 2014 Feb;170(2):274–303.

19.	Li Z, Pei B, Lei T, Yao M, Niu Y, Zhou P, et al. Clinical features and effects of cyclosporine in the treatment of psoriasis: a systemic review and meta-analysis. Arch Dermatol Res. 2024 Oct 26;316(10):705.

20.	Kogan N, Raimondo N, Gusis SE, Izcovich A, Abarca Duran JA, Barahona‐Torres L, et al. Latin American clinical practice guidelines on the systemic treatment of psoriasis SOLAPSO–Sociedad Latinoamericana de Psoriasis (Latin American Psoriasis Society). Int J Dermatol. 2019;58:4–28.

21.	van Huizen AM, Sikkel R, Caron AG, Menting SP, Spuls PI. Methotrexate dosing regimen for plaque-type psoriasis: an update of a systematic review. J Dermatol Treat. 2022;33(8):3104–18.

22.	Hsieh TS, Tsai TF. Combination therapy for psoriasis with methotrexate and other oral disease-modifying antirheumatic drugs: a systematic review. Dermatol Ther. 2023;13(4):891–909.

23.	Cheng Y, Liu GJ, Zhang M. Narrow‐band ultraviolet B phototherapy versus broad‐band ultraviolet B or psoralen‐ultraviolet A photochemotherapy for psoriasis. Cochrane Database Syst Rev. 2013;(10):CD009481.

24.	Sbidian E, Chaimani A, Garcia-Doval I, Doney L, Dressler C, Hua C, et al. Systemic pharmacological treatments for chronic plaque psoriasis: a network meta-analysis. Cochrane Database Syst Rev. 2022 May 23;5(5):CD011535.

25.	Mahmoud A, Ahmed AAS, Naeem A, Abuelazm M, Elshinawy M, Hassan AR, et al. Tyrosine Kinase-2 Inhibitor (Deucravacitinib) for Psoriasis: A Systematic Review and Network Meta-Analysis of Randomized Controlled Trials. Int J Dermatol Venereol. 2024 Dec;7(4):216.

26.	Armstrong AW, Soliman AM, Betts KA, Wang Y, Gao Y, Stakias V, et al. Long-term benefit–risk profiles of treatments for moderate-to-severe plaque psoriasis: a network meta-analysis. Dermatol Ther. 2022;12(1):167–84.

27.	Piragine E, Petri D, Martelli A, Janowska A, Dini V, Romanelli M, et al. Adherence and persistence to biological drugs for psoriasis: systematic review with meta-analysis. J Clin Med. 2022;11(6):1506.

28.	Gottlieb AB, Kalb RE, Blauvelt A, Heffernan MP, Sofen HL, Ferris LK, et al. The efficacy and safety of infliximab in patients with plaque psoriasis who had an inadequate response to etanercept: results of a prospective, multicenter, open-label study. J Am Acad Dermatol. 2012 Oct;67(4):642–50.

29.	Yamauchi PS, Bissonnette R, Teixeira HD, Valdecantos WC. Systematic review of efficacy of anti-tumor necrosis factor (TNF) therapy in patients with psoriasis previously treated with a different anti-TNF agent. J Am Acad Dermatol. 2016 Sept;75(3):612-618.e6.

30.	Piaserico S, Cazzaniga S, Chimenti S, Giannetti A, Maccarone M, Picardo M, et al. Efficacy of switching between tumor necrosis factor-alfa inhibitors in psoriasis: results from the Italian Psocare registry. J Am Acad Dermatol. 2014 Feb;70(2):257-262.e3.

31.	Boswell ND, Singla S, Gordon KB. Sequencing of Targeted Therapy in Psoriasis: Does it Matter? Am J Clin Dermatol. 2024 Sept;25(5):795–810.

32.	Warren RB, Blauvelt A, Poulin Y, Beeck S, Kelly M, Wu T, et al. Efficacy and safety of risankizumab vs. secukinumab in patients with moderate-to-severe plaque psoriasis (IMMerge): results from a phase III, randomized, open-label, efficacy-assessor-blinded clinical trial. Br J Dermatol. 2021 Jan;184(1):50–9.

33.	Blauvelt A, Papp KA, Griffiths CEM, Randazzo B, Wasfi Y, Shen YK, et al. Efficacy and safety of guselkumab, an anti-interleukin-23 monoclonal antibody, compared with adalimumab for the continuous treatment of patients with moderate to severe psoriasis: Results from the phase III, double-blinded, placebo- and active comparator-controlled VOYAGE 1 trial. J Am Acad Dermatol. 2017 Mar;76(3):405–17.

34.	Reich K, Armstrong AW, Foley P, Song M, Wasfi Y, Randazzo B, et al. Efficacy and safety of guselkumab, an anti-interleukin-23 monoclonal antibody, compared with adalimumab for the treatment of patients with moderate to severe psoriasis with randomized withdrawal and retreatment: Results from the phase III, double-blind, placebo- and active comparator-controlled VOYAGE 2 trial. J Am Acad Dermatol. 2017 Mar;76(3):418–31.

35.	Reich K, Gooderham M, Thaçi D, Crowley JJ, Ryan C, Krueger JG, et al. Risankizumab compared with adalimumab in patients with moderate-to-severe plaque psoriasis (IMMvent): a randomised, double-blind, active-comparator-controlled phase 3 trial. Lancet Lond Engl. 2019 Aug 17;394(10198):576–86.

36.	Warren RB, Blauvelt A, Bagel J, Papp KA, Yamauchi P, Armstrong A, et al. Bimekizumab versus Adalimumab in Plaque Psoriasis. N Engl J Med. 2021 July 8;385(2):130–41.

37.	Warren RB, Barker JNWB, Finlay AY, Burden AD, Kirby B, Armendariz Y, et al. Secukinumab for patients failing previous tumour necrosis factor-α inhibitor therapy: results of a randomized open-label study (SIGNATURE). Br J Dermatol. 2020 July;183(1):60–70.

38.	Strober B, Paul C, Blauvelt A, Thaçi D, Puig L, Lebwohl M, et al. Bimekizumab efficacy and safety in patients with moderate to severe plaque psoriasis: Two-year interim results from the open-label extension of the randomized BE RADIANT phase 3b trial. J Am Acad Dermatol. 2023 Sept;89(3):486–95.

39.	Loft N, Halling AS, Egeberg A, Skov L. Efficacy of a second interleukin 17 inhibitor in patients with psoriasis: A systematic review and meta-analysis. J Am Acad Dermatol. 2021 Jan;84(1):130–8.

40.	Loft N, Egeberg A, Isufi D, Rasmussen MK, Bryld LE, Dam TN, et al. Response to Interleukin-17A Inhibitors According to Prior Biologic Exposures: A Danish Nationwide Study. Acta Derm Venereol. 2023 Nov 21;103:adv12616.

41.	Papp K, Blauvelt A, Sullivan J, Tada Y, Polzer P, Mallbris L, et al. Ixekizumab—An Effective and Safe Treatment for Moderate-to-Severe Plaque Psoriasis in Patients Previously Treated With Other IL-17 Inhibitors: Results From IXORA-P. J Psoriasis Psoriatic Arthritis®. 2019 Oct 1;4(4):180–5.

42.	Reddy R, Pannu S, Fiumara K, Kahn J, Rosmarin D. Efficacy of in-class interleukin-23 inhibitor switching: risankizumab following guselkumab failure in moderate-to-severe psoriasis treatment. Br J Dermatol. 2021 Mar;184(3):559–61.

43.	Gasslitter I, Kirsten N, Augustin M, Torz K, Mrowietz U, Eyerich K, et al. Successful intra-class switching among IL-17 antagonists: a multicentre, multinational, retrospective study. Arch Dermatol Res. 2019 July;311(5):421–4.

44.	Daniele SG, Eldirany SA, Damiani G, Ho M, Bunick CG. Structural Basis for p19 Targeting by Anti-IL-23 Biologics: Correlations with Short- and Long-Term Efficacy in Psoriasis. JID Innov Skin Sci Mol Popul Health. 2024 Mar;4(2):100261.

45.	Camela E, Ocampo‐Garza SS, Cinelli E, Villani A, Fabbrocini G, Megna M. Therapeutic update of biologics and small molecules for scalp psoriasis: a systematic review. Dermatol Ther. 2021;34(2):e14857.

46.	Mosca M, Hong J, Hadeler E, Brownstone N, Bhutani T, Liao W. Scalp psoriasis: a literature review of effective therapies and updated recommendations for practical management. Dermatol Ther. 2021;11(3):769–97.

47.	Tsiogkas SG, Karamitrou EK, Grammatikopoulou MG, Zafiriou E, Bogdanos DP. Efficacy of tyrosine-kinase-2 and phosphodiesterase-4 inhibitors for scalp psoriasis: a systematic review and meta-analysis. Curr Med Res Opin. 2024;40(2):155–63.

48.	Duffin KC, Griffiths CE, Hoffmann M, Blauvelt A, Balagula E, Napoli A, et al. Deucravacitinib, an Oral Selective Tyrosine Kinase 2 (TYK2) Inhibitor, in Patients with Moderate to Severe Scalp Psoriasis: Efficacy and Safety Results of a Phase 3b/4, Multicenter, Randomized, Double-blinded, Placebo-controlled Trial (PSORIATYK SCALP). SKIN J Cutan Med. 2024;8(6):s420–s420.

49.	Khan M, Wallace CE, Ahmed F, Rahman SM, Memon N, Haque A. Assessing Comparative Efficacy of Biologics for the Treatment of Psoriasis With Nail Involvement: A Systematic Review. J Psoriasis Psoriatic Arthritis®. 2024;9(2):61–8.

50.	Hwang JK, Ricardo JW, Lipner SR. Efficacy and safety of nail psoriasis targeted therapies: a systematic review. Am J Clin Dermatol. 2023;24(5):695–720.

51.	Hong JJ, Mosca ML, Hadeler EK, Brownstone ND, Bhutani T, Liao WJ. Genital and inverse/intertriginous psoriasis: an updated review of therapies and recommendations for practical management. Dermatol Ther. 2021;11(3):833–44.

52.	Beck KM, Yang EJ, Sanchez IM, Liao W. Treatment of Genital Psoriasis: A Systematic Review. Dermatol Ther. 2018 Dec;8(4):509–25.

53.	Tsiogkas SG, Grammatikopoulou MG, Kontouli KM, Minopoulou I, Goulis DD, Zafiriou E, et al. Efficacy of biologic agents for palmoplantar psoriasis: a systematic review and network meta-analysis. Expert Rev Clin Immunol. 2023;19(12):1485–98.

54.	Huang IH, Wu PC, Chiu HY, Huang YH. Small-molecule inhibitors and biologics for palmoplantar psoriasis and palmoplantar pustulosis: a systematic review and network meta-analysis. Am J Clin Dermatol. 2024;25(3):347–58.

55.	Xia R, Diao Z, Chen D, Wang Y, Zhou C, Gao Y, et al. Efficacy of anti-interleukin-17A biological agents for palmoplantar psoriasis and palmoplantar pustulosis: A network meta-analysis. Int Immunopharmacol. 2024;130:111716.

56.	Reynolds KA, Pithadia DJ, Lee EB, Liao W, Wu JJ. A systematic review of treatment strategies for erythrodermic psoriasis. J Dermatol Treat. 2021 Feb;32(1):49–55.

57.	Lo Y, Tsai TF. Updates on the treatment of erythrodermic psoriasis. Psoriasis Targets Ther. 2021;59–73.

58.	Zhou T, Koussiouris J, Kim L, Vender R. Management of guttate psoriasis: a systematic review. J Cutan Med Surg. 2024;28(6):577–84.

59.	Rega F, Trovato F, Bortone G, Pellacani G, Richetta AG, Dattola A. Therapeutic potential of Spesolimab-Sbzo in the management of generalized pustular psoriasis flares in adults: evidence to date. Psoriasis Targets Ther. 2024;23–7.

60.	Puig L, Fujita H, Thaçi D, Zheng M, Hernandez Daly AC, Leonardi C, et al. Current treatments for generalized pustular psoriasis: a narrative summary of a systematic literature search. Dermatol Ther. 2024;14(9):2331–78.

61.	Chen B lin, Liu Q wei, Dong X wan, Bai Y ping. Biologics for generalized pustular psoriasis: a systematic review and single-arm meta-analysis. Front Immunol. 2024;15:1462158.

62.	Kearns DG, Chat VS, Zang PD, Han G, Wu JJ. Review of treatments for generalized pustular psoriasis. J Dermatol Treat. 2021 July 4;32(5):492–4.

63.	Sánchez‐García V, Hernández‐Quiles R, de‐Miguel‐Balsa E, Giménez‐Richarte Á, Ramos‐Rincón J, Belinchón‐Romero I. Exposure to biologic therapy before and during pregnancy in patients with psoriasis: systematic review and meta‐analysis. J Eur Acad Dermatol Venereol. 2023;37(10):1971–90.

64.	Owczarek W, Walecka I, Lesiak A, Czajkowski R, Reich A, Zerda I, et al. The use of biological drugs in psoriasis patients prior to pregnancy, during pregnancy and lactation: a review of current clinical guidelines. Adv Dermatol Allergol Dermatol Alergol. 2020;37(6):821–30.

65.	Jeong YD, Jo H, Cho H, Jang W, Park J, Lee S, et al. Biologics use for psoriasis during pregnancy and its related adverse outcomes in pregnant women and newborns: Findings from WHO pharmacovigilance study. Int Arch Allergy Immunol. 2025;186(6):579–93.

66.	Battista T, Gallo L, Martora F, Fattore D, Potestio L, Cacciapuoti S, et al. Biological therapy for psoriasis in cancer patients: an 8-year retrospective real-life study. J Clin Med. 2024;13(7):1940.

67.	Gupta A, Peyrin-Biroulet L, Ananthakrishnan AN. Risk of cancer recurrence in patients with immune-mediated diseases with use of immunosuppressive therapies: an updated systematic review and meta-analysis. Clin Gastroenterol Hepatol. 2024;22(3):499–512.

68.	Russell MD, Stovin C, Alveyn E, Adeyemi O, Chan CKD, Patel V, et al. JAK inhibitors and the risk of malignancy: a meta-analysis across disease indications. Ann Rheum Dis. 2023;82(8):1059–67.

69.	Alhalabi MM, Almokdad R. The incidence of hepatitis B reactivation in patients receiving ustekinumab: a systematic review and proportional meta-analysis. Eur J Gastroenterol Hepatol. 2025;37(1):1–9.

70.	Kuo MH, Ko PH, Wang ST, Tseng CW. Incidence of HBV Reactivation in Psoriasis Patients Undergoing Cytokine Inhibitor Therapy: A Single-Center Study and Systematic Review with a Meta-Analysis. Viruses. 2024;17(1):42.

71.	Li L, Jiang X, Fu L, Zhang L, Feng Y. Reactivation rates of hepatitis B or C or HIV in patients with psoriasis using biological therapies: a systematic review and meta-analysis. Clin Exp Med. 2023;23(3):701–15.

72.	Megna M, Lauletta G, Tommasino N, Salsano A, Battista T, Ruggiero A, et al. Management of psoriasis patients with serious infectious diseases. Adv Ther. 2024;41(6):2099–111.

73.	Zhu X, Pan X, Da M, Wang F, Dong Z. Risk for latent tuberculosis infection reactivation among patients with psoriasis on biologics treatment: a meta-analysis. J Infect. 2024;89(3).

74.	Mateu-Arrom L, Puig L. Infection Risk and Vaccination in the Management of Psoriasis: Considerations for Biologic Therapy. Psoriasis Targets Ther. 2025;127–44.

75.	Jafarzadeh A, Behrangi E, Khosravi M, Falakeh S, Amiri JK, Goodarzi A. Emerging treatments for dermatologic diseases in infants, children, and adolescents: a systematic review of clinical trials on biologics and small molecule inhibitors. Inflammopharmacology. 2025;1–56.

76.	Sticherling M, Nikkels AF, Hamza AM, Kwong P, Szepietowski JC, El Sayed M, et al. Secukinumab in pediatric patients with plaque psoriasis: pooled safety analysis from two phase 3 randomized clinical trials. Am J Clin Dermatol. 2023;24(5):821–35.

77.	Li C, Sunhe Y, Zhou H, Dong W. Efficacy and safety evaluations of adalimumab biosimilars in the treatment of psoriasis. J Dermatol Treat. 2023;34(1):2249145.

78.	Phan DB, Elyoussfi S, Stevenson M, Lunt M, Warren RB, Yiu ZZ. Biosimilars for the treatment of psoriasis: a systematic review of clinical trials and observational studies. JAMA Dermatol. 2023;159(7):763–71.

79.	Macaluso FS, Cummings JF, Atreya R, Choi J, Orlando A. A systematic review on infliximab biosimilar SB2: from pre-clinical data to real-world evidence. Expert Opin Biol Ther. 2022;22(2):203–23.

80.	Wang Q, Wang J, Sun X, Liu L, Zhang M, Yu Y, et al. Evidence-based dietary recommendations for patients with psoriasis: A systematic review. Clin Nutr. 2025;47:68–82.

81.	Wei K, Liao X, Yang T, He X, Yang D, Lai L, et al. Efficacy of probiotic supplementation in the treatment of psoriasis—A systematic review and meta‐analysis. J Cosmet Dermatol. 2024;23(7):2361–7.

82.	Lu L, Xu Y, Shi M, Liu A. Psychosocial interventions for psoriasis: a Bayesian network meta-analysis. J Dermatol Treat. 2025;36(1):2427321.

83.	Wei W, Zhang B, Liu T, Lu T. Effect of psychological intervention on quality of life among patients with psoriasis: a meta-analysis. Int J Behav Med. 2024;31(6):911–22.

## QUESTION 17. What are the efficacy and safety of biologic therapies in special situations?

**Psoriasis and pregnancy**

Evidence regarding the safety of biologics in women with psoriasis before and during pregnancy comes mainly from a systematic review [61] dof observational studies, which did not identify an increased risk of spontaneous abortion or congenital malformations compared with the general population. Most exposures occurred during the first trimester. Certainty of evidence is low, and data for newer biologics remain limited.

Clinical guidelines generally recommend anti–TNF agents as the safest option, particularly certolizumab pegol, due to minimal placental transfer, both when planning pregnancy and during pregnancy and lactation [62]. Other TNF inhibitors can be considered later in pregnancy after individualized risk–benefit assessment. Global WHO pharmacovigilance data[63] (1968–2024), including 6,518 reports of pregnancies exposed to biologics for psoriasis, suggest an overall favorable safety profile for IL-12/23, IL-17, and IL-23 inhibitors, although a potential signal of increased risk with risankizumab was identified and requires confirmation. Overall, while findings are reassuring, important evidence gaps remain and decisions should be individualized.

Risk of neonatal outcomes in psoriasis patients treated with biologics

| **Outcome** | **Risk with biologics (95% CI)** | **Comparison with general population** | **No. studies / pregnancies** | **Certainty of evidence (GRADE)** | **Comments** |
| --- | --- | --- | --- | --- | --- |
| Spontaneous abortion | 15.3% (12.7–18.0) | Similar (10–20%) | 50 studies / 689 pregnancies | ⨁◯◯◯ Very low | Observational; risk of bias; no controls. |
| Elective abortion | 10.8% (7.7–14.3) | Similar to population registries | 50 studies / 689 pregnancies | ⨁◯◯◯ Very low | Higher frequency with ixekizumab/tildrakizumab; potential bias. |
| Congenital malformations | 3.0% (1.6–4.8) | Comparable (2–5.5%) | 24 studies / 492 live births | ⨁◯◯◯ Very low | Few events; heterogeneous definitions; possible publication bias. |
| Preterm birth | 14.8% (42/283) | Higher than European population (5–9%) | 36 studies / 283 pregnancies | ⨁◯◯◯ Very low | Relationship to disease activity/comorbidities unclear; linkage to biologics not established. |
| Low birth weight | 9.8% (16/163) | Similar (~10%) | 19 studies / 163 pregnancies | ⨁◯◯◯ Very low | Limited data; small sample sizes. |

***Explanations***

1. Small sample sizes, heterogeneity in outcome definitions, and limited neonatal follow-up.

**Psoriasis and cancer**

A retrospective real-world study [64] (2016–2024) including 20 patients with cancer reported that biologics—mostly IL-17 and IL-23 inhibitors—were effective for psoriasis control and did not show major safety signals, even with prior or concurrent malignancy. Complementing this, a systematic review and meta-analysis[65] (31 studies; 24,328 patients; 85,784 person-years) found similar rates of cancer recurrence or new cancers among patients not receiving immunosuppression and those treated with TNF inhibitors, immunomodulators, or combinations. Ustekinumab and vedolizumab showed numerically lower rates, and timing of immunosuppression initiation (before vs after five years from the index cancer) did not modify risk.

In contrast, evidence for JAK inhibitors supports a more cautious approach. A meta-analysis[66], including more than 78 clinical trials and extension studies found no increase in malignancy risk compared with placebo or methotrexate, but a higher cancer incidence compared with TNF inhibitors. Malignancy events were uncommon across all groups.

Risk of malignancy in psoriasis patients treated with JAK inhibitors (includes trials and long-term extensions)

| **Comparison** | **Rate ratio (95% CI)** | **Interpretation** | **Certainty of evidence (GRADE)** |
| --- | --- | --- | --- |
| TNFi vs placebo | 0.71 (0.43–1.18) | Trend toward lower incidence with TNFi | ⨁⨁◯◯ Low |
| Methotrexate vs placebo | 1.09 (0.53–2.27) | No meaningful difference | ⨁⨁◯◯ Low |
| JAKi vs placebo | 1.16 (0.75–1.80) | No meaningful difference | ⨁⨁◯◯ Low |
| Methotrexate vs TNFi | 1.53 (0.80–2.92) | Trend toward higher risk with MTX | ⨁⨁◯◯ Low |
| JAKi vs TNFi | 1.63 (1.27–2.09) | Higher incidence with JAKi | ⨁⨁⨁◯ Moderate |
| JAKi vs methotrexate | 1.06 (0.58–1.94) | No meaningful difference | ⨁⨁◯◯ Low |

**Psoriasis and hepatitis**

Hepatitis B virus (HBV) reactivation is a clinically relevant risk in psoriasis patients treated with biologics, especially TNF inhibitors, while IL-17 and IL-23 inhibitors appear to be associated with lower risk. A meta-analysis [67] (9 studies, 104 patients) found that with ustekinumab, reactivation occurred mainly in HBsAg-positive patients and less often in occult infection, although evidence is limited by the small number of cases.

Available data show increased reactivation risk in HBsAg-positive patients without antiviral prophylaxis, supporting preventive antiviral therapy [66]. In HBsAg-negative/anti-HBc–positive patients, risk is lower (4% for HBV and 7% for HCV) [67], with reports of severe outcomes. Overall, evidence supports universal HBV and HCV screening before initiating biologics, antiviral prophylaxis in HBsAg carriers, and close monitoring—ideally with hepatology or infectious diseases support—for patients with positive serologies.

Risk of HBV reactivation in psoriasis treated with biologics

| **Patient group** | **No. studies** | **Patients** | **Reactivation rate (%)** | **95% CI** | **Certainty of evidence (GRADE)** |
| --- | --- | --- | --- | --- | --- |
| HBsAg+ without antiviral prophylaxis | 5 | 25 | 21.2 | 9.4–41.0 | ⨁⨁⨁◯ Moderate |
| HBsAg+ with antiviral prophylaxis | 6 | 31 | 0 | 0 | ⨁⨁⨁◯ Moderate |
| HBsAg– / HBcAb+ without prophylaxis | 12 | 218 | 4.4 | 2.2–8.7 | ⨁⨁◯◯ Low |
| IL-12/23 inhibitors | 7 | 117 | 4.9 | 1.9–11.8 | ⨁⨁◯◯ Low |
| IL-17 inhibitors | 3 | 84 | 3.3 | 1.0–10.8 | ⨁⨁◯◯ Low |
| IL-23 inhibitors | 2 | 17 | 6.3 | 0.9–34.3 | ⨁◯◯◯ Very low |
| Asia | 7 | 142 | 4.5 | 1.9–10.6 | ⨁⨁◯◯ Low |
| Non-Asia | 5 | 76 | 4.3 | 1.4–12.5 | ⨁⨁◯◯ Low |
| HBsAb+ | 10 | 118 | 6.0 | 2.6–13.4 | ⨁⨁◯◯ Low |
| HBsAb– | 7 | 31 | 14.0 | 5.5–31.4 | ⨁⨁◯◯ Low |
| HCV | ≈6 | ≈160 | 0.07 | 0.02–0.14 | ⨁⨁◯◯ Low |

**Psoriasis and HIV**

Psoriasis prevalence in patients with HIV is similar to the general population (1–3%), but disease is often more severe and less responsive to conventional therapies. Traditional immunosuppressants such as methotrexate and cyclosporine are contraindicated because they can worsen immunosuppression. In this setting, apremilast is considered a safer alternative, although with limited efficacy. TNF inhibitors have shown favorable outcomes in case series and observational studies, with sustained efficacy and an acceptable safety profile, although opportunistic infections have been reported. Ustekinumab has also demonstrated good efficacy and safety, with reports of stable CD4+ counts. IL-17 and IL-23 inhibitors have likewise been effective and safe in published cases, without evidence of viral reactivation or serious adverse events, with particularly favorable results reported for risankizumab with follow-up up to 2 years.[68]

A meta-analysis [67] including 14 studies and 1,033 psoriasis patients with chronic viral infections reported that, in the HIV subgroup, the pooled reactivation rate under biologic therapy was 0.12 (95% CI 0.00–0.40), with a small number of severe cases. American Academy of Dermatology and National Psoriasis Foundation guidelines recommend preferential use of IL-17 inhibitors in patients with HIV who are on antiretroviral therapy with controlled viral load. In all cases, periodic monitoring of CD4+ counts and coordinated care with infectious diseases are recommended to optimize treatment safety.

HIV reactivation in psoriasis patients treated with biologics

| **Outcome** | **Risk with biologics** | **95% CI** | **No. studies / patients** | **Certainty of evidence (GRADE)** |
| --- | --- | --- | --- | --- |
| HIV reactivation in psoriasis patients treated with biologics | 0.12 | (0.00–0.40) | 14 studies (HIV subgroup; small n) | ⨁◯◯◯ Very low |

**Psoriasis and tuberculosis**

A recent meta-analysis [69] including 1,747 psoriasis patients with latent tuberculosis infection reported the following: the overall reactivation rate during biologic therapy was essentially zero (0.0%; 95% CI 0–0.21), with no differences between those who did or did not receive prophylaxis. Risk was higher in the TNF inhibitor subgroup (1.3%), while no cases were reported with IL-17 or IL-23 inhibitors. Ustekinumab can interfere with the Th1 response needed for granuloma formation, and reactivation cases have been reported with its use. In psoriasis patients with latent TB infection, current recommendations prioritize IL-17 or IL-23 inhibitors, both for individuals with higher exposure risk and for those with limitations to receiving prophylaxis. TNF inhibitors and ustekinumab should be avoided or discontinued in active TB, and restarted only after clinical control with close monitoring.

Reactivation of latent tuberculosis in psoriasis patients treated with biologics

| **Subgroup** | **Reactivation rate (%)** | **95% CI** | **Comparison / Evidence** | **Certainty (GRADE)** |
| --- | --- | --- | --- | --- |
| Overall (20 studies, n = 1747) | 0.0% | 0–0.21 | No meaningful heterogeneity; consistent findings | ⨁⨁⨁◯ Moderate |
| With prophylaxis | 0.0% | 0–0.21 | No difference vs no prophylaxis | ⨁⨁◯◯ Low |
| Without prophylaxis | 0.0% | 0–0.21 | Same risk as prophylaxis group | ⨁⨁◯◯ Low |
| TNF inhibitors | 1.3% | 0–5.8 | Higher risk than IL-17/23 (p < 0.01) | ⨁⨁⨁◯ Moderate |
| IL-17 inhibitors | 0% | 0–0.21 | No reactivations reported | ⨁⨁◯◯ Low |
| IL-23 inhibitors | 0% | 0–0.21 | No reactivations reported | ⨁⨁◯◯ Low |

Universal screening for latent tuberculosis infection and implementation of prophylaxis per local guidelines are mandatory before initiating these agents [70]. Cohort studies show that adherence to these measures reduces the incidence of active TB up to tenfold in patients treated with TNF inhibitors.

**References**

1.	Yasmeen N, Sawyer LM, Malottki K, Levin LÅ, Didriksen Apol E, Jemec GB. Targeted therapies for patients with moderate-to-severe psoriasis: a systematic review and network meta-analysis of PASI response at 1 year. J Dermatol Treat. 2022 Jan 2;33(1):204–18.

2.	Belinchón Romero I, Dauden E, Ferrándiz Foraster C, González-Cantero Á, Carrascosa Carrillo JM. Therapeutic goals and treatment response evaluation in moderate to severe psoriasis: an experts opinion document. Ann Med. 2021 Jan;53(1):1727–36.

3.	Galluzzo M, Talamonti M, Manfreda V, Silvaggio D, Tartaglia C, Roberto D, et al. Impact of secukinumab on patient-reported outcomes in moderate to severe plaque psoriasis: a review of clinical studies. Expert Opin Biol Ther. 2021 Feb 1;21(2):191–200.

4.	Rusk AM, Fleischer AB. In psoriasis treatment, greater improvement in skin severity predicts greater improvement in nail severity. J Dermatol Treat. 2021 Nov 17;32(8):894–7.

5.	Belinchon Romero I, Mateu Puchades A, Ribera Pibernat M, Ruiz Genao DianaP, De La Cueva Dobao P, Carrascosa JM. Criteria used to define tumor necrosis factor-alpha inhibitors failure in patients with moderate-to-severe psoriasis: a systematic literature review. Ann Med. 2023 Dec 12;55(1):1335–45.

6.	Gisondi P, Di Mercurio M, Idolazzi L, Girolomoni G. Concept of Remission in Chronic Plaque Psoriasis. J Rheumatol Suppl. 2015 Nov 1;93(0):57–60.

7.	Puig L. PASI 90 response: the new standard in therapeutic efficacy for psoriasis. J Eur Acad Dermatol Venereol. 2015 Apr;29(4):645–8.

8.	Boswell ND, Singla S, Gordon KB. Sequencing of Targeted Therapy in Psoriasis: Does it Matter? Am J Clin Dermatol. 2024 Sept;25(5):795–810.

9.	Thaçi D, Piaserico S, Gaarn Du Jardin K, Warren RB. Long‐term stability of PASI <3 response to tildrakizumab: Pooled analyses from reSURFACE 1 and reSURFACE 2 phase 3 trials through 5 years. J Eur Acad Dermatol Venereol [Internet]. 2023 Apr [cited 2024 Oct 4];37(4). Available from: https://onlinelibrary.wiley.com/doi/10.1111/jdv.18783

10.	Imafuku S, Ohata C, Okubo Y, Tobita R, Saeki H, Mabuchi T, et al. Effectiveness of brodalumab in achieving treatment satisfaction for patients with plaque psoriasis: The ProLOGUE study. J Dermatol Sci. 2022 Mar;105(3):176–84.

11.	Reich K, Mrowietz U, Menter A, Griffiths CEM, Bagel J, Strober B, et al. Effect of baseline disease severity on achievement of treatment target with apremilast: results from a pooled analysis. J Eur Acad Dermatol Venereol. 2021 Dec;35(12):2409–14.

12.	Campanati A, Diotallevi F, Radi G, Molinelli E, Brisigotti V, Martina E, et al. Psoriatic patients treated with secukinumab reach high levels of minimal disease activity: results from the SUPREME study. Eur J Dermatol. 2021 Oct;31(5):630–7.

13.	Lebwohl MG, Soliman AM, Yang H, Wang J, Koenigsberg SH, Anderson A, et al. 25572 Impact of risankizumab on the duration of PASI90 achievement: A post hoc analysis of 4 phase III clinical trials. J Am Acad Dermatol. 2021 Sept;85(3):AB62.

14.	Puig L, Zhu B, Burge R, Shrom D, Dong Y, Shen W, et al. Early Treatment Targets for Predicting Long-term Dermatology Life Quality Index Response in Patients with Moderate-to-Severe Plaque Psoriasis: A Post-hoc Analysis from a Long-term Clinical Study. J Clin Aesthetic Dermatol. 2020 Oct;13(10):18–22.

15.	Puig L, Dossenbach M, Berggren L, Ljungberg A, Zachariae C. Absolute and Relative Psoriasis Area and Severity Indices (PASI) for Comparison of the Efficacy of Ixekizumab to Etanercept and Placebo in Patients with Moderate-to-severe Plaque Psoriasis: An Integrated Analysis of UNCOVER-2 and UNCOVER-3 Outcomes. Acta Derm Venereol. 2019;99(11):971–7.

16.	Merola JF, Amato DA, See K, Burge R, Mallinckrodt C, Ojeh CK, et al. Evaluation of sPGA × BSA as an Outcome Measure and Treatment Target for Clinical Practice. J Invest Dermatol. 2018 Sept;138(9):1955–61.

17.	Elewski BE, Puig L, Mordin M, Gilloteau I, Sherif B, Fox T, et al. Psoriasis patients with psoriasis Area and Severity Index (PASI) 90 response achieve greater health-related quality-of-life improvements than those with PASI 75–89 response: results from two phase 3 studies of secukinumab. J Dermatol Treat. 2017 Aug 18;28(6):492–9.

18.	Schmitt J, Rosumeck S, Thomaschewski G, Sporbeck B, Haufe E, Nast A. Efficacy and safety of systemic treatments for moderate-to-severe psoriasis: meta-analysis of randomized controlled trials. Br J Dermatol. 2014 Feb;170(2):274–303.

19.	Li Z, Pei B, Lei T, Yao M, Niu Y, Zhou P, et al. Clinical features and effects of cyclosporine in the treatment of psoriasis: a systemic review and meta-analysis. Arch Dermatol Res. 2024 Oct 26;316(10):705.

20.	Kogan N, Raimondo N, Gusis SE, Izcovich A, Abarca Duran JA, Barahona‐Torres L, et al. Latin American clinical practice guidelines on the systemic treatment of psoriasis SOLAPSO–Sociedad Latinoamericana de Psoriasis (Latin American Psoriasis Society). Int J Dermatol. 2019;58:4–28.

21.	van Huizen AM, Sikkel R, Caron AG, Menting SP, Spuls PI. Methotrexate dosing regimen for plaque-type psoriasis: an update of a systematic review. J Dermatol Treat. 2022;33(8):3104–18.

22.	Hsieh TS, Tsai TF. Combination therapy for psoriasis with methotrexate and other oral disease-modifying antirheumatic drugs: a systematic review. Dermatol Ther. 2023;13(4):891–909.

23.	Cheng Y, Liu GJ, Zhang M. Narrow‐band ultraviolet B phototherapy versus broad‐band ultraviolet B or psoralen‐ultraviolet A photochemotherapy for psoriasis. Cochrane Database Syst Rev. 2013;(10):CD009481.

24.	Sbidian E, Chaimani A, Garcia-Doval I, Doney L, Dressler C, Hua C, et al. Systemic pharmacological treatments for chronic plaque psoriasis: a network meta-analysis. Cochrane Database Syst Rev. 2022 May 23;5(5):CD011535.

25.	Mahmoud A, Ahmed AAS, Naeem A, Abuelazm M, Elshinawy M, Hassan AR, et al. Tyrosine Kinase-2 Inhibitor (Deucravacitinib) for Psoriasis: A Systematic Review and Network Meta-Analysis of Randomized Controlled Trials. Int J Dermatol Venereol. 2024 Dec;7(4):216.

26.	Armstrong AW, Soliman AM, Betts KA, Wang Y, Gao Y, Stakias V, et al. Long-term benefit–risk profiles of treatments for moderate-to-severe plaque psoriasis: a network meta-analysis. Dermatol Ther. 2022;12(1):167–84.

27.	Piragine E, Petri D, Martelli A, Janowska A, Dini V, Romanelli M, et al. Adherence and persistence to biological drugs for psoriasis: systematic review with meta-analysis. J Clin Med. 2022;11(6):1506.

28.	Gottlieb AB, Kalb RE, Blauvelt A, Heffernan MP, Sofen HL, Ferris LK, et al. The efficacy and safety of infliximab in patients with plaque psoriasis who had an inadequate response to etanercept: results of a prospective, multicenter, open-label study. J Am Acad Dermatol. 2012 Oct;67(4):642–50.

29.	Yamauchi PS, Bissonnette R, Teixeira HD, Valdecantos WC. Systematic review of efficacy of anti-tumor necrosis factor (TNF) therapy in patients with psoriasis previously treated with a different anti-TNF agent. J Am Acad Dermatol. 2016 Sept;75(3):612-618.e6.

30.	Piaserico S, Cazzaniga S, Chimenti S, Giannetti A, Maccarone M, Picardo M, et al. Efficacy of switching between tumor necrosis factor-alfa inhibitors in psoriasis: results from the Italian Psocare registry. J Am Acad Dermatol. 2014 Feb;70(2):257-262.e3.

31.	Boswell ND, Singla S, Gordon KB. Sequencing of Targeted Therapy in Psoriasis: Does it Matter? Am J Clin Dermatol. 2024 Sept;25(5):795–810.

32.	Warren RB, Blauvelt A, Poulin Y, Beeck S, Kelly M, Wu T, et al. Efficacy and safety of risankizumab vs. secukinumab in patients with moderate-to-severe plaque psoriasis (IMMerge): results from a phase III, randomized, open-label, efficacy-assessor-blinded clinical trial. Br J Dermatol. 2021 Jan;184(1):50–9.

33.	Blauvelt A, Papp KA, Griffiths CEM, Randazzo B, Wasfi Y, Shen YK, et al. Efficacy and safety of guselkumab, an anti-interleukin-23 monoclonal antibody, compared with adalimumab for the continuous treatment of patients with moderate to severe psoriasis: Results from the phase III, double-blinded, placebo- and active comparator-controlled VOYAGE 1 trial. J Am Acad Dermatol. 2017 Mar;76(3):405–17.

34.	Reich K, Armstrong AW, Foley P, Song M, Wasfi Y, Randazzo B, et al. Efficacy and safety of guselkumab, an anti-interleukin-23 monoclonal antibody, compared with adalimumab for the treatment of patients with moderate to severe psoriasis with randomized withdrawal and retreatment: Results from the phase III, double-blind, placebo- and active comparator-controlled VOYAGE 2 trial. J Am Acad Dermatol. 2017 Mar;76(3):418–31.

35.	Reich K, Gooderham M, Thaçi D, Crowley JJ, Ryan C, Krueger JG, et al. Risankizumab compared with adalimumab in patients with moderate-to-severe plaque psoriasis (IMMvent): a randomised, double-blind, active-comparator-controlled phase 3 trial. Lancet Lond Engl. 2019 Aug 17;394(10198):576–86.

36.	Warren RB, Blauvelt A, Bagel J, Papp KA, Yamauchi P, Armstrong A, et al. Bimekizumab versus Adalimumab in Plaque Psoriasis. N Engl J Med. 2021 July 8;385(2):130–41.

37.	Warren RB, Barker JNWB, Finlay AY, Burden AD, Kirby B, Armendariz Y, et al. Secukinumab for patients failing previous tumour necrosis factor-α inhibitor therapy: results of a randomized open-label study (SIGNATURE). Br J Dermatol. 2020 July;183(1):60–70.

38.	Strober B, Paul C, Blauvelt A, Thaçi D, Puig L, Lebwohl M, et al. Bimekizumab efficacy and safety in patients with moderate to severe plaque psoriasis: Two-year interim results from the open-label extension of the randomized BE RADIANT phase 3b trial. J Am Acad Dermatol. 2023 Sept;89(3):486–95.

39.	Loft N, Halling AS, Egeberg A, Skov L. Efficacy of a second interleukin 17 inhibitor in patients with psoriasis: A systematic review and meta-analysis. J Am Acad Dermatol. 2021 Jan;84(1):130–8.

40.	Loft N, Egeberg A, Isufi D, Rasmussen MK, Bryld LE, Dam TN, et al. Response to Interleukin-17A Inhibitors According to Prior Biologic Exposures: A Danish Nationwide Study. Acta Derm Venereol. 2023 Nov 21;103:adv12616.

41.	Papp K, Blauvelt A, Sullivan J, Tada Y, Polzer P, Mallbris L, et al. Ixekizumab—An Effective and Safe Treatment for Moderate-to-Severe Plaque Psoriasis in Patients Previously Treated With Other IL-17 Inhibitors: Results From IXORA-P. J Psoriasis Psoriatic Arthritis®. 2019 Oct 1;4(4):180–5.

42.	Reddy R, Pannu S, Fiumara K, Kahn J, Rosmarin D. Efficacy of in-class interleukin-23 inhibitor switching: risankizumab following guselkumab failure in moderate-to-severe psoriasis treatment. Br J Dermatol. 2021 Mar;184(3):559–61.

43.	Gasslitter I, Kirsten N, Augustin M, Torz K, Mrowietz U, Eyerich K, et al. Successful intra-class switching among IL-17 antagonists: a multicentre, multinational, retrospective study. Arch Dermatol Res. 2019 July;311(5):421–4.

44.	Daniele SG, Eldirany SA, Damiani G, Ho M, Bunick CG. Structural Basis for p19 Targeting by Anti-IL-23 Biologics: Correlations with Short- and Long-Term Efficacy in Psoriasis. JID Innov Skin Sci Mol Popul Health. 2024 Mar;4(2):100261.

45.	Camela E, Ocampo‐Garza SS, Cinelli E, Villani A, Fabbrocini G, Megna M. Therapeutic update of biologics and small molecules for scalp psoriasis: a systematic review. Dermatol Ther. 2021;34(2):e14857.

46.	Mosca M, Hong J, Hadeler E, Brownstone N, Bhutani T, Liao W. Scalp psoriasis: a literature review of effective therapies and updated recommendations for practical management. Dermatol Ther. 2021;11(3):769–97.

47.	Tsiogkas SG, Karamitrou EK, Grammatikopoulou MG, Zafiriou E, Bogdanos DP. Efficacy of tyrosine-kinase-2 and phosphodiesterase-4 inhibitors for scalp psoriasis: a systematic review and meta-analysis. Curr Med Res Opin. 2024;40(2):155–63.

48.	Duffin KC, Griffiths CE, Hoffmann M, Blauvelt A, Balagula E, Napoli A, et al. Deucravacitinib, an Oral Selective Tyrosine Kinase 2 (TYK2) Inhibitor, in Patients with Moderate to Severe Scalp Psoriasis: Efficacy and Safety Results of a Phase 3b/4, Multicenter, Randomized, Double-blinded, Placebo-controlled Trial (PSORIATYK SCALP). SKIN J Cutan Med. 2024;8(6):s420–s420.

49.	Khan M, Wallace CE, Ahmed F, Rahman SM, Memon N, Haque A. Assessing Comparative Efficacy of Biologics for the Treatment of Psoriasis With Nail Involvement: A Systematic Review. J Psoriasis Psoriatic Arthritis®. 2024;9(2):61–8.

50.	Hwang JK, Ricardo JW, Lipner SR. Efficacy and safety of nail psoriasis targeted therapies: a systematic review. Am J Clin Dermatol. 2023;24(5):695–720.

51.	Hong JJ, Mosca ML, Hadeler EK, Brownstone ND, Bhutani T, Liao WJ. Genital and inverse/intertriginous psoriasis: an updated review of therapies and recommendations for practical management. Dermatol Ther. 2021;11(3):833–44.

52.	Beck KM, Yang EJ, Sanchez IM, Liao W. Treatment of Genital Psoriasis: A Systematic Review. Dermatol Ther. 2018 Dec;8(4):509–25.

53.	Tsiogkas SG, Grammatikopoulou MG, Kontouli KM, Minopoulou I, Goulis DD, Zafiriou E, et al. Efficacy of biologic agents for palmoplantar psoriasis: a systematic review and network meta-analysis. Expert Rev Clin Immunol. 2023;19(12):1485–98.

54.	Huang IH, Wu PC, Chiu HY, Huang YH. Small-molecule inhibitors and biologics for palmoplantar psoriasis and palmoplantar pustulosis: a systematic review and network meta-analysis. Am J Clin Dermatol. 2024;25(3):347–58.

55.	Xia R, Diao Z, Chen D, Wang Y, Zhou C, Gao Y, et al. Efficacy of anti-interleukin-17A biological agents for palmoplantar psoriasis and palmoplantar pustulosis: A network meta-analysis. Int Immunopharmacol. 2024;130:111716.

56.	Reynolds KA, Pithadia DJ, Lee EB, Liao W, Wu JJ. A systematic review of treatment strategies for erythrodermic psoriasis. J Dermatol Treat. 2021 Feb;32(1):49–55.

57.	Lo Y, Tsai TF. Updates on the treatment of erythrodermic psoriasis. Psoriasis Targets Ther. 2021;59–73.

58.	Zhou T, Koussiouris J, Kim L, Vender R. Management of guttate psoriasis: a systematic review. J Cutan Med Surg. 2024;28(6):577–84.

59.	Rega F, Trovato F, Bortone G, Pellacani G, Richetta AG, Dattola A. Therapeutic potential of Spesolimab-Sbzo in the management of generalized pustular psoriasis flares in adults: evidence to date. Psoriasis Targets Ther. 2024;23–7.

60.	Puig L, Fujita H, Thaçi D, Zheng M, Hernandez Daly AC, Leonardi C, et al. Current treatments for generalized pustular psoriasis: a narrative summary of a systematic literature search. Dermatol Ther. 2024;14(9):2331–78.

61.	Chen B lin, Liu Q wei, Dong X wan, Bai Y ping. Biologics for generalized pustular psoriasis: a systematic review and single-arm meta-analysis. Front Immunol. 2024;15:1462158.

62.	Kearns DG, Chat VS, Zang PD, Han G, Wu JJ. Review of treatments for generalized pustular psoriasis. J Dermatol Treat. 2021 July 4;32(5):492–4.

63.	Sánchez‐García V, Hernández‐Quiles R, de‐Miguel‐Balsa E, Giménez‐Richarte Á, Ramos‐Rincón J, Belinchón‐Romero I. Exposure to biologic therapy before and during pregnancy in patients with psoriasis: systematic review and meta‐analysis. J Eur Acad Dermatol Venereol. 2023;37(10):1971–90.

64.	Owczarek W, Walecka I, Lesiak A, Czajkowski R, Reich A, Zerda I, et al. The use of biological drugs in psoriasis patients prior to pregnancy, during pregnancy and lactation: a review of current clinical guidelines. Adv Dermatol Allergol Dermatol Alergol. 2020;37(6):821–30.

65.	Jeong YD, Jo H, Cho H, Jang W, Park J, Lee S, et al. Biologics use for psoriasis during pregnancy and its related adverse outcomes in pregnant women and newborns: Findings from WHO pharmacovigilance study. Int Arch Allergy Immunol. 2025;186(6):579–93.

66.	Battista T, Gallo L, Martora F, Fattore D, Potestio L, Cacciapuoti S, et al. Biological therapy for psoriasis in cancer patients: an 8-year retrospective real-life study. J Clin Med. 2024;13(7):1940.

67.	Gupta A, Peyrin-Biroulet L, Ananthakrishnan AN. Risk of cancer recurrence in patients with immune-mediated diseases with use of immunosuppressive therapies: an updated systematic review and meta-analysis. Clin Gastroenterol Hepatol. 2024;22(3):499–512.

68.	Russell MD, Stovin C, Alveyn E, Adeyemi O, Chan CKD, Patel V, et al. JAK inhibitors and the risk of malignancy: a meta-analysis across disease indications. Ann Rheum Dis. 2023;82(8):1059–67.

69.	Alhalabi MM, Almokdad R. The incidence of hepatitis B reactivation in patients receiving ustekinumab: a systematic review and proportional meta-analysis. Eur J Gastroenterol Hepatol. 2025;37(1):1–9.

70.	Kuo MH, Ko PH, Wang ST, Tseng CW. Incidence of HBV Reactivation in Psoriasis Patients Undergoing Cytokine Inhibitor Therapy: A Single-Center Study and Systematic Review with a Meta-Analysis. Viruses. 2024;17(1):42.

71.	Li L, Jiang X, Fu L, Zhang L, Feng Y. Reactivation rates of hepatitis B or C or HIV in patients with psoriasis using biological therapies: a systematic review and meta-analysis. Clin Exp Med. 2023;23(3):701–15.

72.	Megna M, Lauletta G, Tommasino N, Salsano A, Battista T, Ruggiero A, et al. Management of psoriasis patients with serious infectious diseases. Adv Ther. 2024;41(6):2099–111.

73.	Zhu X, Pan X, Da M, Wang F, Dong Z. Risk for latent tuberculosis infection reactivation among patients with psoriasis on biologics treatment: a meta-analysis. J Infect. 2024;89(3).

74.	Mateu-Arrom L, Puig L. Infection Risk and Vaccination in the Management of Psoriasis: Considerations for Biologic Therapy. Psoriasis Targets Ther. 2025;127–44.

75.	Jafarzadeh A, Behrangi E, Khosravi M, Falakeh S, Amiri JK, Goodarzi A. Emerging treatments for dermatologic diseases in infants, children, and adolescents: a systematic review of clinical trials on biologics and small molecule inhibitors. Inflammopharmacology. 2025;1–56.

76.	Sticherling M, Nikkels AF, Hamza AM, Kwong P, Szepietowski JC, El Sayed M, et al. Secukinumab in pediatric patients with plaque psoriasis: pooled safety analysis from two phase 3 randomized clinical trials. Am J Clin Dermatol. 2023;24(5):821–35.

77.	Li C, Sunhe Y, Zhou H, Dong W. Efficacy and safety evaluations of adalimumab biosimilars in the treatment of psoriasis. J Dermatol Treat. 2023;34(1):2249145.

78.	Phan DB, Elyoussfi S, Stevenson M, Lunt M, Warren RB, Yiu ZZ. Biosimilars for the treatment of psoriasis: a systematic review of clinical trials and observational studies. JAMA Dermatol. 2023;159(7):763–71.

79.	Macaluso FS, Cummings JF, Atreya R, Choi J, Orlando A. A systematic review on infliximab biosimilar SB2: from pre-clinical data to real-world evidence. Expert Opin Biol Ther. 2022;22(2):203–23.

80.	Wang Q, Wang J, Sun X, Liu L, Zhang M, Yu Y, et al. Evidence-based dietary recommendations for patients with psoriasis: A systematic review. Clin Nutr. 2025;47:68–82.

81.	Wei K, Liao X, Yang T, He X, Yang D, Lai L, et al. Efficacy of probiotic supplementation in the treatment of psoriasis—A systematic review and meta‐analysis. J Cosmet Dermatol. 2024;23(7):2361–7.

82.	Lu L, Xu Y, Shi M, Liu A. Psychosocial interventions for psoriasis: a Bayesian network meta-analysis. J Dermatol Treat. 2025;36(1):2427321.

83.	Wei W, Zhang B, Liu T, Lu T. Effect of psychological intervention on quality of life among patients with psoriasis: a meta-analysis. Int J Behav Med. 2024;31(6):911–22.

# QUESTION 18. What are the efficacy and safety of biologic therapies for pediatric plaque psoriasis?

Evidence on the use of biologics and small molecules in pediatric psoriasis shows consistent efficacy with an acceptable safety profile. According to a recent meta-analysis [73], among JAK inhibitors, tofacitinib achieved PASI75 responses in 55% of patients at week 12 and 70% at week 36 in a clinical trial. Among TNF inhibitors, adalimumab was superior to methotrexate, with PASI75 rates up to 58% at week 16, while etanercept achieved PASI75 responses of 55%–57% versus 11% with placebo at week 12, with early improvements in quality of life and responses maintained for more than 5 years.

Among IL-17 and IL-23 inhibitors, secukinumab showed strong responses, with PASI75 above 90% and PASI90 around 70% at week 12, maintaining efficacy through 104 weeks and showing superior outcomes to etanercept. Secukinumab has also demonstrated good tolerability in pediatric patients with moderate-to-severe plaque psoriasis across age and body-weight subgroups [71]. Ustekinumab achieved PASI75 rates close to 80% and PGA 0/1 responses in approximately 69% of adolescents at week 12. Ixekizumab demonstrated high response rates, with PASI75 in 90%, PASI90 in 78%, and PASI100 in 50% at week 12, with effectiveness maintained through week 108.

Finally, the PDE-4 inhibitor apremilast showed more modest efficacy, with PASI75 of 45% versus 16% with placebo at week 16, and a favorable safety profile, though with lower potency compared with biologics.

Overall, the evidence supports biologics—particularly IL-17 and IL-23 inhibitors and TNF inhibitors—as effective options for pediatric psoriasis. Outcomes are summarized in the GRADE table.

Risk of neonatal outcomes in psoriasis patients treated with biologics

| **Intervention** | **Primary outcome (psoriasis)** | **Key results** | **No. studies / patients** | **Certainty of evidence (GRADE)** | **Comments** |
| --- | --- | --- | --- | --- | --- |
| Tofacitinib (JAKi) | PASI75 | 55% at week 12; 70% at week 36 | 1 trial; ~30 patients | ⨁⨁◯◯ Low | High efficacy; limited evidence |
| Adalimumab (TNFi) | PASI75 | 58% (0.8 mg/kg) vs 32% MTX (week 16) | 2 RCTs; >200 patients | ⨁⨁⨁◯ Moderate | Superior to MTX; sustained efficacy in extension studies |
| Etanercept (TNFi) | PASI75, CDLQI | 55%–57% PASI75 vs 11% placebo (week 12); quality-of-life improvement | 5 pediatric RCTs | ⨁⨁⨁⨁ High | Responses maintained through 264 weeks |
| Secukinumab (anti–IL-17) | PASI75/90/100, IGA 0/1 | PASI75 >90%; PASI90 ~70%; superior to etanercept at week 52 | 3 RCTs; >200 patients | ⨁⨁⨁◯ Moderate | Strong and sustained efficacy through 104 weeks |
| Ustekinumab (anti–IL-12/23) | PASI75/90, PGA 0/1 | PASI75 ~80%; PGA 0/1 ~69% at week 12 | 1 RCT (Landells 2015) | ⨁⨁◯◯ Low | Limited but consistent data |
| Ixekizumab (anti–IL-17) | PASI75/90/100, sPGA 0/1 | PASI75 ~90%; PASI90 ~78%; PASI100 ~50% | 2 RCTs; >150 patients | ⨁⨁⨁◯ Moderate | Sustained improvement through week 108; favorable safety profile |
| Apremilast (PDE-4) | PASI75/90, PASI reduction | PASI75 45% vs 16% placebo (week 16) | 2 pediatric RCTs | ⨁⨁⨁◯ Moderate | Favorable safety profile; lower efficacy vs biologics |

**References**

1.	Yasmeen N, Sawyer LM, Malottki K, Levin LÅ, Didriksen Apol E, Jemec GB. Targeted therapies for patients with moderate-to-severe psoriasis: a systematic review and network meta-analysis of PASI response at 1 year. J Dermatol Treat. 2022 Jan 2;33(1):204–18.

2.	Belinchón Romero I, Dauden E, Ferrándiz Foraster C, González-Cantero Á, Carrascosa Carrillo JM. Therapeutic goals and treatment response evaluation in moderate to severe psoriasis: an experts opinion document. Ann Med. 2021 Jan;53(1):1727–36.

3.	Galluzzo M, Talamonti M, Manfreda V, Silvaggio D, Tartaglia C, Roberto D, et al. Impact of secukinumab on patient-reported outcomes in moderate to severe plaque psoriasis: a review of clinical studies. Expert Opin Biol Ther. 2021 Feb 1;21(2):191–200.

4.	Rusk AM, Fleischer AB. In psoriasis treatment, greater improvement in skin severity predicts greater improvement in nail severity. J Dermatol Treat. 2021 Nov 17;32(8):894–7.

5.	Belinchon Romero I, Mateu Puchades A, Ribera Pibernat M, Ruiz Genao DianaP, De La Cueva Dobao P, Carrascosa JM. Criteria used to define tumor necrosis factor-alpha inhibitors failure in patients with moderate-to-severe psoriasis: a systematic literature review. Ann Med. 2023 Dec 12;55(1):1335–45.

6.	Gisondi P, Di Mercurio M, Idolazzi L, Girolomoni G. Concept of Remission in Chronic Plaque Psoriasis. J Rheumatol Suppl. 2015 Nov 1;93(0):57–60.

7.	Puig L. PASI 90 response: the new standard in therapeutic efficacy for psoriasis. J Eur Acad Dermatol Venereol. 2015 Apr;29(4):645–8.

8.	Boswell ND, Singla S, Gordon KB. Sequencing of Targeted Therapy in Psoriasis: Does it Matter? Am J Clin Dermatol. 2024 Sept;25(5):795–810.

9.	Thaçi D, Piaserico S, Gaarn Du Jardin K, Warren RB. Long‐term stability of PASI <3 response to tildrakizumab: Pooled analyses from reSURFACE 1 and reSURFACE 2 phase 3 trials through 5 years. J Eur Acad Dermatol Venereol [Internet]. 2023 Apr [cited 2024 Oct 4];37(4). Available from: https://onlinelibrary.wiley.com/doi/10.1111/jdv.18783

10.	Imafuku S, Ohata C, Okubo Y, Tobita R, Saeki H, Mabuchi T, et al. Effectiveness of brodalumab in achieving treatment satisfaction for patients with plaque psoriasis: The ProLOGUE study. J Dermatol Sci. 2022 Mar;105(3):176–84.

11.	Reich K, Mrowietz U, Menter A, Griffiths CEM, Bagel J, Strober B, et al. Effect of baseline disease severity on achievement of treatment target with apremilast: results from a pooled analysis. J Eur Acad Dermatol Venereol. 2021 Dec;35(12):2409–14.

12.	Campanati A, Diotallevi F, Radi G, Molinelli E, Brisigotti V, Martina E, et al. Psoriatic patients treated with secukinumab reach high levels of minimal disease activity: results from the SUPREME study. Eur J Dermatol. 2021 Oct;31(5):630–7.

13.	Lebwohl MG, Soliman AM, Yang H, Wang J, Koenigsberg SH, Anderson A, et al. 25572 Impact of risankizumab on the duration of PASI90 achievement: A post hoc analysis of 4 phase III clinical trials. J Am Acad Dermatol. 2021 Sept;85(3):AB62.

14.	Puig L, Zhu B, Burge R, Shrom D, Dong Y, Shen W, et al. Early Treatment Targets for Predicting Long-term Dermatology Life Quality Index Response in Patients with Moderate-to-Severe Plaque Psoriasis: A Post-hoc Analysis from a Long-term Clinical Study. J Clin Aesthetic Dermatol. 2020 Oct;13(10):18–22.

15.	Puig L, Dossenbach M, Berggren L, Ljungberg A, Zachariae C. Absolute and Relative Psoriasis Area and Severity Indices (PASI) for Comparison of the Efficacy of Ixekizumab to Etanercept and Placebo in Patients with Moderate-to-severe Plaque Psoriasis: An Integrated Analysis of UNCOVER-2 and UNCOVER-3 Outcomes. Acta Derm Venereol. 2019;99(11):971–7.

16.	Merola JF, Amato DA, See K, Burge R, Mallinckrodt C, Ojeh CK, et al. Evaluation of sPGA × BSA as an Outcome Measure and Treatment Target for Clinical Practice. J Invest Dermatol. 2018 Sept;138(9):1955–61.

17.	Elewski BE, Puig L, Mordin M, Gilloteau I, Sherif B, Fox T, et al. Psoriasis patients with psoriasis Area and Severity Index (PASI) 90 response achieve greater health-related quality-of-life improvements than those with PASI 75–89 response: results from two phase 3 studies of secukinumab. J Dermatol Treat. 2017 Aug 18;28(6):492–9.

18.	Schmitt J, Rosumeck S, Thomaschewski G, Sporbeck B, Haufe E, Nast A. Efficacy and safety of systemic treatments for moderate-to-severe psoriasis: meta-analysis of randomized controlled trials. Br J Dermatol. 2014 Feb;170(2):274–303.

19.	Li Z, Pei B, Lei T, Yao M, Niu Y, Zhou P, et al. Clinical features and effects of cyclosporine in the treatment of psoriasis: a systemic review and meta-analysis. Arch Dermatol Res. 2024 Oct 26;316(10):705.

20.	Kogan N, Raimondo N, Gusis SE, Izcovich A, Abarca Duran JA, Barahona‐Torres L, et al. Latin American clinical practice guidelines on the systemic treatment of psoriasis SOLAPSO–Sociedad Latinoamericana de Psoriasis (Latin American Psoriasis Society). Int J Dermatol. 2019;58:4–28.

21.	van Huizen AM, Sikkel R, Caron AG, Menting SP, Spuls PI. Methotrexate dosing regimen for plaque-type psoriasis: an update of a systematic review. J Dermatol Treat. 2022;33(8):3104–18.

22.	Hsieh TS, Tsai TF. Combination therapy for psoriasis with methotrexate and other oral disease-modifying antirheumatic drugs: a systematic review. Dermatol Ther. 2023;13(4):891–909.

23.	Cheng Y, Liu GJ, Zhang M. Narrow‐band ultraviolet B phototherapy versus broad‐band ultraviolet B or psoralen‐ultraviolet A photochemotherapy for psoriasis. Cochrane Database Syst Rev. 2013;(10):CD009481.

24.	Sbidian E, Chaimani A, Garcia-Doval I, Doney L, Dressler C, Hua C, et al. Systemic pharmacological treatments for chronic plaque psoriasis: a network meta-analysis. Cochrane Database Syst Rev. 2022 May 23;5(5):CD011535.

25.	Mahmoud A, Ahmed AAS, Naeem A, Abuelazm M, Elshinawy M, Hassan AR, et al. Tyrosine Kinase-2 Inhibitor (Deucravacitinib) for Psoriasis: A Systematic Review and Network Meta-Analysis of Randomized Controlled Trials. Int J Dermatol Venereol. 2024 Dec;7(4):216.

26.	Armstrong AW, Soliman AM, Betts KA, Wang Y, Gao Y, Stakias V, et al. Long-term benefit–risk profiles of treatments for moderate-to-severe plaque psoriasis: a network meta-analysis. Dermatol Ther. 2022;12(1):167–84.

27.	Piragine E, Petri D, Martelli A, Janowska A, Dini V, Romanelli M, et al. Adherence and persistence to biological drugs for psoriasis: systematic review with meta-analysis. J Clin Med. 2022;11(6):1506.

28.	Gottlieb AB, Kalb RE, Blauvelt A, Heffernan MP, Sofen HL, Ferris LK, et al. The efficacy and safety of infliximab in patients with plaque psoriasis who had an inadequate response to etanercept: results of a prospective, multicenter, open-label study. J Am Acad Dermatol. 2012 Oct;67(4):642–50.

29.	Yamauchi PS, Bissonnette R, Teixeira HD, Valdecantos WC. Systematic review of efficacy of anti-tumor necrosis factor (TNF) therapy in patients with psoriasis previously treated with a different anti-TNF agent. J Am Acad Dermatol. 2016 Sept;75(3):612-618.e6.

30.	Piaserico S, Cazzaniga S, Chimenti S, Giannetti A, Maccarone M, Picardo M, et al. Efficacy of switching between tumor necrosis factor-alfa inhibitors in psoriasis: results from the Italian Psocare registry. J Am Acad Dermatol. 2014 Feb;70(2):257-262.e3.

31.	Boswell ND, Singla S, Gordon KB. Sequencing of Targeted Therapy in Psoriasis: Does it Matter? Am J Clin Dermatol. 2024 Sept;25(5):795–810.

32.	Warren RB, Blauvelt A, Poulin Y, Beeck S, Kelly M, Wu T, et al. Efficacy and safety of risankizumab vs. secukinumab in patients with moderate-to-severe plaque psoriasis (IMMerge): results from a phase III, randomized, open-label, efficacy-assessor-blinded clinical trial. Br J Dermatol. 2021 Jan;184(1):50–9.

33.	Blauvelt A, Papp KA, Griffiths CEM, Randazzo B, Wasfi Y, Shen YK, et al. Efficacy and safety of guselkumab, an anti-interleukin-23 monoclonal antibody, compared with adalimumab for the continuous treatment of patients with moderate to severe psoriasis: Results from the phase III, double-blinded, placebo- and active comparator-controlled VOYAGE 1 trial. J Am Acad Dermatol. 2017 Mar;76(3):405–17.

34.	Reich K, Armstrong AW, Foley P, Song M, Wasfi Y, Randazzo B, et al. Efficacy and safety of guselkumab, an anti-interleukin-23 monoclonal antibody, compared with adalimumab for the treatment of patients with moderate to severe psoriasis with randomized withdrawal and retreatment: Results from the phase III, double-blind, placebo- and active comparator-controlled VOYAGE 2 trial. J Am Acad Dermatol. 2017 Mar;76(3):418–31.

35.	Reich K, Gooderham M, Thaçi D, Crowley JJ, Ryan C, Krueger JG, et al. Risankizumab compared with adalimumab in patients with moderate-to-severe plaque psoriasis (IMMvent): a randomised, double-blind, active-comparator-controlled phase 3 trial. Lancet Lond Engl. 2019 Aug 17;394(10198):576–86.

36.	Warren RB, Blauvelt A, Bagel J, Papp KA, Yamauchi P, Armstrong A, et al. Bimekizumab versus Adalimumab in Plaque Psoriasis. N Engl J Med. 2021 July 8;385(2):130–41.

37.	Warren RB, Barker JNWB, Finlay AY, Burden AD, Kirby B, Armendariz Y, et al. Secukinumab for patients failing previous tumour necrosis factor-α inhibitor therapy: results of a randomized open-label study (SIGNATURE). Br J Dermatol. 2020 July;183(1):60–70.

38.	Strober B, Paul C, Blauvelt A, Thaçi D, Puig L, Lebwohl M, et al. Bimekizumab efficacy and safety in patients with moderate to severe plaque psoriasis: Two-year interim results from the open-label extension of the randomized BE RADIANT phase 3b trial. J Am Acad Dermatol. 2023 Sept;89(3):486–95.

39.	Loft N, Halling AS, Egeberg A, Skov L. Efficacy of a second interleukin 17 inhibitor in patients with psoriasis: A systematic review and meta-analysis. J Am Acad Dermatol. 2021 Jan;84(1):130–8.

40.	Loft N, Egeberg A, Isufi D, Rasmussen MK, Bryld LE, Dam TN, et al. Response to Interleukin-17A Inhibitors According to Prior Biologic Exposures: A Danish Nationwide Study. Acta Derm Venereol. 2023 Nov 21;103:adv12616.

41.	Papp K, Blauvelt A, Sullivan J, Tada Y, Polzer P, Mallbris L, et al. Ixekizumab—An Effective and Safe Treatment for Moderate-to-Severe Plaque Psoriasis in Patients Previously Treated With Other IL-17 Inhibitors: Results From IXORA-P. J Psoriasis Psoriatic Arthritis®. 2019 Oct 1;4(4):180–5.

42.	Reddy R, Pannu S, Fiumara K, Kahn J, Rosmarin D. Efficacy of in-class interleukin-23 inhibitor switching: risankizumab following guselkumab failure in moderate-to-severe psoriasis treatment. Br J Dermatol. 2021 Mar;184(3):559–61.

43.	Gasslitter I, Kirsten N, Augustin M, Torz K, Mrowietz U, Eyerich K, et al. Successful intra-class switching among IL-17 antagonists: a multicentre, multinational, retrospective study. Arch Dermatol Res. 2019 July;311(5):421–4.

44.	Daniele SG, Eldirany SA, Damiani G, Ho M, Bunick CG. Structural Basis for p19 Targeting by Anti-IL-23 Biologics: Correlations with Short- and Long-Term Efficacy in Psoriasis. JID Innov Skin Sci Mol Popul Health. 2024 Mar;4(2):100261.

45.	Camela E, Ocampo‐Garza SS, Cinelli E, Villani A, Fabbrocini G, Megna M. Therapeutic update of biologics and small molecules for scalp psoriasis: a systematic review. Dermatol Ther. 2021;34(2):e14857.

46.	Mosca M, Hong J, Hadeler E, Brownstone N, Bhutani T, Liao W. Scalp psoriasis: a literature review of effective therapies and updated recommendations for practical management. Dermatol Ther. 2021;11(3):769–97.

47.	Tsiogkas SG, Karamitrou EK, Grammatikopoulou MG, Zafiriou E, Bogdanos DP. Efficacy of tyrosine-kinase-2 and phosphodiesterase-4 inhibitors for scalp psoriasis: a systematic review and meta-analysis. Curr Med Res Opin. 2024;40(2):155–63.

48.	Duffin KC, Griffiths CE, Hoffmann M, Blauvelt A, Balagula E, Napoli A, et al. Deucravacitinib, an Oral Selective Tyrosine Kinase 2 (TYK2) Inhibitor, in Patients with Moderate to Severe Scalp Psoriasis: Efficacy and Safety Results of a Phase 3b/4, Multicenter, Randomized, Double-blinded, Placebo-controlled Trial (PSORIATYK SCALP). SKIN J Cutan Med. 2024;8(6):s420–s420.

49.	Khan M, Wallace CE, Ahmed F, Rahman SM, Memon N, Haque A. Assessing Comparative Efficacy of Biologics for the Treatment of Psoriasis With Nail Involvement: A Systematic Review. J Psoriasis Psoriatic Arthritis®. 2024;9(2):61–8.

50.	Hwang JK, Ricardo JW, Lipner SR. Efficacy and safety of nail psoriasis targeted therapies: a systematic review. Am J Clin Dermatol. 2023;24(5):695–720.

51.	Hong JJ, Mosca ML, Hadeler EK, Brownstone ND, Bhutani T, Liao WJ. Genital and inverse/intertriginous psoriasis: an updated review of therapies and recommendations for practical management. Dermatol Ther. 2021;11(3):833–44.

52.	Beck KM, Yang EJ, Sanchez IM, Liao W. Treatment of Genital Psoriasis: A Systematic Review. Dermatol Ther. 2018 Dec;8(4):509–25.

53.	Tsiogkas SG, Grammatikopoulou MG, Kontouli KM, Minopoulou I, Goulis DD, Zafiriou E, et al. Efficacy of biologic agents for palmoplantar psoriasis: a systematic review and network meta-analysis. Expert Rev Clin Immunol. 2023;19(12):1485–98.

54.	Huang IH, Wu PC, Chiu HY, Huang YH. Small-molecule inhibitors and biologics for palmoplantar psoriasis and palmoplantar pustulosis: a systematic review and network meta-analysis. Am J Clin Dermatol. 2024;25(3):347–58.

55.	Xia R, Diao Z, Chen D, Wang Y, Zhou C, Gao Y, et al. Efficacy of anti-interleukin-17A biological agents for palmoplantar psoriasis and palmoplantar pustulosis: A network meta-analysis. Int Immunopharmacol. 2024;130:111716.

56.	Reynolds KA, Pithadia DJ, Lee EB, Liao W, Wu JJ. A systematic review of treatment strategies for erythrodermic psoriasis. J Dermatol Treat. 2021 Feb;32(1):49–55.

57.	Lo Y, Tsai TF. Updates on the treatment of erythrodermic psoriasis. Psoriasis Targets Ther. 2021;59–73.

58.	Zhou T, Koussiouris J, Kim L, Vender R. Management of guttate psoriasis: a systematic review. J Cutan Med Surg. 2024;28(6):577–84.

59.	Rega F, Trovato F, Bortone G, Pellacani G, Richetta AG, Dattola A. Therapeutic potential of Spesolimab-Sbzo in the management of generalized pustular psoriasis flares in adults: evidence to date. Psoriasis Targets Ther. 2024;23–7.

60.	Puig L, Fujita H, Thaçi D, Zheng M, Hernandez Daly AC, Leonardi C, et al. Current treatments for generalized pustular psoriasis: a narrative summary of a systematic literature search. Dermatol Ther. 2024;14(9):2331–78.

61.	Chen B lin, Liu Q wei, Dong X wan, Bai Y ping. Biologics for generalized pustular psoriasis: a systematic review and single-arm meta-analysis. Front Immunol. 2024;15:1462158.

62.	Kearns DG, Chat VS, Zang PD, Han G, Wu JJ. Review of treatments for generalized pustular psoriasis. J Dermatol Treat. 2021 July 4;32(5):492–4.

63.	Sánchez‐García V, Hernández‐Quiles R, de‐Miguel‐Balsa E, Giménez‐Richarte Á, Ramos‐Rincón J, Belinchón‐Romero I. Exposure to biologic therapy before and during pregnancy in patients with psoriasis: systematic review and meta‐analysis. J Eur Acad Dermatol Venereol. 2023;37(10):1971–90.

64.	Owczarek W, Walecka I, Lesiak A, Czajkowski R, Reich A, Zerda I, et al. The use of biological drugs in psoriasis patients prior to pregnancy, during pregnancy and lactation: a review of current clinical guidelines. Adv Dermatol Allergol Dermatol Alergol. 2020;37(6):821–30.

65.	Jeong YD, Jo H, Cho H, Jang W, Park J, Lee S, et al. Biologics use for psoriasis during pregnancy and its related adverse outcomes in pregnant women and newborns: Findings from WHO pharmacovigilance study. Int Arch Allergy Immunol. 2025;186(6):579–93.

66.	Battista T, Gallo L, Martora F, Fattore D, Potestio L, Cacciapuoti S, et al. Biological therapy for psoriasis in cancer patients: an 8-year retrospective real-life study. J Clin Med. 2024;13(7):1940.

67.	Gupta A, Peyrin-Biroulet L, Ananthakrishnan AN. Risk of cancer recurrence in patients with immune-mediated diseases with use of immunosuppressive therapies: an updated systematic review and meta-analysis. Clin Gastroenterol Hepatol. 2024;22(3):499–512.

68.	Russell MD, Stovin C, Alveyn E, Adeyemi O, Chan CKD, Patel V, et al. JAK inhibitors and the risk of malignancy: a meta-analysis across disease indications. Ann Rheum Dis. 2023;82(8):1059–67.

69.	Alhalabi MM, Almokdad R. The incidence of hepatitis B reactivation in patients receiving ustekinumab: a systematic review and proportional meta-analysis. Eur J Gastroenterol Hepatol. 2025;37(1):1–9.

70.	Kuo MH, Ko PH, Wang ST, Tseng CW. Incidence of HBV Reactivation in Psoriasis Patients Undergoing Cytokine Inhibitor Therapy: A Single-Center Study and Systematic Review with a Meta-Analysis. Viruses. 2024;17(1):42.

71.	Li L, Jiang X, Fu L, Zhang L, Feng Y. Reactivation rates of hepatitis B or C or HIV in patients with psoriasis using biological therapies: a systematic review and meta-analysis. Clin Exp Med. 2023;23(3):701–15.

72.	Megna M, Lauletta G, Tommasino N, Salsano A, Battista T, Ruggiero A, et al. Management of psoriasis patients with serious infectious diseases. Adv Ther. 2024;41(6):2099–111.

73.	Zhu X, Pan X, Da M, Wang F, Dong Z. Risk for latent tuberculosis infection reactivation among patients with psoriasis on biologics treatment: a meta-analysis. J Infect. 2024;89(3).

74.	Mateu-Arrom L, Puig L. Infection Risk and Vaccination in the Management of Psoriasis: Considerations for Biologic Therapy. Psoriasis Targets Ther. 2025;127–44.

75.	Jafarzadeh A, Behrangi E, Khosravi M, Falakeh S, Amiri JK, Goodarzi A. Emerging treatments for dermatologic diseases in infants, children, and adolescents: a systematic review of clinical trials on biologics and small molecule inhibitors. Inflammopharmacology. 2025;1–56.

76.	Sticherling M, Nikkels AF, Hamza AM, Kwong P, Szepietowski JC, El Sayed M, et al. Secukinumab in pediatric patients with plaque psoriasis: pooled safety analysis from two phase 3 randomized clinical trials. Am J Clin Dermatol. 2023;24(5):821–35.

77.	Li C, Sunhe Y, Zhou H, Dong W. Efficacy and safety evaluations of adalimumab biosimilars in the treatment of psoriasis. J Dermatol Treat. 2023;34(1):2249145.

78.	Phan DB, Elyoussfi S, Stevenson M, Lunt M, Warren RB, Yiu ZZ. Biosimilars for the treatment of psoriasis: a systematic review of clinical trials and observational studies. JAMA Dermatol. 2023;159(7):763–71.

79.	Macaluso FS, Cummings JF, Atreya R, Choi J, Orlando A. A systematic review on infliximab biosimilar SB2: from pre-clinical data to real-world evidence. Expert Opin Biol Ther. 2022;22(2):203–23.

80.	Wang Q, Wang J, Sun X, Liu L, Zhang M, Yu Y, et al. Evidence-based dietary recommendations for patients with psoriasis: A systematic review. Clin Nutr. 2025;47:68–82.

81.	Wei K, Liao X, Yang T, He X, Yang D, Lai L, et al. Efficacy of probiotic supplementation in the treatment of psoriasis—A systematic review and meta‐analysis. J Cosmet Dermatol. 2024;23(7):2361–7.

82.	Lu L, Xu Y, Shi M, Liu A. Psychosocial interventions for psoriasis: a Bayesian network meta-analysis. J Dermatol Treat. 2025;36(1):2427321.

83.	Wei W, Zhang B, Liu T, Lu T. Effect of psychological intervention on quality of life among patients with psoriasis: a meta-analysis. Int J Behav Med. 2024;31(6):911–22.

## QUESTION 19. What are the efficacy and safety of biosimilars of biologic therapies in psoriasis?

Evidence supports that biosimilars of TNF inhibitors and ustekinumab are clinically equivalent to their reference products in psoriasis, with moderate certainty. The meta-analysis by Li 2023 [72] (8 RCTs; n = 2,589) found no differences between adalimumab biosimilars and Humira in PASI 50/75/90/100 at 16 weeks. No differences were observed in adverse events at weeks 16, 24, and 51, or in discontinuations due to adverse events. The authors concluded that biosimilars have efficacy and safety comparable to reference products in moderate-to-severe psoriasis, with the added benefit of lower cost. Another systematic review (Phan 2023) [73] similarly reports clinical equivalence between the reference biologic and adalimumab biosimilars (PASI efficacy, DLQI, and safety) both at treatment initiation and after switching, with moderate-to-high certainty for efficacy and low certainty for rare adverse events.

For infliximab, the systematic literature review by Macaluso 2022 [74] synthesized preclinical data, clinical trials, and real-world evidence on the biosimilar. Although pivotal RCTs were conducted in rheumatoid arthritis (demonstrating PK/efficacy and safety equivalence, and that switching from the reference product to SB2 does not increase immunogenicity), the review also includes real-world cohorts (IBD, rheumatology, and psoriasis) relevant to psoriasis through regulatory extrapolation. In plaque psoriasis, the review includes an observational study (Gisondi 2020) evaluating switching from CT-P13 to SB2, showing stable PASI over 6 months and discontinuation in ~10% (mainly due to loss of response or infusion reactions), with no new serious safety signals, supporting clinical interchangeability in this indication.

Efficacy and safety outcomes with an adalimumab biosimilar

| Outcome | **No. participants (study type)** | **Risk with originator** | **Risk with biosimilar** | **Absolute difference (95% CI)** | **Relative difference (RR or mean Δ, 95% CI)** | **Certainty of evidence (GRADE)** |
| --- | --- | --- | --- | --- | --- | --- |
| Biologic-naïve – PASI efficacy (52 wk) ~1,500 (6 RCTs) | ~1,500 (6 RCTs) | 85.6%–93.9% | 86.3%–92.8% | −4.5% to +3.1% (95% CI: −10.1% to +8.8%) | Mean Δ near 0 (±5%) | ⨁⨁⨁◯ Moderate |
| Switch – PASI efficacy (52 wk) ~450 (5 RCTs) | ~450 (5 RCTs) | 85.6%–93.9% | 84.2%–94.8% | −6.6% to +0.9% (95% CI: −12.9% to +4.8%) | Mean Δ near 0 (±5%) | ⨁⨁⨁◯ Moderate |
| Adverse events (AEs, 16 wk) ~1,800 (8 RCTs) | ~1,800 (8 RCTs) | 45%–55% (approx.) | 43%–52% | −3% to +6% (wide 95% CI includes benefit and harm) | RR 0.87–1.08 (95% CI 0.72–1.29) | ⨁⨁⨁◯ Moderate |
| Serious adverse events (SAEs, 16 wk) ~1,800 (8 RCTs) | ~1,800 (8 RCTs) | 2%–6% | 2%–5% | −2% to +1% (very wide 95% CI) | RR 0.40–1.35 | ⨁⨁◯◯ Low |

***Explanations***

1. Certainty downgraded for imprecision.

Efficacy and safety outcomes with an etanercept biosimilar

| Outcome | **No. participants (study type)** | **Risk with originator** | **Risk with biosimilar** | **Absolute difference (95% CI)** | **Relative difference (RR or mean Δ, 95% CI)** | **Certainty of evidence (GRADE)** |
| --- | --- | --- | --- | --- | --- | --- |
| PASI (% improvement from baseline) at 52 wk | 438 (1 RCT) | 82.9% (213/213; mean 82.9, SD 18.6) | 80.9% (225/225; mean 80.9, SD 25.1) | −2.0% (95% CI: −6.1 to +2.1) | Mean Δ −2.0% (95% CI: −6.1 to +2.1) | ⨁⨁⨁◯ Moderate |
| Adverse events (AEs, 16 wk) | 521 (1 RCT) | 199/260 (76.5%) | 191/261 (73.2%) | −3.3% (95% CI: −13% to +6%) | RR 0.96 (95% CI: 0.87–1.06) | ⨁⨁⨁◯ Moderate |
| Serious adverse events (SAEs, 16 wk) | 521 (1 RCT) | 10/260 (3.8%) | 7/261 (2.7%) | −1.1% (95% CI: −3% to +2%) | RR 0.70 (95% CI: 0.27–1.80) | ⨁⨁◯◯ Low |

***Explanations***

1. Certainty downgraded for imprecision.

Efficacy and safety outcomes with an infliximab biosimilar (CT-P13 vs Remicade)

| Outcome | **No. participants (study type)** | **Risk with originator** | **Risk with biosimilar** | **Absolute difference (95% CI)** | **Relative difference (RR or mean Δ, 95% CI)** | **Certainty of evidence (GRADE)** |
| --- | --- | --- | --- | --- | --- | --- |
| Biologic-naïve – PASI efficacy (52–78 wk) 481 (1 RCT) | 481 (1 RCT) | 241 patients (mean 47.5 years) | 240 patients (mean 48.2 years) | No meaningful difference | Mean Δ ≈ 0 (no meaningful CI) | ⨁⨁⨁◯ Moderate |
| Switch – PASI efficacy (2 years) 148 | 148 | HR 1.64 (95% CI: 0.69–3.89) | — | — | — | ⨁◯◯◯ Very low |
| Adverse events (AEs, 16 wk) 481 (1 RCT) | 481 (1 RCT) | Overall data, no differences | — | RR near 1 (95% CI includes 1) | RR ≈ 1.0 | ⨁⨁◯◯ Low |
| Serious adverse events (SAEs, 16 wk) 481 (1 RCT) | 481 (1 RCT) | Very few events | — | Very wide 95% CI | RR ≈ 1.0 | ⨁◯◯◯ Very low |

***Explanations***

1. Certainty downgraded for imprecision and a single study.

Efficacy and safety outcomes with an ustekinumab biosimilar (CT-P43 vs Stelara)

| **Outcome** | **No. participants (study type)** | **Risk with originator** | **Risk with biosimilar** | **Absolute difference (95% CI)** | **Relative difference (RR or mean Δ, 95% CI)** | **Certainty of evidence (GRADE)** |
| --- | --- | --- | --- | --- | --- | --- |
| Biologic-naïve – PASI efficacy (16 wk) 509 (1 RCT) | 509 (1 RCT) | 84.0% (248) | 86.1% (256) | +2.1% (95% CI: −0.7 to +4.9) | RR 1.12 (95% CI: 1.02–1.23) | ⨁⨁⨁◯ Moderate |
| Switch – PASI efficacy (32 wk) 248 (1 RCT) – PASI | 248 (1 RCT) – PASI | 92.9% (124) | 92.6% (124) | −0.3% (95% CI: −3.4 to +2.8) | Mean Δ ≈ 0 | ⨁⨁⨁⨁ High |
| Adverse events (AEs, 16 wk) 509 (1 RCT) | 509 (1 RCT) | 75/253 (29.6%) | 95/256 (37.1%) | +7.5% (95% CI: −2% to +17%) | RR 1.25 (95% CI: 0.98–1.60) | ⨁⨁⨁◯ Moderate |
| Serious adverse events (SAEs, 16 wk) 509 (1 RCT) | 509 (1 RCT) | 4/253 (1.6%) | 4/256 (1.6%) | 0% | RR 0.99 (95% CI: 0.25–3.91) | ⨁⨁◯◯ Low |

***Explanations***

1. Certainty downgraded for imprecision and a single study.

## QUESTION 20. What are the efficacy and safety of other nonpharmacologic interventions in psoriasis?

**Dieta**

A recent systematic review [75] reports that adherence to a Mediterranean diet, caloric restriction (including very-low-calorie ketogenic diets), increased fiber intake (including algae-derived fibers), and use of probiotics/prebiotics and omega-3 fatty acids are associated with improvements in disease activity (PASI), inflammatory markers, and metabolic comorbidities. In contrast, high salt and alcohol intake are associated with higher risk and worse psoriasis course. Evidence quality is heterogeneous (many observational studies and small trials), and findings for vitamin D and oral antioxidants are inconsistent or negative.

| **Outcome** | **Narrative effect** | **Certainty (GRADE)** | **Comments** |
| --- | --- | --- | --- |
| Mediterranean diet / caloric restriction / VLCKD | Associated with improvements in PASI, inflammation, and metabolic comorbidities in small and observational studies. | ⨁⨁◯◯ Low | Benefit appears clearer in overweight/obese patients; limited sample sizes. |
| Fiber / prebiotics / ω-3 | Signals of modest benefit on PASI and inflammatory markers; adjunctive effects. | ⨁⨁◯◯ Low | Favorable findings with heterogeneity in formulations and small trials. |
| Vitamin D / oral antioxidants | Most studies do not show a consistent effect on PASI. | ⨁◯◯◯ Very low | Inconsistent or negative evidence. |
| High salt / alcohol | Higher intake is associated with increased risk and worse psoriasis course in observational studies. | ⨁⨁◯◯ Low | Evidence based on observational associations. |

**Probiotics**

A meta-analysis [76] of 5 trials (n = 286) found that, compared with placebo, probiotics reduce severity (PASI) and improve quality of life (DLQI) in patients with psoriasis, with heterogeneity across studies and strain combinations.

| **Outcome** | **Narrative effect** | **Certainty (GRADE)** | **Comments** |
| --- | --- | --- | --- |
| PASI (severity) | Meta-analysis of 5 RCTs (n ≈ 286): meaningful reduction in PASI vs placebo (SMD −1.40, 95% CI −2.22 to −0.58). | ⊕⊕⊕◯ Moderate | Clinically relevant benefit with heterogeneity in studies and strain combinations. |
| DLQI (quality of life) | Meta-analysis: improvement in quality of life (SMD −0.92, 95% CI −1.63 to −0.21). | ⊕⊕⊕◯ Moderate | Consistent signal, limited by variability in strains used. |
| Adverse events | RCTs reported limited data; no clear differences vs placebo. | ⨁⨁◯◯ Low | Safety underreported; no meaningful harm signals. |

**Psychological interventions**

Psychological interventions in psoriasis (such as CBT, mindfulness, and psychoeducation) show clear benefit in reducing anxiety. The meta-analysis by Lu 2025 [77] (13 RCTs, n ≈ 1,048) found a reduction in anxiety (SMD −0.41; 95% CI −0.72 to −0.09), without consistent improvements in depression or quality of life. Key limitations included heterogeneity, small trials, and risk of bias due to lack of blinding.

The systematic review by Wei 2024 [78] found that more intensive, multicomponent programs integrated with dermatology care can improve depression and quality of life, although effects do not always reach statistical significance. Overall, evidence supports recommending psychological interventions as useful adjuncts to reduce anxiety, with potential benefits for depression and quality of life when delivered in structured, longer-duration formats.

| **Outcome** | **Narrative effect** | **Certainty (GRADE)** | **Comments** |
| --- | --- | --- | --- |
| Anxiety | Meta-analysis of 13 RCTs (n ≈ 1,048): reduction in anxiety (SMD −0.41, 95% CI −0.72 to −0.09). | ⊕⊕⊕◯ Moderate | Clinically relevant effect; heterogeneity and blinding limitations. |
| Depression | No difference (SMD −0.21; 95% CI −0.60 to 0.18). Benefit suggested when interventions are multicomponent and longer in duration. | ⊕⊕⊕◯ Moderate | No consistent effect; heterogeneous interventions. |
| Quality of life | No difference (SMD −0.01; 95% CI −0.38 to 0.35). Benefit suggested when interventions are multicomponent and longer in duration. | ⊕⊕⊕◯ Moderate | No consistent effect; heterogeneous interventions. |

**References**

1.	Yasmeen N, Sawyer LM, Malottki K, Levin LÅ, Didriksen Apol E, Jemec GB. Targeted therapies for patients with moderate-to-severe psoriasis: a systematic review and network meta-analysis of PASI response at 1 year. J Dermatol Treat. 2022 Jan 2;33(1):204–18.

2.	Belinchón Romero I, Dauden E, Ferrándiz Foraster C, González-Cantero Á, Carrascosa Carrillo JM. Therapeutic goals and treatment response evaluation in moderate to severe psoriasis: an experts opinion document. Ann Med. 2021 Jan;53(1):1727–36.

3.	Galluzzo M, Talamonti M, Manfreda V, Silvaggio D, Tartaglia C, Roberto D, et al. Impact of secukinumab on patient-reported outcomes in moderate to severe plaque psoriasis: a review of clinical studies. Expert Opin Biol Ther. 2021 Feb 1;21(2):191–200.

4.	Rusk AM, Fleischer AB. In psoriasis treatment, greater improvement in skin severity predicts greater improvement in nail severity. J Dermatol Treat. 2021 Nov 17;32(8):894–7.

5.	Belinchon Romero I, Mateu Puchades A, Ribera Pibernat M, Ruiz Genao DianaP, De La Cueva Dobao P, Carrascosa JM. Criteria used to define tumor necrosis factor-alpha inhibitors failure in patients with moderate-to-severe psoriasis: a systematic literature review. Ann Med. 2023 Dec 12;55(1):1335–45.

6.	Gisondi P, Di Mercurio M, Idolazzi L, Girolomoni G. Concept of Remission in Chronic Plaque Psoriasis. J Rheumatol Suppl. 2015 Nov 1;93(0):57–60.

7.	Puig L. PASI 90 response: the new standard in therapeutic efficacy for psoriasis. J Eur Acad Dermatol Venereol. 2015 Apr;29(4):645–8.

8.	Boswell ND, Singla S, Gordon KB. Sequencing of Targeted Therapy in Psoriasis: Does it Matter? Am J Clin Dermatol. 2024 Sept;25(5):795–810.

9.	Thaçi D, Piaserico S, Gaarn Du Jardin K, Warren RB. Long‐term stability of PASI <3 response to tildrakizumab: Pooled analyses from reSURFACE 1 and reSURFACE 2 phase 3 trials through 5 years. J Eur Acad Dermatol Venereol [Internet]. 2023 Apr [cited 2024 Oct 4];37(4). Available from: https://onlinelibrary.wiley.com/doi/10.1111/jdv.18783

10.	Imafuku S, Ohata C, Okubo Y, Tobita R, Saeki H, Mabuchi T, et al. Effectiveness of brodalumab in achieving treatment satisfaction for patients with plaque psoriasis: The ProLOGUE study. J Dermatol Sci. 2022 Mar;105(3):176–84.

11.	Reich K, Mrowietz U, Menter A, Griffiths CEM, Bagel J, Strober B, et al. Effect of baseline disease severity on achievement of treatment target with apremilast: results from a pooled analysis. J Eur Acad Dermatol Venereol. 2021 Dec;35(12):2409–14.

12.	Campanati A, Diotallevi F, Radi G, Molinelli E, Brisigotti V, Martina E, et al. Psoriatic patients treated with secukinumab reach high levels of minimal disease activity: results from the SUPREME study. Eur J Dermatol. 2021 Oct;31(5):630–7.

13.	Lebwohl MG, Soliman AM, Yang H, Wang J, Koenigsberg SH, Anderson A, et al. 25572 Impact of risankizumab on the duration of PASI90 achievement: A post hoc analysis of 4 phase III clinical trials. J Am Acad Dermatol. 2021 Sept;85(3):AB62.

14.	Puig L, Zhu B, Burge R, Shrom D, Dong Y, Shen W, et al. Early Treatment Targets for Predicting Long-term Dermatology Life Quality Index Response in Patients with Moderate-to-Severe Plaque Psoriasis: A Post-hoc Analysis from a Long-term Clinical Study. J Clin Aesthetic Dermatol. 2020 Oct;13(10):18–22.

15.	Puig L, Dossenbach M, Berggren L, Ljungberg A, Zachariae C. Absolute and Relative Psoriasis Area and Severity Indices (PASI) for Comparison of the Efficacy of Ixekizumab to Etanercept and Placebo in Patients with Moderate-to-severe Plaque Psoriasis: An Integrated Analysis of UNCOVER-2 and UNCOVER-3 Outcomes. Acta Derm Venereol. 2019;99(11):971–7.

16.	Merola JF, Amato DA, See K, Burge R, Mallinckrodt C, Ojeh CK, et al. Evaluation of sPGA × BSA as an Outcome Measure and Treatment Target for Clinical Practice. J Invest Dermatol. 2018 Sept;138(9):1955–61.

17.	Elewski BE, Puig L, Mordin M, Gilloteau I, Sherif B, Fox T, et al. Psoriasis patients with psoriasis Area and Severity Index (PASI) 90 response achieve greater health-related quality-of-life improvements than those with PASI 75–89 response: results from two phase 3 studies of secukinumab. J Dermatol Treat. 2017 Aug 18;28(6):492–9.

18.	Schmitt J, Rosumeck S, Thomaschewski G, Sporbeck B, Haufe E, Nast A. Efficacy and safety of systemic treatments for moderate-to-severe psoriasis: meta-analysis of randomized controlled trials. Br J Dermatol. 2014 Feb;170(2):274–303.

19.	Li Z, Pei B, Lei T, Yao M, Niu Y, Zhou P, et al. Clinical features and effects of cyclosporine in the treatment of psoriasis: a systemic review and meta-analysis. Arch Dermatol Res. 2024 Oct 26;316(10):705.

20.	Kogan N, Raimondo N, Gusis SE, Izcovich A, Abarca Duran JA, Barahona‐Torres L, et al. Latin American clinical practice guidelines on the systemic treatment of psoriasis SOLAPSO–Sociedad Latinoamericana de Psoriasis (Latin American Psoriasis Society). Int J Dermatol. 2019;58:4–28.

21.	van Huizen AM, Sikkel R, Caron AG, Menting SP, Spuls PI. Methotrexate dosing regimen for plaque-type psoriasis: an update of a systematic review. J Dermatol Treat. 2022;33(8):3104–18.

22.	Hsieh TS, Tsai TF. Combination therapy for psoriasis with methotrexate and other oral disease-modifying antirheumatic drugs: a systematic review. Dermatol Ther. 2023;13(4):891–909.

23.	Cheng Y, Liu GJ, Zhang M. Narrow‐band ultraviolet B phototherapy versus broad‐band ultraviolet B or psoralen‐ultraviolet A photochemotherapy for psoriasis. Cochrane Database Syst Rev. 2013;(10):CD009481.

24.	Sbidian E, Chaimani A, Garcia-Doval I, Doney L, Dressler C, Hua C, et al. Systemic pharmacological treatments for chronic plaque psoriasis: a network meta-analysis. Cochrane Database Syst Rev. 2022 May 23;5(5):CD011535.

25.	Mahmoud A, Ahmed AAS, Naeem A, Abuelazm M, Elshinawy M, Hassan AR, et al. Tyrosine Kinase-2 Inhibitor (Deucravacitinib) for Psoriasis: A Systematic Review and Network Meta-Analysis of Randomized Controlled Trials. Int J Dermatol Venereol. 2024 Dec;7(4):216.

26.	Armstrong AW, Soliman AM, Betts KA, Wang Y, Gao Y, Stakias V, et al. Long-term benefit–risk profiles of treatments for moderate-to-severe plaque psoriasis: a network meta-analysis. Dermatol Ther. 2022;12(1):167–84.

27.	Piragine E, Petri D, Martelli A, Janowska A, Dini V, Romanelli M, et al. Adherence and persistence to biological drugs for psoriasis: systematic review with meta-analysis. J Clin Med. 2022;11(6):1506.

28.	Gottlieb AB, Kalb RE, Blauvelt A, Heffernan MP, Sofen HL, Ferris LK, et al. The efficacy and safety of infliximab in patients with plaque psoriasis who had an inadequate response to etanercept: results of a prospective, multicenter, open-label study. J Am Acad Dermatol. 2012 Oct;67(4):642–50.

29.	Yamauchi PS, Bissonnette R, Teixeira HD, Valdecantos WC. Systematic review of efficacy of anti-tumor necrosis factor (TNF) therapy in patients with psoriasis previously treated with a different anti-TNF agent. J Am Acad Dermatol. 2016 Sept;75(3):612-618.e6.

30.	Piaserico S, Cazzaniga S, Chimenti S, Giannetti A, Maccarone M, Picardo M, et al. Efficacy of switching between tumor necrosis factor-alfa inhibitors in psoriasis: results from the Italian Psocare registry. J Am Acad Dermatol. 2014 Feb;70(2):257-262.e3.

31.	Boswell ND, Singla S, Gordon KB. Sequencing of Targeted Therapy in Psoriasis: Does it Matter? Am J Clin Dermatol. 2024 Sept;25(5):795–810.

32.	Warren RB, Blauvelt A, Poulin Y, Beeck S, Kelly M, Wu T, et al. Efficacy and safety of risankizumab vs. secukinumab in patients with moderate-to-severe plaque psoriasis (IMMerge): results from a phase III, randomized, open-label, efficacy-assessor-blinded clinical trial. Br J Dermatol. 2021 Jan;184(1):50–9.

33.	Blauvelt A, Papp KA, Griffiths CEM, Randazzo B, Wasfi Y, Shen YK, et al. Efficacy and safety of guselkumab, an anti-interleukin-23 monoclonal antibody, compared with adalimumab for the continuous treatment of patients with moderate to severe psoriasis: Results from the phase III, double-blinded, placebo- and active comparator-controlled VOYAGE 1 trial. J Am Acad Dermatol. 2017 Mar;76(3):405–17.

34.	Reich K, Armstrong AW, Foley P, Song M, Wasfi Y, Randazzo B, et al. Efficacy and safety of guselkumab, an anti-interleukin-23 monoclonal antibody, compared with adalimumab for the treatment of patients with moderate to severe psoriasis with randomized withdrawal and retreatment: Results from the phase III, double-blind, placebo- and active comparator-controlled VOYAGE 2 trial. J Am Acad Dermatol. 2017 Mar;76(3):418–31.

35.	Reich K, Gooderham M, Thaçi D, Crowley JJ, Ryan C, Krueger JG, et al. Risankizumab compared with adalimumab in patients with moderate-to-severe plaque psoriasis (IMMvent): a randomised, double-blind, active-comparator-controlled phase 3 trial. Lancet Lond Engl. 2019 Aug 17;394(10198):576–86.

36.	Warren RB, Blauvelt A, Bagel J, Papp KA, Yamauchi P, Armstrong A, et al. Bimekizumab versus Adalimumab in Plaque Psoriasis. N Engl J Med. 2021 July 8;385(2):130–41.

37.	Warren RB, Barker JNWB, Finlay AY, Burden AD, Kirby B, Armendariz Y, et al. Secukinumab for patients failing previous tumour necrosis factor-α inhibitor therapy: results of a randomized open-label study (SIGNATURE). Br J Dermatol. 2020 July;183(1):60–70.

38.	Strober B, Paul C, Blauvelt A, Thaçi D, Puig L, Lebwohl M, et al. Bimekizumab efficacy and safety in patients with moderate to severe plaque psoriasis: Two-year interim results from the open-label extension of the randomized BE RADIANT phase 3b trial. J Am Acad Dermatol. 2023 Sept;89(3):486–95.

39.	Loft N, Halling AS, Egeberg A, Skov L. Efficacy of a second interleukin 17 inhibitor in patients with psoriasis: A systematic review and meta-analysis. J Am Acad Dermatol. 2021 Jan;84(1):130–8.

40.	Loft N, Egeberg A, Isufi D, Rasmussen MK, Bryld LE, Dam TN, et al. Response to Interleukin-17A Inhibitors According to Prior Biologic Exposures: A Danish Nationwide Study. Acta Derm Venereol. 2023 Nov 21;103:adv12616.

41.	Papp K, Blauvelt A, Sullivan J, Tada Y, Polzer P, Mallbris L, et al. Ixekizumab—An Effective and Safe Treatment for Moderate-to-Severe Plaque Psoriasis in Patients Previously Treated With Other IL-17 Inhibitors: Results From IXORA-P. J Psoriasis Psoriatic Arthritis®. 2019 Oct 1;4(4):180–5.

42.	Reddy R, Pannu S, Fiumara K, Kahn J, Rosmarin D. Efficacy of in-class interleukin-23 inhibitor switching: risankizumab following guselkumab failure in moderate-to-severe psoriasis treatment. Br J Dermatol. 2021 Mar;184(3):559–61.

43.	Gasslitter I, Kirsten N, Augustin M, Torz K, Mrowietz U, Eyerich K, et al. Successful intra-class switching among IL-17 antagonists: a multicentre, multinational, retrospective study. Arch Dermatol Res. 2019 July;311(5):421–4.

44.	Daniele SG, Eldirany SA, Damiani G, Ho M, Bunick CG. Structural Basis for p19 Targeting by Anti-IL-23 Biologics: Correlations with Short- and Long-Term Efficacy in Psoriasis. JID Innov Skin Sci Mol Popul Health. 2024 Mar;4(2):100261.

45.	Camela E, Ocampo‐Garza SS, Cinelli E, Villani A, Fabbrocini G, Megna M. Therapeutic update of biologics and small molecules for scalp psoriasis: a systematic review. Dermatol Ther. 2021;34(2):e14857.

46.	Mosca M, Hong J, Hadeler E, Brownstone N, Bhutani T, Liao W. Scalp psoriasis: a literature review of effective therapies and updated recommendations for practical management. Dermatol Ther. 2021;11(3):769–97.

47.	Tsiogkas SG, Karamitrou EK, Grammatikopoulou MG, Zafiriou E, Bogdanos DP. Efficacy of tyrosine-kinase-2 and phosphodiesterase-4 inhibitors for scalp psoriasis: a systematic review and meta-analysis. Curr Med Res Opin. 2024;40(2):155–63.

48.	Duffin KC, Griffiths CE, Hoffmann M, Blauvelt A, Balagula E, Napoli A, et al. Deucravacitinib, an Oral Selective Tyrosine Kinase 2 (TYK2) Inhibitor, in Patients with Moderate to Severe Scalp Psoriasis: Efficacy and Safety Results of a Phase 3b/4, Multicenter, Randomized, Double-blinded, Placebo-controlled Trial (PSORIATYK SCALP). SKIN J Cutan Med. 2024;8(6):s420–s420.

49.	Khan M, Wallace CE, Ahmed F, Rahman SM, Memon N, Haque A. Assessing Comparative Efficacy of Biologics for the Treatment of Psoriasis With Nail Involvement: A Systematic Review. J Psoriasis Psoriatic Arthritis®. 2024;9(2):61–8.

50.	Hwang JK, Ricardo JW, Lipner SR. Efficacy and safety of nail psoriasis targeted therapies: a systematic review. Am J Clin Dermatol. 2023;24(5):695–720.

51.	Hong JJ, Mosca ML, Hadeler EK, Brownstone ND, Bhutani T, Liao WJ. Genital and inverse/intertriginous psoriasis: an updated review of therapies and recommendations for practical management. Dermatol Ther. 2021;11(3):833–44.

52.	Beck KM, Yang EJ, Sanchez IM, Liao W. Treatment of Genital Psoriasis: A Systematic Review. Dermatol Ther. 2018 Dec;8(4):509–25.

53.	Tsiogkas SG, Grammatikopoulou MG, Kontouli KM, Minopoulou I, Goulis DD, Zafiriou E, et al. Efficacy of biologic agents for palmoplantar psoriasis: a systematic review and network meta-analysis. Expert Rev Clin Immunol. 2023;19(12):1485–98.

54.	Huang IH, Wu PC, Chiu HY, Huang YH. Small-molecule inhibitors and biologics for palmoplantar psoriasis and palmoplantar pustulosis: a systematic review and network meta-analysis. Am J Clin Dermatol. 2024;25(3):347–58.

55.	Xia R, Diao Z, Chen D, Wang Y, Zhou C, Gao Y, et al. Efficacy of anti-interleukin-17A biological agents for palmoplantar psoriasis and palmoplantar pustulosis: A network meta-analysis. Int Immunopharmacol. 2024;130:111716.

56.	Reynolds KA, Pithadia DJ, Lee EB, Liao W, Wu JJ. A systematic review of treatment strategies for erythrodermic psoriasis. J Dermatol Treat. 2021 Feb;32(1):49–55.

57.	Lo Y, Tsai TF. Updates on the treatment of erythrodermic psoriasis. Psoriasis Targets Ther. 2021;59–73.

58.	Zhou T, Koussiouris J, Kim L, Vender R. Management of guttate psoriasis: a systematic review. J Cutan Med Surg. 2024;28(6):577–84.

59.	Rega F, Trovato F, Bortone G, Pellacani G, Richetta AG, Dattola A. Therapeutic potential of Spesolimab-Sbzo in the management of generalized pustular psoriasis flares in adults: evidence to date. Psoriasis Targets Ther. 2024;23–7.

60.	Puig L, Fujita H, Thaçi D, Zheng M, Hernandez Daly AC, Leonardi C, et al. Current treatments for generalized pustular psoriasis: a narrative summary of a systematic literature search. Dermatol Ther. 2024;14(9):2331–78.

61.	Chen B lin, Liu Q wei, Dong X wan, Bai Y ping. Biologics for generalized pustular psoriasis: a systematic review and single-arm meta-analysis. Front Immunol. 2024;15:1462158.

62.	Kearns DG, Chat VS, Zang PD, Han G, Wu JJ. Review of treatments for generalized pustular psoriasis. J Dermatol Treat. 2021 July 4;32(5):492–4.

63.	Sánchez‐García V, Hernández‐Quiles R, de‐Miguel‐Balsa E, Giménez‐Richarte Á, Ramos‐Rincón J, Belinchón‐Romero I. Exposure to biologic therapy before and during pregnancy in patients with psoriasis: systematic review and meta‐analysis. J Eur Acad Dermatol Venereol. 2023;37(10):1971–90.

64.	Owczarek W, Walecka I, Lesiak A, Czajkowski R, Reich A, Zerda I, et al. The use of biological drugs in psoriasis patients prior to pregnancy, during pregnancy and lactation: a review of current clinical guidelines. Adv Dermatol Allergol Dermatol Alergol. 2020;37(6):821–30.

65.	Jeong YD, Jo H, Cho H, Jang W, Park J, Lee S, et al. Biologics use for psoriasis during pregnancy and its related adverse outcomes in pregnant women and newborns: Findings from WHO pharmacovigilance study. Int Arch Allergy Immunol. 2025;186(6):579–93.

66.	Battista T, Gallo L, Martora F, Fattore D, Potestio L, Cacciapuoti S, et al. Biological therapy for psoriasis in cancer patients: an 8-year retrospective real-life study. J Clin Med. 2024;13(7):1940.

67.	Gupta A, Peyrin-Biroulet L, Ananthakrishnan AN. Risk of cancer recurrence in patients with immune-mediated diseases with use of immunosuppressive therapies: an updated systematic review and meta-analysis. Clin Gastroenterol Hepatol. 2024;22(3):499–512.

68.	Russell MD, Stovin C, Alveyn E, Adeyemi O, Chan CKD, Patel V, et al. JAK inhibitors and the risk of malignancy: a meta-analysis across disease indications. Ann Rheum Dis. 2023;82(8):1059–67.

69.	Alhalabi MM, Almokdad R. The incidence of hepatitis B reactivation in patients receiving ustekinumab: a systematic review and proportional meta-analysis. Eur J Gastroenterol Hepatol. 2025;37(1):1–9.

70.	Kuo MH, Ko PH, Wang ST, Tseng CW. Incidence of HBV Reactivation in Psoriasis Patients Undergoing Cytokine Inhibitor Therapy: A Single-Center Study and Systematic Review with a Meta-Analysis. Viruses. 2024;17(1):42.

71.	Li L, Jiang X, Fu L, Zhang L, Feng Y. Reactivation rates of hepatitis B or C or HIV in patients with psoriasis using biological therapies: a systematic review and meta-analysis. Clin Exp Med. 2023;23(3):701–15.

72.	Megna M, Lauletta G, Tommasino N, Salsano A, Battista T, Ruggiero A, et al. Management of psoriasis patients with serious infectious diseases. Adv Ther. 2024;41(6):2099–111.

73.	Zhu X, Pan X, Da M, Wang F, Dong Z. Risk for latent tuberculosis infection reactivation among patients with psoriasis on biologics treatment: a meta-analysis. J Infect. 2024;89(3).

74.	Mateu-Arrom L, Puig L. Infection Risk and Vaccination in the Management of Psoriasis: Considerations for Biologic Therapy. Psoriasis Targets Ther. 2025;127–44.

75.	Jafarzadeh A, Behrangi E, Khosravi M, Falakeh S, Amiri JK, Goodarzi A. Emerging treatments for dermatologic diseases in infants, children, and adolescents: a systematic review of clinical trials on biologics and small molecule inhibitors. Inflammopharmacology. 2025;1–56.

76.	Sticherling M, Nikkels AF, Hamza AM, Kwong P, Szepietowski JC, El Sayed M, et al. Secukinumab in pediatric patients with plaque psoriasis: pooled safety analysis from two phase 3 randomized clinical trials. Am J Clin Dermatol. 2023;24(5):821–35.

77.	Li C, Sunhe Y, Zhou H, Dong W. Efficacy and safety evaluations of adalimumab biosimilars in the treatment of psoriasis. J Dermatol Treat. 2023;34(1):2249145.

78.	Phan DB, Elyoussfi S, Stevenson M, Lunt M, Warren RB, Yiu ZZ. Biosimilars for the treatment of psoriasis: a systematic review of clinical trials and observational studies. JAMA Dermatol. 2023;159(7):763–71.

79.	Macaluso FS, Cummings JF, Atreya R, Choi J, Orlando A. A systematic review on infliximab biosimilar SB2: from pre-clinical data to real-world evidence. Expert Opin Biol Ther. 2022;22(2):203–23.

80.	Wang Q, Wang J, Sun X, Liu L, Zhang M, Yu Y, et al. Evidence-based dietary recommendations for patients with psoriasis: A systematic review. Clin Nutr. 2025;47:68–82.

81.	Wei K, Liao X, Yang T, He X, Yang D, Lai L, et al. Efficacy of probiotic supplementation in the treatment of psoriasis—A systematic review and meta‐analysis. J Cosmet Dermatol. 2024;23(7):2361–7.

82.	Lu L, Xu Y, Shi M, Liu A. Psychosocial interventions for psoriasis: a Bayesian network meta-analysis. J Dermatol Treat. 2025;36(1):2427321.

83.	Wei W, Zhang B, Liu T, Lu T. Effect of psychological intervention on quality of life among patients with psoriasis: a meta-analysis. Int J Behav Med. 2024;31(6):911–22.
