# Supplementary material for: Clinical practice guideline for psoriasis management in Latin America
Source: An Bras Dermatol. 2026 Aug 29;101(5):501449. doi: 10.1016/j.abd.2026.501449 (PMC13545376; doi:10.1016/j.abd.2026.501449)
Supplement: Supplementary file 3 [file mmc3.docx]

**CLINICAL PRACTICE GUIDELINE FOR PSORIASIS MANAGEMENT IN LATIN AMERICA**

**Latin American Psoriasis Society – SOLAPSO**

**Supplementary material 3. Evidence-to-decision framework**

## **Intervention: Methotrexate**

| **Criterion** | **Judgment** |
| --- | --- |
| **Priority of the problem: Magnitude** | Yes. A core option for patients with moderate-to-severe psoriasis who do not respond to topical therapies. |
| **Expected desirable effects: Effectiveness** | Moderate. Has demonstrated sustained efficacy in disease control and improved quality of life. |
| **Anticipated undesirable effects: Safety** | Moderate/small. Can cause toxicities that require periodic monitoring. Not for use in pregnancy. Requires folic acid supplementation. |
| **Balance of benefits and harms** | Probably favors the intervention. In selected patients, benefits outweigh harms when dose, route of administration, and clinical follow-up are maintained. |
| **Resources required for implementation** | Moderate cost savings. More accessible than biologic therapies and effective in many settings. |
| **Acceptability by patients and stakeholders** | Probably yes. Widely known and used. Requires well-established protocols. |
| **Feasibility of implementation** | Yes. Available in most health systems. Use is feasible with medical follow-up. Not all formulations may be available in every country. |

## **Intervention: Cyclosporine**

| **Criterion** | **Judgment** |
| --- | --- |
| **Priority of the problem: Magnitude** | Yes. Useful, especially in patients with acute presentations. |
| **Expected desirable effects: Effectiveness** | Moderate/large. Effective in acute presentations. Sustained results in the short to medium term. |
| **Anticipated undesirable effects: Safety** | Large. Prolonged use should not exceed 1 year because of potential nephrotoxicity and infections; requires frequent monitoring. |
| **Balance of benefits and harms** | Probably favors the intervention/favors the intervention. Provides an acceptable safety profile as a treatment option for <1 year. |
| **Resources required for implementation** | Moderate costs. Less expensive than biologics. Targeted use reduces economic impact. Requires follow-up, which implies greater resource use. |
| **Acceptability by patients and stakeholders** | Probably yes. A recognized and valued option in specific clinical settings, although it requires adverse event monitoring. |
| **Feasibility of implementation** | Yes. Available in participating countries’ health systems. |

## **Intervention: Acitretin**

| **Criterion** | **Judgment** |
| --- | --- |
| **Priority of the problem: Magnitude** | Yes. A core option in patients with special forms of psoriasis or when alternative therapeutic options are needed. |
| **Expected desirable effects: Effectiveness** | Moderate. Has demonstrated efficacy in disease control and improved quality of life. |
| **Anticipated undesirable effects: Safety** | Moderate. Can cause toxicities that require periodic monitoring. Not for use in pregnancy. |
| **Balance of benefits and harms** | Probably favors the intervention. Has an acceptable safety profile and clear clinical benefits. Given its teratogenic potential, adequate contraception in women of reproductive potential is required. |
| **Resources required for implementation** | Moderate costs. More accessible than biologic therapies and effective in some settings. |
| **Acceptability by patients and stakeholders** | Probably yes. Widely known and used. Not appropriate for women seeking pregnancy, which limits use in this subgroup. |
| **Feasibility of implementation** | Probably yes/yes. Available in most health systems in the region, although barriers exist in some countries. Use is feasible with medical follow-up. |

## **Intervention: Phototherapy**

| **Criterion** | **Judgment** |
| --- | --- |
| **Priority of the problem: Magnitude** | Yes. Moderate-to-severe psoriasis requires effective therapeutic alternatives, and phototherapy is a valuable option, especially in patients for whom other topical or systemic therapies are not feasible or are contraindicated. |
| **Expected desirable effects: Effectiveness** | Moderate. Effective for extensive or resistant forms of psoriasis, achieving good clinical response and improved quality of life in selected patients. |
| **Anticipated undesirable effects: Safety** | Small. Can cause erythema, mild burns, or photoaging effects with prolonged use. Generally safe when administered under medical supervision. |
| **Balance of benefits and harms** | Probably favors the intervention. Phototherapy offers a favorable safety profile and acceptable efficacy when appropriate dose and frequency protocols are followed. |
| **Resources required for implementation** | Moderate costs. Requires specialized equipment, adequate facilities, and multiple sessions, which can limit access in some settings. |
| **Acceptability by patients and stakeholders** | Probably yes. Well regarded by those who can access it, although treatment frequency can be a barrier. |
| **Feasibility of implementation** | Probably yes. Not available in all centers across the region. Specialized services offer it. |

## **Intervention: Biologic agents anti–IL-12/23 (ustekinumab), anti–IL-17 (secukinumab, ixekizumab, brodalumab, and bimekizumab), or anti–IL-23 (guselkumab, risankizumab, tildrakizumab).**

| **Criterion** | **Judgment** |
| --- | --- |
| **Priority of the problem: Magnitude** | Yes. Moderate-to-severe psoriasis in adults carries a high disease burden, which supports effective and safe interventions. |
| **Expected desirable effects: Effectiveness** | Large. Have demonstrated high, sustained efficacy in disease control and improved quality of life. |
| **Anticipated undesirable effects: Safety** | Varies. Generally well tolerated, although there is a risk of infections and other adverse events that require monitoring, as well as TB reactivation in countries with high prevalence. |
| **Balance of benefits and harms** | Favors the intervention. High efficacy and an acceptable safety profile support use in indicated cases. |
| **Resources required for implementation** | High/moderate costs. Represent an important economic burden, although cost-effective in severe disease. Reduces costs by preventing comorbidities such as arthropathy. |
| **Acceptability by patients and stakeholders** | Yes. Well accepted for efficacy, convenience, and safety profile, although administration logistics are required. |
| **Feasibility of implementation** | Probably yes. Available in several countries, although access can depend on the health system and coverage policies. |

## **Intervention: Anti-TNF (adalimumab, certolizumab)**

| **Criterion** | **Judgment** |
| --- | --- |
| **Priority of the problem: Magnitude** | Yes. Moderate-to-severe psoriasis in adults carries a high disease burden, which supports effective and safe interventions. |
| **Expected desirable effects: Effectiveness** | Moderate. Have demonstrated high, sustained efficacy in disease control and improved quality of life. |
| **Anticipated undesirable effects: Safety** | Moderate. Generally well tolerated, although there is a risk of infections and other adverse events that require monitoring. Use is limited in cancer. |
| **Balance of benefits and harms** | Probably favors the intervention/favors the intervention. High efficacy and an acceptable safety profile support use in indicated cases. |
| **Resources required for implementation** | Varies. Represent an important economic burden, although cost-effective in severe disease. |
| **Acceptability by patients and stakeholders** | Yes. Well accepted for efficacy, convenience, and safety profile, although administration logistics are required. |
| **Feasibility of implementation** | Probably yes/yes. Available in several countries, although access can depend on the health system and coverage policies. |

## **Intervention: Deucravacitinib**

| **Criterion** | **Judgment** |
| --- | --- |
| **Priority of the problem: Magnitude** | Yes. Moderate-to-severe psoriasis in adults carries a high disease burden, which supports effective and safe interventions. |
| **Expected desirable effects: Effectiveness** | Moderate. Has demonstrated sustained efficacy in disease control and improved quality of life. |
| **Anticipated undesirable effects: Safety** | Moderate. Generally well tolerated, although there is a risk of infections and other adverse events that require monitoring. |
| **Balance of benefits and harms** | Probably favors the intervention/favors the intervention. High efficacy and an acceptable safety profile support use in indicated cases. |
| **Resources required for implementation** | Varies. Represents an important economic burden, although cost-effective in severe disease. |
| **Acceptability by patients and stakeholders** | Probably yes. Well accepted for efficacy, convenience, and safety profile. |
| **Feasibility of implementation** | Probably yes/yes. Available in several countries, although access can depend on the health system and coverage policies. |

## **Intervention: Apremilast**

| **Criterion** | **Judgment** |
| --- | --- |
| **Priority of the problem: Magnitude** | Yes. Moderate-to-severe psoriasis in adults carries a high disease burden, which supports effective and safe interventions. |
| **Expected desirable effects: Effectiveness** | Moderate. Has demonstrated efficacy in selected cases. |
| **Anticipated undesirable effects: Safety** | Moderate. Generally well tolerated, although there is a risk of infections and other adverse events that require monitoring. |
| **Balance of benefits and harms** | Probably favors the intervention/favors the intervention. High efficacy and an acceptable safety profile support use in indicated cases. |
| **Resources required for implementation** | Varies. Represents an important economic burden, although cost-effective in severe disease. |
| **Acceptability by patients and stakeholders** | Probably yes. Accepted for efficacy, convenience, and safety profile. Newer therapies are increasingly preferred. |
| **Feasibility of implementation** | Probably yes/yes. Available in several countries, although access can depend on the health system and coverage policies. |

# Intervention: Biosimilars (for TNF-α and IL-12/23 inhibitors)

| **Criterio** | **Judgment** |
| --- | --- |
| **Priority of the problem: Magnitude** | Yes. Moderate-to-severe psoriasis in adults carries a high disease burden, which supports effective and safe interventions. |
| **Expected desirable effects: Effectiveness** | Large. Have demonstrated high, sustained efficacy in disease control and improved quality of life. |
| **Anticipated undesirable effects: Safety** | Varies. Generally well tolerated, with a safety profile similar to the reference biologic. |
| **Balance of benefits and harms** | Favors the intervention. Efficacy and an acceptable safety profile support use in indicated cases. |
| **Resources required for implementation** | Moderate costs. Represent an important economic burden, although at lower costs than originator biologics, which can expand access in resource-limited health systems. |
| **Acceptability by patients and stakeholders** | Probably yes. Can be accepted if efficacy and safety are comparable to the reference biologic. |
| **Feasibility of implementation** | Probably yes. Available in several countries, although access can depend on the health system and coverage policies. |

# Intervention: Topical treatment in pregnant patients

| **Criterion** | **Judgment** |
| --- | --- |
| **Priority of the problem: Magnitude** | Yes. Psoriasis carries a high disease burden, which supports effective and safe interventions. Topical therapies can be first-line in psoriasis management or in cases where other therapies cannot be used. |
| **Expected desirable effects: Effectiveness** | Large. Have demonstrated sustained efficacy in disease control and improved quality of life, especially as part of combination therapy. There is extensive experience with use. |
| **Anticipated undesirable effects: Safety** | Probably favors the intervention. Generally well tolerated. Require follow-up, although adverse events are usually local and manageable. Topical corticosteroids require closer follow-up to prevent overuse and risk of adverse events. |
| **Balance of benefits and harms** | Favors the intervention. High efficacy and an acceptable safety profile support use. |
| **Resources required for implementation** | Low cost. Lower cost than biologics. May involve out-of-pocket spending. |
| **Acceptability by patients and stakeholders** | Yes. Well accepted for efficacy, convenience, and safety profile, although daily use can affect adherence. |
| **Feasibility of implementation** | Probably yes. Available in several countries, although access can depend on the health system and coverage policies. Facilitates use at all levels of care. |

# Intervention: Certolizumab pegol in pregnant patients

| **Criterion** | **Judgment** |
| --- | --- |
| **Priority of the problem: Magnitude** | Yes. Psoriasis carries a high disease burden, which supports effective and safe interventions. It is the first option in pregnant patients who require biologic therapy. |
| **Expected desirable effects: Effectiveness** | Moderate. Has been useful in pregnant patients because it minimizes placental transfer. |
| **Anticipated undesirable effects: Safety** | Probably favors the intervention. Generally well tolerated. |
| **Balance of benefits and harms** | Favors the intervention. Available evidence indicates that certolizumab pegol has a favorable safety profile during pregnancy and lactation, without a meaningful increase in malformations or neonatal complications. |
| **Resources required for implementation** | Moderate/high cost. High health system spending, but use minimizes development of new comorbidities. |
| **Acceptability by patients and stakeholders** | Yes. Well accepted for efficacy, convenience, and safety profile, although administration logistics are required. |
| **Feasibility of implementation** | Probably yes. Available in several countries, although access can depend on the health system and coverage policies. |

## **Intervention: Biologic agents (etanercept, adalimumab, ixekizumab, secukinumab, ustekinumab).**

| **Criterion** | **Judgment** |
| --- | --- |
| **Priority of the problem: Magnitude** | Yes. Psoriasis in children carries a high disease burden, which supports effective and safe interventions. It has greater physical, emotional, and social impact at this stage of life. |
| **Expected desirable effects: Effectiveness** | Large. Have demonstrated high, sustained efficacy in disease control and improved quality of life. Studies in this population are limited. |
| **Anticipated undesirable effects: Safety** | Varies. Generally well tolerated, although there is a risk of infections and other adverse events that require monitoring, as well as tuberculosis reactivation in countries with high prevalence. |
| **Balance of benefits and harms** | Favors the intervention. High efficacy and an acceptable safety profile support use in indicated cases. |
| **Resources required for implementation** | High/moderate costs. Represent an important economic burden, although cost-effective in severe disease. Reduces costs by preventing comorbidities. |
| **Acceptability by patients and stakeholders** | Yes. Well accepted for efficacy, convenience, and safety profile, although administration logistics are required. |
| **Feasibility of implementation** | Probably yes. Available in several countries, although access can depend on the health system and coverage policies. Biologic options in children are reduced by age-group restrictions. In some countries, they are used off-label. |
