# Supplementary material for: Clinical practice guideline for psoriasis management in Latin America
Source: An Bras Dermatol. 2026 Aug 29;101(5):501449. doi: 10.1016/j.abd.2026.501449 (PMC13545376; doi:10.1016/j.abd.2026.501449)
Supplement: Supplementary file 4 [file mmc4.docx]

**CLINICAL PRACTICE GUIDELINE FOR PSORIASIS MANAGEMENT IN LATIN AMERICA**

**Latin American Psoriasis Society – SOLAPSO**

**Supplementary material 4. Monitoring indicators**

The indicators presented below are proposed as a guiding framework to monitor implementation of recommendations from the Clinical Practice Guideline for Psoriasis Management in Latin America. These indicators are suggested and should be adapted by each country and health system according to its epidemiologic, regulatory, organizational context and resource availability.

The star symbol next to the indicator name represents tracer indicators suggested for priority monitoring of guideline implementation. A tracer indicator is a priority measure selected for its clinical impact, representativeness, and feasibility, allowing concise and ongoing monitoring of care quality and the degree of clinical practice guideline implementation. These indicators summarize performance of relevant care processes and facilitate identification of gaps, follow-up of clinically meaningful outcomes, and decision-making at the institutional, regional, or national level.

| **Indicator name** | **Comprehensive patient assessment** |
| --- | --- |
| **Dimension** | Process |
| **Operational definition** | Proportion of patients with psoriasis with documented comprehensive clinical assessment. |
| **Numerator** | Patients with clinical assessment, comorbidities, and psychosocial aspects recorded. |
| **Denominator** | Total patients with psoriasis seen. |
| **Source** | Medical record. |
| **Frequency** | Semiannual. |
| **Suggested target** | ≥80%. |

| **Indicator name** | **Quality-of-life measurement** |
| --- | --- |
| **Dimension** | Process |
| **Operational definition** | Proportion of patients with documented quality-of-life measurement. |
| **Numerator** | Patients with DLQI or another validated instrument recorded. |
| **Denominator** | Patients with psoriasis in follow-up. |
| **Source** | Medical record. |
| **Frequency** | Semiannual. |
| **Suggested target** | ≥70%. |

| **Indicator name** | **Objective severity measurement** |
| --- | --- |
| **Dimension** | Process |
| **Operational definition** | Proportion of patients with objective severity assessment according to clinical phenotype. |
| **Numerator** | Patients with PASI, PGA, NAPSI, or GPPASI documented. |
| **Denominator** | Patients with psoriasis on active treatment. |
| **Source** | Medical record. |
| **Frequency** | At each visit. |
| **Suggested target** | ≥90%. |

| **Indicator name** | **Appropriate use of first-line systemic therapy** |
| --- | --- |
| **Dimension** | Process |
| **Operational definition** | Proportion of patients treated with conventional systemic therapies or phototherapy as first-line therapy. |
| **Numerator** | Patients treated according to first-line indications. |
| **Denominator** | Patients with an indication for systemic treatment. |
| **Source** | Medical record / Pharmacy. |
| **Frequency** | Annual. |
| **Suggested target** | ≥75%. |

| **Indicator name** | **Appropriate use of biologic therapies** |
| --- | --- |
| **Dimension** | Process |
| **Operational definition** | Proportion of patients who initiate biologic therapy with documented indication according to the guideline. |
| **Numerator** | Patients with the correct indication. |
| **Denominator** | Patients initiating biologic therapy. |
| **Source** | Medical record. |
| **Frequency** | Annual. |
| **Suggested target** | ≥90%. |

| **Indicator name** | **Timely therapeutic adjustment** |
| --- | --- |
| **Dimension** | Process |
| **Operational definition** | Proportion of patients without adequate response who undergo therapeutic adjustment according to the guideline. |
| **Numerator** | Patients with documented pharmacologic adjustment. |
| **Denominator** | Patients with therapeutic failure. |
| **Source** | Medical record. |
| **Frequency** | Quarterly. |
| **Suggested target** | ≥80%. |

| **Indicator name** | **Infectious screening before immunosuppression** |
| --- | --- |
| **Dimension** | Process |
| **Operational definition** | Proportion of patients with complete infectious screening before systemic therapy. |
| **Numerator** | Patients who are candidates for immunosuppression with TB, HBV, HCV, and HIV screening. |
| **Denominator** | Patients who are candidates for immunosuppression. |
| **Source** | Medical record. |
| **Frequency** | Pretreatment. |
| **Suggested target** | ≥90%. |

| **Indicator name** | **Documented patient education** |
| --- | --- |
| **Dimension** | Process |
| **Operational definition** | Proportion of patients with documented education on disease and self-care. |
| **Numerator** | Patients with documented education. |
| **Denominator** | Patients in follow-up. |
| **Source** | Medical record. |
| **Frequency** | Annual. |
| **Suggested target** | ≥80%. |

| **Indicator name** | **Achievement of therapeutic target** |
| --- | --- |
| **Dimension** | Outcome |
| **Operational definition** | Proportion of patients who achieve the therapeutic target defined according to psoriasis type. |
| **Numerator** | Patients with PASI <3, PASI 90, PGA 0/1, DLQI <5, or equivalent targets. |
| **Denominator** | Patients assessed. |
| **Source** | Medical record. |
| **Frequency** | 12–16 weeks. |
| **Suggested target** | ≥60–70%. |

| **Indicator name** | **Quality-of-life improvement** |
| --- | --- |
| **Dimension** | Outcome |
| **Operational definition** | Proportion of patients with clinically meaningful improvement in DLQI. |
| **Numerator** | Patients with DLQI <5 or a meaningful reduction. |
| **Denominator** | Patients with baseline DLQI. |
| **Source** | Medical record. |
| **Frequency** | Follow-up. |
| **Suggested target** | ≥60%. |

| **Indicator name** | **Sustained disease control** |
| --- | --- |
| **Dimension** | Outcome |
| **Operational definition** | Proportion of patients who maintain the therapeutic target at successive visits. |
| **Numerator** | Patients with sustained control. |
| **Denominator** | Patients who achieved the target. |
| **Source** | Medical record. |
| **Frequency** | Annual. |
| **Suggested target** | ≥70%. |

| **Indicator name** | **Treatment adherence** |
| --- | --- |
| **Dimension** | Outcome |
| **Operational definition** | Proportion of patients with adequate adherence to treatment. |
| **Numerator** | Adherent patients (specify a validated measurement method). |
| **Denominator** | Patients on treatment. |
| **Source** | Medical record / Pharmacy. |
| **Frequency** | Annual. |
| **Suggested target** | ≥80%. |

| **Indicator name** | **Safety monitoring** |
| --- | --- |
| **Dimension** | Outcome |
| **Operational definition** | Proportion of patients with complete safety monitoring during treatment. |
| **Numerator** | Patients with adequate monitoring. |
| **Denominator** | Patients on systemic or biologic therapy. |
| **Source** | Medical record. |
| **Frequency** | Semiannual. |
| **Suggested target** | ≥90%. |

| **Indicator name** | **Adverse event reporting** |
| --- | --- |
| **Dimension** | Outcome |
| **Operational definition** | Proportion of serious adverse events reported to pharmacovigilance. |
| **Numerator** | Serious adverse events reported. |
| **Denominator** | Serious adverse events identified. |
| **Source** | Pharmacovigilance system. |
| **Frequency** | Continuous. |
| **Suggested target** | 100%. |
